# Inhospital coagulation management and fluid replacement therapy in patients with multiple and/or severe injuries – a systematic review and clinical practice guideline update

**DOI:** 10.1007/s00068-025-02919-2

**Published:** 2025-06-27

**Authors:** Heiko Lier, Käthe Goossen, Charlotte M. Kugler, Erwin Strasser, Björn Hussmann, Marc Maegele, Peter Hilbert-Carius

**Affiliations:** 1 Anaesthesiology and Pain Therapy, Mediapark Klinik, Cologne, Germany; 2 Institute for Research in Operative Medicine (IFOM), Witten/Herdecke University, Cologne, Germany; 3 Department of Transfusion Medicine, Cellular Therapeutics and Haemostaseology, LMU Munich University Hospital, Munich, Germany; 4 Department of Trauma Surgery, Hochsauerland Hospital, Arnsberg, Germany; 5 Department of Orthopaedics, Trauma Surgery, and Sports Traumatology, Cologne-Merheim Medical Centre (CMMC), Institute for Research in Operative Medicine (IFOM), Witten/Herdecke University, Cologne, Germany; 6 Department of Anaesthesiology, Intensive Care, Emergency Medicine, and Pain Therapy, Bergmannstrost Hospital, Halle, Germany

**Keywords:** Coagulation management, Fluid replacement therapy, Transfusion, Outcome, Polytrauma guideline

## Abstract

**Purpose:**

Our aim was to update the evidence-based and consensus-based recommendations for inhospital coagulation management and fluid replacement therapy in patients with multiple and/or severe injuries on the basis of current evidence. This guideline topic is part of the 2022 update of the German Guideline on the Treatment of Patients with Multiple and/or Severe Injuries.

**Methods:**

MEDLINE and Embase were systematically searched to May 2021. Further literature reports were obtained from clinical experts. Randomised controlled trials, prospective cohort studies, and comparative registry studies were included if they compared interventions for the prevention of acidaemia, hypocalcaemia and hypothermia, for coagulation management, fluid replacement therapy, blood product transfusions, viscoelastic assays, or central venous access in patients with multiple and/or severe injuries in the hospital setting. We considered patient-relevant clinical outcomes, such as mortality and bleeding control, or coagulation parameters as surrogate outcomes. Risk of bias was assessed using NICE 2012 checklists. The evidence was synthesised narratively, and expert consensus was used to develop recommendations and determine their strength.

**Results:**

Fifty-nine new studies were identified. Interventions covered were blood products (*n* = 19 studies), coagulation management (*n* = 14), viscoelastic assays (*n* = 12), temperature management (*n* = 5), fluid replacement therapy (*n* = 4), base excess/lactate (*n* = 3), calcium (*n* = 1), and intravenous access (*n* = 1). Twelve recommendations were modified, and seven additional recommendations were developed. All achieved strong consensus.

**Conclusion:**

The key recommendations are summarised as follows.

- Trauma-induced coagulopathy (TIC) is a distinct clinical entity requiring early diagnostic and therapeutic interventions.
- Perform viscoelastic assays in order to aid in the diagnosis and treatment of TIC in severely bleeding trauma patients.
- Since only approximately 20% of trauma patients are hyperfibrinolytic and tranexamic acid is not beneficial in the absence of hyperfibrinolysis, TXA should not be indiscriminately used in all patients in the emergency department.
- Coagulation factor concentrates as well as TXA are indicated in patients with life-threatening haemorrhage and/or haemorrhagic shock.

**Supplementary Information:**

The online version contains supplementary material available at 10.1007/s00068-025-02919-2.

## Introduction

Since the 2016 version of this guideline, our understanding of abnormal coagulation after severe trauma or, in other words, trauma-induced coagulopathy (TIC) has grown considerably. In this article, we review the pathophysiology, diagnosis and management of TIC.

Our aim was to update the evidence-based and consensus-based recommendations for inhospital coagulation management and fluid replacement therapy in patients with multiple and/or severe injuries on the basis of current evidence.

## Methods

This guideline topic is part of the 2022 update of the German Guideline on the Treatment of Patients with Multiple and/or Severe Injuries [1]. The guideline update is reported according to the RIGHT tool [2], the systematic review part according to the Preferred Reporting Items for Systematic Reviews and Meta-Analyses (PRISMA) 2020 reporting guideline [3]. The development and updating of recommendations followed the standard methodology set out in the guideline development handbook issued by the Association of the Scientific Medical Societies in Germany (AWMF) [4]. All methods were defined a priori, following the methods report of the previous guideline version from July 2016 [5] with minor modifications, as detailed below. This work is based on the corresponding chapter of the German Polytrauma Guideline [1]. Publication as a systematic review has the advantage that, in contrast to the full guideline, the relevant parts of the method report, the guideline chapter and the evidence tables are directly related to each other. As a result, the reader gets a clear overview of all these aspects in one work. This approach was chosen to increase the dissemination and improve implementation of the guideline content overall.

### PICO questions and eligibility criteria

Population, intervention, comparison, and outcome (PICO) questions were retained from the previous guideline version. We added PICO questions on inhospital fluid replacement therapy, which had previously been addressed in Sect. 1.3 (Volume Replacement) of the 2016 guideline. In addition, the participating professional societies involved in guideline development were asked to submit new PICO questions. The overarching PICO question for this topic area was:

*In adult patients (≥ 14 years) with known or suspected polytrauma and/or severe injuries*,* does a specific inhospital approach to coagulation management*,* fluid replacement therapy*,* or blood product transfusions improve patient-relevant outcomes compared to any other intervention?*

The full set of predefined PICO questions is listed in Table S1 (Online Resource 1). The study selection criteria in the PICO format are shown in Table 1.

**Table 1:** Predefined selection criteria

PICO element	Selection criteria
Population:	adult patients (≥ 14 years) with polytrauma and/or severe injuries^a, b^
**Intervention** **/comparison**:	• inhospital diagnosis of coagulation status, or coagulation therapy (incl. administration of blood products, diagnosis and management of acidosis, base excess/lactate, hypocalcaemia, temperature management), *or* • inhospital management of severe bleeding, massive transfusion, or thromboprophylaxis, *or* • inhospital diagnosis of volume status or volume therapy, *or* • inhospital central venous catheter placement
**Outcomes**:	any patient-relevant clinical outcomes, such as mortality and bleeding control; if unavailable, coagulation parameters as surrogate outcomes
**Study type**:	• comparative, prospective studies (randomised controlled trials, cohort studies) • comparative registry^c^ data (incl. case-control studies) • systematic reviews based on the above primary study types
**Language**:	English or German
**Other inclusion criteria**:	• full text of study published and accessible • study matches predefined PICO question
**Exclusion criteria**:	• multiple publications of the same study without additional information • study already included in previous guideline version

^a^ Defined by an Injury Severity Score (ISS) > 15, Glasgow Coma Scale (GCS) < 9, or comparable values on other scales, or, in the prehospital setting, clinical suspicion of polytrauma/severe injury with a need for life-saving interventions

^b^ For new PICO questions, indirect evidence from other populations was eligible for inclusion if direct evidence was unavailable.

^c^ Using the Agency for Healthcare Research and Quality (AHRQ) definition of registries [6].

### Literature search

An information specialist systematically searched for literature in MEDLINE (Ovid) and Embase (Elsevier). MEDLINE was used because it allowed the use of proximity operators in the search strategy. The search strategy described in the 2016 guideline update was used with minor modifications. It contained index (MeSH/Emtree) and free text terms for the population and intervention. Additional terms were included for new PICO questions. For the topic area of coagulation management and fluid replacement therapy, a combined search was conducted covering both the prehospital and hospital settings. All searches were completed on 7 May 2021. No start date was used in the searches for new PICO questions. For update searches, the start date was 1 January 2014. Table S2 (Online Resource 1) provides details for all searches. Studies referenced in the Methods section of included studies were also retrieved, and clinical experts were asked to submit additional relevant references. We did not search for grey literature or preprints.

### Study selection

Study selection was performed independently by two reviewers with > 10 years (KG) and 4 years (CK) of experience in conducting systematic reviews. We used a two-step process using the predefined eligibility criteria: (1) title/abstract screening of all references retrieved from database searches using Rayyan software [7] and (2) full-text screening of all articles deemed potentially relevant by at least one reviewer at the title/abstract level in Endnote (Endnote, Version: 20 [Software], Clarivate, Boston, Massachusetts, USA, https://endnote.com/). Studies limited to the prehospital setting were excluded during full-text screening. Disagreements were resolved through consensus or by consulting a third reviewer (HL, a clinical expert). The reasons for full-text exclusion were recorded (Table S3, Online Resource 1).

### Assessment of risk of bias and level of evidence

Two reviewers (KG, CK) sequentially assessed the risk of bias of included studies at study level using the relevant checklists from the NICE guidelines manual 2012 [8]. We assigned each study an initial level of evidence (LoE) using the Oxford Centre for Evidence-based Medicine Levels of Evidence (2009) [9]. For studies with post-hoc subgroup analyses, indirectness of the study population, or low power and imprecision of the effect estimate, the LoE was downgraded and marked with an arrow (↓). Any disagreements were resolved through consensus or by consulting a third reviewer.

### Data extraction and data items

Data were extracted into a standardised data table by one reviewer and checked by another (KG, CK). A predefined data set was collected for each study. This consisted of study characteristics (study type, aims, setting), patient selection criteria and baseline characteristics (age, gender, injury scores, other relevant variables), intervention and control group treatments (including important co-interventions), patient flow (number of patients included and analysed), matching/adjusting variables, and data on outcomes for any time point reported.

### Outcome measures

Outcomes were extracted as reported in the study publications. For prospective cohort studies and registry data, preference was given to data obtained after propensity-score matching or statistical adjustment for risk-modulating variables over unadjusted data.

### Synthesis of studies

Studies were grouped by interventions. An interdisciplinary expert group used their clinical experience to synthesise studies narratively by balancing beneficial and adverse effects extracted from the available evidence. Priority was given to reducing mortality, immediate complications, and long-term adverse effects. Clinical heterogeneity was explored by comparing inclusion criteria and patient characteristics at baseline as well as clinical differences in the interventions and co-interventions. We did not conduct formal meta-analyses due to the high clinical and methodological heterogeneity among included studies.

### Development and updating of recommendations

For each PICO question, the following updating options were available: (1) the recommendation of the preceding version remains valid and requires no changes (“confirmed”); (2) the recommendation requires modification (“modified”); (3) the recommendation is no longer valid or required and is deleted; (4) a new recommendation needs to be developed (“new”). An interdisciplinary expert group of clinicians with expertise in trauma surgery, transfusion, and anaesthesiology reviewed the body of evidence, drafted recommendations based on the homogeneity of clinical characteristics and outcomes, the balance between benefits and harms as well as their clinical expertise, and proposed grades of recommendation (Table 2). In the absence of eligible evidence, good practice recommendations were made in cases where the Guideline Group felt a statement was required due to the importance of the topic. These were based on clinical experience, data from studies with a lower level of evidence, and expert consensus. They were not graded, and instead labelled as good (clinical) practice points (GPP). For GPPs, the strength of a recommendation is presented in the wording shown in Table 2.

**Table 2:** Grading of recommendations

Symbol	Grade of recommendation	Description	Wording (examples)
⇑⇑	A	strong recommendation	“use…”, “do not use…”
⇑	B	recommendation	“should use…”, “should not use…”
⇔	0	open recommendation	“consider using…”, “… can be considered”

### Consensus process

The Guideline Group finalised the recommendations during a web-based, structured consensus conference on 15 March 2022 via Zoom (Zoom, Version: 5.x [Software], Zoom Video Communications, Inc., San José, California, USA, https://zoom.us). A neutral moderator facilitated the consensus conference. Voting members of the Guideline Group were delegates of all participating professional organisations, including clinicians, emergency medical services personnel and nurses. Guideline methodologists attended in a supporting role. Members with a moderate, thematically relevant conflict of interest abstained from voting on recommendations. Members with a high, relevant conflict of interest were not permitted to vote or participate in the discussion. Attempts to recruit patient representatives were unsuccessful. The included literature was made available to the Guideline Group in aggregate form. A member of the expert group presented recommendations. Following discussion, the Guideline Group refined the wording of the recommendations and modified the grade of recommendation as needed. Agreement with both the wording and the grade of recommendation was assessed by anonymous online voting using the survey function of Zoom. Abstentions were subtracted from the denominator of the agreement rate. Consensus strength was classified as shown in Table 3.

**Table 3:** Classification of consensus strength

Description	Agreement rate
strong consensus	> 95% of participants
consensus	> 75 to 95% of participants
majority approval	> 50 to 75% of participants
no approval	< 50% of participants

Recommendations were accepted if they reached consensus or strong consensus. For consensus recommendations with ≤ 95% agreement, diverging views by members of the Guideline Group were detailed in the background texts. Recommendations with majority approval were returned to the expert group for revision and further discussion at a subsequent consensus conference. Recommendations without approval were considered rejected.

### Peer review

During a four-week consultation phase, the recommendations and background texts were submitted to all participating professional organisations for review. Comments were collected using a structured review form. The results were then assessed, discussed and incorporated into the text by the guideline coordinator with the relevant author group.

The guideline was adopted by the executive board of the German Trauma Society on 17 January 2023.

### Quality assurance

The guideline recommendations were reviewed for consistency between guideline topic areas by the steering group. Where necessary, changes were made in collaboration with the clinical leads for all topic areas concerned. The final guideline document was checked for errors by the guideline chair and methodologist.

### Updating the guideline

An update of this guideline topic is planned within five years of its publication. For this update, we will use the GRADE approach [10].

## Results

**Fig. 1:**
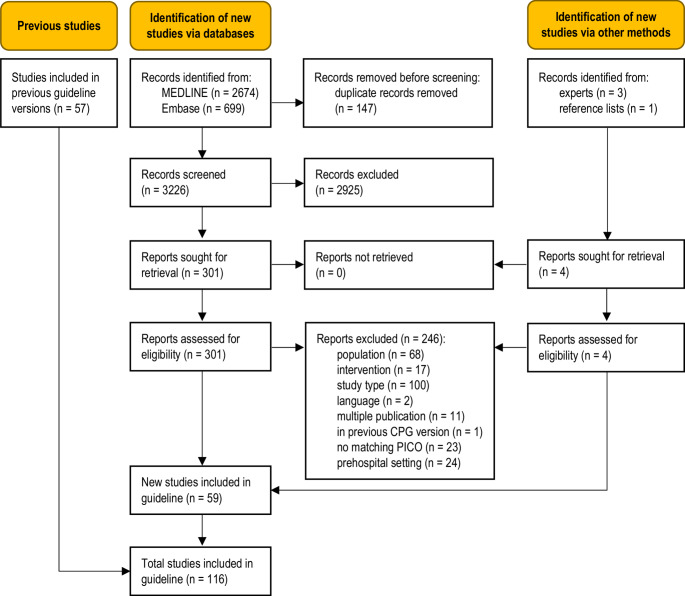
Modified PRISMA 2020 flow diagram showing the systematic literature search and selection of studies

The database searches identified 3226 unique records (Fig. 1). Four additional records were obtained from clinical experts and from the reference list of an included study. Fifty-nine new studies were eligible for this update [11–69], adding to the body of evidence from the 57 studies previously included in the guideline [70–126]. A total of 246 full-text articles were excluded (Table S3, Online Resource 1).

### Characteristics of studies included in this update

Study characteristics, main outcomes, levels of evidence, and risk-of-bias assessments are presented in Table 4. Full details are provided in Table S4, Online Resource 1. This update included twenty RCTs [11–30], six secondary analyses of RCT data [31–36], nine prospective cohort studies [37–45], thirteen cross-sectional studies [46–58], and eleven comparative registry studies [59–69]. Thirty-two studies were performed in North America, ten in Europe, ten in Asia, one in Australia, five were international multi-centre studies, and one was performed in a military setting. Eligible patient populations were adults with severe injuries, mostly with severe bleeding or known/suspected haemorrhagic shock. Some studies were limited to subpopulations such as patients with traumatic brain injury [17–19, 23, 24, 37, 49, 69], patients with abdominal trauma [20, 45], or pelvic fractures [44].

**Table 4:** Characteristics of studies included in the update (see Table S4, online resource 1 for details)

Study, ref., design	Population	Index/reference test or interventions (*N* patients)	Main outcomes (selection)^*^		LoE, risk of bias (RoB)^§^, comments
*Viscoelastic assays*					
Albert 2019 [49] Cross-sectional study	Patients with isolated TBI^a^	Index: thromboelastography (TEG) Reference: conventional coagulation tests (CCT) (*N* = 58 development cohort, *N* = 39 validation cohort)	**Diagnosis of acute coagulopathy** *Sensitivity*,* % (95% CI)* κ-time: 64 (45.8–79.3) α-angle: 62 (44.0–77.3) κ-time + α-angle: 63 (45.3–77.1) *Specificity*,* % (95% CI)* κ-time: 46 (21.3–72.0) α-angle: 40 (16.8–68.7) κ-time + α-angle: 43 (15.8–75.0)		LoE 2b Unclear RoB Limited to isolated severe TBI population patients, multiply injured patients excluded
Baksaas-Aasen 2021 [11] RCT	Patients with major trauma haemorrhage	CCT (*N* = 195 in ITT analysis) VHA: viscoelastic haemostatic assay (*N* = 201 in ITT analysis)	*6-hour mortality*,* OR (95% CI)* CCT vs. VHA: 0.97 (0.52–1.80)		LoE 1b Unclear RoB Study powered to detect a 15% reduction in death/MT
Balendran 2017 [50] Cross-sectional study	Patients with trauma haemorrhage	Tests at admission: prothrombin time (PT) EXTEM CT EXTEM MCF *(N* = 358 UK, *N* = 331 Austria)	**Prediction of 24-hour mortality** *AUC (95% CI)* *UK*: PT: 0.90 (0.82–0.97) EXTEM CT: 0.66 (0.48–0.82) EXTEM MCF: 0.81 (0.66–0.96) *A*: PT: 0.78 (0.68–0.89) EXTEM CT: 0.74 (0.62–0.86) EXTEM MCF: 0.67 (0.54–0.81)		LoE 2b No RoB tool prespecified^b^ Patients taking anticoagulants excluded
Barrett 2020 [51] Cross-sectional study	Patients with trauma activation^c^	Index 1: plasmin TEG Index 2: tissue plasminogen activator-challenged TEG Reference: TEG without exogenous additives (*N* = 148 analysed)	**Diagnosis of hyperfibrinolysis** *Sensitivity*,* % (95% CI)* P-TEG (rTEG LY30 > 3%): 0.19 (0.08–0.37) P-TEG (rTEG LY30 > 7.6%): 0.31 (0.14–0.56) tPA TEG TMA (rTEG LY30 > 3%): 0.50 (0.31–0.69) tPA TEG TMA (rTEG LY30 > 7.6%): 0.77 (0.50–0.92) *Specificity % (95% CI)* P-TEG (rTEG LY30 > 3%): 0.88 (0.81–0.92) P-TEG (rTEG LY30 > 7.6%): 0.89 (0.82–0.93) tPA TEG TMA (rTEG LY30 > 3%): 0.87 (0.79–0.92) tPA TEG TMA (rTEG LY30 > 7.6%): 0.87 (0.80–0.92)		LoE 2b Unclear RoB Patients taking anticoagulants excluded
Cohen 2019 [52] Cross-sectional study	Military trauma patients undergoing DCR	Index: definition of ATC using an integrated ROTEM model Reference: established definition of ATC (*N* = 40 analysed)	**Prediction of need of MT** *Sensitivity: %* Index test: 86 Reference test: 64 *Specificity: %* Index test: 38 Reference test: 50		LoE 2b No RoB tool prespecified^b^ Small sample size, no measure of variance provided, military setting
Connelly 2017 [53] Cross-sectional study	Trauma patients at risk for coagulopathy and haemorrhage^d^	MA: Multiplate aggregometry aspirin area under the platelet aggregation curve (ASPI AUC) TEG-PM: TEG Platelet Mapping percent inhibition of arachidonic acid VN: VerifyNow Aspirin Reaction Units (ARU) (*N* = 64)	**Detection of antiplatelet therapy** *Sensitivity: %* MA: 80 TEG-PM: 56 VN: 100 *Specificity: %* MA: 92 TEG-PM: 92 VN: 70		LoE: 2b High RoB for conduct of index test Small sample size, no measure of variance provided, test cut-offs not yet validated, patients on oral anticoagulants excluded
Gonzalez 2016 [12] RCT	Injured patients with MTP activation^e^	IG: MTP goal directed by point-of-care TEG (*N* = 56) CG: MTP goal directed by CCT (*N* = 55)	*28-day mortality*,* HR (95% CI)* CG vs. IG: HR 2.17 (1.04–4.58)		LoE: 1b High risk of performance bias (lack of blinding)
Hagemo 2015 [56] Cross-sectional study	Patients requiring full trauma team activation	EXTEM: citrated sample recalcified before TF activation FIBTEM: cytochalasin D added for platelet inhibition to isolate the fibrin component of the clot CCT: INR, fibrinogen, platelet count (*N* = 808)	**Diagnosis of acute coagulopathy** *Sensitivity: %* EXTEM CA5 ≤ 37 mm: 66.3 (55.1–76.3) FIBTEM CA5 ≤ 8 mm: 67.5 (55.9–77.8) Fibrinogen ≤ 1.61 g/L: 73.6 (63.0–82.4) Platelet ct ≤ 199 × 109/L: 61.7 (46.4–75.5) *False positive rate*,* % (95% CI)* EXTEM CA5 ≤ 40 mm: 18.8 (15.9–21.9) FIBTEM CA5 ≤ 8 mm: 20.7 (17.7–23.9) Fibrinogen ≤ 1.61 g/L: 11.5 (9.2–14.1) Platelet ct ≤ 199 × 109/L: 29.9 (26.6–33.4)		LoE: 2b High RoB for conduct of index test Non-consecutive recruitment, test cut-offs not yet validated, patients on oral anticoagulants excluded
Moore 2017 [58] Cross-sectional study	Patients requiring highest level of trauma activation	R-TEG; INR; SI; ABC; TASH Lt-TEG: r-TEG with low tPA dose Ht-TEG: TEG with high tPA dose (*N* = 324)	**Prediction of MT > 4U RBC** *Sensitivity/specificity*,* %* Lt-LY30: 84/82 Lt-TMA: 67/85 Ht-LY30: 80/84 Ht-TMA: 84/70 R-TEG MA: 68/79 INR: 90/69 TASH: 87/76 SI: 63/77 ABC: 85/37		LoE: 2b No RoB tool prespecified^b^ Non-standard outcome definition for massive transfusion, no measure of variance provided, test cut-offs not yet validated
Peng 2019 [46] Cross-sectional study	Severe trauma patients^f^	TEG FF MA: maximum amplitude by standard functional fibrinogen TEG test TEG FIBTEM MA: MA by crossover test using ROTEM reagents on TEG ROTEM FIBTEM MCF: maximum clot firmness by ROTEM FIBTEM ROTEM EXTEM MCF: maximum clot firmness by ROTEM EXTEM (*N* = 45)	**Diagnosis of hypofibrinogenemia** *AUC (95% CI)* TEG FF MA: 0.95 (0.89–1.00) TEG FIBTEM MA: 0.95 (0.89–0.997) ROTEM FIBTEM MCF: 0.96 (0.90–1.00) ROTEM EXTEM MCF: 0.92 (0.83–1.00) **Diagnosis of coagulopathy** *AUC (95% CI)* TEG FF MA: 0.56 (0.48–0.63) TEG FIBTEM MA: 0.53 (0.46–0.61) ROTEM FIBTEM MCF: 0.56 (0.49–0.64) ROTEM EXTEM MCF: 0.61 (0.54–0.68)		LoE: 3b High RoB for conduct of index test Limited to AUC values, no test cut-offs derived within study, patients on oral anticoagulants excluded
Rizoli 2016 [47] Cross-sectional study	Severely injured patients^g, h^	TEG MA EXTEM MCF FIBTEM MCF (*N* = 33)	**Diagnosis of hypofibrinogenemia** *AUC (95% CI)* TEG MA: 0.74 (0.53–0.96) EXTEM MCF: 0.55 (0.29–0.81), FIBTEM MCF: 0.56 (0.35–0.77) **Diagnosis of coagulopathy** *AUC (95% CI)* TEG MA: 0.60 (0.45–0.74) EXTEM MCF: 0.57 (0.42–0.71) FIBTEM MCF: 0.60 (0.45–0.74)		LoE: 3b High RoB for conduct of index test Limited to AUC values, no test cut-offs derived within study, patients on oral anticoagulants excluded
Spagnolello 2020 [48] Cross-sectional study	Major trauma patients thought to be bleeding	EXTEM A5 < 35 mm clot firmness iTACTIC ROTEM algorithm thresholds, see Hagemo 2015 RIE A5 Edinburgh ROTEM, clot firmness after 5 min. RIE A10 Edinburgh ROTEM, clot firmness after 10 min. CCT (INR > 1.5; fibrinogen ≤ 1.5 g/L; platelet count ≤ 50 or ≤ 100) (*N* = 57)	**Prediction of MT** *Sensitivity: % (95% CI)* EXTEM A5 < 35 mm: 54.5 (23.3–83.2) iTACTIC: 100.0 (71.5–100.0) RIE A5: 36.3 (10.9–69.2) RIE A10: 45.4 (16.7–76.6) CCT: 54.5 (23.3–83.2) *Specificity: % (95% CI)* EXTEM A5 < 35 mm: 65.2 (49.7–78.6) iTACTIC: 23.9 (12.6–38.7) RIE A5: 93.4 (82.1–98.6) RIE A10: 93.5 (82.1–98.6) CCT: 82.6 (68.6–92.2)		LoE: 2b No RoB tool prespecified^b^ Wide confidence intervals, potentially non-consecutive recruitment, patients on oral anticoagulants excluded
*Fluid replacement therapy*					
Carrick 2016 [13] RCT	Penetrating trauma patients^i^	IG: LMAP (*N* = 89) CG: HMAP (*N* = 91)	*30-day mortality*,* n/N (%)* 18/84 (21.4) vs. 21/80 (26.3), *p* = 0.47	LoE: 2b↓ Low RoB Study underpowered due to early termination (imprecision)	
Gu 2020 [14] RCT	Patients with traumatic haemorrhagic shock^j^	IG: restricted fluid resuscitation (*N* = 80) CG: routine fluid resuscitation (*N* = 80)	*Death: n (%)* 5 (6.3) vs. 13 (16.3), *p* = 0.045	LoE: 1b Unclear RoB Insufficient information reported	
Han 2015 [15] RCT	Trauma patients in hypovolemic shock^k^	IG1: 3% HSS (*N* = 82) IG2: 7.5% HSS (*N* = 80) CG: LRS (*N* = 84)	*24-hour survival* In IG1 and IG2 “better” than in CG but difference n.s. *Coagulopathy*,* n (%)* IG1: 0 vs. CG: 9 (10.7), *p* < 0.001 IG2: 2 (2.5) vs. CG: 9 (10.7), *p* < 0.001	LoE: 1b Unclear RoB Insufficient information reported, mortality endpoint not reported numerically	
Lu 2018 [16] RCT	Admission to ICU due to severe multiple injuries^g, l^	IG: fluid resuscitation to MAP 40–50 mmHg with 7.5% NaCl/plasma (*N* = 82) CG: conventional fluid resuscitation to MAP 60–80 mmHg (*N* = 82)	*Recovery time [min]*,* mean ± SD * IG: 89.7 ± 25.2, *p* = 0.00 CG: 193.5 ± 38.7 *Fatality rate*,* n/N (%)* IG: 2/82 (2.4), *p* = 0.041 CG: 15/82 (18.3)	LoE: 1b Unclear RoB Insufficient information reported	
*Base excess/lactate*					
Fligor 2017 [54] Cross-sectional study	Patients requiring highest level of trauma activation	PTH: parathyroid hormone LA: lactic acid (*N* = 46)	**Transfusions within 24 h** *AUC* PTH: 0.876 vs. LA: 0.793 *Sensitivity/specificity [%]* PTH ≥ 100 pg/mL: 88/86		LoE: 3b No RoB tool prespecified^b^ Test cut-offs not yet validated, majority of patients not severely injured (indirectness)
Gale 2016 [55] Cross-sectional study	Patients with severe blunt trauma^m^	iBD: initial base deficit iLA: initial lactate (*N* = 1829)	**Prediction of inhospital mortality** *adj. OR (95% CI) for each unit increase* iBD: 1.0 (1.0–1.1) iLA: 1.2 (1.1–1.2)		LoE: 3b↓ No RoB tool prespecified^b^ Limited to AUC values, no test cut-offs derived within study, retrospective data analysis
Hutchings 2018 [57] Cross-sectional study	Patients with traumatic haemorrhagic shock^n^	PVD: perfused vessel density by IDF videomicroscopy in the tongue MFI: microcirculatory flow index by IDF videomicroscopy LA: highest lactate prior to ICU (*N* = 58)	*Prediction of MODS at day 7*,* AUC* PVD: 0.87 (0.76–0.99) MFI: 0.83 (0.71–0.95) LA: 0.69 (0.53–0.84)		LoE: 3b↓ No RoB tool prespecified^b^ Limited to AUC values, no test cut-offs derived within study
*Temperature management*					
Cooper 2018 [17] RCT	Patients with severe TBI^o^	IG: hypothermia, 33 ± 0.5 °C (*N* = 240/260 for ITT analysis Primary/secondary outcome) CG: normothermia, 37 ± 0.5 °C (*N* = 226/239 for ITT analysis Primary/secondary outcome)	*GOS-E score 5–8 at 6 m*,* n/N (%)* 117/240 (48.8) vs. 111/226 (49.1) *Inhospital mortality*,* n/N (%) * 52/260 (20.0) vs. 43/239 (18.0) RR (95% CI): 1.11 (0.77–1.60)		LoE: 1b Unclear RoB Higher median ISS in IG, 32% of IG patients did not reach target temperature
Hifumi 2017 [18] RCT	Patients with coagulopathy following severe TBI^p^	IG: mild therapeutic hypothermia, 32–34 °C (*N* = 20) CG: normothermia, 35.5–37 °C (*N* = 12)	*Favourable GOS at 6 m*,* n/N (%) * 7/20 (35) vs. 4/12 (33.3), *p* = 1.00 *Survival at 6 m: n/N (%)* 12/20 (60.0) vs. 6/12 (50.0), *p* = 0.72		LoE: 2b Unclear RoB Post-hoc secondary analysis, underpowered for mortality (imprecision)
Maekawa 2015 [19] RCT	Patients with severe TBI^p^	IG: mild therapeutic hypothermia (*N* = 98) CG: fever control (*N* = 50)	*Mortality at 6 m: n/N (%); RR (98% CI)* 33/94 (35) CG: 11/48 (23) RR 1.8 (0.8–4.0)		LoE: 1b Unclear RoB Recruitment stopped early, possibly underpowered
Quine 2021 [37] Prospective cohort study	TBI patients undergoing therapeutic hypothermia	IG: induced hypothermia, 33 °C (*N* = 10) CG: patients with diagnosis at 37 °C prior to induced hypothermia (*N* = 10)	*R time [s]*,* mean ± SD* 7.57 ± 2.6 vs. 6.8 ± 1.7, *p* = 0.41 *α-angle [°]*,* median (IQR)* 69.2 (63.5–69.9) vs. 72.0 (68.7–73.5) *MA [mm]*,* mean ± SD* 73.9 ± 3.5 vs. 73.1 ± 4.4, *p* = 0.79		LoE: 3b↓ Unclear RoB Small sample size, no missing patient-relevant outcomes (indirectness)
Zhou 2018 [20] RCT	Severe abdominal trauma patients with hemorrhagic shock^g, q^	IG: phased temperature management (*N* = 45) CG: routine body temperature treatment (*N* = 45)	*Mortality*,* n (%)* 1 (2.2) vs. 6 (13.3), *p* = 0.049 *Complication rate: n (%) * 3 (6.7) vs. 12 (26.7), *p* = 0.011		LoE: 1b Unclear RoB Very limited reporting
*Calcium*					
Moore 2020 [31] Secondary analysis of two RCTs	Patients with traumatic haemorrhagic shock	IG: 2 units of universal donor thawed plasma (*N* = 76) CG: normal saline with or without RBCs if required (*N* = 84)	*Hypocalcemia: n (%)* 40 (52.6) vs. 30 (35.7) adj. RR (95% CI): 1.5 (1.0–2.1)		LoE: 2b↓ High risk of performance bias Effect of hypocalcemia on treatment decisions not investigated (indirectness)
*Blood products*					
Akbari 2018 [21] RCT	Severe blunt trauma patients (ISS > 16)	Fib: 2 g fibrinogen (*N* = 30) FFP: ≥2 units of FFP (*N* = 30) CG: neither (*N* = 30)	*Mortality during hospital stay*,* n (%) * Fib: 3 (10.0) vs. FFP: 11 (36.7) vs. CG: 11 (36.7), *p* = 0.029		LoE: 1b Unclear RoB Limited reporting, small study size
Bui 2016 [38] Prospective cohort study	Trauma patients requiring massive transfusion^r^	IG: FFF: PRBC < 1:1.5 (*N* = 54) CG: FFP: PRBC > 1:1.5 (*N* = 49)	**Unadjusted analysis** *Mortality: n/N (%)* 17/54 (31.5) vs. 7/49 (14.3), *p* = 0.042		LoE: 2b Unclear RoB Transfusion volume differed between groups
Cardenas 2018 [59] Registry study	Patients requiring highest level of trauma activation	IG: platelets (1:1:1 group) (*N* = 137) CG: no platelets (1:1:2 group) (*N* = 124)	**Analysis adjusted for plasma volume** *24-hour mortality*,* n (%) * 8 (5.8) vs. 21 (16.9), p_adj_<0.01 *30-day mortality*,* n (%)* 13 (9.5) vs. 25 (20.2), p_adj_<0.01		LoE: 2b High risk of selection bias Transfusion volume differed between groups, adjusted analyses
Chehab 2021 [60] Registry study	Trauma patients receiving early plasma transfusions	IG: FFP within 24 h of ED (*N* = 214) CG: never-frozen liquid plasma within 24 h of ED (*N* = 107)	**Matched cohort analysis** *24-hour mortality: n (%)* 8 (3.7) vs. 3 (2.8), *p* = 0.664		LoE: 2b Unclear RoB Majority of patients excluded during matching
De Roulet 2020 [61] Registry study	Patients requiring highest level of trauma activation and massive transfusion	IG: group A emergency-release plasma (ERP) (*N* = 122) CG: group AB ERP (*N* = 462)	**Multivariable regression** *30-day mortality*,* n (%)* 30 (24.6) vs. 111 (24.0), *p* = 0.90 adj. HR (95% CI): 1.15 (0.91–1.45)		LoE: 2b High risk of selection bias Analysis adjusted for important confounders
Innerhofer 2017 [22] RCT	Severe trauma patients^g^	CFC: coagulation factor concentrates (*N* = 50 in modified ITT analysis) FFP: fresh frozen plasma (*N* = 44 in modified ITT analysis)	*Multiple organ failure*,* OR (95% CI)* FFP vs. CFC: 1.92 (0.78–4.86) *Inhospital mortality*,* OR (95% CI)* FFP vs. CFC: 0.43 (0.04–2.82)		LoE: 1b Low RoB Low event rate for mortality, trial terminated early
Jones 2017 [62] Registry study	Patients with major trauma^g^	IG: PRBC: FFP and PRBC: PLT ratios close to 1 (0.5 to 1.5) CG: ratios other than 0.5–1.5 (*N* = 1538 overall)	**Multivariable regression** *Inhospital inflammatory complications*,* adj. HR (95% CI)* PRBC: FFP vs. CG: 1.07 (0.9–1.3) PRBC: PLT vs. CG: 0.97 (0.8–1.2)		LoE: 2b High risk of attrition bias for time outcome Analysis adjusted for important confounders
Jones 2014 [63] Registry study	Patients with major trauma (ISS > 25)	WBT: whole blood transfusion (*N* = 83) BCT: blood component transfusion (PRBC + PLT) (*N* = 1662)	**Multivariable regression** *Inhospital mortality*,* adj. OR (95% CI)* BCT vs. WBT: 3.2 (1.3–7.6), *p* = 0.010		LoE: 2b Unclear RoB Baseline imbalance, adjusted analysis
Kutcher 2014 [39] Prospective cohort study	Patients requiring highest-level trauma activation^s^	IG: low ratio RBC: FFO (*N* = 91) CG: high ratio RBC: FFP (*N* = 52)	**Unadjusted analysis** *Mortality (%)* 42.9 vs. 55.8, *p* = 0.165		LoE: 2b High risk of selection bias Unadjusted analysis
Nederpelt 2019 [64] Registry study	Trauma patients^t^	FFP: PRBC 1:1: (*N* = 1392) 1:2: (*N* = 1801) 1:3: (*N* = 492) 1:4: (*N* = 190) 1:5: (*N* = 79) 1:6: (*N* = 51) 1:6+: (*N* = 422)	**Multivariable regression** *24-hour mortality*,* adj. OR (95% CI)* 1:1 ratio as reference 1:2: 1.2 (1.0–1.5) 1:3: 1.62 (1.2–2.1) 1:4: 2.11 (1.4–3.1) 1:5: 4.11 (2.3–7.3) 1:6: 2.98 (2.3–6.1) 1:6+: OR 1.25 (0.9–1.7)		LoE: 2b Unclear RoB Large sample size, adjusted analysis
Pusateri 2020 [32] Secondary analysis of 2 RCTs	Trauma patients in/at risk for hemorrhagic shock^u^	IG: prehospital plasma (*N* = 297) CG: standard care (crystalloids) (*N* = 329)	**Adjusted analysis (age**,** ISS)** *24-hour mortality*,* adj. HR (95% CI)* *transport ≤ 20 min*: 1.89 (0.65–5.40) *transport > 20 min*: 0.53 (0.34–0.82)	LoE 2b↓ High risk of performance bias Post-hoc subgroup analysis	
Reitz 2020 [33] Secondary analysis of 2 RCTs	Trauma patients in/at risk for hemorrhagic shock^u^	IG: prehospital plasma (N = n.r.) CG: standard care (crystalloids) (N = n.r.)	**Multivariable regression** *24-hour mortality*,* HR (95% CI)* *Blunt*: 0.59 (0.370–0.947) *Penetrating*: 1.16 (0.430–3.103)	LoE 2b↓ High risk of performance bias Post-hoc subgroup analysis	
Roquet 2019 [65] Registry study	Patients with severe bleeding after trauma^v^	IG: high ratio, FFP-to-PRBC > 1:1.5 (*N* = 506) CG: low ratio, FFP-to-PRBC ≤ 1:1.5 (*N* = 391)	**Multivariable regression** *30-day mortality: adj. HR (95% CI)* 0.57 (0.33–0.97)		LoE: 2b Unclear RoB Attrition accounted for by imputation methods
Shea 2020 [40] Prospective cohort study	Patients with severe traumatic haemorrhage	IG: low-titer group O whole blood (*N* = 44) CG: component therapy (*N* = 42)	**Multivariable regression** *24-hour mortality: adj. OR (95% CI)* 0.81 (0.69–0.96)		LoE: 2b High risk of performance bias Interventions during consecutive time periods
Stanworth 2016 [41] Prospective cohort study	Trauma patients with major haemorrhage	IG: FFP: PRBC ratio < 1:2 (*N* = 92) CG: FFP: PRBC ratio ≥ 1:2 (*N* = 206)	**Unadjusted analysis** *24-hour mortality*,* n/N (%)* IG: 26/92 (28) vs. CG: 25/206 (12.1)		LoE: 2b High risk of selection bias Mortality analysis unadjusted, ISS balanced
Stevens 2017 [66] Registry study	Trauma patients requiring massive transfusion protocol	IG: compatible type A plasma (*N* = 1416) CG: incompatible type A plasma (*N* = 120)	**Multivariable regression** *28-day mortality*,* adj. OR (95% CI)* 1.00 (0.65–1.51)		LoE: 2b Unclear RoB Co-interventions vary, adjusted mortality analysis
Zeeshan 2019 [67] Registry study	Severe trauma patients	IG: 4-PCC + FPP (*N* = 234) CG: FPP alone (*N* = 234)	**Matched cohort analysis** *Inhospital mortality: n (%)* 41 (17.5) vs. 65 (27.7), *p* = 0.01		LoE: 2b Unclear RoB Large proportion of patients without match
Zhang 2019 [23] RCT	Patients with severe TBI	IG: 5 mL/kg of FFP (*N* = 20 analysed) CG: normal saline (5 mL/kg) (*N* = 32 analysed)	*6 m mortality*,* n (%)* 10 (50) vs. 15 (46.9), *p* = 1.000 RR (95% CI): 1.13 (0.37–3.47)		LoE: 2b↓ High risk of performance bias Small study size, substantial dropout rate (imprecision)
Zielinski 2015 [68] Registry study	Blunt trauma patients	IG: group A plasma (*N* = 115) CG: group AB plasma (*N* = 76)	**Multivariable regression** *Mortality: adj. OR (95% CI)* 0.66 (0.21–2.06)		LoE: 2b High risk of performance bias Different co-interventions
*Coagulation management: tranexamic acid*					
CRASH-3 collaborators 2019 [24] RCT	TBI patients^w^ treated ≤ 3 h of injury	IG: TXA (*N* = 6406) CG: 0.9% NaCl (*N* = 6331)	*Head injury-related 28-day mortality*,* RR (95% CI)* 0.94 (0.86–1.02) GCS 9–15: 0.78 (0.64–0.95) GCS 3–8: 0.99 (0.91–1.07)	LoE 1b Low RoB Good quality RCT, large sample size and event rate, pre-planned subgroup	
Guyette 2020 [25] RCT	Injured patients at risk for haemorrhage	IG: TXA (*N* = 488) CG: placebo (*N* = 456)	*30-day mortality*,* RR (95% CI)* RR (95% CI) 0.82 (0.60–1.11)		LoE: 1b Low RoB Good quality RCT
Khan 2018 [34] Secondary analysis of an RCT	Trauma patients with hyperfibrinolysis^x^	IG: TXA (*N* = 31) CG: no TXA (*N* = 62)	**Matched cohort analysis** *24 h mortality*,* %* 26 vs. 39, *p* = 0.25 *30-day mortality: %* 45 vs. 50, *p* = 0.82	LoE 2b Low RoB Matched for important confounders	
Moore 2017 [42] Prospective cohort study	Severely injured trauma patients	IG: TXA (*N* = 26) CG: no TXA (*N* = 206)	**Analysis adjusted for NISS** *Inhospital mortality*,* %*,* adj. p-value* *Hyperfibrinolysis*: 56 vs. 19, *p* = 0.116 *Shutdown*: 38 vs. 28, *p* = 0.597 *Physiologic fibrinolysis*: 63 vs. 11, *p* = 018	LoE 2b High risk of selection/performance bias Different co-interventions between groups	
Nishijima 2019 [26] Secondary analysis of an RCT	Adults with/at risk of significant traumatic bleeding ^y^	IG: TXA (*N* = 6753) CG: placebo (*N* = 6679)	*Oxford Handicap Score*,* on day 28 or at discharge*,* n (%)* Dependent: 807 (11.9) vs. 779 (11.7) Fully dependent: 421 (6.2) vs. 396 (5.9)		LoE: 1b Low RoB Prospective data analysis
Roberts 2017 [35] Secondary analysis of an RCT	Trauma patients with/at risk of significant bleeding^y^	IG: TXA (*N* = 10,060) CG: PBO (*N* = 10,067)	*Death due to bleeding*,* RR (95% CI)* TXA ≤ 1 h: 0.68 (0.57–0.82) TXA > 1 to ≤ 3 h: 0.79 (0.64–0.97) TXA > 3 h: 1.44 (1.12–1.84)	LoE 1b Low RoB Predefined subgroup analysis	
Roberts 2014 [36] Secondary analysis of an RCT	Trauma patients with/at risk of significant bleeding^y^	IG: TXA (*N* = 10,093) CG: PBO (*N* = 10,114)	*Death due to bleeding*,* HR (95% CI)* Day 0 since injury: 0.83 (0.73, 0.93) Day 1: 0.91 (0.79, 1.04) Day 2: 0.96 (0.77, 1.19) Day 3: 1.01 (0.76, 1.34)	LoE 1b Low RoB Exploratory subgroup analysis	
Spinella 2020 [27] RCT	Patients with severe traumatic bleeding^z^	IG1: 2 g of TXA (*N* = 49) IG2: 4 g of TXA (*N* = 50) CG: PBO (*N* = 50)	*28-day mortality n/N*,* (%)* CG: 6/49 (12.2) TXA 2 g: 5/44 (11.4) TXA 4 g: 4/48 (8.33), *p* = 0.8	LoE 2b↓ Low RoB Underpowered for mortality (imprecision)	
*Coagulation management: fibrinogen*					
Curry 2018 [28] RCT	Trauma patients with major haemorrhage	IG: 6 g of fibrinogen (*N* = 20) CG: 0.9% saline (*N* = 19)	*28-day all-cause mortality*,* % (95% CI)* 42.0 (25.2–64.0) vs. 29.2 (15.1–51.6)		LoE 2b↓ Low RoB Underpowered (imprecision)
Garrigue 2018 [29] RCT	Severe trauma patients	IG: French lyophilized plasma (*N* = 21) CG: fresh frozen plasma (*N* = 21)	*30-day inhospital mortality*,* n/N (%)* 5/21 (22) vs. 7/21 (29), *p* = 0.56		LoE: 1b High risk of performance bias
Nascimento 2016 [30] RCT	Severe trauma patients (blunt or penetrating)^$^	IG: lyophilized fibrinogen concentrate (FC, 6 g) (*N* = 21) CG: placebo (*N* = 24)	*28-day all-cause mortality: n/N (%)* 2/25 (8) vs. 3/24 (12.5) RR (95% CI) 2.4 (−0.2 to 23)		LoE 2b↓ High risk of attrition bias Underpowered for mortality (imprecision)
*Coagulation management VTE prophylaxis*					
Byrne 2016 [69] Registry study	Patients with isolated severe TBI^#^	IG: early VTE prophylaxis < 72 h (*N* = 1234) CG: late VTE prophylaxis > 72 h (*N* = 1234)	**Matched cohort analysis** *Mortality*,* adj. OR (95% CI)* 1.10 (0.84–1.45) *VTE*,* adj. OR (95% CI)* 0.48 (0.35–0.66)		LoE: 2b Unclear RoB Matched for important confounders
Schellenberg 2021 [44] Prospective cohort study	Pelvic fracture patients	IG: early VTE prophylaxis (≤ 48 h) (*N* = 74) CG: late VTE prophylaxis (> 48 h) (*N* = 72)	**Multivariable regression** *VTE*,* adj. OR (95% CI)* 0.65 (0.002–4.51)		LoE 3b↓ High risk of selection bias Higher ISS in control group, underpowered (imprecision)
Schellenberg 2019 [45] Prospective cohort study	Blunt solid-organ trauma patients	IG: early VTE prophylaxis (≤ 48 h) (*N* = 61) CG: late VTE prophylaxis (> 48 h) (*N* = 57)	**Unadjusted analysis** *Mortality: n (%)* 2 (3) vs. 1 (2), *p* = 1.000 *VTE: n (%)* 2 (3) vs. 6 (11), *p* = 0.153		LoE: 2b High risk of selection bias Unadjusted for baseline imbalance
*Intravenous access*					
Kunhahamed 2019 [43] Prospective cohort study	ED patients	IG: ultrasonography-guided technique (*N* = 35) CG: anatomical landmark (AL) technique (*N* = 35)	*Successful cannulation*,* n/N (%)* 35/35 (100) vs. 32/35 (91.4), *p* = 0.239		LoE 3b↓ Unclear RoB Population incl. few trauma patients (indirectness)

* Data for IG versus CG unless otherwise specified. ^§^ Risk of bias: low RoB = RoB low for all domains; unclear RoB = RoB unclear for at least one domain, no high RoB in any domain; for studies with high RoB, all domains with high RoB are named, with RoB low or unclear for all other domains (for full details Table S4, Online Resource 1); ^a^ GCS ≤ 8. ^b^ NICE 2012 checklists were used to determine RoB, which did not contain a RoB tool for prognostic studies. ^c^ GCS < 8 with presumed thoracic, abdominal or pelvic injury, *or* blunt trauma, *or* mechanically unstable pelvic injury, *or* penetrating injuries with injury to neck and/or torso with systolic blood pressure of < 90 mmHg. ^d^ GCS < 10 or ICH on initial head CT or SBP < 90 mmHg or base deficit > 6 mEq/L. ^e^ SBP < 70 mmHg or SBP 70–90 mmHg with heart rate > 107 bpm, penetrating wound or unstable pelvic fracture or abdominal ultrasound suspicious of bleeding. ^f^ SBP < 100 mmHg and uncrossmatched RBC transfusion < 30 min of arrival. ^g^ ISS ≥ 16 or NISS ≥ 15. ^h^ Significant bleeding, ABC ≥ 2, INR ≥ 1.2; ^i^ SBP ≤ 90 mmHg, in need of laparotomy or thoracotomy. ^j^ Patients who met the diagnostic criteria of haemorrhagic shock in the “Chinese emergency medicine expert consensus on diagnosis and treatment of traumatic hemorrhagic shock” issued by the Chinese College of Emergency Physicians in 2017. ^k^ Defined as out-of-hospital systolic blood pressure (SBP) of </≤70 mmHg or SBP 70/71 to 90 mmHg with a heart rate of ≥ 108 beats/min. ^I^ Average arterial pressure < 65 mmHg or systolic pressure < 40mmHg; ^m^ AIS > 2, ED arrival, SBP < 90mmHg or BD > 6mEg/L; ^n^ lactate concentration ≥ 2 mmol/L, were intubated and ventilated, enrolment as early as was feasible up to 12 h after ICU admission. ^o^ GCS < 9. ^p^ GCS: 4–8, ability to initiate cooling within 2 h after the onset of TBI; ^q^ body temperature < 35 °C, blood pH < 7.3; ^r^ defined as ≥ 10 units of PRBCs in ≤ 24 h. ^s^ SBP < 90, heart rate > 110, GCS < 8, subsequent ICU admission; ^t^ transfused ≥ 10 PRBCs and ≥ 1 FFP within 24 h; ^u^ hypovolaemic shock defined as out-of-hospital systolic blood pressure (SBP) of </≤70 mmHg, or SBP 70/71 to 90 mmHg with a heart rate of ≥ 108 beats/min; ^v^ defined as ≥ 4 PRBC units ≤ 6 h after admission; ^w^ patients with TBI defined as adults with GCS ≤ 12 or any intracranial bleeding; ^x^ defined as admission Ly30 ≥ 3% on thromboelastography; ^y^ SBP < 90 mmHg and/or heart rate > 110 beats/min; ^z^ patients who sustained a traumatic injury that required them to receive at least one unit of red blood cells (RBCs) or required an emergent operation for possible bleeding control; ^$^ identified as being at risk for significant haemorrhage as evidenced by systolic arterial pressure ≤ 100 mmHg and requiring uncrossmatched red blood cell (RBC) transfusion at any time from injury until 30 min after hospital arrival; ^#^ defined as head Abbreviated Injury Scale [AIS] ≥ 3 and Glasgow Coma Scale ≤ 8. For abbreviations and acronyms see list included.

### Risk-of-bias assessment for included studies and levels of evidence

The risk of bias was unclear for 21 studies that reported insufficient study details. Ten studies (RCTs or secondary analyses of RCT data) were judged to be of low risk of bias in all domains. The risk of selection bias was high in seven studies, nine were at high risk of performance bias, and two were at high risk of attrition bias.

The level of evidence was downgraded for 17 studies. Reasons for downgrading were post-hoc subgroup analyses (two studies), low power and imprecision of the effect estimate (seven studies), indirectness for four studies that included non-severe trauma patients or reported surrogate outcomes only, and limitations of diagnostic studies to reporting AUC values (four studies).

### Recommendations

Twelve recommendations were modified. Seven additional recommendations or good practice points were developed based on the updated evidence and expert consensus (Table 5). All achieved strong consensus. One recommendation from the 2016 Guideline was not retained in the 2022 update (Table S5, Online Resource 1).

**Table 5:** List of recommendations with grade of recommendation and strength of consensus

No.	GoR	New evidence, consensus	Recommendations	Status 2022	
*Trauma-induced coagulopathy*					
1	A ⇑⇑	– 100%	Trauma-induced coagulopathy is a distinct clinical entity with a considerable impact on survival. For this reason, perform diagnostic coagulation tests and initiate coagulation therapy in the resuscitation room, if not before.	Modified	
2	A ⇑⇑	– 100%	Perform early and serial basic diagnostic tests on severely injured, bleeding patients, i.e. arterial blood gas analysis, measurements of prothrombin time (PT), activated partial thromboplastin time (aPTT), fibrinogen levels, and platelet counts, and determine blood type.	Modified	
3	A ⇑⇑	[12, 46–48, 56, 58] 100%	Use viscoelastic assays for the diagnosis and treatment of trauma-induced coagulopathy at an early stage in the management of severely injured, bleeding patients in the resuscitation room.	Modified	
*Damage control resuscitation*					
4	B ⇑	– 100%	Permissive hypotension (mean arterial pressure [MAP] of approximately 65 mmHg, systolic arterial pressure of approximately 80 mmHg) should be the goal in actively bleeding patients until surgical bleeding control can be achieved.	Modified	
5	B ⇑	[14, 16] 100%	Patients (without a history of cardiopulmonary disease) in haemorrhagic shock should receive fluid resuscitation at a target MAP of approximately 65 mmHg before, during and soon (3–6 h) after surgery.	New	
6	B ⇑	– 100%	In patients with a combination of haemorrhagic shock and traumatic brain injury (GCS < 9) and/or spinal trauma with neurological signs and symptoms, the target MAP should be approximately 85 mmHg.	Modified	
7	A ⇑⇑	– 100%	Use serial lactate and/or base excess measurements to assess and manage the severity and treatment of shock.	Modified	
8	B ⇑	– 100%	Appropriate measures should be taken to avoid hypothermia and normalise body temperature.	Confirmed	
9	B ⇑	– 100%	Acidaemia should be prevented by using appropriate and early measures to treat shock.	Modified	
10	B ⇑	– 100%	Hypocalcaemia < 0.9 mmol/L should be avoided and the goal should be to achieve normal levels of calcium.	Confirmed	
*Replacement of coagulation-promoting substances*					
11	B ⇑	– 100%	A local protocol for massive transfusion and coagulation therapy should be available.	Modified	
12	B ⇑	– 100%	In actively bleeding patients, the decision to transfuse is made on a case-by-case basis and depends on clinical criteria, injury severity, the amount of blood loss, haemodynamic status, and oxygenation. After haemodynamic stabilisation, the goal should be to restore blood volume and to achieve a target haemoglobin (Hb) level of 7–9 g/dL [4.3–5.6 mmol/L].	Modified	
13	B ⇑	– 100%	If massive haemorrhage requires the replacement of plasma volume, therapeutic plasma should be used as early as possible.	Modified	
*Coagulation management*					
14	A ⇑⇑	[12, 46–48, 56, 58] 100%	Use viscoelastic assays to assess coagulation status and to guide treatment.	New	
15	B ⇑	[53] 100%	Diagnostic coagulation tests and coagulation therapy should be supplemented by platelet *function* assays.	New	
16	B ⇑	– 100%	If therapeutic plasma is used in massive transfusion, a ratio of 4:4:1 for units of therapeutic plasma to PRBCs to platelets should be aimed for.^*a*^ Otherwise, therapeutic plasma should be given in a restrictive manner.	Modified	
17	A ⇑	100%	Treat patients with life-threatening haemorrhage and/or in shock and patients with confirmed hyperfibrinolysis with 1 g of tranexamic acid (TXA) over ten minutes as early as possible/in the prehospital setting, followed, if necessary, by 1 g infused over eight hours.	Modified	
18	B ⇑	– 100%	Tranexamic acid treatment should not be initiated beyond three hours of injury (except in patients with confirmed hyperfibrinolysis).	Confirmed	
19	B ⇑	[27, 42] 100%	Since only approximately 20% of trauma patients present with hyperfibrinolysis and tranexamic acid (TXA) has adverse effects in the absence of hyperfibrinolysis, TXA should not be automatically given to every injured patient.	New	
20	A ⇑⇑	[21, 22, 30] 100%	Additionally, administer fibrinogen (at an initial dose of 3–6 g or 30–60 mg/kg) to patients with life-threatening haemorrhage and/or in shock.	New	
21	B ⇑	[22, 67] 100%	In addition to fibrinogen, prothrombin complex concentrates (PCCs) should be administered to patients with life-threatening haemorrhage and/or in shock.^*b*^	New	
22	GPP	100%	Make decisions on what method of thrombosis prevention to use and when to initiate thromboprophylaxis within 24 h of bleeding control.	Confirmed	
23	GPP	92%	Use ultrasonography (if immediately available) to guide the placement of central venous catheters.	New	

*aPTT *activated partial thromboplastin time, *GCS *Glasgow Coma Scale, *GoR *grade of recommendation, *GPP* good (clinical) practice point, *Hb *haemoglobin, *MAP *mean arterial pressure, *PCC *prothrombin complex concentrate, *PT *prothrombin time, *PRBC *packed red blood cell, *SBP *systolic blood pressure, *TXA *tranexamic acid. ^*a*^ adaptation of PROPPR-data with US-single donor platelet concentrates to German pool or apheresis-platelet concentrates with 2 × 10^11^ platelets. ^*b*^ In Germany, only 4 factor-PCCs are approved

## Discussion

### Rationale for recommendations

Trauma-induced coagulopathy (TIC) results from a combination of cellular hypoperfusion, i.e. a lack of oxygen at the level of microcirculation (which appears to be the key factor), and sufficiently severe tissue damage. Hypoxia releases tissue plasminogen activator (tPA) from endothelial cells [127]. Shock can thus lead to coagulopathy. Other factors, such as age and sex, are implicated in the response to trauma [128]. Physiologically, trauma leads to temporary activation of fibrinolysis, which then rapidly decreases. Apart from hy*per*fibrinolysis (persistent and excessive activation), fibrinolysis shutdown (low fibrinolytic activity after previous fibrinolysis activation), and hyp*o*fibrinolysis (low fibrinolytic activity without evidence of previous fibrinolysis activation) can occur [129, 130]. This is a time-dependent development, i.e. fibrinolytic activity changes within a few hours [130]. Other studies demonstrated that approximately 20% of patients were hyp*er*fibrinolytic (mortality: >40%) and almost 20% had physiologic fibrinolysis (low mortality). Most patients (approximately 60%), however, developed fibrinolysis shutdown (mortality: approximately 20%) [131, 132]. Damage to the endothelial glycocalyx is not polytrauma-specific but is caused by ischaemia and triggers local and systemic inflammatory mediator release [133]. Shock-induced endotheliopathy and glycocalyx degradation lead to hyperfibrinolysis as a key component of primary endogenous coagulopathy (acute traumatic coagulopathy, ATC), which is an important cause of early mortality in the first hours after injury. Secondary coagulopathy is the result of iatrogenic interventions and treatment, which lead to an increase in bleeding. Patients die within 24 h or transition into another fibrinolysis phenotype (prothrombotic coagulopathy) [134]. TIC is a component of the lethal triad of coagulopathy, hypothermia, and acidosis. In recent years, this triad has become the diamond of death, which now includes hypocalcaemia [135].

Permissive hypotension is a strategy that is used for two reasons. First, a lower than normal blood pressure is tolerated or targeted in order to promote thrombus formation. Second, fluid administration is restricted in order to prevent iatrogenic dilution, while enough perfusion to end organs is still provided. Permissive hypotension as a component of damage control resuscitation (DCR) has already been recommended in the 2016 Guideline. The recent literature has provided specific recommendations applying to patients who have no pre-existing cardiopulmonary diseases [14] and are in haemorrhagic shock in the preoperative, the intra-operative [14], or the early postoperative phase (3–6 h [16]). Using restricted fluid resuscitation and a target mean arterial pressure (MAP) of approximately 60 mmHg, Gu et al. were able to significantly reduce mortality (6.3% vs. 16.3% in a group of patients who received routine fluid resuscitation, *p* = 0.045) and the incidences of acute respiratory distress syndrome (ARDS) (12.5% vs. 27.5%, *p* = 0.018) and multiple organ dysfunction syndrome (MODS) (8.8% vs. 22.5%, *p* = 0.017) [14]. Lu et al. too significantly reduced mortality (2.4% vs. 18.3%, *p* = 0.041) and the incidences of coagulation disorders (2.4% vs. 17.1%, *p* = 0.039), ARDS (12.2% vs. 30.5%, *p* = 0.006) and MODS (12.2% vs. 29.3%, *p* = 0.027) [16]. Patients with a history of hypertension may need a higher MAP, but there is no definitive evidence. The optimum blood pressure level in the further course is also unclear. The use of permissive hypotension does not mean that shock-related acidosis should be tolerated [136]. Possible target levels are base excess (BE) > −6 mEq/L, lactate < 4 mmol/L, and a pCO2 gap < 6 mmHg. Volume therapy is guided by the time course of these parameters [137]. Occult hypoperfusion (associated with elevated plasma soluble thrombomodulin and syndecan-1, indicating shock-induced endothelial dysfunction) can occur in the setting of normal vital signs. On arrival at the emergency department, more patients present with occult hypoperfusion than with shock [138]. In the presence of regular cardiac output, persistent microcirculatory hypoperfusion after initial resuscitation was found to be associated with an increased risk of MODS [57]. Lactate (odds ratio [OR] 1.17; 95% confidence interval [CI]: 1.12–1.23; *p* < 0.00001) and base deficit (BD) (OR 1.04; 95% CI: 1.01–1.07; *p* < 0.005) predicted overall mortality. Initial lactate was reported to be a better predictor of mortality than BD for patients with severe blunt trauma. This applied to patients with or without shock and to patients with or without severe traumatic brain injury (TBI) [55].

Zhou et al. compared normothermia and moderate hypothermia in patients with haemorrhagic shock during surgery (35.9 ± 0.6 °C vs. 34.9 ± 0.6 °C) and in the intensive care unit (36.9 ± 0.4 °C vs. 35.8 ± 0.6 °C) [20]. The maintenance of normothermia, which can, for example, be achieved by increasing room temperature and by using only intravenous fluid warmed to 37 °C, was found to have beneficial effects, to shorten length of hospital stay (21.6 ± 4.1 days vs. 27.7 ± 6.1 days), and to reduce complication rates (6.6% vs. 26.6%) and mortality (2.2% vs. 13.3%). Maekawa et al. compared hypothermia (32–34 °C) and normothermia (35.3–37 °C) in patients with TBI [19]. There were no differences in the likelihood of poor neurological outcome after six months (relative risk [RR] 1.24, 95% confidence interval [CI] 0.62–2.48, *p* = 0.597) and mortality (relative risk, 1.82, 95% CI 0.82–4.03, *p* = 0.180). Excluded were two studies addressing therapeutic hypothermia for organ protection [17, 18] and another study investigating the effects of hypothermia on thromboelastography (TEG) [37].

Every second patient with multiple injuries is hypocalcaemic on arrival at the emergency department [139]. Citrate, which is added to stored blood as an anticoagulant, causes a decrease in ionised calcium (Ca^++^) levels after transfusions. This applies in particular to fresh frozen plasma (FFP). The prehospital administration of plasma was reported to lead to a considerable increase in the proportion of patients with hypocalcaemia (≤ 1 mmol/L) on admission and to be significantly associated with decreased survival (adjusted hazard ratio [HR]: 1.07; 95% CI: 1.02–1.13, *p* = 0.01) and massive transfusion (adjusted relative risk = 2.70; 95% CI: 1.13–6.46, *p* = 0.03) [31].

An analysis of fourteen studies on the effectiveness of a massive transfusion protocol for trauma patients demonstrated a significant reduction in overall mortality (odds ratio: 0.71, 95% CI: 0.56–0.90) [140].

The early transfusion of red blood cells (RBCs) is a life-saving intervention in patients with massive haemorrhage and haemorrhagic shock. The target of PRBC transfusion is a haemoglobin level between 7 and 9 g/dL (4.3–5.6 mmol/L) as recommended by the German Medical Association (1 C + recommendation) [141].

In patients with massive blood loss, it is appropriate in the acute phase to administer not only PRBCs but also therapeutic plasma, clotting products, and platelets [141]. Therapeutic plasma is a collective term that is used by the German Medical Association for plasma that has been stored in quarantine and has not undergone pathogen reduction treatment (Q-plasma, quarantine plasma, previously: fresh-frozen plasma), plasma that has been treated with methylene blue and red light or with amotosalen and UVA light for the purpose of pathogen reduction (PR plasma), lyophilised [freeze-dried] human plasma (LHP), which was made using a pool of ABO-identical leukocyte-depleted plasma donations, and plasma that was treated with solvent and detergent for virus inactivation (SD plasma). In the amended guidelines on the use of blood components and plasma derivatives, the German Medical Association specified for the first time that the use of therapeutic plasma is indicated when plasma volume must be replaced in patients with massive bleeding. A minimum dose of 30 mL/kg (30–50 mL/min) was recommended [141]. A post hoc analysis of the PAMPer [142] and COMBAT [143] trials suggested that prehospital plasma was associated with a survival benefit (adjusted HR 0.65; 95% CI 0.47–0.90; *p* = 0.01), especially when transport times were longer than twenty minutes (HR 0.78; 95% CI 0.40–1.51; Pp0.46) [32]. Since in this analysis patients received a mean of two units of plasma, it appears to be unlikely that this was a coagulation-related effect (rather than a volume-related effect) [144]. The same data showed that prehospital plasma was associated with a 32% lower hazard for 28-day mortality in patients with blunt injuries compared to patients with penetrating injuries (HR 0.68; 95% CI 0.47–0.96; *p* = 0.03) [33]. A secondary analysis of the PROPPR [75] trial showed that group A plasma was non-inferior to group AB plasma [61]. Incompatible transfusion of type A plasma did not lead to significant increases in the rates of ARDS (6% vs. 8%; *p* = 0.589), thromboembolic events (9% vs. 7%; *p* = 0.464), sepsis (6% vs. 8%; *p* = 0.589), acute renal failure (8%vs. 8%, *p* = 0.860), or mortality at 6 h (17% vs. 15%, *p* = 0.775), at 24 h (25% vs. 23%, *p* = 0.544) and at 28 days (38% vs. 35%, *p* = 0.486) when compared to compatible plasma transfusions [66]. Another study demonstrated that the utilisation of Group A plasma for emergency blood resuscitation did not result in higher mortality (17% vs. 26%, *p* = 0.15) but higher rates of ARDS (2% vs. 8%, *p* = 0.06) and sepsis (0% vs. 5%, *p* = 0.024) when compared to Group AB plasma transfusion [68]. Never-frozen liquid plasma can be an alternative to FFP. The time to transfusion of the first unit of never-frozen liquid plasma to trauma patients was reported to be significantly shorter compared with the transfusion of the first FFP unit (54 [28–79] minutes vs. 98 [59–133] minutes; *p* < 0.001), and there were no differences in mortality, complications and hospital length of stay [60].

The German Medical Association recommends a 1:1 to 1:2 ratio of plasma to PRBC units in the early management of patients with massive haemorrhage (1 C recommendation) [141]. In Germany, there are two types of platelet concentrates, i.e. pooled from 4 to 6 donors or prepared by apheresis from a single donor [141]. Accordingly, one German platelet concentrate unit is equal to approximately 4–6 US units of platelets from a single donor. For this reason, one platelet concentrate unit should be transfused after the first six PRBC and FFP units and then after every fourth transfusion of RBCs and FFP with a view to achieving a 1:1:1 ratio of RBCs to FFP to platelets which is recommended in US studies (1B recommendation of the German Medical Association) [141]. This recommendation is supported by a meta-analysis that was conducted by Rijnhout et al. who found that the US 1:1 ratio of platelets to RBCs significantly reduced mortality at 1 h to 6 h (OR 0.18; 95% CI 0.07–0.49; *p* = 0.0007), at 24 h (OR 0.2; 95% CI 0.08–0.21; *P* < 0.00001) and at 28 days (OR 0.68; 95% CI 0.50–0.91; *p* = 0.01) as well as ICU length of stay [145]. Bui et al. found that achieving an FFP: PRBC ratio ≥ 1:1.5 after the initial 24 h of resuscitation significantly improved survival in massively transfused patients (≥ 10 PRBC units in ≤ 24 h) compared to patients who achieved a ratio < 1:1.5 (14.3% vs. 31.5%, *p* = 0.042) [38]. In an analysis of 1,002,595 patients, 4427 patients were transfused ≥ 10 PRBCs and ≥ 1 FFP within 24 h. This analysis, which was conducted by Nederpelt et al., showed that mortality was 28% at a 1:1 FFP: PRBC ratio and the odds of mortality independently and incrementally increased to 1.23 (95% CI 1.02 to 1.48) for a 1:2 ratio, 2.11 (95% CI 1.42 to 3.13) for 1:4, and as high as 4.11 (95% CI 2.31 to 7.31) for 1:5 (all *p* < 0.05) [64]. An analysis of data from the French trauma registry, which included 897 patients who received ≥ 4 PRBC units during the first 6 h or died before receiving 4 PRBC units, revealed that a high transfusion ratio was associated with a significant reduction in 30-day mortality (HR, 0.74; 95% CI, 0.58–0.94; *p* = 0.01) [65]. Stanworth et al. conducted a study at 22 hospitals in the United Kingdom over a period of 20 months. They reported that an FFP: PRBC ratio < 1:2 correlated with an elevated mortality rate. Patients who did not receive a transfusion of FFP with PRBCs at a ratio < 1:2 had a 3.6 times higher mortality after 3 h, and a 2.3 times higher mortality at 24 h [41]. Kutcher et al. analysed laboratory parameters and demonstrated that transfusions with ratios of RBC: FFP approaching 1:1 led to more effective repletion of specific trauma-related coagulation factor deficits [39]. On the basis of data from the PROPPR trial [75], Cardenas et al. found that patients who received platelets had significantly decreased 24-hour mortality (5.8% vs. 16.9%; *P* < 0.05) and 30-day mortality (9.5% vs. 20.2%; *P* < 0.05) and fewer died as a result of exsanguination (1.5% vs. 12.9%; *P* < 0.01). It is interesting to note that the patients included in the PROPPR trial had a median platelet count of 243 × 10^9^/L prior to transfusion [59]. Apart from platelet count, platelet function plays an important role in achieving adequate haemostasis [141].

Compared to conventional coagulation tests [46, 48], viscoelastic assays can be completed in a shorter time [51] and were reported to be superior in improving survival [12], predicting the need for massive transfusion [51, 58, 146], detecting TIC [46, 48, 146], reducing ICU stay [12, 22] and mechanical ventilation times [12, 22], and reducing blood product transfusion [48]. Viscoelastic assays were also found to be useful in patients with isolated TBI [49]. Barret et al. used a modified thromboelastometry assay to predict the risk for massive transfusion and mortality and found a theoretical survival advantage based on extrapolation of data [51]. In their Reversal of Trauma-Induced Coagulopathy (RETIC) study, Innerhofer et al. demonstrated an indirect advantage since rotational thromboelastometry (ROTEM) was required to identify a survival advantage with coagulation factor concentrates [76]. Hagamo et al. reported that ROTEM parameters (EXTEM CA5 ≤ 40 mm, FIBTEM CA5 ≤ 9 mm) effectively detected TIC and predicted massive transfusion after five minutes [146]. Moore et al. used modified thromboelastography, i.e. tissue plasminogen activator thromboelastography (tPA-TEG) to predict massive transfusion [58]. Peng et al. came to the conclusion that viscoelastic assays were better than laboratory conventional coagulation tests (CCTs), TEG and ROTEM for monitoring coagulation profiles and predicting transfusion requirements. In addition, TEG and ROTEM detected increases in clot strength following the use of fibrinogen. ROTEM also detected changes in coagulation time and clot lysis [46]. Rizoli et al. reported that ROTEM maximum clot firmness (MCF) and TEG maximum amplitude (MA) clinically showed reasonable predictive accuracy for mortality and plasma transfusion and strong accuracy for any or massive blood transfusion [47]. Spagnolello et al. developed a ROTEM Sigma based algorithm that allowed coagulation results to be obtained faster than laboratory CCTs and thus led to earlier clinical intervention [48]. Gratz et al. introduced a ROTEM-guided algorithm in four European medical centres. This algorithm allowed them to obtain results significantly earlier than conventional coagulation tests (median (IQR [range]) 33 (20–40 [14–250]) min vs. 71 (51–101 [32–290]) min; *p* = 0.037) [147]. Viscoelastic assays were reported to have high negative predictive values (NPVs) for TIC, massive transfusion and mortality in trauma [146, 148–150]. Algorithms based on viscoelastic assays thus mainly provide data on what does *not* need to be replaced. VET should complement, not replace, clinical judgment of the patient’s situation [151]. The treatment of a bleeding patient depends on the patient’s individual clinical situation, not on reference values. Results obtained with different viscoelastic assays (ROTEM™ vs. TEG™ vs.…) [47] are not interchangeable. Different generations of the same system (i.e. ROTEM™delta vs. ROTEM™sigma or TEG™ 5000 vs. TEG™ 6 s) and even different cartridges (single use vs. liquid reagent vs. controlled cartridge) can lead to different results [148, 152]. Since viscoelastic assays cannot identify all causes of bleeding, a blood count is useful.

Platelet dysfunction is a central component of TIC. Shock and tissue injury have different effects on platelet aggregation [153]. In addition, a wide variety of medications can adversely affect platelets. Connelly et al. found that Multiplate™, TEG™ PM and VerifyNow™ are useful point-of-care (POC) assays that can detect antiplatelet medications or trauma-induced platelet dysfunction [53].

The European Guideline [154] recommends a goal-directed approach for the management of massive bleeding on the basis of what is known as the Copenhagen Concept [155], which is a hybrid approach [148] consisting of the initial ratio-driven administration of blood products followed as rapidly as possible by goal-directed replacement therapy based on viscoelastic assays and platelet function tests.

A secondary analysis of data from 20,211 CRASH-2 patients [156] showed that the survival benefit of tranexamic acid (TXA) was only evident in patients in whom treatment was initiated within three hours of their injury (HR ≤ 3 h = 0.78, 95% CI 0.68–0.90; HR > 3 h = 1.02, 95% CI 0.76–1.36) [36]. Another secondary analysis [156] demonstrated that early administration of TXA reduced the risk of death due to bleeding. Treatment initiated within an hour of injury was associated with an RR of 0.68 (95% CI 0.57 to 0.82), treatment initiated between 1 and 3 h after injury with an RR of 0.79 (95% CI 0.64 to 0.97), and treatment beyond 3 h with an RR of 1.44 (95% CI 1.12 to 1.84) [35]. In a study that was conducted by Guyette et al., patients received 1 g of TXA (or placebo) infused for 10 min in 100 mL of 0.9% saline. Treatment with TXA reduced 30-day mortality when TXA was administered within 1 h of injury (4.6% vs. 7.6%; difference, −3.0%; 95% CI −5.7% to −0.3%; *P* < 0.002) or when patients were in severe shock (systolic blood pressure ≤ 70 mmHg) (18.5% vs. 35.5%; difference, −17%; 95% CI, −25.8% to −8.1%; *P* < 0.003) [25]. In patients with hyperfibrinolysis (Ly30 > 3%) on thromboelastography, TXA significantly lowered 6-hour mortality rate (34% vs. 13%, *p* = 0.04). However, there was no difference between patients who received TXA and patients who were not treated with TXA in 12-hour (*p* = 0.24), 24-hour (*p* = 0.25) and 30-day mortality (*p* = 0.82) [34]. In patients with TBI, TXA appeared to be effective only in those with mild-to-moderate head injury (GCS ≥9) (risk ratio, 0.78, 95% CI 0.64–0.95) [156]. The different phenotypes of TIC were described as early as 2014. Hyperfibrinolysis is associated with the highest mortality rate (> 40%) but occurs in only approximately 20% of patients. Less than 20% of patients have physiologic fibrinolysis, which has a lower mortality rate. Approximately two thirds of trauma patients have decreased clot degradation (hypofibrinolysis and shutdown), which has a mortality of about 20% [129, 131]. TXA was found to be a significant predictor of mortality for patients without hyperfibrinolysis. This applies to both physiologic fibrinolysis (after adjusting for ISS, *p* = 0.018) [42] and shutdown (adj. RR 1.35; 95% CI 1.10–1.64; *p* = 0.004) [132]. TXA can be an independent risk factor for thromboembolic events in patients with multiple trauma [27, 157]. The positive immunological [27] and neurological [158, 159] effects that were reported in the literature could not be demonstrated, but active Duplex screening revealed a dose-dependent increase in thromboembolic events [27]. TXA must therefore be administered on a selective individual basis [158, 160]. As a general rule, the likelihood of hyperfibrinolysis increases with the severity of injury and the severity of shock [25, 160, 161].

Multiply injured patients who required transfusion and received fibrinogen had significantly better results than patients who received FFP in terms of mortality (*p* = 0.029), sepsis (*p* = 0.001), the need for admission to an ICU (*p* = 0.020), the need for transfusion (*p* = 0.044) and intravenous fluid in the initial 24 h of hospitalisation (*p* = 0.022), and hospital length of stay (*p* = 0.045). In addition, the number of patients with multiple organ failure was about one fourth the number in the other group of patients (*p* = 0.106) [21]. Nascimento et al. showed that early infusion of 6 g of fibrinogen concentrate was feasible and increased plasma fibrinogen concentration by approximately 1 g/L [30]. In their RETIC study, Innerhofer et al. compared initial ROTEM™-guided administration of fibrinogen concentrate (50 mg/kg, if FIBTEM A10 < 9 mm) and prothrombin complex concentrates (PCCs, 20 IU/kg, if EXTEM CT > 90 s) with FFP (15 mL/kg). If the effect of treatment was insufficient, the fibrinogen group received FFP or, vice versa, the FFP group received fibrinogen. All patients were treated with TXA. Following a preplanned interim analysis, the study was terminated early because the number of patients who required rescue therapy (52% vs. 4%; OR 25.34; 95% CI 5.47 to 240.03; *P* < 0.0001) and massive transfusion (30% vs. 12%; OR 3.04, 95% CI 0.95 to 10.87; *p* = 0.042) was significantly higher in the FFP group [22]. Time to initiation of therapy was significantly longer in the FFP group (median, 50.5 min [IQR 39.5 to 70.0] vs. 10 min [10 to 16]; difference, −40 [95% CI −46 to −33], *P* < 0.0001). Time to haemostasis too was significantly longer in the FFP group (128.0 min [48.3 to 186.3] vs. 22.5 min [13.5 to 40.0]; difference, −97 min [−126 to −60], *P* < 0.0001). A mean dose of 3.8 g (±1.2 g) of fibrinogen was reported to increase FIBTEM CA5 by 5.2 mm (IQR: 4.1–6.3 mm) [162]. Other authors too recommended a dose of 4 g of fibrinogen concentrate and defined a FIBTEM CA5 < 10 mm and an FF TEG < 20 mm as indications for fibrinogen administration [148]. There is no evidence-based data on the fibrinogen level that should be reached in trauma patients. In trauma patients, the effectiveness of fibrinogen administration in limiting blood loss appears to depend considerably on early administration and the achievement of a level of > 2 g/L [163].

For a long time, decreased thrombin generation has not been identified as a problem in the early phase of TIC [164, 165]. This, however, appears to be the case in trauma patients with shock [166]. Endogenous thrombin potential was significantly higher for three days in patients who received PCCs than in patients who were treated with fibrinogen concentrate. A theoretical risk for developing thromboembolism is therefore present [88]. As described above, Innerhofer et al. compared fibrinogen and PCCs versus FFP [22]. Patients received PCCs (20 IU/kg of body weight [BW]) if EXTEM CT was > 90 s. Zeeshan et al. reported that patients who received a combination of PCCs and FFP had a lower mortality (17.5% vs. 27.7%, *p* = 0.01) and lower rates of ARDS (1.3% vs. 4.7%, *p* = 0.04) and acute kidney injury (2.1% vs. 7.3%, *p* = 0.01) than patients who were treated with FFP alone [67]. In a meta-analysis, van den Brink et al. too found that, unlike PCCs alone, a combination of PPC and FFP was associated with significantly reduced mortality in trauma patients (OR 0.64; 95% CI 0.46–0.88; *p* = 0.007) [167].

All patients with multiple injuries develop a hyp*o*fibrinolytic state within 24 h of injury and are thus at risk of thromboembolism. It is often difficult to determine the optimal time to initiate thromboprophylaxis. Omission of thromboprophylaxis within the first 24 h was reported to be associated with an increased risk of mortality [168]. In a study on patients with isolated TBI, Byrne et al. found that early initiation of pharmacological prophylaxis (< 72 h after trauma) was associated with lower rates of pulmonary embolism (OR, 0.48; 95% CI, 0.25–0.91) and deep vein thrombosis (OR, 0.51; 95% CI, 0.36–0.72) but not with an increased risk of surgical intervention or death when compared with late prophylaxis (> 72 h) [69]. Initiation of pharmacological thromboprophylaxis ≤ 48 h after trauma resulted in a lower rate of deep vein thrombosis (0% vs. 9%, *p* = 0.024) in patients who sustained isolated blunt intra-abdominal solid organ injuries and were managed non-operatively [45]. The same recommendation applies to blunt pelvic fractures. Pelvic angioembolisation was found to be independently associated with venous thromboembolism (VTE) (OR, 1.296; *p* = 0.044) [44]. If pharmacologic venous thromboembolism prophylaxis is contraindicated, mechanical prophylaxis, e.g. in the form of intermittent pneumatic compression (IPC) devices, should be considered.

The following table summarises the aforementioned escalating pharmacological treatment options for coagulopathic bleeding (*without* the use of viscoelastic assays).

**Table:** 

Escalating treatment options	Treatment details
1. Stabilisation of concomitant factors (prophylaxis *and* therapy)	Core temperature ≥34 °C (normothermia, if possible)
	pH ≥7.2
	Ionised Ca^++^levels of > 0.9 mmol/L (normocalcaemia, if possible)
2. Inhibition of potential (hyper)fibrinolysis *as early as possible* (always *BEFORE* the administration of fibrinogen)	Loading dose of **tranexamic acid** of **1 g** (15–30 mg/kg BW) over 10 min; if necessary,, followed by 1 g over 8 h
3. Replacement of oxygen carriers	**PRBCs** in patients with massive bleeding, target haemoglobin level of approximately 7–9 g/dL (4.3–5.5 mmol/L)
4. Replacement of coagulation factors (in patients with ongoing severe haemorrhage) If patients in need of massive transfusion or with life-threatening shock from bleeding are treated with therapeutic plasma, they can benefit from a high ratio of 4(–6):4(–6):1 for units of therapeutic plasma to PRBCs to platelet concentrates or from a combination of therapeutic plasma and coagulation factor concentrates and platelet concentrates.	
	**Fibrinogen** 3–6 g (30–60 mg/kg BW; target: 2–2.5 g/L)
	*and*, if required, **PCCs** at an initial dose of 25 IU/kg BW
	**FXIII** 20 IU/kg BW, if required Target: FXIII activity > 60%
*and* (if thrombocytopathy is suspected) increased platelet adhesion to the endothelium + release of von Willebrand factor and FVIII from liver sinusoids (→ vasopressin receptor type 2 agonist)	**Desmopressin (DDAVP)** 0.3 µg/kg BW over 30 minutes (rule of thumb: „one ampoule per 10 kg BW over 30 minutes”)
5. Replacement of plasma volume	**FFP** ≥ 30 mL/kg BW
6. Replacement of platelets for primary haemostasis	**Platelet concentrates**: target in patients requiring transfusion for bleeding and/or TBI > 100,000/µL
7. If necessary, thrombin burst with platelet and coagulation activation (pay attention to concomitant factors! Off-label use!)	If necessary, **rFVIIa** on a case-by-case basis and if all other treatment options fail Initial dose of 90 µg/kg BW

Initiation of thromboprophylaxis is mandatory within 24 h of bleeding cessation

Ultrasound-guided placement of central venous catheters is recommended in many clinical situations. In a study on 70 patients which, however, included only 12 patients with multiple trauma, Kunhahamed et al. found that the ultrasound-guided technique allowed catheters to be placed more rapidly and more successfully than the traditional landmark technique [43].

### Limitations of the guideline

Patient values and preferences were sought but not received. The effect of this on the guideline is unclear, and there is a lack of research evidence on the effect of patient participation on treatment decisions or outcomes in the emergency setting. This guideline applies to trauma care in Germany, i.e. a high-income setting, well-developed medical infrastructure, and emergency medical service teams comprising a physician and at least one paramedic.

## Electronic supplementary material

Below is the link to the electronic supplementary material.

Supplementary Material 1

## Data Availability

Data is provided within the manuscript or supplementary information files.

## Abbreviations and acronyms

ABC: Assessment of blood consumption
adj.: Adjusted
aPTT: Activated partial thromboplastin time
ARDS: Acute respiratory distress syndrome
ATC: Acute traumatic coagulopathy
BD: Base excess
BE: Base excess
BW: Body weight
CCT: Conventional coagulation test
CFC: Coagulation factor concentrate
CG: Control group
d: Day
DCR: Damage control resuscitation
ERP: Emergency-release plasma
FF: Functional fibrinogen
FFP: Fresh frozen plasma
GCS: Glasgow Coma Scale
h: Hour
HR: Hazard ratio
HSS: Hypertonic saline solution
ICH: Intracranial haemorrhage
IG: Intervention group
INR: International normalised ratio
IPC: Intermittent pneumatic compression
IQR: Interquartile range
ISS: Injury Severity Score
ITT: Intention-to-treat
LHP: Lyophilised human plasma
LRS: Lactated Ringer´s solution
MA: Maximum amplitude or maximum clot strength
MAP: Mean arterial pressure
MCF: Maximum clot firmness
MD: Mean difference
MODS: Multiple organ dysfunction syndrome
MTP: Massive transfusion protocol
NISS: New Injury Severity Score
n.r.: Not reported
n.s.: Not significant
OR: Odds ratio
PBO: Placebo
P/F ratio: Ratio of arterial oxygen partial pressure (PaO2) to fractional inspired oxygen (FiO2)
PRBC: Packed red blood cell
PT: Prothrombin time
PTH: Parathyroid hormone
RBC: Red blood cell
RCT: Randomised controlled trial
ROTEM: Rotational thromboelastometry
RR: Risk ratio
R-TEG: Rapid thromboelastography
SBP: Systolic blood pressure
SD: Standard deviation
SI: Shock index
TASH: Trauma-associated severe haemorrhage
TBI: Traumatic brain injury
TEG: Thromboelastography
TF: Tissue factor
TIC: Trauma-induced coagulopathy
tPA: Tissue plasminogen activator
TXA: Tranexamic acid
unadj.: Unadjusted
y: Year
VHA: Viscoelastic haemostatic assay
VTE: Venous thromboembolism
