# Supplementary material for: Inhospital coagulation management and fluid replacement therapy in patients with multiple and/or severe injuries – a systematic review and clinical practice guideline update
Source: Eur J Trauma Emerg Surg. 2025 Jun 27;51(1):240. doi: 10.1007/s00068-025-02919-2 (PMC12204895; doi:10.1007/s00068-025-02919-2)
Supplement: Supplementary file 1 — Supplementary Material 1 [file 68_2025_2919_MOESM1_ESM.pdf]

Online Resource

Inhospital coagulation management and fluid replacement therapy in patients with multiple and/or severe injuries – A systematic review and clinical practice guideline update

Table S1. PICO questions[1]

The target population of the guideline consists of adults (≥14 years) with suspected polytrauma or trauma-related severe injury (ISS ≥16 and/or GCS <9) in the prehospital phase of care.

	Population	Intervention(s)	Control(s)	Outcome(s)
	**Coagulation assays and therapy**			
1	target population	early coagulation assays	delayed coagulation assays	clinically relevant outcomes (coagulopathy, others)
2	target population	early coagulation therapy	delayed coagulation therapy	clinically relevant outcomes (coagulopathy, others)
3	target population	standard laboratory: ABG, Quick (prothrombin time), aPTT, fibrinogen and platelet count	not specified	clinically relevant outcomes (coagulopathy, other)
4	target population	early measurement of ABG, Quick (prothrombin time), aPTT, fibrinogen and platelet count	delayed measurement	clinically relevant outcomes (coagulopathy, other)
5	target population	multiple measurement of ABG, Quick (prothrombin time), aPTT, fibrinogen and platelet count	single measurement	clinically relevant outcomes (coagulopathy, other)
6	target population	blood grouping	no blood grouping	clinically relevant outcomes

[1] Table translated from German using DeepL Translate (https://www.deepl.com/translator) with manual modifications

	Population	Intervention(s)	Control(s)	Outcome(s)
				(coagulopathy, other)
7	target population	viscoelastic testing	not specified (incl. other viscoelastic test procedure)	coagulopathy and other clinically relevant outcomes (coagulopathy, other)
8	target population	early viscoelastic assays	viscoelastic assays at any other time	coagulopathy and other clinically relevant outcomes
	**Volume therapy**			
9	target population, actively bleeding	volume therapy with permissive hypotension (defined blood pressure target)	volume therapy with blood pressure target normotension (other defined blood pressure target)	clinically relevant outcomes
10	target population, with haemorrhagic shock and<br>- traumatic brain injury (GCS <9)<br>- Spinal trauma with neurological symptoms	volume therapy with blood pressure target MAP 85-90 mmHg	volume therapy with a different blood pressure target	clinically relevant outcomes
11	target population, without central nervous injury and without CHD	volume therapy with blood pressure target MAP ~50 mmHg	volume therapy with a different blood pressure target	clinically relevant outcomes
	**Infusions**			
12[§]	target population	colloids	not specified	coagulopathy, mortality, other clinically relevant endpoints
	**Base excess/lactate**			
13	target population, with shock	measurement of base excess / lactate during shock treatment	not specified	coagulopathy, other outcome, therapy decision, sufficient perfusion despite hypotension
14	target population, with shock	repeated measurement of base excess / lactate during shock treatment	single measurement of base excess / lactate	coagulopathy, other outcome, therapy decision, sufficient perfusion despite hypotension

	Population	Intervention(s)	Control(s)	Outcome(s)
	**Temperature management**			
15	target population	Measures to avoid cooling (≤34°C), aim for normothermia, (use of warmed infusion solutions (40-42°C), blankets, removal of wet clothing, warming mat, radiant warmer, hot air blower, RT 28-29°C).	none / other measure, no normothermia	mortality and other clinically relevant outcomes, platelet function, coagulation factor activity
16§	target population	heat exchange catheter	not specified	mortality and other clinically relevant outcomes
	**Acidaemia, hypocalcaemia**			
17	target population	procedures to avoid acidaemia (≤7.2, buffering)	no procedures to avoid acidaemia (procedures that exacerbate acidosis, such as hypoventilation, NaCl infusion)	coagulopathy, mortality, other clinically relevant outcomes
18	target population	acidaemia treatment (≤7.2) by appropriate therapy of shock	no acidaemia treatment	acidaemia, mortality, other clinically relevant outcomes
19	target population	procedures to avoid hypocalcaemia <0.9 mmol/l (e.g. less FFP or slower transfusion)	no procedures to avoid of hypocalcaemia	hypocalcaemia, mortality, other clinically relevant outcomes
20	target population	procedures aiming to achieve normocalcaemia (substitution with Ca2+)	no procedures aiming to achieve normocalcaemia	hypocalcaemia, mortality and other clinically relevant outcomes
	**Blood products, transfusion**			
21	target population	treatment according to massive transfusion or coagulation therapy protocol	treatment not according to massive transfusion or coagulation therapy protocol	mortality, coagulopathy, other clinically relevant outcomes
22	target population, actively bleeding	decision on indication for transfusion individually	no individual decision / decision based on other criteria (incl. scores)	mortality, coagulopathy, other clinically relevant outcomes

	Population	Intervention(s)	Control(s)	Outcome(s)
		according to clinical criteria, degree of injury, extent of blood loss, circulatory situation and oxygenation.		
23[§]	target population	plasma (FFP, lyophilised plasma, SD plasma)	not specified	mortality, coagulopathy, other clinically relevant outcomes
24	target population, (expected) massive transfusion	early use of FFP	different timing for use of FFP	mortality, coagulopathy, other clinically relevant outcomes
25	target population, with (massive) transfusion	administration of FFP:EC:TC in a defined ratio (e.g. 4:4:1)	different ratio of FFP:EC:TC	mortality, other clinically relevant outcomes
26	target population, with massive transfusion	massive transfusion with factor concentrates controlled by appropriate test procedure	massive transfusion with factor concentrates without test procedure (always same dose)	mortality, other clinically relevant outcomes
27	target population, receiving massive transfusion with factor concentrates	test procedure to control interventions with factor concentrates	other test procedure	mortality, other clinically relevant outcomes
28	target population	substitution with fibrinogen	not specified	mortality, other clinically relevant outcomes
29	target population	substitution with fibrinogen at fibrinogen levels of about <1.5 g/l (150 mg/dl)	substitution with fibrinogen according to other criteria	mortality, other clinically relevant outcomes
	**Tranexamic acid (TXA)**			
30	target population	TXA	no TXA; other intervention	mortality, other clinically relevant outcomes
31	target population	one TXA dose regimen (amount, infusion duration, time(s))	other TXA dose regimen (amount, infusion duration, timing(s))	mortality, other clinically relevant outcomes

	Population	Intervention(s)	Control(s)	Outcome(s)
32	target population	no TXA >3h after trauma	TXA >3h after trauma	mortality, other clinically relevant outcomes
33	target population	TXA without time limit	no TXA	mortality, other clinically relevant outcomes
	**Thrombosis prophylaxis**			
30	target population	early thrombosis prophylaxis	later thrombosis prophylaxis	mortality, other clinically relevant outcomes, VTE

§ new PICO question

Table S2. Literature search details

Search strategy 2021, MEDLINE (via Ovid)	Date: 07.05.2021	2.545 Hits

1. exp Multiple Trauma/
2. (polytrauma* or trauma patient?).ti,ab,kf. or (severe adj2 shock).ti,ab,kf.
3. ((multiple or major or severe* or serious*) adj3 (trauma* or injur*)).ti,ab,kf.
4. ((blunt or penetrating) adj5 (trauma* or injur*)).ti,ab,kf.
5. (*Critical Care/ or *Emergencies/ or (life threatening or critical care or emergen*).ti,ab,kf.) and (trauma* or injur*).ti,ab,kf.
6. 1 or 2 or 3 or 4 or 5
7. exp animals/ not humans.sh.
8. (comment or editorial or letter).pt. or case report*.mp.
9. Shock, Hemorrhagic/ or exp Exsanguination/ or exp Hypovolemia/ or exp Shock, Traumatic/
10. (((uncontrolled or uncontrollable or acute or active or massive or life threatening or severe) adj2 (bleeding or h?emorrhage)) or ((hypovol?emic or h?emorrhagic or traumatic) adj (shock or trauma*)) or "damage control resuscitation").ti,ab,kf.
11. exp Hemostasis/ or exp Blood Coagulation/ or exp disseminated intravascular coagulation/ or exp Hemorrhage/
12. (h?emosta* or coagula* or clotting or coagulopath* or h?emorrhag* or bleed*).ti,ab,kf.
13. 9 or 10 or 11 or 12
14. exp Fluid Therapy/ or exp rehydration solutions/ or exp hypotonic solutions/ or exp isotonic solutions/ or exp crystalloid solutions/ or exp Colloids/ or exp ringer's lactate/ or exp saline solution/ or exp ringer's solution/ or exp Hydroxyethyl Starch Derivatives/ or Hypertonic Solutions/ or exp Saline Solution, Hypertonic/ or exp Plasma/ or exp Erythrocytes/ or exp Blood Transfusion/ or prothrombin complex concentrates.mp. or exp Deamino Arginine Vasopressin/ or exp Factor VIII/ or exp Platelet Transfusion/ or exp Fibrinogen/ or exp Tranexamic Acid/ or exp Blood Coagulation Tests/ or exp Thrombelastography/
15. (((fluid or volume) adj2 (therap* or replacement? or expansion or management or substitute or substitution or administration or resuscitation)) or "hypotensive resuscitation" or "permissive hypotension").ti,ab,kf. or (((isotonic or normal) adj (saline or sodium chloride or NACL)) or ((hypotonic or isotonic or buffered or balanced) adj (infusion? or solution? or electrolyte?)) or crystalloid? or colloid? or ringer* or albumin or hydroxyethyl starch* or hetastarch or dextran).ti,ab,kf. or ((hypertonic or hyperosmotic or hyperoncotic or hyperosmolar) adj2 (infusion? or solution? or electrolyte? or saline or resuscitation)).ti,ab,kf. or (((lactic acid or lactate) and shock) or (base adj (excess or deficit)) or acidosis or acid-base-status).ti,ab,kf. or (normotherm* or hypotherm* or ((preservation or retention or conservation or control or management) adj1 (temperature or thermic* or heat or warm*))).ti,ab,kf. or acid?emia.ti,ab,kf. or (hypocalc?emia or (reduced adj2 calcium)).ti,ab,kf. or (plasma or PRBC or PRBCs or transfusion or "factor concentrate?" or fibrinogen or hypofibrinogen?emia or factor xiia or factor viia or rfviia or thrombin).ti,ab,kf. or (tranexamic acid or txa).ti,ab,kf. or (thrombo* adj1 prophyla*).ti,ab,kf. or (blood gas analysis or quick or partial thromboplastin time or aptt or ((platelet or thrombocyte) adj count) or viscoelastic test? or thromb?elasto* or ROTEM or (rotation* and thromb?elastometry)).ti,ab,kf.
16. 14 or 15
17. 13 and 6 and 16
18. 17 not 7
19. 18 not 8
20. limit 19 to dt=20140101-20210322

Search strategy 2021, Additional question on intraosseous access, MEDLINE (via Ovid)
Date: 07.05.2021 83 Hits

1. exp Multiple Trauma/
2. (polytrauma* or trauma patient?).ti,ab,kf. or (severe adj2 shock).ti,ab,kf.
3. ((multiple or major or severe* or serious*) adj3 (trauma* or injur*)).ti,ab,kf.
4. ((blunt or penetrating) adj5 (trauma* or injur*)).ti,ab,kf.
5. (*Critical Care/ or *Emergencies/ or (life threatening or critical care or emergen*).ti,ab,kf.) and (trauma* or injur*).ti,ab,kf.
6. 1 or 2 or 3 or 4 or 5
7. exp animals/ not humans.sh.
8. (comment or editorial or letter).pt. or case report*.mp.
9. exp Infusions, Intraosseous/
10. ((intraosseous adj2 (infusion? or puncture? or access or device?))).ti,ab,kf.
11. 9 or 10
12. 6 and 11
13. 12 not 7
14. 13 not 8
15. limit 13 to dt=19460101-20210322

Search strategy 2021, Additional questions on access/catheterisation, MEDLINE (via Ovid)
Date: 07.05.2021 46 Hits

1. exp Multiple Trauma/
2. (polytrauma* or trauma patient?).ti,ab,kf. or (severe adj2 shock).ti,ab,kf.
3. ((multiple or major or severe* or serious*) adj3 (trauma* or injur*)).ti,ab,kf.
4. ((blunt or penetrating) adj5 (trauma* or injur*)).ti,ab,kf.
5. (*Critical Care/ or *Emergencies/ or (life threatening or critical care or emergen*).ti,ab,kf.) and (trauma* or injur*).ti,ab,kf.
6. 1 or 2 or 3 or 4 or 5
7. exp animals/ not humans.sh.
8. (comment or editorial or letter).pt. or case report*.mp.
9. Shock, Hemorrhagic/ or exp Exsanguination/ or exp Hypovolemia/ or exp Shock, Traumatic/
10. (((uncontrolled or uncontrollable or acute or active or massive or life threatening or severe) adj2 (bleeding or h?emorrhage)) or ((hypovol?emic or h?emorrhagic or traumatic) adj (shock or trauma*)) or "damage control resuscitation").ti,ab,kf.
11. exp Hemostasis/ or exp Blood Coagulation/ or exp disseminated intravascular coagulation/ or exp Hemorrhage/
12. (h?emosta* or coagula* or clotting or coagulopath* or h?emorrhag* or bleed*).ti,ab,kf.
13. 9 or 10 or 11 or 12
14. exp Catheterization, Central Venous/
15. (((heat exchang* or thermoregulatory or warming or hypothermia) adj4 catheter) or ((endovascular or intravascular) adj cooling)).ti,ab,kf. or (((central venous or arterial) adj catheter*) and (subclavian or femoral* or jugular*)).ti,ab,kf. or ((intravascular or vascular or intravenous or venous or iv or vein or arterial) adj (access* or cannulation)).ti,ab,kf. and (exp Ultrasonography/ or (ultrasonography* or ultrasound).ti,ab,kf.)

16 14 or 15

17. 13 and 6 and 16
18. 17 not 7

19. 18 not 8

20. limit 19 to dt=19460101-20210322

**Search strategy 2021, Embase (via Elsevier)**	**Date: 07.05.2021**	**667 Hits**

#1 'multiple trauma'/exp

#2 (polytrauma* OR "trauma patient?"):ti,ab,kw OR (severe NEXT/2 shock):ti,ab,kw

#3 ((multiple OR major OR severe* OR serious*) NEXT/3 (trauma* OR injur*)):ti,ab,kw

#4 ((blunt OR penetrating) NEXT/5 (trauma* OR injur*)):ti,ab,kw

#5 ('intensive care'/mj OR 'emergency'/mj OR ("life threatening" OR "critical care" OR emergen*):ti,ab,kw) AND (trauma* OR injur*):ti,ab,kw

#6 #1 OR #2 OR #3 OR #4 #5

#7 'animals'/exp NOT 'humans'/de

#8 (comment OR editorial OR letter):it OR "case report*":ti,ab,kw

#9 [1-1-2014]/sd NOT [23-3-2021]/sd

#10 [embase]/lim

#11 embase NOT (embase AND medline)

#12 'hemorrhagic shock'/exp OR 'hypovolemic shock'/exp OR 'traumatic shock'/exp OR 'exsanguination'/exp OR 'hypovolemia'/exp

#13 (((uncontrolled OR uncontrollable OR acute OR active OR massive OR "life threatening" OR severe) NEAR/2 (bleeding OR h$emorrhage)) OR ((hypovol$emic OR h$emorrhagic OR traumatic) NEXT/1 (shock OR trauma*)) OR "damage control resuscitation"):ti,ab,kw

#14 'hemostasis'/exp OR 'blood clotting'/exp OR 'disseminated intravascular clotting'/exp OR 'bleeding'/exp

#15 (h$emosta* OR coagula* OR clotting OR coagulopath*OR h$emorrhag* OR bleed*):ti,ab,kw

#16 #12 OR #13 OR #14 OR #15

#17 'fluid therapy'/exp OR 'rehydration'/exp OR 'hypotonic solution'/exp OR 'isotonic solution'/exp OR 'crystalloid'/exp OR 'colloid'/exp OR 'Ringer lactate solution'/exp OR 'sodium chloride'/exp OR 'Ringer solution'/exp OR 'hetastarch'/exp OR 'hypertonic solution'/exp OR 'sodium chloride'/exp OR 'blood clotting test'/exp OR 'thromboelastography'/exp OR 'plasma'/exp OR 'erythrocyte'/exp OR 'blood transfusion'/exp OR 'prothrombin complex'/exp OR 'argipressin[1 deamino]'/exp OR 'blood clotting factor 8'/exp OR 'thrombocyte transfusion'/exp OR 'fibrinogen'/exp OR 'tranexamic acid'/exp

#18 (((fluid OR volume) NEAR/2 (therap* OR replacement? OR expansion OR management OR substitute OR substitution OR administration OR resuscitation)) OR "hypotensive resuscitation" OR "permissive hypotension"):ti,ab,kw OR (((isotonic OR normal) NEAR/1 (saline OR “sodium chloride” OR NACL)) OR ((hypotonic OR isotonic OR buffered OR balanced) NEAR/1 (infusion? OR solution? OR electrolyte?)) OR crystalloid? OR colloid? OR ringer* OR albumin OR “hydroxyethyl starch*” OR hetastarch OR dextran):ti,ab,kw OR ((hypertonic OR hyperosmotic OR hyperoncotic OR hyperosmolar) NEAR/2 (infusion? OR solution? OR electrolyte? OR saline OR resuscitation)):ti,ab,kw OR (“blood gas analysis” OR quick OR “partial thromboplastin time” OR aptt OR ((platelet OR thrombocyte) NEXT/1 count) OR “viscoelastic test?” OR thromb$elasto* OR ROTEM OR (rotation* AND thromb$elastometry)):ti,ab,kw OR (((“lactic acid” OR lactate) AND shock) OR (base NEXT/1 (excess or deficit)) OR acidosis OR acid-base-status):ti,ab,kw OR (normotherm* OR hypotherm* OR ((preservation OR retention OR conservation OR control OR management) NEAR/1 (temperature OR thermic* OR heat OR warm*))):ti,ab,kw OR acid$emia:ti,ab,kw OR (hypocalc$emia OR (reduced NEAR/2 calcium)):ti,ab,kw OR (plasma OR PRBC OR PRBCs OR transfusion OR "factor concentrate?" OR fibrinogen OR hypofibrinogen$emia OR “factor xiia” OR “factor viia” OR rfviia OR thrombin):ti,ab,kw OR (“tranexamic acid” OR txa):ti,ab,kw OR (thrombo* NEAR/1 prophyla*):ti,ab,kw

#19 #17 OR #18
#20 #6 AND #16 AND #19
#21 #20 NOT #7
#22 #21 NOT #8
#23 #22 AND #9
#24 #23 AND #10
#25 #24 AND #11
#26 #25 AND ('article'/it OR 'article in press'/it OR 'erratum'/it)

Search strategy 2021, Additional question on intraosseous access, Embase (via Elsevier)
Date: 06.05.2021 **15 Hits**

#1 'multiple trauma'/exp
#2 (polytrauma* OR "trauma patient?"):ti,ab,kw OR (severe NEXT/2 shock):ti,ab,kw
#3 ((multiple OR major OR severe* OR serious*) NEXT/3 (trauma* OR injur*)):ti,ab,kw
#4 ((blunt OR penetrating) NEXT/5 (trauma* OR injur*)):ti,ab,kw
#5 ('intensive care'/mj OR 'emergency'/mj OR ("life threatening" OR "critical care" OR emergen*):ti,ab,kw) AND (trauma* OR injur*):ti,ab,kw
#6 #1 OR #2 OR #3 OR #4 #5
#7 'animals'/exp NOT 'humans'/de
#8 (comment OR editorial OR letter):it OR "case report*":ti,ab,kw
#9 [embase]/lim
#10 embase NOT (embase AND medline)
#11 'intraosseous drug administration'/exp
#12 (intraosseous NEAR/2 (infusion? OR puncture? OR access OR device?)):ti,ab,kw
#13 #11 OR #12
#14 #6 AND #13
#15 #14 NOT #7
#16 #15 NOT #8
#17 #16 AND #9
#18 #17 AND #10
#19 #18 AND ('article'/it OR 'article in press'/it OR 'review'/it)
#20 [1-1-1947]/sd NOT [23-3-2021]/sd
#21 #19 AND #20

Search strategy 2021, Additional questions on access/catheterisation, Embase (via Elsevier)
Date: 06.05.2021 **17 Hits**

#1 'multiple trauma'/exp
#2 (polytrauma* OR "trauma patient?"):ti,ab,kw OR (severe NEXT/2 shock):ti,ab,kw
#3 ((multiple OR major OR severe* OR serious*) NEXT/3 (trauma* OR injur*)):ti,ab,kw
#4 ((blunt OR penetrating) NEXT/5 (trauma* OR injur*)):ti,ab,kw
#5 ('intensive care'/mj OR 'emergency'/mj OR ("life threatening" OR "critical care" OR emergen*):ti,ab,kw) AND (trauma* OR injur*):ti,ab,kw
#6 #1 OR #2 OR #3 OR #4 #5
#7 'animals'/exp NOT 'humans'/de
#8 (comment OR editorial OR letter):it OR "case report*":ti,ab,kw
#9 [embase]/lim
#10 embase NOT (embase AND medline)

#11 'hemorrhagic shock'/exp OR 'hypovolemic shock'/exp OR 'traumatic shock'/exp OR 'exsanguination'/exp OR 'hypovolemia'/exp

#12 (((uncontrolled OR uncontrollable OR acute OR active OR massive OR "life threatening" OR severe) NEAR/2 (bleeding OR h$emorrhage)) OR ((hypovol$emic OR h$emorrhagic OR traumatic) NEXT/1 (shock OR trauma*)) OR "damage control resuscitation"):ti,ab,kw

#13 'hemostasis'/exp OR 'blood clotting'/exp OR 'disseminated intravascular clotting'/exp OR 'bleeding'/exp

#14 (h$emosta* OR coagula* OR clotting OR coagulopath*OR h$emorrhag* OR bleed*):ti,ab,kw

#15 #11 OR #12 OR #13 OR #14

#16 'central venous catheterization'/exp

#17 (((“heat exchang*” OR thermoregulatory OR warming OR hypothermia) NEXT/4 catheter) OR ((endovascular OR intravascular) NEXT/1 cooling)):ti,ab,kw OR (((“central venous” OR arterial) NEXT/1 catheter*) AND (subclavian OR femoral* OR jugular*)):ti,ab,kw OR (((intravascular OR vascular OR intravenous OR venous OR i.v. OR vein OR arterial) NEXT/1 (access* or cannulation)):ti,ab,kw AND ('echography'/exp OR (ultrasonography* OR ultrasound):ti,ab,kw))

#18 #16 OR #17

#19 #6 AND #15 AND #18

#20 #19 NOT #7

#21 #20 NOT #8

#22 #21 AND #9

#23 #22 AND #10

#24 #23 AND ('article'/it OR 'review'/it)

#25 [1-1-1947]/sd NOT [23-3-2021]/sd

#26 #15 AND #20

Table S3. List of excluded studies

Study	Year	Title	Reason for exclusion
Abdoulhossein, D., I. Taheri, M. A. Saba, H. Akbari, S. Shafagh and A. Zataollah	2018	Effect of vitamin C and vitamin E on lung contusion: A randomized clinical trial study	not relevant to any question
Abt, R., T. Lustenberger, J. F. Stover, E. Benninger, P. M. Lenzlinger, R. Stocker and M. Keel	2009	Base excess determined within one hour of admission predicts mortality in patients with severe pelvic fractures and severe hemorrhagic shock	Study type/Outcome
Adair, K. E., J. D. Patrick, E. J. Kliber, M. N. Peterson, Holl and S. R.	2020	TXA (Tranexamic Acid) Risk Evaluation in Combat Casualties (TRECC)	Study type/Outcome
Ageron, F. X., A. Gayet-Ageron, K. Ker, T. J. Coats, H. Shakur-Still, I. Roberts and C. Antifibrinolytics Trials	2020	Effect of tranexamic acid by baseline risk of death in acute bleeding patients: a meta-analysis of individual patient-level data from 28 333 patients	Population
Ageron, F. X., T. J. Coats, V. Darioli and I. Roberts	2021	Validation of the BATT score for prehospital risk stratification of traumatic haemorrhagic death: usefulness for tranexamic acid treatment criteria	Study type/Outcome
Agyabeng-Dadzie, K., J. E. Hunter, T. R. Smith, M. Jordan, K. Safcsak, J. A. Ibrahim, M. L. Cheatham and I. S. Bhullar	2020	Antiplatelet Agent Reversal Is Unnecessary in Blunt Traumatic Brain Injury Patients Not Requiring Immediate Craniotomy	Study type/Outcome
Albreiki, M. and D. Voegeli	2018	Permissive hypotensive resuscitation in adult patients with traumatic haemorrhagic shock: a systematic review	Multiple publication without additional information
Allam, M. G. I. M.	2020	Activated factor seven (Afvii) versus aminocaproic acid for treatment of traumatic retro-peritoneal hematoma	not relevant to any question
Almuwallad, A., E. Cole, J. Ross, Z. Perkins and R. Davenport	2021	The Impact of Pre-Hospital TXA on Mortality among Bleeding Trauma Patients: A Systematic Review and Meta-Analysis	Study type/Outcome
Alsawadi, A.	2012	The clinical effectiveness of permissive hypotension in blunt abdominal trauma with hemorrhagic shock but without head or spine injuries or burns: a systematic review	Population
Anderson, T. N., H. E. Hinson, E. N. Dewey, E. A. Rick, M. A. Schreiber and S. E. Rowell	2020	Early Tranexamic Acid Administration After Traumatic Brain Injury Is Associated With Reduced Syndecan-1 and Angiopoietin-2 in Patients With Traumatic Intracranial Hemorrhage	Population
Auten, J. D., N. L. Lunceford, J. L. Horton, M. R. Galarneau, R. M. Galindo, C. D. Shepps, T. J. Zieber and C. B. Dewing	2015	The safety of early fresh, whole blood transfusion among severely battle injured at US Marine Corps forward surgical care facilities in Afghanistan	not relevant to any question
Avery, P., S. Morton, H. Tucker, L. Green, A. Weaver and R. Davenport	2020	Whole blood transfusion versus component therapy in adult trauma patients with acute major haemorrhage	Study type/Outcome
Baksaas-Aasen, K., S. Van Dieren, K. Balvers, N. P. Juffermans, P. A. Naess, C. Rourke, S. Eaglestone, S. R. Ostrowski, J. Stensballe, S. Stanworth, M. Maegele, J. C. Goslings, P. I. Johansson, K. Brohi, C. Gaarder and T. I. collaborators	2019	Data-driven Development of ROTEM and TEG Algorithms for the Management of Trauma Hemorrhage: A Prospective Observational Multicenter Study	Population

Study	Year	Title	Reason for exclusion
Barrett, C. D., N. Vigneshwar, H. B. Moore, A. Ghasabyan, Ch, J. ler, E. E. Moore and M. B. Yaffe	2020	Tranexamic acid is associated with reduced complement activation in trauma patients with hemorrhagic shock and hyperfibrinolysis on thromboelastography	Study type/Outcome
Berndtson, A. E., T. W. Costantini, J. Lane, K. Box and R. Coimbra	2016	If some is good, more is better: An enoxaparin dosing strategy to improve pharmacologic venous thromboembolism prophylaxis	Study type/Outcome
Bohonek, M., D. Kutac, L. ova, M. Koranova, E. Sladkova, E. Staskova, M. Voldrich and T. Tyll	2019	The use of cryopreserved platelets in the treatment of polytraumatic patients and patients with massive bleeding	Population
Bohonek, M., D. Kutác, L. ová, M. Koránová, E. Sládková, E. Stašková, M. Voldrich and T. Tyll	2016	Frozen platelets in clinical praxis: Comparative study of native platelets	Sprache
Boon, Y., W. S. Kuan, Y. H. Chan, I. Ibrahim and M. T. Chua	2021	Agreement between arterial and venous blood gases in trauma resuscitation in emergency department (AGREE)	Population
Borgman, M. A., M. Zaar, J. K. Aden, Z. J. Schlader, D. Gagnon, E. Rivas, J. Kern, N. J. Koons, V. A. Convertino, A. P. Cap, Cr and C. all	2019	Hemostatic responses to exercise, dehydration, and simulated bleeding in heat-stressed humans	Population
Boudreau, R. M., Deshp, K. K. e, G. M. Day, W. R. Hinckley, N. Harger, T. A. Pritts, A. T. Makley and M. D. Goodman	2019	Prehospital Tranexamic Acid Administration During Aeromedical Transport After Injury	Study type/Outcome
Boutin, A., L. Moore, F. Lauzier, M. Chasse, S. English, R. Zarychanski, L. McIntyre, D. Griesdale, D. A. Fergusson and A. F. Turgeon	2017	Transfusion of red blood cells in patients with traumatic brain injuries admitted to Canadian trauma health centres: a multicentre cohort study	Population
Bradburn, E. H., K. M. Ho, M. E. Morgan, L. D'Andrea, T. M. Vernon and F. B. Rogers	2021	Massive Transfusion Protocol and Subsequent Development of Venous Thromboembolism: Statewide Analysis	Population
Brilej, D., D. Stropnik, R. Lefering and R. Komadina	2017	Algorithm for activation of coagulation support treatment in multiple injured patients--cohort study	not relevant to any question
Brohi, K. and S. Eaglestone	2017	Traumatic coagulopathy and massive transfusion: improving outcomes and saving blood	Study type/Outcome
Brown, J. B., M. D. Neal, F. X. Guyette, A. B. Peitzman, T. R. Billiar, B. S. Zuckerbraun and J. L. Sperry	2015	Design of the Study of Tranexamic Acid during Air Medical Prehospital Transport (STAAMP) Trial: Addressing the Knowledge Gaps	Study type/Outcome
Bugaev, N., J. J. Como, G. Golani, J. J. Freeman, J. S. Sawhney, C. J. Vatsaas, B. K. Yorkgitis, L. A. Kreiner, N. M. Garcia, H. A. Aziz, P. A. Pappas, E. J. Mahoney, Z. W. Brown and G. Kasotakis	2020	Thromboelastography and rotational thromboelastometry in bleeding patients with coagulopathy: Practice management guideline from the Eastern Association for the Surgery of Trauma	Study type/Outcome
Byars, D. V., S. N. Tsuchitani, E. Erwin, B. Anglemyer and J. Eastman	2011	Evaluation of success rate and access time for an adult sternal intraosseous device deployed in the prehospital setting	Population
Callcut, R. A., M. W. Cripps, M. F. Nelson, A. S. Conroy, B. B. Robinson and M. J. Cohen	2016	The Massive Transfusion Score as a decision aid for resuscitation: Learning when to turn the massive transfusion protocol on and off	Study type/Outcome
Cannon, J. W., M. A. Khan, A. S. Raja, M. J. Cohen, J. J. Como, B. A. Cotton, J. J. Dubose, E. E. Fox, K. Inaba, C. J. Rodriguez, J. B. Holcomb and J. C. Duchesne	2017	Damage control resuscitation in patients with severe traumatic hemorrhage: A practice management guideline from the Eastern Association for the Surgery of Trauma	Study type/Outcome

Study	Year	Title	Reason for exclusion
Carothers, C., A. Giancarelli, J. Ibrahim and B. Hobbs	2018	Activated prothrombin complex concentrate for warfarin reversal in traumatic intracranial hemorrhage	Study type/Outcome
Chakroun-Walha, O., A. Samet, M. Jerbi, A. Nasri, A. Talbi, H. Kanoun, B. Souissi, K. Chtara, M. Bouaziz, H. Ksibi and N. Rekik	2019	Benefits of the tranexamic acid in head trauma with no extracranial bleeding: a prospective follow-up of 180 patients	Population
Chang, R., E. E. Fox, T. J. Greene, M. D. Swartz, S. M. DeSantis, D. M. Stein, E. M. Bulger, S. M. Melton, M. D. Goodman, M. A. Schreiber, M. D. Zielinski, T. O'Keeffe, K. Inaba, J. S. Tomasek, J. M. Podbielski, S. Appana, M. Yi, P. I. Johansson, H. H. He	2018	Abnormalities of laboratory coagulation tests versus clinically evident coagulopathic bleeding: results from the prehospital resuscitation on helicopters study (PROHS)	Study type/Outcome
Chapman, M. P., E. E. Moore, T. L. Chin, A. Ghasabyan, Ch, J. ler, J. Stringham, E. Gonzalez, H. B. Moore, A. Banerjee, C. C. Silliman and A. Sauaia	2015	Combat: Initial Experience with a Randomized Clinical Trial of Plasma-Based Resuscitation in the Field for Traumatic Hemorrhagic Shock	Multiple publication without additional information
Choi, S., M. H. Rahbar, J. Ning, D. J. Del Junco, E. Rahbar, C. Hong, J. Piao, E. E. Fox and J. B. Holcomb	2016	Recurrent event frailty models reduced time-varying and other biases in evaluating transfusion protocols for traumatic hemorrhage	Study type/Outcome
Chow, J. H., B. Fedeles, J. E. Richards, K. A. Tanaka, J. J. Morrison, P. Rock, T. M. Scalea, M. A. Mazzeffi and T. R.-T. Investigators	2020	Thromboelastography Reaction-Time Thresholds for Optimal Prediction of Coagulation Factor Deficiency in Trauma	Study type/Outcome
Coats, T. J. and M. Morsy	2020	Biological mechanisms and individual variation in fibrinolysis after major trauma	Intervention
Coccolini, F., G. Pizzilli, D. Corbella, M. Sartelli, V. Agnoletti, V. Agostini, G. L. Baiocchi, L. Ansaloni and F. Catena	2019	Pre-hospital plasma in haemorrhagic shock management: current opinion and meta-analysis of randomized trials	not relevant to any question
Cole, E., R. Davenport, K. Willett and K. Brohi	2015	Tranexamic acid use in severely injured civilian patients and the effects on outcomes: a prospective cohort study	not relevant to any question
Connelly, C. R., P. Y. Van, K. D. Hart, S. G. Louis, K. A. Fair, A. S. Erickson, E. A. Rick, E. C. Simeon, E. M. Bulger, S. Arbabi, J. B. Holcomb, L. J. Moore and M. A. Schreiber	2016	Thrombelastography-Based Dosing of Enoxaparin for Thromboprophylaxis in Trauma and Surgical Patients: A Randomized Clinical Trial	Population
Consunji, R., A. Elseed, A. El-Menyar, B. Sathian, S. Rizoli, H. Al-Thani and R. Peralta	2020	The effect of massive transfusion protocol implementation on the survival of trauma patients: a systematic review and meta-analysis	Population
Corbett, J. M., K. M. Ho and S. Honeybul	2019	Prognostic significance of abnormal hematological parameters in severe traumatic brain injury requiring decompressive craniectomy	Study type/Outcome
Cornero, S. G., M. Maegele, R. Lefering, C. Abbati, S. Gupta, F. Sammartano, S. Cimbanassi and O. Chiara	2020	Predictive Factors for Massive Transfusion in Trauma: A Novel Clinical Score from an Italian Trauma Center and German Trauma Registry	Study type/Outcome
Cruciani, M., M. Franchini, C. Mengoli, G. Marano, I. Pati, F. Masiello, E. Veropalumbo, S. Pupella, S. Vaglio, V. Agostini and G. M. Liumbruno	2021	The use of whole blood in traumatic bleeding: a systematic review	Multiple publication without additional information

Study	Year	Title	Reason for exclusion
Curry, N., C. Rourke, R. Davenport, S. Beer, L. Pankhurst, A. Deary, H. Thomas, C. Llewelyn, L. Green, H. Doughty, G. Nordmann, K. Brohi and S. Stanworth	2015	Early cryoprecipitate for major haemorrhage in trauma: a randomised controlled feasibility trial	not relevant to any question
Da Luz, L. T., B. Nascimento, A. K. Shankarakutty, S. Rizoli and N. K. Adhikari	2014	Effect of thromboelastography (TEG R) and rotational thromboelastometry (ROTEM R) on diagnosis of coagulopathy, transfusion guidance and mortality in trauma: descriptive systematic review	Study type/Outcome
da Luz, L. T., P. S. Shah, R. Strauss, A. A. Mohammed, P. P. D'Empaire, H. Tien, A. B. Nathens and B. Nascimento	2019	Does the evidence support the importance of high transfusion ratios of plasma and platelets to red blood cells in improving outcomes in severely injured patients: a systematic review and meta-analyses	Multiple publication without additional information
de Crescenzo, C., F. Gorouhi, E. S. Salcedo and J. M. Galante	2017	Prehospital hypertonic fluid resuscitation for trauma patients: A systematic review and meta-analysis	Multiple publication without additional information
Dias, J. D., C. G. Lopez-Espina, J. Ippolito, L. H. Hsiao, F. Zaman, A. A. Muresan, S. G. Thomas, M. Walsh, A. J. Jones, A. Grisoli, B. C. Thurston, R. Artang, K. P. Bilden, J. Hartmann and H. E. Achneck	2019	Rapid point-of-care detection and classification of direct-acting oral anticoagulants with the TEG 6s: Implications for trauma and acute care surgery	Population
Ditillo, M., K. Hanna, L. Castanon, M. Zeeshan, N. Kulvatunyou, A. Tang, J. Sakran, L. Gries and B. Joseph	2020	The role of cryoprecipitate in massively transfused patients: Results from the Trauma Quality Improvement Program database may change your mind	not relevant to any question
Dubendorfer, C., A. T. Billeter, B. Seifert, M. Keel and M. Turina	2013	Serial lactate and admission SOFA scores in trauma: an analysis of predictive value in 724 patients with and without traumatic brain injury	Study type/Outcome
Duchesne, J., A. Smith, S. Lawicki, J. Hunt, A. Houghton, S. Taghavi, R. Schroll, O. Jackson-Weaver, C. Guidry and D. Tatum	2020	Single Institution Trial Comparing Whole Blood vs Balanced Component Therapy: 50 Years Later	not relevant to any question
Dunham, C. M., R. J. Malik, G. S. Huang, C. M. Kohli, B. P. Brocker and K. T. Ugokwe	2018	Hypertonic saline administration and complex traumatic brain injury outcomes: a retrospective study	Study type/Outcome
Ebrahimi, P., J. Mozafari, R. B. Ilkhchi, M. G. Hanafi and M. Mousavinejad	2019	Intravenous Tranexamic Acid for Subdural and Epidural Intracranial Hemorrhage: Randomized, Double-Blind, Placebo-Controlled Trial	Population
Ellenberger, C., N. Garofano, G. Barcelos, J. Diaper, G. Pavlovic and M. Licker	2017	Assessment of Haemostasis in patients undergoing emergent neurosurgery by rotational Elastometry and standard coagulation tests: a prospective observational study	Population
El-Menyar, A., B. Sathian, M. Asim, R. Latifi and H. Al-Thani	2018	Efficacy of prehospital administration of tranexamic acid in trauma patients: A meta-analysis of the randomized controlled trials	Study type/Outcome
Endo, A., A. Shiraishi, Y. Otomo, S. Kushimoto, D. Saitoh, M. Hayakawa, H. Ogura, K. Murata, A. Hagiwara, J. Sasaki, T. Matsuoka, T. Uejima, N. Morimura, H. Ishikura, M. Takeda, N. Kaneko, H. Kato, D. Kudo, T. Kanemura, T. Shibusawa, Y. Hagiwara, S. Furug	2016	Development of Novel Criteria of the "Lethal Triad" as an Indicator of Decision Making in Current Trauma Care: A Retrospective Multicenter Observational Study in Japan	not relevant to any question

Study	Year	Title	Reason for exclusion
Fadeyi, E. A., A. K. Saha, T. Naal, H. Martin, E. Fenu, J. H. Simmons, M. R. Jones and G. J. Pomper	2020	A comparison between leukocyte reduced low titer whole blood vs non-leukocyte reduced low titer whole blood for massive transfusion activation	not relevant to any question
Faraoni, D. and P. D. Van Linden	2014	A systematic review of antifibrinolytics and massive injury	Multiple publication without additional information
Fei, A., Q. Lin, J. Liu, F. Wang, H. Wang and S. Pan	2015	The relationship between coagulation abnormality and mortality in ICU patients: a prospective, observational study	Population
Fischer, M., P. Lackner, R. Beer, R. Helbok, B. Pfausler, D. Schneider, E. Schmutzhard and G. Broessner	2015	Cooling Activity is Associated with Neurological Outcome in Patients with Severe Cerebrovascular Disease Undergoing Endovascular Temperature Control	Population
Fletcher-S, A. ersjoo, E. P. Thelin, M. Maegele, M. Svensson, Bell and B. M. er	2020	Time Course of Hemostatic Disruptions After Traumatic Brain Injury: A Systematic Review of the Literature	Population
Fragou, M., A. Gravvanis, V. Dimitriou, A. Papalois, G. Kouraklis, A. Karabinis, T. Saranteas, J. Poularas, J. Papanikolaou, P. Davlouros, N. Labropoulos and D. Karakitsos	2011	Real-time ultrasound-guided subclavian vein cannulation versus the landmark method in critical care patients: a prospective randomized study	Population
Franz, N. D., Machado-Ar, D. a, J. T. Miller and N. Farina	2020	Impact of Obesity on Tranexamic Acid Efficacy in Adult Patients With Major Bleeding	Study type/Outcome
Fu, H. P., Y. X. Zhang, Y. F. Wei, Z. Q. Wang, G. X. Wang and X. L. Li	2018	Correlation analysis between both the platelet-to-lymphocyte ratio and maximum amplitude of thrombus and death in severe traumatic patients	Sprache
Furmaga, W., S. Cohn, T. J. Prihoda, M. T. Muir, V. Mikhailov, J. McCarthy and Y. Arar	2015	Novel markers predict death and organ failure following hemorrhagic shock	not relevant to any question
Gao, Y. K., Gui, C. J., Xin, W. Q., Hu, D., Yang, X. Y.	2019	Assessment of mild hypothermia combined with edaravone for the treatment of severe craniocerebral injury	Intervention
Giancarelli, A., K. L. Birrer, R. F. Alban, B. P. Hobbs and X. Liu-DeRyke	2016	Hypocalcemia in trauma patients receiving massive transfusion	Study type/Outcome
Godfrey, B. W., A. Martin, P. J. Chestovich, G. H. Lee, N. K. Ingalls and V. Saldanha	2017	Patients with multiple traumatic amputations: An analysis of operation enduring freedom joint theatre trauma registry data	Intervention
Gonzalez-Guerrero, C., T. Lozano-Andreu, M. Roch-Santed, L. Rivera-Sanchez, Br, D. ariz-Nunez, L. Pasto-Cardona, J. C. Juarez-Gimenez and J. B. Montoro-Ronsano	2017	Evaluation of the efficiency under current use of human fibrinogen concentrate in trauma patients with life-threatening hemorrhagic disorders	Intervention
Gozal, Y. M., C. P. Carroll, B. M. Krueger, J. Khoury and N. O. Andaluz	2017	Point-of-care testing in the acute management of traumatic brain injury: Identifying the coagulopathic patient	Study type/Outcome
Grba-Bujević, M., I. Bošan-Kilibarda and N. Strikić	2012	The use of hypertonic-hyperoncotic solution for hypovolemic shock in trauma patients in prehospital setting	Study type/Outcome
Gunning, A. C., R. V. Maier, D. de Rooij, L. P. H. Leenen and F. Hietbrink	2021	Venous thromboembolism (VTE) prophylaxis in severely injured patients: an international comparative assessment	Study type/Outcome

Study	Year	Title	Reason for exclusion
Guo, S. B., Y. X. Chen and X. Z. Yu	2017	Clinical Characteristics and Current Interventions in Shock Patients in Chinese Emergency Departments: A Multicenter Prospective Cohort Study	Intervention
Haas, T., K. Gorlinger, A. Grassetto, V. Agostini, P. Simioni, G. Nardi and M. Ranucci	2014	Thromboelastometry for guiding bleeding management of the critically ill patient: a systematic review of the literature	Study type/Outcome
Hanna, K., L. Bible, M. Chehab, S. Asmar, M. Douglas, M. Ditillo, L. Castanon, A. Tang and B. Joseph	2020	Nationwide analysis of whole blood hemostatic resuscitation in civilian trauma	not relevant to any question
Hartholt, K. A., E. M. M. van Lieshout, W. C. Thies, P. Patka and I. B. Schipper	2010	Intraosseous devices: a randomized controlled trial comparing three intraosseous devices	not relevant to any question
Haverkamp, F. J. C., G. G. Giesbrecht and E. Tan	2018	The prehospital management of hypothermia - An up-to-date overview	Study type/Outcome
Hazelton, J. P., J. W. Cannon, C. Zatorski, J. S. Roman, S. A. Moore, A. J. Young, M. Subramanian, J. F. Guzman, F. Fogt, A. Moran, J. Gaughan, M. J. Seamon and J. Porter	2019	Cold-stored whole blood: A better method of trauma resuscitation?	not relevant to any question
Heidari, K., M. Taghizadeh, S. Mahmoudi, H. Panahi, E. Ghaffari Shad and S. Asadollahi	2017	FAST for blunt abdominal trauma: Correlation between positive findings and admission acid-base measurement	Population
Heming, N., S. Elatrous, S. Jaber, A. S. Dumenil, J. Cousson, X. Forceville, A. Kimmoun, J. L. Trouillet, J. Fichet, N. Anguel, M. Darmon, C. Martin, S. Chevret, D. Annane and C. Investigators	2017	Haemodynamic response to crystalloids or colloids in shock: an exploratory subgroup analysis of a randomised controlled trial	Population
Hoedemaekers, C. W., M. Ezzahti, A. Gerritsen and J. G. van der Hoeven	2007	Comparison of cooling methods to induce and maintain normo- and hypothermia in intensive care unit patients: a prospective intervention study	Population
Holcomb, J. B., D. P. Donathan, B. A. Cotton, D. J. Del Junco, G. Brown, T. V. Wenckstern, J. M. Podbielski, E. A. Camp, R. Hobbs, Y. Bai, M. Brito, E. Hartwell, J. R. Duke and C. E. Wade	2015	Prehospital Transfusion of Plasma and Red Blood Cells in Trauma Patients	Population
Huang, G. S. and C. M. Dunham	2017	Mortality outcomes in trauma patients undergoing prehospital red blood cell transfusion: a systematic literature review	Study type/Outcome
Huebner, B. R., W. C. Dorlac and C. Cribari	2017	Tranexamic Acid Use in Prehospital Uncontrolled Hemorrhage	Population
Human, T., A. M. Cook, B. Anger, K. Bledsoe, A. Castle, D. Deen, H. Gibbs, C. Lesch, N. Liang, K. McAllen, C. Morrison, D. Parker, Jr., A. S. Rowe, D. Rhoney, K. Sangha, E. Santayana, S. Taylor, E. Tesoro and G. Brophy	2017	Treatment of Hyponatremia in Patients with Acute Neurological Injury	Population
Hunt, H., S. Stanworth, N. Curry, T. Woolley, C. Cooper, O. Ukoumunne, Z. Zhelev and C. Hyde	2015	Thromboelastography (TEG) and rotational thromboelastometry (ROTEM) for trauma induced coagulopathy in adult trauma patients with bleeding	Study type/Outcome
Imran, J. B., T. D. Madni, A. T. Clark, P. Rizk, E. Huang, C. T. Minshall, L. R. Taveras, H. B. Cunningham, A. L. Eastman, J. P. Koshy, C. D. Kacir and M. W. Cripps	2018	Inability to predict subprophylactic anti-factor Xa levels in trauma patients receiving early low-molecular-weight heparin	Study type/Outcome

Study	Year	Title	Reason for exclusion
Ishii, K., T. Kinoshita, K. Kiridume, A. Watanabe, K. Yamakawa, S. Nakao, S. Fujimi and T. Matsuoka	2019	Impact of initial coagulation and fibrinolytic markers on mortality in patients with severe blunt trauma: a multicentre retrospective observational study	Study type/Outcome
Jachetti, A., R. B. Massenat, N. Edema, S. C. Woolley, G. Benedetti, R. Van Den Bergh and M. Trelles	2019	Introduction of a standardised protocol, including systematic use of tranexamic acid, for management of severe adult trauma patients in a low-resource setting: the MSF experience from Port-au-Prince, Haiti	Study type/Outcome
Javali, R. H., P. Ravindra, A. Patil, M. Srinivasarangan, H. Mundada, S. B. Adarsh and S. Nisarg	2017	A Clinical Study on the Initial Assessment of Arterial Lactate and Base Deficit as Predictors of Outcome in Trauma Patients	Population
Javier, P. T. C., G. G. Celia, B. R. Virginia, M. T. Javier, L. A. Antonio and M. R. J. Bruno	2019	Use of fibrinogen concentrate in surgical and bleeding trauma patients	Study type/Outcome
Jehan, F., T. O'Keeffe, M. Khan, A. Chi, A. Tang, N. Kulvatunyou, L. Gries and B. Joseph	2017	Early thromboprophylaxis with low-molecular-weight heparin is safe in patients with pelvic fracture managed nonoperatively	Population
Jensen, K. O., L. Held, A. Kraus, Hildebr, F. , P. Mommsen, L. Mica, G. A. Wanner, P. Steiger, R. M. Moos, H. P. Simmen and K. Sprengel	2016	The impact of mild induced hypothermia on the rate of transfusion and the mortality in severely injured patients: a retrospective multi-centre study	Study type/Outcome
Johnston, L. R., C. J. Rodriguez, E. A. Elster and M. J. Bradley	2018	Evaluation of Military Use of Tranexamic Acid and Associated Thromboembolic Events	Study type/Outcome
Jokar, A., K. Ahmadi, T. Salehi, M. Sharif-Alhoseini and V. Rahimi-Movaghar	2017	The effect of tranexamic acid in traumatic brain injury: A randomized controlled trial	Population
Joseph, B., H. Aziz, M. Snell, V. Pandit, D. Hays, N. Kulvatunyou, A. Tang, T. O'Keeffe, J. Wynne, R. S. Friese and P. Rhee	2014	The physiological effects of hyperosmolar resuscitation: 5% vs 3% hypertonic saline	Study type/Outcome
Joseph, B., V. it, C. Harrison, D. Lubin, N. Kulvatunyou, B. Zangbar, A. Tang, T. O'Keeffe, D. J. Green, L. Gries, R. S. Friese and P. Rhee	2015	Early thromboembolic prophylaxis in patients with blunt solid abdominal organ injuries undergoing nonoperative management: is it safe?	Study type/Outcome
Jouffroy, R. and B. Vivien	2020	Prehospital Plasma Transfusion and Survival in Trauma Patients With Hemorrhagic Shock	Study type/Outcome
Kang, W. S., I. S. Shin, J. S. Pyo, S. Ahn, S. Chung, Y. J. Ki, J. Seok, C. Y. Park and S. Lee	2019	Prognostic Accuracy of Massive Transfusion, Critical Administration Threshold, and Resuscitation Intensity in Assessing Mortality in Traumatic Patients with Severe Hemorrhage: a Meta-Analysis	Study type/Outcome
Kaserer, A., M. Casutt, K. Sprengel, B. Seifert, D. R. Spahn and P. Stein	2018	Comparison of two different coagulation algorithms on the use of allogenic blood products and coagulation factors in severely injured trauma patients: a retrospective, multicentre, observational study	Study type/Outcome
Kasotakis, G., N. Starr, E. Nelson, B. Sarkar, P. A. Burke, D. G. Remick, R. G. Tompkins, Inflammation and I. Host Response to Injury	2019	Platelet transfusion increases risk for acute respiratory distress syndrome in non-massively transfused blunt trauma patients	not relevant to any question
Ker, K., I. Roberts, H. Shakur and T. J. Coats	2015	Antifibrinolytic drugs for acute traumatic injury	Population

Study	Year	Title	Reason for exclusion
Khan, S., R. Davenport, I. Raza, S. Glasgow, H. D. De'Ath, P. I. Johansson, N. Curry, S. Stanworth, C. Gaarder and K. Brohi	2015	Damage control resuscitation using blood component therapy in standard doses has a limited effect on coagulopathy during trauma hemorrhage	Study type/Outcome
Kim, J. H., A. Nagy, A. Putzu, A. Belletti, G. Biondi-Zoccai, V. V. Likhvantsev, A. G. Yavorovskiy and G. oni	2020	Therapeutic Hypothermia in Critically Ill Patients: A Systematic Review and Meta-Analysis of High Quality Randomized Trials	Population
Kornblith, L. Z., A. J. Robles, A. S. Conroy, B. J. Redick, B. M. Howard, C. M. Hendrickson, S. Moore, M. F. Nelson, F. Moazed, R. A. Callcut, C. S. Calfee and M. J. Cohen	2019	Predictors of postinjury acute respiratory distress syndrome: Lung injury persists in the era of hemostatic resuscitation	Study type/Outcome
Kreutziger, J., A. Rafetseder, S. Mathis, V. Wenzel, R. El Attal and S. Schmid	2015	Admission blood glucose predicted haemorrhagic shock in multiple trauma patients	Study type/Outcome
Kutcher, M. E., B. M. Howard, J. L. Sperry, A. E. Hubbard, A. L. Decker, J. Cuschieri, J. P. Minei, E. E. Moore, B. H. Brownstein, R. V. Maier and M. J. Cohen	2015	Evolving beyond the vicious triad: Differential mediation of traumatic coagulopathy by injury, shock, and resuscitation	Intervention
Kwan, I., F. Bunn, P. Chinnock and I. Roberts	2014	Timing and volume of fluid administration for patients with bleeding	Population
Laursen, T. H., M. A. S. Meyer, A. S. P. Meyer, T. Gaarder, P. A. Naess, J. Stensballe, S. R. Ostrowski and P. I. Johansson	2018	Thrombelastography early amplitudes in bleeding and coagulopathic trauma patients: Results from a multicenter study	Population
Lefering, R., D. Zielske, B. Bouillon, C. Hauser and H. Levy	2013	Lactic acidosis is associated with multiple organ failure and need for ventilator support in patients with severe hemorrhage from trauma	Intervention
Leidel, B. A., C. Kirchhoff, V. Bogner, J. Stegmaier, W. Mutschler, K.-G. Kanz and V. Braunstein	2009	Is the intraosseous access route fast and efficacious compared to conventional central venous catheterization in adult patients under resuscitation in the emergency department? A prospective observational pilot study	Population
Leidel, B. A., C. Kirchhoff, V. Braunstein, V. Bogner, P. Biberthaler and K.-G. Kanz	2010	Comparison of two intraosseous access devices in adult patients under resuscitation in the emergency department: A prospective, randomized study	Population
Lewis, C. J., P. Li, L. Stewart, A. C. Weintrob, M. L. Carson, C. K. Murray, D. R. Tribble and J. D. Ross	2016	Tranexamic acid in life-threatening military injury and the associated risk of infective complications	Population
Lewis, S. R., M. W. Pritchard, D. J. Evans, A. R. Butler, P. Alderson, A. F. Smith and I. Roberts	2018	Colloids versus crystalloids for fluid resuscitation in critically ill people	Population
Lim, G., K. Harper-Kirksey, R. Parekh and A. F. Manini	2018	Efficacy of a massive transfusion protocol for hemorrhagic trauma resuscitation	Study type/Outcome
Lin, W., X. Lin, Y. Zhuang, X. Pan, C. Wu, S. Zhang, L. Zhang, J. Lin, S. Shi and S. Shi	2019	Significance of Early Postoperative Arterial Lactic Acid, Inferior Vena Cava Variability, and Central Venous Pressure in Hypovolemic Shock	Study type/Outcome
Liu, Y. H., Z. D. Shang, C. Chen, N. Lu, Q. F. Liu, M. Liu and J. Yan	2015	'Cool and quiet' therapy for malignant hyperthermia following severe traumatic brain injury: A preliminary clinical approach	Study type/Outcome
Lombardo, S., D. Millar, G. J. Jurkovich, R. Coimbra and R. Nirula	2018	Factor VIIa administration in traumatic brain injury: an AAST-MITC propensity score analysis	Intervention
Luehr, E., G. Grone, M. Pathak, C. Austin and S. Thompson	2017	Administration of tranexamic acid in trauma patients under stricter inclusion criteria increases the treatment window for stabilization from 24 to 48 hours-a retrospective review	Study type/Outcome

Study	Year	Title	Reason for exclusion
Lui, C. T., O. F. Wong, K. L. Tsui, C. W. Kam, S. M. Li, M. Cheng and K. K. G. Leung	2018	Predictive model integrating dynamic parameters for massive blood transfusion in major trauma patients: The Dynamic MBT score	Population
Mackenzie, C. F., Y. Wang, P. F. Hu, S. Y. Chen, H. H. Chen, G. Hagegeorge, L. G. Stansbury, S. Shackelford and O. S. Group	2014	Automated prediction of early blood transfusion and mortality in trauma patients	Intervention
Madden, L. K., M. Hill, T. L. May, T. Human, M. M. Guanci, J. Jacobi, M. V. Moreda and N. Badjatia	2017	The Implementation of Targeted Temperature Management: An Evidence-Based Guideline from the Neurocritical Care Society	Study type/Outcome
Mahmood, A., K. Needham, H. Shakur-Still, T. Harris, S. F. Jamaluddin, D. Davies, A. Belli, F. L. Mohamed, C. Leech, H. M. Lotfi, P. Moss, F. Lecky, P. Hopkins, D. Wong, A. Boyle, M. Wilson, M. Darwent and I. Roberts	2020	Effect of tranexamic acid on intracranial haemorrhage and infarction in patients with traumatic brain injury: a pre-planned substudy in a sample of CRASH-3 trial patients	Population
Malkin, M., A. Nevo, S. I. Brundage and M. Schreiber	2021	Effectiveness and safety of whole blood compared to balanced blood components in resuscitation of hemorrhaging trauma patients - A systematic review	Study type/Outcome
Mangram, A., O. F. Oguntodu, Dz, J. K. u, A. K. Hollingworth, S. Hall, C. Cung, J. Rodriguez, I. Yusupov and J. F. Barletta	2016	Is there a difference in efficacy, safety, and cost-effectiveness between 3-factor and 4-factor prothrombin complex concentrates among trauma patients on oral anticoagulants?	Population
Marsden, M. E. R., A. Rossetto, C. A. B. Duffield, T. G. D. Woolley, W. P. Buxton, S. Steynberg, R. Bagga and N. R. M. Tai	2019	Prehospital tranexamic acid shortens the interval to administration by half in Major Trauma Networks: a service evaluation	Population
Masoumi, K., A. Forouzan, A. A. Darian and A. Rafaty Navaii	2016	Comparison of the Effectiveness of Hydroxyethyl Starch (Voluven) Solution With Normal Saline in Hemorrhagic Shock Treatment in Trauma	Study type/Outcome
Matsushima, K., E. Benjamin and D. Demetriades	2015	Prothrombin complex concentrate in trauma patients	Study type/Outcome
McCully, B. H., C. R. Connelly, K. A. Fair, J. B. Holcomb, E. E. Fox, C. E. Wade, E. M. Bulger, M. A. Schreiber and P. S. Group	2017	Onset of Coagulation Function Recovery Is Delayed in Severely Injured Trauma Patients with Venous Thromboembolism	Study type/Outcome
McMillen, J. C., C. M. Lawson and A. Shaun Rowe	2013	Futility Assessment of Recombinant Factor VII Activated for the Treatment of Hemorrhagic Shock Requiring Massive Transfusion	Study type/Outcome
McQuilten, Z. K., G. Crighton, S. Brunskill, J. K. Morison, T. H. Richter, N. Waters, M. F. Murphy and E. M. Wood	2018	Optimal Dose, Timing and Ratio of Blood Products in Massive Transfusion: Results from a Systematic Review	Population
McQuilten, Z. K., G. Crighton, S. Engelbrecht, R. Gotmaker, S. J. Brunskill, M. F. Murphy and E. M. Wood	2015	Transfusion interventions in critical bleeding requiring massive transfusion: a systematic review	Multiple publication without additional information
Meizoso, J. P., C. A. Karcutskie, J. J. Ray, N. Namias, C. I. Schulman and K. G. Proctor	2017	Persistent Fibrinolysis Shutdown Is Associated with Increased Mortality in Severely Injured Trauma Patients	Intervention
Mengoli, C., M. Franchini, G. Marano, S. Pupella, S. Vaglio, M. Marietta and G. M. Liumbruno	2017	The use of fibrinogen concentrate for the management of trauma-related bleeding: a systematic review and meta-analysis	Study type/Outcome

Study	Year	Title	Reason for exclusion
Mesghali, E., S. Fitter, K. Bahjri and K. Moussavi	2019	Safety of Peripheral Line Administration of 3% Hypertonic Saline and Mannitol in the Emergency Department	Study type/Outcome
Meyer, A. S., M. A. Meyer, A. M. Sorensen, L. S. Rasmussen, M. B. Hansen, J. B. Holcomb, B. A. Cotton, C. E. Wade, S. R. Ostrowski and P. I. Johansson	2014	Thrombelastography and rotational thromboelastometry early amplitudes in 182 trauma patients with clinical suspicion of severe injury	Study type/Outcome
Mica, L., H. Simmen, C. M. Werner, M. Plecko, C. Keller, S. H. Wirth and K. Sprengel	2016	Fresh frozen plasma is permissive for systemic inflammatory response syndrome, infection, and sepsis in multiple-injured patients	Study type/Outcome
Miyata, K., H. Ohnishi, K. Maekawa, T. Mikami, Y. Akiyama, S. Iihoshi, M. Wanibuchi, N. Mikuni, S. Uemura, K. Tanno, E. Narimatsu and Y. Asai	2016	Therapeutic temperature modulation in severe or moderate traumatic brain injury: a propensity score analysis of data from the Nationwide Japan Neurotrauma Data Bank	Intervention
Mizushima, Y., S. Nakao, K. Idoguchi and T. Matsuoka	2017	Fluid resuscitation of trauma patients: How much fluid is enough to determine the patient's response?	Intervention
Mojallal, F., M. Nikooieh, M. Hajimaghsoudi, M. Baqherabadi, M. Jafari, A. Esmaeili, N. M. Karimi and E. Zarepur	2020	The effect of intravenous tranexamic acid on preventing the progress of cerebral hemorrhage in patients with brain traumatic injuries compared to placebo: A randomized clinical trial	Population
Mok, G., R. Hoang, M. W. Khan, D. Pannell, H. Peng, H. Tien, A. Nathens, J. Callum, K. Karkouti, A. Beckett and L. T. da Luz	2021	Freeze-dried plasma for major trauma - Systematic review and meta-analysis	Study type/Outcome
Monsef Kasmaei, V., A. Javadi and S. A. Naseri Alavi	2019	Effects of tranexamic acid on reducing blood loss in pelvic trauma: A randomised double-blind placebo controlled study	Population
Moore, H. B., E. E. Moore, E. Gonzalez, M. P. Chapman, T. L. Chin, C. C. Silliman, A. Banerjee and A. Sauaia	2014	Hyperfibrinolysis, physiologic fibrinolysis, and fibrinolysis shutdown: the spectrum of postinjury fibrinolysis and relevance to antifibrinolytic therapy	Study type/Outcome
Moore, H. B., E. E. Moore, M. P. Chapman, K. C. Hansen, M. J. Cohen, F. M. Pieracci, Ch, J. ler and A. Sauaia	2019	Does Tranexamic Acid Improve Clot Strength in Severely Injured Patients Who Have Elevated Fibrin Degradation Products and Low Fibrinolytic Activity, Measured by Thrombelastography?	Population
Mousavinejad, M., J. Mozafari, R. B. Ilkhchi, M. G. Hanafi and P. Ebrahimi	2020	Intravenous Tranexamic Acid for Brain Contusion with Intraparenchymal Hemorrhage: Randomized, Double-Blind, Placebo-Controlled Trial	Population
Myers, S. P., M. R. Dyer, A. Hassoune, J. B. Brown, J. L. Sperry, M. P. Meyer, M. R. Rosengart and M. D. Neal	2020	Correlation of Thromboelastography with Apparent Rivaroxaban Concentration: Has Point-of-Care Testing Improved?	Population
Nadler, R., S. Gendler, A. Benov, R. Strugo, A. Abramovich and E. Glassberg	2014	Tranexamic acid at the point of injury: the Israeli combined civilian and military experience	Population
Naumann, D. N., J. Hazeldine, D. J. Davies, J. Bishop, M. J. Midwinter, A. Belli, P. Harrison and J. M. Lord	2018	Endotheliopathy of Trauma is an on-Scene Phenomenon, and is Associated with Multiple Organ Dysfunction Syndrome: A Prospective Observational Study	Study type/Outcome
Nederpelt, C. J., M. El Hechi, J. Parks, J. Fawley, A. E. Mendoza, N. Saillant, D. R. King, P. J. Fagenholz, G. C. Velmahos and H. M. A. Kaafarani	2020	The dose-dependent relationship between blood transfusions and infections after trauma: A population-based study	not relevant to any question

Study	Year	Title	Reason for exclusion
Neeki, M. M., F. Dong, J. Toy, J. Salameh, M. Rabiei, J. Powell, R. Vara, K. Inaba, D. Wong, M. E. Comunale, A. Lowe, Ch, D. wani, J. Quispe and R. Borger	2020	Safety and Efficacy of Hospital Utilization of Tranexamic Acid in Civilian Adult Trauma Resuscitation	not relevant to any question
Neeki, M. M., F. Dong, J. Toy, R. Vaezazizi, J. Powell, N. Jabourian, A. Jabourian, D. Wong, R. Vara, K. Seiler, T. W. Pennington, J. Powell, C. Yoshida-McMath, S. Kissel, K. Schulz-Costello, J. Mistry, M. S. Surrusco, K. R. O'Bosky, D. Van Stralen, D. Lu	2017	Efficacy and Safety of Tranexamic Acid in Prehospital Traumatic Hemorrhagic Shock: Outcomes of the Cal-PAT Study	Study type/Outcome
Nguyen, M., R. Pirracchio, L. Z. Kornblith, R. Callcut, E. E. Fox, C. E. Wade, M. Schreiber, J. B. Holcomb, J. Coyle, M. Cohen and A. Hubbard	2020	Dynamic impact of transfusion ratios on outcomes in severely injured patients: Targeted machine learning analysis of the Pragmatic, Randomized Optimal Platelet and Plasma Ratios randomized clinical trial	Study type/Outcome
Nistor, M., W. Behringer, M. Schmidt and R. Schiffner	2017	A Systematic Review of Neuroprotective Strategies during Hypovolemia and Hemorrhagic Shock	Population
Novak, D. J., Y. Bai, R. K. Cooke, M. B. Marques, M. J. Fontaine, J. L. Gottschall, P. M. Carey, R. M. Scanlan, E. W. Fiebig, I. A. Shulman, J. M. Nelson, S. Flax, V. Duncan, J. A. Daniel-Johnson, J. L. Callum, J. B. Holcomb, E. E. Fox, S. Baraniuk, B. C.	2015	Making thawed universal donor plasma available rapidly for massively bleeding trauma patients: experience from the Pragmatic, Randomized Optimal Platelets and Plasma Ratios (PROPPR) trial	Study type/Outcome
Oberladstatter, D., W. Voelckel, C. Schlimp, J. Zipperle, B. Ziegler, O. Grottke and H. Schochl	2021	A prospective observational study of the rapid detection of clinically-relevant plasma direct oral anticoagulant levels following acute traumatic injury	Population
Ogura, T., Y. Nakamura, M. Nakano, Y. Izawa, M. Nakamura, K. Fujizuka, M. Suzukawa and A. T. Lefor	2014	Predicting the need for massive transfusion in trauma patients: the Traumatic Bleeding Severity Score	Intervention
Olaussen, A., J. Bade-Boon, M. C. Fitzgerald and B. Mitra	2018	Management of injured patients who were Jehovah's Witnesses, where blood transfusion may not be an option: a retrospective review	Population
Otsuka, H., N. Sakoda, A. Uehata, T. Sato, K. Sakurai, H. Aoki, T. Yamagiwa, S. Iizuka and S. Inokuchi	2020	Indications for early plasma transfusion and its optimal use following trauma	Study type/Outcome
Owattanapanich, N., K. Chittawatanarat, T. Benyakorn and J. Sirikun	2018	Risks and benefits of hypotensive resuscitation in patients with traumatic hemorrhagic shock: a meta-analysis	Study type/Outcome
Ozakin, E., N. O. Yazlamaz, F. B. Kaya, E. M. Karakilic and M. Bilgin	2020	Perfusion Index Measurement in Predicting Hypovolemic Shock in Trauma Patients	Study type/Outcome
Payen, J. F., M. Berthet, C. Genty, P. Declety, D. Garrigue-Huet, N. Morel, P. Bouzat, B. Riou, J. L. Bosson and i. Novoseven Trauma	2016	Reduced mortality by meeting guideline criteria before using recombinant activated factor VII in severe trauma patients with massive bleeding	Study type/Outcome
Perel, P., T. Clayton, D. G. Altman, P. Croft, I. Douglas, H. Hemingway, A. Hingorani, K. I. Morley, R. Riley, A. Timmis, D. Van der Windt, I. Roberts and P. Partnership	2014	Red blood cell transfusion and mortality in trauma patients: risk-stratified analysis of an observational study	not relevant to any question

Study	Year	Title	Reason for exclusion
Plurad, D. S., W. Chiu, A. S. Raja, S. M. Galvagno, U. Khan, D. Y. Kim, S. A. Tisherman, J. Ward, M. E. Hamill, V. Bennett, B. Williams and B. Robinson	2018	Monitoring modalities and assessment of fluid status: A practice management guideline from the Eastern Association for the Surgery of Trauma	not relevant to any question
Pommerening, M. J., M. D. Goodman, D. L. Farley, J. C. Cardenas, J. Podbielski, N. Matijevic, C. E. Wade, J. B. Holcomb and B. A. Cotton	2014	Early diagnosis of clinically significant hyperfibrinolysis using thrombelastography velocity curves	Study type/Outcome
Presneill, J., D. Gantner, A. Nichol, C. McArthur, A. Forbes, J. Kasza, T. Trapani, L. Murray, S. Bernard, P. Cameron, G. Capellier, O. Huet, L. Newby, S. Rashford, J. V. Rosenfeld, T. Smith, M. Stephenson, D. Varma, S. Vallance, T. Walker, S. Webb, D. Ja	2018	Statistical analysis plan for the POLAR-RCT: The Prophylactic hypOthermia trial to Lessen trAumatic bRain injury-Randomised Controlled Trial	Study type/Outcome
Puranik, G. N., T. Y. P. Verma and G. A. it	2018	The Study of Coagulation Parameters in Polytrauma Patients and Their Effects on Outcome	Study type/Outcome
Rhee, P., K. Inaba, V. it, M. Khalil, S. Siboni, G. Vercruysse, N. Kulvatunyou, A. Tang, A. Asif, T. O'Keeffe and B. Joseph	2015	Early autologous fresh whole blood transfusion leads to less allogeneic transfusions and is safe	Study type/Outcome
Rijnhout, T. W. H., K. E. Wever, R. Marinus, N. Hoogerwerf, L. M. G. Geeraedts, Jr. and E. Tan	2019	Is prehospital blood transfusion effective and safe in haemorrhagic trauma patients? A systematic review and meta-analysis	Study type/Outcome
Ritchie, D. T., F. G. A. Philbrook, S. Leadbitter, K. N. Kokwe, E. Meehan, M. McGeady and M. Beaton	2020	Empirical transfusion strategies for major hemorrhage in trauma patients: A systematic review	Multiple publication without additional information
Roberts, D. J., N. Bobrovitz, D. A. Zygun, A. W. Kirkpatrick, C. G. Ball, P. D. Faris, H. T. Stelfox and G. for the Indications for Trauma Damage Control Surgery International Study	2021	Evidence for use of damage control surgery and damage control interventions in civilian trauma patients: a systematic review	Intervention
Ross, S. W., A. B. Christmas, P. E. Fischer, H. Holway, A. L. Walters, R. Seymour, M. A. Gibbs, B. T. Heniford and R. F. Sing	2015	Impact of common crystalloid solutions on resuscitation markers following Class I hemorrhage: A randomized control trial	Population
Rowell, S. E., E. N. Meier, B. McKnight, D. Kannas, S. May, K. Sheehan, E. M. Bulger, A. H. Idris, J. Christenson, L. J. Morrison, R. J. Frascone, P. L. Bosarge, M. R. Colella, J. Johannigman, B. A. Cotton, J. Callum, J. McMullan, D. J. Dries, B. Tibbs, N	2020	Effect of Out-of-Hospital Tranexamic Acid vs Placebo on 6-Month Functional Neurologic Outcomes in Patients With Moderate or Severe Traumatic Brain Injury	Population
Safari, H., P. Farrahi, S. Rasras, Mar, H. J. i and M. Zeinali	2020	Effect of Intravenous Tranexamic Acid on Intracerebral Brain Hemorrhage in Traumatic Brain Injury	Population
Safiejko, K., J. Smereka, K. J. Filipiak, A. Szarpak, M. Dabrowski, J. R. Ladny, M. J. Jaguszewski and L. Szarpak	2020	Effectiveness and safety of hypotension fluid resuscitation in traumatic hemorrhagic shock: a systematic review and meta-analysis of randomized controlled trials	Population

Study	Year	Title	Reason for exclusion
Safiejko, K., J. Smereka, M. Pruc, J. R. Ladny, M. J. Jaguszewski, K. J. Filipiak, R. Yakubtsevich and L. Szarpak	2020	Efficacy and safety of hypertonic saline solutions fluid resuscitation on hypovolemic shock: A systematic review and meta-analysis of randomized controlled trials	Population
Schindler, P., A. Helfen, M. Wildgruber, W. Heindel, C. Schulke and M. Masthoff	2019	Intraosseous contrast administration for emergency computed tomography: A case-control study	Study type/Outcome
Schoeneberg, C., D. Schmitz, S. Schoeneberg, B. Hussmann and S. Lendemans	2015	Gender-specific differences in therapy and laboratory parameters and validation of mortality predictors in severely injured patients--results of a German level 1 trauma center	Study type/Outcome
Shand, S. , K. Curtis, M. Dinh and B. Burns	2019	What is the impact of prehospital blood product administration for patients with catastrophic haemorrhage: an integrative review	Study type/Outcome
Shih, A. W., S. Al Khan, A. Y. Wang, P. Dawe, P. Y. Young, A. Greene, M. Hudoba and E. Vu	2019	Systematic reviews of scores and predictors to trigger activation of massive transfusion protocols	Study type/Outcome
Singla, A., S. Kaur, N. Kaur and C. S. Gill	2016	Arterial ammonia levels: Prognostic marker in traumatic hemorrhage	Population
Smith, J. W., P. J. Matheson, G. A. Franklin, B. G. Harbrecht, J. D. Richardson and R. N. Garrison	2017	Randomized Controlled Trial Evaluating the Efficacy of Peritoneal Resuscitation in the Management of Trauma Patients Undergoing Damage Control Surgery	Intervention
Sorgjerd, R., G. A. Sunde and J. K. Heltne	2019	Comparison of two different intraosseous access methods in a physician-staffed helicopter emergency medical service - a quality assurance study	Population
Spano, P. J., 2nd, S. Shaikh, D. Boneva, S. Hai, M. McKenney and A. Elkbuli	2020	Anticoagulant chemoprophylaxis in patients with traumatic brain injuries: A systematic review	Study type/Outcome
Stansfield, R., D. Morris and E. Jesulola	2020	The Use of Tranexamic Acid (TXA) for the Management of Hemorrhage in Trauma Patients in the Prehospital Environment: Literature Review and Descriptive Analysis of Principal Themes	Study type/Outcome
Stein, P., J. D. Studt, R. Albrecht, S. Muller, D. von Ow, S. Fischer, B. Seifert, S. Mariotti, D. R. Spahn and O. M. Theusinger	2018	The Impact of Prehospital Tranexamic Acid on Blood Coagulation in Trauma Patients	Study type/Outcome
Stettler, G. R., E. E. Moore, G. R. Nunns, Ch, J. ler, E. Peltz, C. C. Silliman, A. Banerjee and A. Sauaia	2018	Rotational thromboelastometry thresholds for patients at risk for massive transfusion	Study type/Outcome
Sunde, G. A., B. E. Heradstveit, B. H. Vikenes and J. K. Heltne	2010	Emergency intraosseous access in a helicopter emergency medical service: a retrospective study	Population
Tauber, H., N. Innerhofer, D. von Langen, M. Strohle, D. Fries, M. Mittermayr, T. Hell, E. Oswald and P. Innerhofer	2020	Dynamics of Platelet Counts in Major Trauma: The Impact of Haemostatic Resuscitation and Effects of Platelet Transfusion-A Sub-Study of the Randomized Controlled RETIC Trial	not relevant to any question
Thorn, S., R. Lefering, M. Maegele, R. L. Gruen and B. Mitra	2021	Early prediction of acute traumatic coagulopathy: a validation of the COAST score using the German Trauma Registry	Intervention
Tisherman, S. A., H. B. Alam, P. M. Rhee, T. M. Scalea, T. Drabek, R. M. Forsythe and P. M. Kochanek	2017	Development of the emergency preservation and resuscitation for cardiac arrest from trauma clinical trial	Study type/Outcome

Study	Year	Title	Reason for exclusion
Tisherman, S. A., R. H. Schmicker, K. J. Brasel, E. M. Bulger, J. D. Kerby, J. P. Minei, J. L. Powell, D. A. Reiff, S. B. Rizoli and M. A. Schreiber	2015	Detailed description of all deaths in both the shock and traumatic brain injury hypertonic saline trials of the Resuscitation Outcomes Consortium	Study type/Outcome
Tonglet, M. L., J. M. Minon, L. Seidel, J. L. Poplavsky and M. Vergnion	2014	Prehospital identification of trauma patients with early acute coagulopathy and massive bleeding: results of a prospective non-interventional clinical trial evaluating the Trauma Induced Coagulopathy Clinical Score (TICCS)	Intervention
Tracy, B. M., J. R. Dunne, C. M. O'Neal and E. Clayton	2016	Venous thromboembolism prophylaxis in neurosurgical trauma patients	Population
Tran, A., J. Yates, A. Lau, J. Lampron and M. Matar	2018	Permissive hypotension versus conventional resuscitation strategies in adult trauma patients with hemorrhagic shock: A systematic review and meta-analysis of randomized controlled trials	Multiple publication without additional information
Tran, A., M. Matar, J. Lampron, E. Steyerberg, M. Taljaard and C. Vaillancourt	2018	Early identification of patients requiring massive transfusion, embolization or hemostatic surgery for traumatic hemorrhage: A systematic review and meta-analysis	Population
Valle, E. J., C. J. Allen, R. M. Van Haren, J. M. Jouria, H. Li, A. S. Livingstone, N. Namias, C. I. Schulman and K. G. Proctor	2014	Do all trauma patients benefit from tranexamic acid?	Study type/Outcome
van den Brink, D. P., M. R. Wirtz, A. S. Neto, H. Schochl, V. Viersen, J. Binnekade and N. P. Juffermans	2020	Effectiveness of prothrombin complex concentrate for the treatment of bleeding: A systematic review and meta-analysis	Study type/Outcome
Van Haren, R. M., E. J. Valle, C. M. Thorson, J. M. Jouria, A. M. Busko, G. A. Guarch, N. Namias, A. S. Livingstone and K. G. Proctor	2014	Hypercoagulability and other risk factors in trauma intensive care unit patients with venous thromboembolism	not relevant to any question
van Turenhout, E. C., S. M. Bossers, S. A. Loer, G. F. Giannakopoulos, L. A. Schwarte and P. Schober	2020	Pre-hospital transfusion of red blood cells. Part 2: A systematic review of treatment effects on outcomes	Population
van Wessem, K. J. P. and L. P. H. Leenen	2017	Thromboelastography does not provide additional information to guide resuscitation in the severely injured	Study type/Outcome
Vassallo, J., S. Horne and J. E. Smith	2014	Intraosseous access in the military operational setting	not relevant to any question
Vasudeva, M., J. K. Mathew, M. C. Fitzgerald, Z. Cheung and B. Mitra	2020	Hypocalcaemia and traumatic coagulopathy: an observational analysis	Study type/Outcome
Veigas, P. V., J. Callum, S. Rizoli, B. Nascimento and L. T. da Luz	2016	A systematic review on the rotational thrombelastometry (ROTEM R) values for the diagnosis of coagulopathy, prediction and guidance of blood transfusion and prediction of mortality in trauma patients	Study type/Outcome
Walker, C. K., E. A. mann, T. J. Horyna and M. A. Gales	2017	Increased Enoxaparin Dosing for Venous Thromboembolism Prophylaxis in General Trauma Patients	Study type/Outcome
Warren, J., A. Moazzez, V. Chong, B. Putnam, A. Neville, G. Singer, M. Deane and D. Y. Kim	2019	Narrowed pulse pressure predicts massive transfusion and emergent operative intervention following penetrating trauma	Study type/Outcome

Study	Year	Title	Reason for exclusion
Weber, B. J., Kjell and C. B.	2012	The use of tranexamic acid for trauma patients?	Multiple publication without additional information
Whiting, P., M. Al, M. Westwood, I. C. Ramos, S. Ryder, N. Armstrong, K. Misso, J. Ross, J. Severens and J. Kleijnen	2015	Viscoelastic point-of-care testing to assist with the diagnosis, management and monitoring of haemostasis: a systematic review and cost-effectiveness analysis	Multiple publication without additional information
Winkelmann, M., J. D. Clausen, P. Graeff, C. Schroter, C. Zeckey, S. Weber-Spickschen and P. Mommsen	2019	Impact of Accidental Hypothermia on Pulmonary Complications in Multiply Injured Patients With Blunt Chest Trauma - A Matched-pair Analysis	Study type/Outcome
Wirtz, M. R., D. V. Schalkers, J. C. Goslings and N. P. Juffermans	2020	The impact of blood product ratio and procoagulant therapy on the development of thromboembolic events in severely injured hemorrhaging trauma patients	Study type/Outcome
Woernley, T. C., B. Maida, J. Melville, J. Marchena and N. Demian	2019	The Effect of Deep Vein Thrombosis Prophylaxis on Bleeding in Periorbital Surgery in Trauma Patients	Study type/Outcome
Wu, M. C., T. Y. Liao, E. M. Lee, Y. S. Chen, W. T. Hsu, M. G. Lee, P. Y. Tsou, S. C. Chen and C. C. Lee	2017	Administration of Hypertonic Solutions for Hemorrhagic Shock: A Systematic Review and Meta-analysis of Clinical Trials	Population
Yamamoto, K., A. Yamaguchi, M. Sawano, M. Matsuda, M. Anan, K. Inokuchi and S. Sugiyama	2016	Pre-emptive administration of fibrinogen concentrate contributes to improved prognosis in patients with severe trauma	Study type/Outcome
Yanamadala, V., B. P. Walcott, P. E. Fecci, P. Rozman, J. I. Kumar, B. V. Nahed and B. Swearingen	2014	Reversal of warfarin associated coagulopathy with 4-factor prothrombin complex concentrate in traumatic brain injury and intracranial hemorrhage	Population
Yang, X., Y. Chen, J. Li, L. Chen, H. Ren, Y. Liu and X. Zhang	2019	Hypertonic saline maintains coagulofibrinolytic homeostasis following moderate-to-severe traumatic brain injury by regulating monocyte phenotype via expression of lncRNAs	Population
Zeeshan, M., M. Khan, T. O'Keeffe, N. Pollack, M. Hamidi, N. Kulvatunyou, J. V. Sakran, L. Gries and B. Joseph	2018	Optimal timing of initiation of thromboprophylaxis in spine trauma managed operatively: A nationwide propensity-matched analysis of trauma quality improvement program	Population
Zehtabchi, S., S. G. Abdel Baki, L. Falzon and D. K. Nishijima	2014	Tranexamic acid for traumatic brain injury: a systematic review and meta-analysis	Population
Zhang, L. M., R. Li, X. C. Zhao, Q. Zhang and X. L. Luo	2017	Increased Transfusion of Fresh Frozen Plasma is Associated with Mortality or Worse Functional Outcomes After Severe Traumatic Brain Injury: A Retrospective Study	Study type/Outcome
Zhao, G., W. Wu, Q. M. Feng and J. Sun	2017	Evaluation of the clinical effect of small-volume resuscitation on uncontrolled hemorrhagic shock in emergency	Study type/Outcome
Ziegler, B., W. Voelckel, J. Zipperle, O. Grottke and H. Schochl	2019	Comparison between the new fully automated viscoelastic coagulation analysers TEG 6s and ROTEM Sigma in trauma patients: A prospective observational study	Study type/Outcome

Table S4. Evidence Tables

Viscoelastic assays

Study: Reference, aim, design, setting	Participants: selection criteria, characteristics	N Participants; study groups; tests evaluated	Main outcomes	Assessment: LoE, risk of bias; Conclusions
**Albert (2019)**<br>„Efficacy of Thromboelastography (TEG) in Predicting Acute Trauma-Induced Coagulopathy (ATIC) in Isolated Severe Traumatic Brain Injury (iSTBI)". *Indian J Hematol Blood Transfus* 2019; 35(2): 325-331.<br>**Study design**<br>Diagnostic cross-sectional study<br>**Aim of the study**<br>"to establish a clinically significant cut-off for TEG parameters to identify acute trauma-induced coagulopathy (ATIC) in isolated severe traumatic brain injury (TBI) patients and to validate the established TEG definition to determine whether TEG can replace traditional coagulation assays in the emergency department."<br>**Setting**<br>India, 16 months period*	**Inclusion criteria**<br>• Isolated TBI patients with GCS≤8<br>• age 16–65 years<br>• blood withdrawn for analysis <12h of injury, prior to fluid/blood transfusion<br>**Exclusion criteria**<br>• associated extracranial injuries (n=22)<br>• clinical evidence of brain death (n=3)<br>• secondary admissions (n=24)<br>**Characteristics (validation cohort)**<br><u>Age [y], median (IQR)</u><br>35 (29–42)<br><u>Male, %$</u><br>87<br><u>Mean time from injury to admission [h]</u><br>2<br>$ n/N not reported	**Participants**<br>N=58 patients in the development cohort, N=39 patients in the validation cohort<br>**Tests evaluated**<br><u>Index text</u>: TEG was performed using citrated whole blood and without any additional coagulation activator on TEM-A, automated thromboelastometer (Framar Biomedica, Rome) within 2 h of blood collection. All TEG parameters were recorded from a graphical tracing: r-time (1.8–14.2 min), κ-time (0.7–7.3 min), α-angle (27.3°–72.3°), maximum amplitude (32.1–87.9 mm; MA) and lysis at 30 min (LY30, %).<br>Hypocoagulability was defined as prolonged r & κ-time and shortened α-angle & MA. Hypercoagulability was defined as shortened r & κ-time and prolonged α-angle & MA<br><u>Reference standard</u>: conventional coagulation tests (CCT) using sodium citrate-anticoagulated blood<br>**Comparison (CCT-based definition)**<br>ATIC+: acute trauma-induced coagulopathy (N=24) (INR ≥1.27 and/or PT ≥16.7 s and/or aPTT ≥28.8 s at hospital admission)<br>ATIC–: no acute trauma-induced coagulopathy (N=15)	**TEG cut-offs for identification of ATIC in isolated severe TBI patients**<br>κ-time ≥3.7 min<br>α-angle ≤48.0°<br>**Diagnostic test performance**<br><u>true positive</u>, n=20<br><u>false positive</u>, n=12<br><u>true negative</u>, n=3<br><u>false negative</u>, n=4<br><u>Sensitivity, % (95% CI)</u><br>κ-time: 64 (45.83–79.29)<br>α-angle: 62 (44–77.31)<br>κ-time and α-angle: 63 (45.25–77.07)<br><u>Specificity, % (95% CI)</u><br>κ-time: 46 (21.27–71.99)<br>α-angle: 40 (16.82–68.73)<br>κ-time and α-angle: 43 (15.82–74.95)<br><u>PPV, % (95% CI)</u><br>κ-time: 75 (55.1–88)<br>α-angle: 75 (55.1–88)<br>κ-time and α-angle: 83 (64.15–93.32)<br><u>NPV, % (95% CI)</u><br>κ-time: 33 (43.42–72.92)<br>α-angle: 27 (10.9–51.95)<br>κ-time and α-angle:20 (7.047–45.19)	**Level of evidence**<br>2b<br>**Risk of bias (QUADAS)**<br>Patient selection: ?<br>Index test: ?<br>Reference standard: +<br>Flow and timing: +<br>**Authors' conclusion**<br>"TEG's ability to assess the spectrum of different coagulopathies in whole blood renders it to be ideal for rapidly identifying ATIC and transfusion guidance. However in our study, we observed low specificity, therefore TEG cannot replace the conventional coagulation assays for identifying ATIC but may potentially be clinically sensitive in depicting the underlying coagulopathy following brain trauma."<br>**Reviewers' conclusion**<br>The results should be interpreted with caution due to the split-sample design and small sample size. Because the

Study: Reference, aim, design, setting	Participants: selection criteria, characteristics	N Participants; study groups; tests evaluated	Main outcomes	Assessment: LoE, risk of bias; Conclusions
* study years n.r.			Diagnostic accuracy<br>κ-time: 59 (43.42–72.92)<br>α-angle: 56 (40.98–70.7)<br>κ-time and α-angle: 59 (43.42–72.92)	population was isolated severe TBI patients and multiply injured patients were excluded, the applicability to the polytraumatised population is limited.
**Baksaas-Aasen (2021)**<br>„Viscoelastic haemostatic assay augmented protocols for major trauma haemorrhage (ITACTIC): a randomized, controlled trial". *Intensive Care Med* 2021; 47: 49-59.<br>**Study design**<br>Randomised controlled trial<br>(ITACTIC trial)<br>**Aim of the study**<br>"hypothesized that VHA-augmented MHPs would improve mortality and reduce the need for massive transfusion (ten or more units of red blood cell (RBC) transfusions) in the first 24 h after injury."<br>**Setting**<br>International Trauma Research Network (Denmark, the Netherlands, Norway, Germany, UK), 2016-2018	**Inclusion criteria**<br>• clinical signs of bleeding activating the local MHP<br>• RBC transfusion initiated<br>• ≤3 h after injury<br>• ≤1 h after ED admission<br>**Exclusion criteria**<br>• none<br>**Characteristics**<br>Age [y], median (IQR)<br>VHA: 40 (26–54), N=197<br>CCT: 43 (28–59), N=194<br>Male, n/N (%)<br>VHA: 145/198 (73)<br>CCT: 159/194 (82)<br>Prior oral anticoagulation, n/N (%)<br>VHA: 12/198 (6%)<br>CCT: 15/192 (8%)<br>ISS, median (IQR)<br>VHA 26 (17–37), N=196<br>CCT: 26 (16–35), N=191<br>GCS, median (IQR)<br>VHA: 12 (3–15), N=194<br>CCT: 13 (3–15), N=191	**Participants**<br>N=411 patients randomised, 396 in ITT analysis*<br>**Study groups**<br>CCT: conventional coagulation test (CCT) in the laboratory (N=203 randomised, 195 in ITT analysis)*<br>VHA: point-of-care viscoelastic haemostatic assay (VHA) (N=208 randomised, 201 in ITT analysis)*<br>Haemostatic therapy was delivered based on results of these according to the TAC-TIC algorithms, which define triggers for additional administration of platelet, fibrinogen, plasma and antifibrinolytic therapies.<br>**Co-interventions**<br>All patients received their local hospital's standard MHP, based on the empiric delivery of tranexamic acid; blood components delivered in a 1:1:1 ratio of RBCs, plasma and platelet transfusions; and limited infusion of crystalloid fluids. In both groups, blood was drawn for coagulation analysis at baseline and after every four units of RBCs transfused, until haemostasis.	**Primary outcome**<br>Subjects alive and free of massive transfusion, 24h after injury, %; OR (95% CI)<br>CCT: 64% vs. VHA: 67%<br>OR 1.15 (0.76–1.73)<br>**Secondary outcomes (ITT population)**<br>Mortality at 6 h, n/N (%); OR (95% CI)<br>CCT: 22/195 (11) vs. VHA: 22/201 (11)<br>OR 0.97 (0.52–1.80), p=0.915<br>Mortality at 24 h, n/N (%); OR (95% CI)<br>CCT: 33/195 (17) vs. VHA: 29/201 (14)<br>OR 0.83 (0.48–1.42), p=0.495<br>Mortality at 28 days, n/N (%); OR (95% CI)<br>CCT: 55/194 (28) vs. VHA: 50/201 (25)<br>OR 0.84 (0.54–1.31), p=0.435<br>Mortality at 90 days, n/N (%); OR (95% CI)<br>CCT: 56/177 (31) vs. VHA: 53/179 (29)<br>OR 0.91 (0.58–1.42), p=0.678<br>Death from exsanguination, n/N (%); OR (95% CI)<br>CCT: 17/56 (30) vs. VHA: 13/51 (25)<br>OR 0.78 (0.34–1.82), p=0.576<br>Died before haemostasis, n/N (%); OR (95% CI)<br>CCT: 24/54 (44) vs. VHA: 19/50 (38)<br>OR 0.77 (0.35–1.67), p=0.505	**Level of evidence**<br>1b<br>**Risk of bias**<br>Selection bias: +<br>Performance bias: ?<br>Attrition bias: +<br>Detection bias: +<br>**Authors' conclusion**<br>"There was no difference in overall outcomes between VHA- and CCT-augmented-major haemorrhage protocols."<br>**Reviewers' conclusion**<br>This was a well-conducted RCT, though lack of blinding the clinical team leads to a risk of performance bias. When interpreting the study results, one needs to be aware that the study was powered to detect a 15% reduction in death/MT.

Study: Reference, aim, design, setting	Participants: selection criteria, characteristics	N Participants; study groups; tests evaluated	Main outcomes	Assessment: LoE, risk of bias; Conclusions
	<u>Systolic BP [mmHg], median (IQR)</u><br>VHA: 95 (73–120), N=178<br>CCT: 90 (74–110), N=170<br><br><u>Heart rate [beats/min], median (IQR)</u><br>VHA: 103 (87–127), N=190<br>CCT: 105 (82–123), N=181<br><br><u>With PTr>1.2, n/N (%)</u><br>VHA: 58/181 (32)<br>CCT: 44/175 (25)	* rest did not provide informed consent	<u>Massive transfusion at 24 h, n/N (%); OR (95% CI)</u><br>CCT: 55/195 (28) vs. VHA: 53/201 (26)<br>OR 0.91 (0.59–1.42), p=0.682<br><br><u>Patients with symptomatic thromboembolism, n/N (%); OR (95% CI)</u><br>CCT: 27/195 (14) vs. VHA: 17/201 (9)<br>OR 0.57 (0.31–1.08), p=0.088<br><br><u>Patients with MODS, n/N (%); OR (95% CI)</u><br>CCT: 134/159 (84) vs. VHA: 141/164 (86)<br>OR 1.14 (0.62–2.10), p=0.668<br><br><u>EQ-5D index at discharge / 28 days: median (IQR)</u><br>CCT: 49 (25-60), N=86 vs. VHA: 40 (28-60), N=92, p=0.672<br><br><u>EQ-5D index at 90 days: median (IQR)</u><br>CCT: 60 (40–70), N=75 vs. VHA: 53 (40–70), N=72, p=0.718	
**Balendran (2017)**<br>„Prothrombin time is predictive of low plasma prothrombin concentration and clinical outcome in patients with trauma hemorrhage: analyses of prospective observational cohort studies“. *Scandinavian Journal of Trauma, Resuscitation and Emergency Medicine* 2017; 25: 30.<br><br>**Study design**<br>Prognostic cross-sectional study	**Inclusion criteria ACIT (UK)**<br>• adult trauma patients (>15 y)<br>• met the local criteria for full trauma team activation<br><br>**Inclusion criteria DIA-TRE-TIC (Austria, A)**<br>• adults<br>• severe polytrauma patients (ISS≥15, injury of ≥2 body regions) <u>or</u> patients with isolated TBI (GCS≤14 after blunt head trauma, AIS<3 in any other body region)<br><br>**Exclusion criteria ACIT (UK)**<br>• ED arrival 2 h after injury<br>• administration of 2000 mL of intravenous fluid before ED arrival<br>• transfer from another hospital	**Participants**<br>N=689 patients (UK: N=358, A: N=331)<br><br>**Tests evaluated**<br>• admission prothrombin time (PT)<br>• admission EXTEM CT<br>• admission EXTEM MCF<br><br>**Comparison**<br>24h survival (N=655/689, 95.1%)	**Prognostic test performance: Prediction of 24h-mortality**<br><br><u>AUC (95% CI)</u><br>**UK:** PT: 0.90 (0.82-0.97)<br>EXTEM CT: 0.66 (0.48-0.82), p<0.001§<br>EXTEM MCF: 0.81 (0.66-0.96), p=0.04§<br><br>**A:** PT: 0.78 (0.68-0.89)<br>EXTEM CT: 0.74 (0.62-0.86), p=0.44§<br>EXTEM MCF: 0.67 (0.54-0.81), p=0.05§<br><br>§ null hypothesis: no difference in AUC between PT and EXTEM marker	**Level of evidence**<br>2b<br><br>**Risk of bias**<br>no tool available for prognostic studies<br><br>**Authors' conclusion**<br>"Our analyses suggest that prothrombin concentration at admission is predictive of mortality and transfusion and indicates that prothrombin and fibrinogen are rate limiting in coagulopathy."<br><br>**Reviewers' conclusion**

Study: Reference, aim, design, setting	Participants: selection criteria, characteristics	N Participants; study groups; tests evaluated	Main outcomes	Assessment: LoE, risk of bias; Conclusions
(data from prospective ACIT and DIA-TRE-TIC studies)<br><br>**Aim of the study**<br><br>“The aims of the study were, firstly, to investigate the consequence of admission prothrombin concentration on massive transfusion (more than 10 PRBC units) and mortality at 24 h. Secondly, to determine the relationship between admission biomarkers (PT, ROTEM EXTEM CT and MCF) and prothrombin concentration, and thirdly, to understand the ability of admission biomarkers to act as a surrogate for low prothrombin concentration and predict outcome.”<br><br>**Setting**<br><br>UK, 2008-2013 and Austria, 2005-2008	• burns covering 5% of the total body surface area<br>• taking anticoagulant medications<br>• moderate or severe liver disease<br>• known bleeding diathesis<br><br>**Exclusion criteria DIA-TRE-TIC (A)**<br><br>• patients <18 y<br>• penetrating injuries<br>• admittance >12 h after trauma<br>• pre-existing coagulopathy<br>• burn injury<br>• malignant disease<br>• avalanche victims<br>• exhibition of non-head single trauma<br><br>**Characteristics**<br><br><u>Age [y], median (IQR)</u><br>UK: 35 (23–50), A: 43 (27–56)<br><br><u>Male, n (%)</u><br>UK: 288 (80), A: 259 (78)<br><br><u>ISS, median (IQR)</u><br>UK: 13 (5–27), A: 34 (24–45)<br><br><u>SBP [mmHg], median (IQR)</u><br>UK: 132 (110–150), A: 120 (100–140)<br><br><u>GCS, median (IQR)</u><br>UK: 15 (14–15), A: 11 (6–15)			The study is limited by its inhomogeneous sample and low event rate. In addition, and measurements were not standardised.<br><br>Part of the population excluded a potentially relevant group of patients who were taking oral anticoagulant medications or with pre-existing coagulopathy.
**Barrett (2020)**<br><br>„Plasmin thrombelastography rapidly identifies trauma patients at risk for massive transfusion, mortality, and	**Inclusion criteria**<br><br>• adult patients (≥18 years old)<br>• trauma activation<br>• GCS <8 with presumed thoracic, abdominal, or pelvic injury, *or*	**Participants**<br><br>N=167 patients in total, 148 analysed<br><br>19 excluded from analysis because of incomplete TEG assays (4 rTEG, 5 citrated native TEG, 10 tPA TEG)	<u>Time to results [min], median (IQR)</u><br>P-TEG positive: 4.7 (2.5–9.1)<br>tPA TEG TMA positive: 12.7 (9.2–13.8), p<0.001<br>tPA TEG LY30: 47.1 (42.6–51.2), p<0.001<br>rTEG LY30: 54.2 (51.1–58.1), p<0.001<br><br>**Diagnostic test performance: hyperfibrinolysis**	**Level of evidence**<br><br>2b<br><br>**Risk of bias (QUADAS)**<br><br>Patient selection: ?<br><br>Index test: ?

Study: Reference, aim, design, setting	Participants: selection criteria, characteristics	N Participants; study groups; tests evaluated	Main outcomes	Assessment: LoE, risk of bias; Conclusions
hyperfibrinolysis: A diagnostic tool to resolve an international debate on tranexamic acid?" *J Trauma Acute Care Surg* 2020; 89: 991-998.<br><br>**Study design**<br>Diagnostic/prognostic cross-sectional study<br><br>**Aim of the study**<br>"to develop an assay, plasmin TEG (P-TEG), to more expeditiously stratify which trauma patients are highest risk for bleeding, hyperfibrinolysis, and death and may therefore benefit most from TXA."<br><br>**Setting**<br>USA, 2018-2019	• respiratory compromise, obstruction, and/or intubation with presumed thoracic, abdominal, or pelvic injury, *or*<br>• blunt trauma with systolic blood pressure of <90 mmHg, *or*<br>• mechanically unstable pelvic injury, *or*<br>• penetrating injuries with injury to neck and/or torso with systolic blood pressure of <90 mmHg, gunshot wound penetrating the neck/torso, or stab wounds to the neck/torso that require endotracheal intubation, *or*<br>• amputation proximal to the ankle or wrist, *or*<br>• the emergency medicine attending or chief surgical resident suspects that the patient is likely to require urgent operative intervention<br><br>**Exclusion criteria**<br>• age <18 years<br>• initial blood collected >1h post-injury<br>• infusion of blood products before the collection of blood samples<br>• patients presenting as consultations from external hospitals<br>• documented chronic liver disease (total bilirubin >2.0mg/dL) or advanced cirrhosis discovered on laparotomy<br>• known inherited defects of coagulation function (e.g., hemophilia or von Willebrand disease)<br>• patients on anticoagulants at the time of their injury<br>• subsequent downgrades from trauma activation to trauma alert or nontrauma status in the ED<br>• patients who were pregnant<br>• prisoners	**Tests evaluated**<br>Index test 1: plasmin TEG assay (P-TEG)<br><br>The P-TEG was considered positive (P-TEG positive) when the P-TEG R time is greater than or equal to native TEG R time.<br><br>Index test 2: tissue plasminogen activator challenged TEG (tPA TEG)<br><br>Reference test: TEG assays without exogenous additives (rapid/native)<br><br>**Indices obtained from TEG tracings**<br>• R time (minutes)<br>• angle (°)<br>• maximum amplitude (MA, mm)<br>• clot lysis 30 minutes after MA (LY30, %)	**reference test in brackets**<br><br><u>Sensitivity, % (95% CI)</u><br>P-TEG (rTEG LY30 >3%): 0.19 (0.08–0.37)<br>P-TEG (rTEG LY30 >7.6%): 0.31 (0.14–0.56)<br>tPA TEG TMA (rTEG LY30 >3%): 0.50 (0.31–0.69)<br>tPA TEG TMA (rTEG LY30 >7.6%): 0.77 (0.50–0.92)<br><br><u>Specificity, % (95% CI)</u><br>P-TEG (rTEG LY30 >3%): 0.88 (0.81–0.92)<br>P-TEG (rTEG LY30 >7.6%): 0.89 (0.82–0.93)<br>tPA TEG TMA (rTEG LY30 >3%): 0.87 (0.79–0.92)<br>tPA TEG TMA (rTEG LY30 >7.6%): 0.87 (0.80–0.92)<br><br><u>PPV, % (95% CI)</u><br>P-TEG (rTEG LY30 >3%): 25 (11–47)<br>P-TEG (rTEG LY30 >7.6%): 25 (11–47)<br>tPA TEG TMA (rTEG LY30 >3%): 42 (26–0.61)<br>tPA TEG TMA (rTEG LY30 >7.6%): 38 (22–57)<br><br><u>NPV, % (95% CI)</u><br>P-TEG (rTEG LY30 >3%): 83 (75–88)<br>P-TEG (rTEG LY30 >7.6%): 91 (85–95)<br>tPA TEG TMA (rTEG LY30 >3%): 90 (83–94)<br>tPA TEG TMA (rTEG LY30 >7.6%): 97 (92–99)<br><br>**Diagnostic test performance: depletion of fibrinolytic inhibitors (DFI)**<br>reference test: DFI measured by rTEG<br><br><u>Sensitivity, % (95% CI)</u><br>P-TEG: 0.32 (0.19–0.49)<br>tPA TEG TMA: 0.64 (0.47–0.78)<br><br><u>Specificity, % (95% CI)</u><br>P-TEG: 0.92 (0.86–0.96)<br>tPA TEG TMA: 0.95 (0.89–0.98)<br><br><u>PPV, % (95% CI)</u>	Reference standard: +<br><br>Flow and timing: +<br><br>no tool available for prognostic studies<br><br>**Authors' conclusion**<br>"we have developed a novel modified diagnostic assay for use on currently available commercial equipment commonly used in trauma that can, in under 5 minutes, rapidly identify trauma patients at highest risk for MT (i.e., massive bleeding), death at both 24 hours and 30 days, and high risk for hyperfibrinolysis (DFI) and, potentially, with future improvements (with or without composite use of tPATEG TMA), may be able to help guide selective TXA dosing."<br><br>**Reviewers' conclusion**<br>The time outcome may be subject to detection bias, as no mention is made of assessor blinding. The risk of bias of the index test cannot be assessed because it is unclear whether the results were interpreted without knowledge of the reference test.

Study: Reference, aim, design, setting	Participants: selection criteria, characteristics	N Participants; study groups; tests evaluated	Main outcomes	Assessment: LoE, risk of bias; Conclusions
	**Characteristics**<br><br><u>Age [y], median (range)</u><br>35 (18–90)<br><br><u>Male, n (%)</u><br>114 (77)<br><br><u>ISS, median (IQR)</u><br>20 (9–30)		P-TEG: 58 (36–77)<br>tPA TEG TMA: 81 (62–91)<br><br><u>NPV, % (95% CI)</u><br>P-TEG: 81 (73–87)<br>tPA TEG TMA: 89 (82–94)<br><br>**Prognostic test performance: prediction of patients in need of massive transfusion**<br><br><u>Sensitivity, % (95% CI)</u><br>P-TEG: 0.40 (0.20–0.64)<br>tPA TEG TMA: 0.69 (0.42–0.87)<br><br><u>Specificity, % (95% CI)</u><br>P-TEG: 0.89 (0.83–0.94)<br>tPA TEG TMA: 0.86 (0.79–0.91)<br><br><u>PPV, % (95% CI)</u><br>P-TEG: 30 (15–52)<br>tPA TEG TMA: 35 (19–54)<br><br><u>NPV, % (95% CI)</u><br>P-TEG: 93 (87–96)<br>tPA TEG TMA: 96 (91–99)<br><br>**Prognostic test performance: prediction of patients likely to benefit from TXA**<br><br><u>Sensitivity, % (95% CI)</u><br>P-TEG: 0.36 (0.16–0.61)<br>tPA TEG TMA: 0.77 (0.50–0.92)<br><br><u>Specificity, % (95% CI)</u><br>P-TEG: 0.89 (0.82–0.93)<br>tPA TEG TMA: 0.87 (0.80–0.92)<br><br><u>PPV, % (95% CI)</u><br>P-TEG: 26 (12–49)<br>tPA TEG TMA: 38 (22–57)	The effect on patient-relevant outcomes of using these tests to take therapeutic decisions was not investigated.<br><br>The population excluded a potentially relevant group of patients with bleeding disorders or who were taking oral anticoagulant medications.

Study: Reference, aim, design, setting	Participants: selection criteria, characteristics	N Participants; study groups; tests evaluated	Main outcomes	Assessment: LoE, risk of bias; Conclusions
			<u>NPV, % (95% CI)</u><br>P-TEG: 93 (87–96)<br>tPA TEG TMA: 97 (92–99)	
**Cohen (2019)**<br>"A prospective evaluation of thromboelastometry (ROTEM) to identify acute traumatic coagulopathy and predict massive transfusion in military trauma patients in Afghanistan". *Transfusion* 2019; 59: 1601-1607.<br>**Study design**<br>Prognostic cross-sectional study<br>**Aim of the study**<br>"to determine the relative capacities to identify coagulopathy and predict MT between 1) the established ATC definition using an INR cutoff of 1.2 and 2) an integrated ROTEM model that also includes EXTEM A5 35 mm or less and/or EXTEM LI30 less than 97%."<br>**Setting**<br>US military (Afghanistan), 2012-2013	**Inclusion criteria**<br>• trauma patients<br>• injuries resulting in activation of DCR (based on clinical status at presentation)<br>**Exclusion criteria**<br>n.r.<br>**Characteristics**<br><u>Age [y], median (95% CI)</u><br>26 (17–35)<br><u>Male, n (%)</u><br>40 (100)<br><u>ISS, median (95% CI)</u><br>22 (14–27)<br><u>GCS, median (95% CI)</u><br>15 (11.5–15)<br><u>SBP [mmHg], median (95% CI)</u><br>119 (110–130)<br><u>Heart rate [beats/min], median (95% CI)</u><br>129 (103–144)	**Participants**<br>N=88 patients enrolled, N=40 analysed<br>("due to intermittent lack of reagent availability, not patient selection")<br>**Tests evaluated**<br>Index test: definition of acute traumatic coagulopathy (ATC) by an integrated ROTEM model, incl. INR >1.2, EXTEM A5 ≤35 mm and/or EXTEM LI30 <97% on admission.<br>Reference test: established definition of ATC using INR >1.2<br>Blood was obtained upon admission and at 6 and 24 hours after admission by a designated research team and analyzed by ROTEM with multiple assays (EXTEM, FIBTEM, APTEM).	**Prognostic test performance: prediction of patients in need of massive transfusion**<br><u>Sensitivity: %</u><br>Index test: 86<br>Reference test: 64<br><u>Specificity: %</u><br>Index test: 38<br>Reference test: 50<br><u>Positive likelihood ratio:</u><br>Index test: 1.4<br>Reference test: 1.3<br><u>Negative likelihood ratio:</u><br>Index test: 0.4<br>Reference test: 0.7	**Level of evidence**<br>2b<br>**Risk of bias**<br>no tool available for prognostic studies<br>**Authors' conclusion**<br>"Our integrated ROTEM model of ATC demonstrated a 15% increased burden of coagulopathy above those captured by INR alone and increased the detection of those that required MT by 22%. The specificity, however, was poor, arguing for its use as an adjunct to clinical presentation in the ultimate decision to initiate MT in the combat setting."<br>**Reviewers' conclusion**<br>The cohort is small, and no measure of variance or significance is provided, so that only limited conclusions may be drawn. The effect on patient-relevant outcomes of using the index test to take therapeutic decisions was not investigated.

Study: Reference, aim, design, setting	Participants: selection criteria, characteristics	N Participants; study groups; tests evaluated	Main outcomes	Assessment: LoE, risk of bias; Conclusions
**Connelly (2017)**<br>"Assessment of three point-of-care platelet function assays in adult trauma patients". *J Surg Res* 2017; 212: 260-269.<br>**Study design**<br>Diagnostic and prognostic cross-sectional study<br>**Aim of the study**<br>"to assess the ability of each assay to identify AP medication use in trauma patients immediately on arrival. The secondary aims were to compare the ability of VerifyNow and TEG-PM with Multiplate aggregometry to identify platelet dysfunction and to determine if any of these tests were predictive of ICH progression."<br>**Setting**<br>USA, 2013-2015	**Inclusion criteria**<br>• adult trauma patients<br>• at risk for coagulopathy and hemorrhage<br>• Glasgow Coma Scale <10 or<br>• intracranial hemorrhage (ICH) on initial head CT scan or<br>• systolic blood pressure <90 mmHg or<br>• intubation or<br>• base deficit >6 mEq/L or<br>• penetrating injury to the torso, groin, or neck or<br>• amputation proximal to the ankle or wrist or<br>• uncontrolled external hemorrhage or<br>• two or more long bone fractures or<br>• pelvic fracture or<br>• combination trauma with burns (<20% total body surface area)<br>**Exclusion criteria**<br>• Children aged <15 y<br>• patients with significant burns (>20% total body surface area)<br>• prehospital cardiopulmonary resuscitation<br>• prisoners<br>• Patients on other anticoagulation medications (than anti-platelet)<br>• transferred from another facility >6 h since injury<br>**Characteristics**<br><u>Age [y], mean ± SD</u><br>AP: 35.9 (14.7)<br>non-AP: 71.6 (10.8), p<0.001<br><u>Male, n (%)</u>	**Participants**<br>N=64 patients<br>**Tests evaluated**<br>MA: Multiplate aggregometry aspirin area under the platelet aggregation curve (ASPI AUC)<br>TEG-PM: Thrombelastography Platelet Mapping percent inhibition of arachidonic acid (TEG-PM AA)<br>VN: The Verify Now Aspirin Reaction Units (ARU)<br>**Study groups**<br>Anti-platelet (AP) group: patients currently taking aspirin (ASA) or clopidogrel, verified by outpatient medication lists or medical records (N=25)<br>non-AP group: not taking ASA or clopidogrel (N=39)	**Diagnostic test performance: detection of patients on any antiplatelet therapy**<br><u>Area under ROC curve (AUC)</u><br>MA: 0.90<br>TEG-PM: 0.77<br>VN: 0.90<br><u>Cut-offs:</u><br>MA: 33.5<br>TEG-PM: 48.2<br>VN: 614<br><u>Sensitivity (%)</u><br>MA: 80<br>TEG-PM: 56<br>VN: 100<br><u>Specificity (%)</u><br>MA: 92<br>TEG-PM: 92<br>VN: 70<br><u>PPV (%)</u><br>MA: 87<br>TEG-PM: 82<br>VN: 71<br><u>NPV (%)</u><br>MA: 88<br>TEG-PM: 77<br>VN: 100<br>**Prognostic test performance: detection of patients at risk for intracranial haemorrhage progression**<br>ICH progression defined based on attending radiologist interpretation of all initial and follow-up head CT scan images.	**Level of evidence**<br>2b<br>**Risk of bias (QUADAS)**<br>Patient selection: ?<br>Index test: –<br>Reference standard: +<br>Flow and timing: ?<br>no tool available for prognostic studies<br>**Authors' conclusion**<br>"Multiplate ASPI AUC, TEG-PM AA percent inhibition, and VerifyNow ARU accurately identified AP medication use and platelet dysfunction in trauma patients. In addition, admission TEG-PM AA percent inhibition in a trauma patient may be associated with ICH progression. However, additional larger, prospective confirmatory studies are needed to confirm these findings."<br>**Reviewers' conclusion**<br>The cohort is small, and no measure of variance or significance is provided, so that only limited conclusions may be drawn. There were unexplained missing data for the

Study: Reference, aim, design, setting	Participants: selection criteria, characteristics	N Participants; study groups; tests evaluated	Main outcomes	Assessment: LoE, risk of bias; Conclusions
	AP: 31 (79)<br>non-AP: 20 (80), p=1.00<br><br><u>ISS by strata, n (%)</u><br>p=0.56<br><br>ISS 0-8: AP: 3 (8) vs. non-AP: 2 (8)<br>ISS 9-15: AP: 8 (21) vs. non-AP: 2 (8)<br>ISS 16-24: AP: 15 (38) vs. non-AP: 13 (52)<br>ISS 27-75: AP: 13 (33) vs. non-AP: 8 (32)<br><br><u>GCS</u><br>p=0.35<br><br>GCS 13-15: AP: 26 (67) vs. non-AP: 20 (80)<br>GCS 9-12: AP: 1 (3) vs. non-AP: 1 (4)<br>GCS 3-8: AP: 12 (31) vs. non-AP: 4 (16)		<u>Area under ROC curve (AUC)</u><br>MA: 0.50<br>TEG-PM: 0.66<br>VN: 0.59<br><br><u>Cut-offs:</u><br>MA: 11.0<br>TEG-PM: 31.9<br>VN: 592.5<br><br><u>Sensitivity (%)</u><br>MA: 92<br>TEG-PM: 67<br>VN: 80<br><br><u>Specificity (%)</u><br>MA: 24<br>TEG-PM: 71<br>VN: 47<br><br><u>PPV (%)</u><br>MA: 46<br>TEG-PM: 62<br>VN: 47<br><br><u>NPV (%)</u><br>MA: 80<br>TEG-PM: 75<br>VN: 80	VerifyNow test. Cutoffs for the tests were developed in this study, and are not yet validated.<br><br>The population excluded a potentially relevant group of patients who were taking oral anticoagulant medications.
**Gonzalez (2016)**<br>“Goal-directed Hemostatic Resuscitation of Trauma-induced Coagulopathy: A Pragmatic Randomized Clinical Trial Comparing a Viscoelastic Assay to Conventional Coagulation	**Inclusion criteria**<br>• ≥18 years of age<br>• met criteria for MTP activation upon ED arrival (SBP <70 mmHg or SBP 70-90 mmHg with heart rate ≥108 bpm)<br>• injured patients (penetrating torso wound, unstable pelvic fracture, or abdominal ultrasound suspicious of bleeding in more than one region)	**Participants**<br>N=111 patients<br><br>**Study groups**<br>IG: massive transfusion protocol (MTP) goal directed by point-of-care thrombelastography (TEG) (N=56)	**Primary outcome**<br><u>28 d-mortality: n/N (%); HR (95% CI)</u><br>IG: 11/56 (19.6) vs. CG: 20/55 (36.4), p=0.049<br>HR 2.17 (1.034–4.576); p=0.043<br><br>**Secondary outcomes**<br><u>6 h-mortality: n/N (%)</u>	**Level of evidence**<br>1b<br><br>**Risk of bias**<br>Selection bias: +<br>Performance bias: –<br>Attrition bias: +<br>Detection bias: +

Study: Reference, aim, design, setting	Participants: selection criteria, characteristics	N Participants; study groups; tests evaluated	Main outcomes	Assessment: LoE, risk of bias; Conclusions
Assays". *Ann Surg* 2016; 263(6): 1051-1059.<br>**Study design**<br>Pragmatic randomised controlled trial<br>**Aim of the study**<br>"to compare the effect of an MTP goal directed by TEG to a standard MTP guided by CCA on the primary outcome of survival after injury."<br>**Setting**<br>USA, 2011-2014	**Exclusion criteria**<br>• prisoners<br>• pregnant patients<br>**Characteristics**<br><u>Age [y], median (IQR)</u><br>IG: 41.0 (28–54) vs. CG: 38.0 (25–53)<br><u>Male, n (%)</u><br>IG: 37 (66.0) vs. 41 (74.5)<br><u>ISS, median (IQR)</u><br>IG: 29.5 (23–41) vs. CG: 33.0 (25–43)<br><u>GCS, median (IQR)</u><br>IG: 14.5 (6–15) vs. CG: 14.0 (3–15)<br><u>SBP [mmHg], median (IQR)</u><br>IG: 97.0 (78–120) vs. CG: 90.0 (76–110)<br><u>Heart rate [beats/min], median (IQR)</u><br>IG: 107.5 (90–123) vs. CG: 112.5 (94–134)	CG: MTP goal directed by conventional coagulation assays, i.e. INR, PTT, fibrinogen, and D-dimer (N=55)<br>Platelet counts were available to both groups as part of the complete blood cell count. Both groups had all tests performed (INR, PTT, fibrinogen, D-dimer, and TEG); however, managing clinicians only had access to the test(s) assigned to the study group and were blinded to the other tests.<br>**Co-interventions**<br>The amounts of administered crystalloid and RBC units at 2, 4, 6, 12, and 24 hours from time of injury were similar between the 2 groups.<br><u>RBC units during initial 2 h of resuscitation</u><br>IG: 4.5 (2–8), CG: 5.0 (2–11), p=0.317<br><u>plasma units during initial 2 h</u><br>IG: 0.0 (0–3), CG: 2.0 (0–4), p=0.022<br><u>platelets units during initial 2 h</u><br>IG: 0.0 (0–0), CG: 0.0 (0–1), p=0.041<br><u>cryoprecipitate during initial 24h</u><br>IG: 0.0 (0–2), CG: 1.0 (0–2), p=0.040	IG: 4/56 (7.1) vs. CG: 12/55 (21.8), p=0.032<br><u>Time to death [h] from ED arrival, median (IQR)</u><br>IG: 10.4 (4.5–200.3) vs. CG: 4.2 (2.4–9.9), p=0.181<br><u>ICU-free time [d], value (95% CI)</u><br>IG: 16 (0–22) vs. CG: 8.5 (0–19.5), p=0.091	**Authors' conclusion**<br>"This trial demonstrates that a goal-directed, TEG-guided MTP improves survival after injury and promotes appropriate use of hemostatic blood products while favourably impacting ICU stay and mechanical ventilation time."<br>**Reviewers' conclusion**<br>This is a pragmatic RCT limited by the constraints in the context of trauma care. Lack of blinding leads to a risk of performance bias, which needs to be accounted for when interpreting the results.
**Hagemo (2015)**<br>"Detection of acute traumatic coagulopathy and massive transfusion requirements by means of rotational thromboelastometry: an international prospective validation study". *Critical Care* 2015; 19: 97.	**Inclusion criteria**<br>• patients ≥18 years<br>• requiring full trauma team activation<br>**Exclusion criteria**<br>• patients who received ≥2,000 mL of fluids before arrival<br>• who arrived in the ED ≥2h from time of injury<br>• patients who were pregnant	**Participants**<br>N=808 patients<br>**Tests evaluated**<br>EXTEM: the citrated sample is recalcified before it is activated by tissue factor (TF)<br>FIBTEM: the platelet inhibitor cytochalasin D was added for platelet inhibition, to isolate the fibrin component of the clot.	**Diagnostic test performance: acute traumatic coagulopathy (ATC)**<br>Reference standard: laboratory INR >1.2<br><u>AUC value (95% CI)</u><br>EXTEM CA5: 0.79 (0.76-0.81)<br>FIBTEM CA5: 0.80 (0.77-0.83)<br>Fibrinogen: 0.87* (0.84-0.89)<br>Platelet count: 0.74 (0.70-0.77)<br>analysis using new "optimum" thresholds, i.e.	**Level of evidence**<br>2b<br>**Risk of bias (QUADAS)**<br>Patient selection: ?<br>Index test: –<br>Reference standard: +<br>Flow and timing: +

Study: Reference, aim, design, setting	Participants: selection criteria, characteristics	N Participants; study groups; tests evaluated	Main outcomes	Assessment: LoE, risk of bias; Conclusions
**Study design**<br>Diagnostic/prognostic cross-sectional study<br>(part of the Activation of Coagulation and Inflammation in Trauma (ACIT) 3 study)<br>**Aim of the study**<br>“to identify the threshold values that most accurately identify ATC and the need for massive transfusion, using the EXTEM assay, as well as the platelet-inhibited FIBTEM assay.”<br>**Setting**<br>UK, Denmark, Norway, 2007-2011	• known liver failure<br>• bleeding disorders<br>• taking oral anticoagulant medications other than acetylsalicylic acid<br>**Characteristics**<br>Age [y], median (IQR)<br>38 (28)<br>Male, %[$]<br>77.4<br>ISS, median (IQR)<br>16 (20)<br>INR <1.2, n (%)<br>89 (11.0)<br>[$] n/N not reported	CCT: conventional coagulation tests, incl. PT converted to international normalized ratio (INR), fibrinogen concentration and platelet count.<br>Blood samples were collected within 20 minutes of arrival in hospital. ROTEM assays were performed within one hour by dedicated study personnel using the ROTEM Delta.	EXTEM CA5 ≤37 (34-39) mm<br>FIBTEM CA5 ≤8 (5-8) mm<br>Fibrinogen concentration ≤1.61 (1.36-1.9) g/L<br>Platelet count ≤199 (128-199) x 10^9/L<br>Sensitivity (detection rate), % (95% CI)<br>EXTEM CA5 ≤37 mm: 66.3 (55.1-76.3)<br>FIBTEM CA5 ≤8 mm: 67.5 (55.9-77.8)<br>Fibrinogen ≤1.61 g/L: 73.6 (63.0-82.4)<br>Platelet count ≤199 x 10^9/L: 61.7 (46.4-75.5)<br>False positive rate, % (95% CI)<br>EXTEM CA5 ≤40mm: 18.8 (15.9-21.9)<br>FIBTEM CA5 ≤8 mm: 20.7 (17.7-23.9)<br>Fibrinogen ≤1.61 g/L: 11.5 (9.2-14.1)<br>Platelet count ≤199 x 10^9/L: 29.9 (26.6-33.4)<br>PPV, % (95% CI)<br>EXTEM CA5 ≤40mm: 29.9 (23.4-37.1)<br>FIBTEM CA5 ≤8 mm: 26.9 (20.8-33.8)<br>Fibrinogen ≤1.61 g/L: 45.1 (36.7-53.6)<br>Platelet count ≤199 x 10^9/L: 11.9 (8.1-16.7)<br>NPV, % (95% CI)<br>EXTEM CA5 ≤40mm: 95.2 (93.2-96.8)<br>FIBTEM CA5 ≤8 mm: 95.6 (93.5-97.1)<br>Fibrinogen ≤1.61 g/L: 96.3 (94.5-97.7)<br>Platelet count ≤199 x 10^9/L: 96.5 (94.6-97.9)<br>**Prognostic test performance: prediction of patients in need of massive transfusion**<br>analysis using previous thresholds<br>Sensitivity (detection rate), % (95% CI)<br>INR >1.2: 51.1 (36.1-65.9)<br>CT >94s: 28.9 (16.4-44.3)<br>EXTEM CA5 <35 mm: 45.5 (30.4-61.2)<br>α-angle <65°: 37.2 (23.0-53.3)<br>False positive rate, % (95% CI)	no tool available for prognostic studies<br>**Authors’ conclusion**<br>“this study confirms the previous finding that the ROTEM CA5 value measured on arrival is a valid marker for ATC and predicts MT requirements. An EXTEM CA5 threshold value of ≤40 mm has a detection rate of 72.7%, whereas a FIBTEM CA5 threshold value of ≤9 mm detects MT requirements in 77.5% of cases. Fibrinogen concentration was significantly better than ROTEM assays in predicting ATC, and a fibrinogen concentration ≤1.90 g/L had a detection rate of 77.8% for MT requirement.”<br>**Reviewers’ conclusion**<br>Patients were non-consecutively recruited, which may lead to selection bias. Cutoffs for the tests were developed in this study, and are not yet validated.<br>The population excluded a potentially relevant group of patients with bleeding disorders or who were taking oral anticoagulant medications.

Study: Reference, aim, design, setting	Participants: selection criteria, characteristics	N Participants; study groups; tests evaluated	Main outcomes	Assessment: LoE, risk of bias; Conclusions
			INR >1.2: 8.8 (6.8-11.0)<br>CT >94s: 8.8 (6.9-11.2)<br>EXTEM CA5 <35 mm: 16.1 (13.5-19.0)<br>α-angle <65°: 12.2 (9.9-14.8)<br><br><u>PPV, % (95% CI)</u><br>INR >1.2: 27.3 (18.3-37.9)<br>CT >94s: 16.5 (9.1-26.5)<br>EXTEM CA5 <35 mm: 14.4 (9.0-21.3)<br>α-angle <65°: 15.1 (8.9-23.4)<br><br><u>NPV, % (95% CI)</u><br>INR >1.2: 96.7 (95.0-97.9)<br>CT >94s: 95.5 (93.7-96.9)<br>EXTEM CA5 <35 mm: 96.3 (94.5-97.6)<br>α-angle <65°: 96.0 (94.2-97.3)<br><br>**ROC analyses, prediction of massive transfusion**<br><br><u>AUC value (95% CI)</u><br>EXTEM CA5: 0.75 (0.72-0.78)<br>FIBTEM CA5: 0.78 (0.74-0.81)<br>Fibrinogen: 0.81 (0.78-0.83)<br>INR: 0.82 (0.79-0.84)<br>Platelet count: 0.70 (0.66-0.73)<br><br>analysis using new “optimum” thresholds:<br><br>EXTEM CA5 ≤40 (32-40) mm<br>FIBTEM CA5 ≤9 (6-9) mm<br>Fibrinogen concentration ≤1.90 (1.39-2.18) g/L<br>INR ≥1.13 (1.0-1.16)<br>Platelet count ≤174 (159-182) x 10^9/L<br><br><u>Sensitivity (detection rate), % (95% CI)</u><br>EXTEM CA5 ≤40 mm: 72.7 (57.2-85.0)<br>FIBTEM CA5 ≤9 mm: 77.5 (61.5-89.2)<br>Fibrinogen ≤1.90 g/L: 77.8 (62.9-88.8)<br>INR ≥1.13: 70.2 (55.1-82.7)<br>Platelet count ≤174 x 10^9/L: 52.8 (41.9-63.5)<br><br><u>False positive rate, % (95% CI)</u>	

Study: Reference, aim, design, setting	Participants: selection criteria, characteristics	N Participants; study groups; tests evaluated	Main outcomes	Assessment: LoE, risk of bias; Conclusions
			EXTEM CA5 ≤40 mm: 31.3 (28.0-34.8)<br>FIBTEM CA5 ≤9 mm: 32.8 (29.4-36.4)<br>Fibrinogen ≤1.90 g/L: 29.7 (26.4-30.1)<br>INR ≥1.13: 19.0 (16.2-22.1)<br>Platelet count ≤174 x 10^9/L: 14.8 (12.2-17.7)<br><br><u>PPV, % (95% CI)</u><br>EXTEM CA5 ≤40 mm: 12.2 (8.5-16.8)<br>FIBTEM CA5 ≤9 mm: 11.4 (7.9-15.8)<br>Fibrinogen ≤1.90 g/L: 14.0 (9.9-18.9)<br>INR ≥1.13: 19.2 (13.6-25.9)<br>Platelet count ≤174 x 10^9/L: 32.2 (24.7-40.4)<br><br><u>NPV, % (95% CI)</u><br>EXTEM CA5 ≤40 mm: 97.7 (96.0-98.8)<br>FIBTEM CA5 ≤9 mm: 98.2 (96.6-99.2)<br>Fibrinogen ≤1.90 g/L: 98.1 (96.5-99.1)<br>INR ≥1.13: 97.7 (96.2-98.7)<br>Platelet count ≤174 x 10^9/L: 93.1 (90.8-95.0)<br><br>[*] AUC is significantly larger than the AUC of the ROTEM parameters (p=0.002 for difference to FIBTEM CA5).	
**Moore (2017)**<br>"Viscoelastic Tissue Plasminogen Activator Challenge Predicts Massive Transfusion in 15 Minutes". *J Am Coll Surg* 2017; 225(1): 138-147.<br>**Study design**<br>Prognostic cross-sectional study<br>**Aim of the study**<br>"We hypothesize that a modified thrombelastogram (tPA-TEG) with the addition of	**Inclusion criteria**<br>• Consecutive adult trauma patients<br>• meeting criteria for the highest level of activation<br>**Exclusion criteria**<br>• n.r.<br>**Characteristics**<br><u>Age [y], median (IQR?)</u>[*]<br>33 (26–48)<br><u>Male, %</u>[$]<br>81<br><u>ISS, median (IQR?)</u>[*]	**Participants**<br>N=324 patients<br>**Study groups**<br>R-TEG: rapid thrombelastogram (TEG) Reaction time (R-time min.), angle (°), maximum amplitude (MA [mm]), time to MA (TMA min.) and lysis 30 min after MA (LY30 [%]).<br>Lt-TEG: r-TEG assay with low dose of tissue plasminogen activator (tPA)<br>Ht-TEG: TEG assay with high dose of tPA<br>INR: international normalized ratio	<u>Time to result [min], median (IQR)</u><br>Lt-LY30 >27%: 50 (47–53)<br>Lt-TMA <23min: 19 (16–21)<br>Ht-LY30 >71%: 42 (40–44)<br>R-TEG MA <57mm: 23 (20–28)<br>**Prognostic test performance: prediction of massive transfusion**<br>Massive transfusion defined as >4 units of RBC per h or death attributed to hemorrhagic shock during the initial 6 h postinjury<br><u>AUC (95% CI)</u><br>Lt-LY30: 0.86 (0.79–0.93)<br>Lt-TMA: 0.79 (0.71–0.87)<br>Ht-LY30: 0.84 (0.77–0.91)<br>Ht-TMA: 0.78 (0.71 – 8.5)	**Level of evidence**<br>2b<br>**Risk of bias**<br>no tool available for prognostic studies<br>**Authors' conclusion**<br>"Using a high dose tPA TEG to help guide the activation of the massive transfusion protocol provides actionable results within 16 minutes. Combining this assay with an INR can raise its positive predictive value from 36% to 49% while

Study: Reference, aim, design, setting	Participants: selection criteria, characteristics	N Participants; study groups; tests evaluated	Main outcomes	Assessment: LoE, risk of bias; Conclusions
exogenous tPA (tPA-TEG) predicts the patients' risk for requiring a MT more efficiently than current scoring systems."<br>**Setting**<br>USA, 2014-2016	16 (5–27)<br>GCS, median (IQR?)*<br>15 (7–15)<br>Systolic blood pressure [mmHg], median (IQR?)*<br>112 (90–138)<br>Heart rate, median [beats/min] (IQR?)*<br>102 (82–118)<br><br>* measure of variance unclear<br>$ n/N not reported	SI: shock index = heart rate/systolic blood pressure<br>ABC: assessment of blood consumption score<br>TASH: trauma associated severe hemorrhage score	R-TEG MA: 0.79 (0.73–0.85)<br>INR: 0.86 (0.81–0.91)<br>TASH: 0.84 (0.79–0.90)<br>SI: 0.70 (0.61–0.80)<br>ABC: 0.66 (0.58–0.74)<br>Thresholds by Youden index<br>Lt-LY30: 27%<br>Lt-TMA: 23 min<br>Ht-LY30: 71%<br>Ht-TMA: 16 min<br>R-TEG MA: 57mm<br>INR: 1.1<br>TASH: 8<br>SI: 1.07<br>ABC: 1<br>Sensitivity, % (95% CI)<br>Lt-LY30: 84<br>Lt-TMA: 67<br>Ht-LY30: 80<br>Ht-TMA: 84<br>R-TEG MA: 68<br>INR: 90<br>TASH: 87<br>SI: 63<br>ABC: 85<br>Specificity, % (95% CI)<br>Lt-LY30: 82<br>Lt-TMA: 85<br>Ht-LY30: 84<br>Ht-TMA: 70<br>R-TEG MA: 79<br>INR: 69<br>TASH: 76<br>SI: 77<br>ABC: 37<br>PPV, % (95% CI)	excluding 97% of patients that did not require a massive transfusion."<br>**Reviewers' conclusion**<br>A non-standard definition for massive transfusion is used, limiting comparability with other studies. Cutoffs for the tests were developed in this study, and are not yet validated. No measure of variance or significance is provided, so that only limited conclusions may be drawn. The effect on patient-relevant outcomes of using these tests to take therapeutic decisions was not investigated.

Study: Reference, aim, design, setting	Participants: selection criteria, characteristics	N Participants; study groups; tests evaluated	Main outcomes	Assessment: LoE, risk of bias; Conclusions
			Lt-LY30: 50<br>Lt-TMA: 45<br>Ht-LY30: 50<br>Ht-TMA: 36<br>R-TEG MA:41<br>INR: 33<br>TASH: 38<br>SI: 37<br>ABC: 22<br><br><u>NPV, % (95% CI)</u><br>Lt-LY30: 96<br>Lt-TMA: 92<br>Ht-LY30: 95<br>Ht-TMA: 94<br>R-TEG MA:92<br>INR: 97<br>TASH: 97<br>SI: 92<br>ABC: 92	
**Peng (2019)**<br>“A comparative study of viscoelastic hemostatic assays and conventional coagulation tests in trauma patients receiving fibrinogen concentrate”. *Clinica Chimica Acta* 2019; 495: 253-262.<br><br>**Study design**<br>Diagnostic cross-sectional study<br><br>(substudy of the FiiRST RCT)<br><br>**Aim of the study**<br>“we conducted a comparative study of	**Inclusion criteria**<br>• adults (age >18 y)<br>• severe trauma patients<br>• identified as being at risk for significant haemorrhage by hypotension (SBP ≤100 mmHg) and need for uncrossmatched RBC transfusion ≤30 min of arrival<br><br>**Exclusion criteria**<br>• received any blood or blood products before admission<br>• presented >6 h after injury<br>• estimated body weight < 50 kg<br>• known or suspected pregnancy<br>• catastrophic brain injury (any of: GCS of 3 as a result of brain injury; need of immediate neurosurgery, focal signs such as anisocoria or imaging evidence	**Participants**<br>N=45 patients<br><br>**Tests evaluated**<br>TEG FF MA: maximum amplitude or maximum clot strength (MA) determined by standard functional fibrinogen (FF) thrombelastography (TEG) test<br><br>TEG FIBTEM MA: MA determined by a crossover test using ROTEM reagents on TEG with the same reagent:blood ratio as the ROTEM FIBTEM test<br><br>ROTEM FIBTEM MCF: rotational thromboelastometry (ROTEM) FIBTEM was performed using 300 μL of citrated whole blood and 20 μL of ex-tem together with 20 μL of fib-tem following the procedure as recommended by the company.	**Diagnosis of hypofibrinogenemia**<br>Reference test: 48h fibrinogen <1 g/L<br><br><u>AUC (95% CI)</u><br>TEG FF MA: 0.948 (0.886–1.000), p=0.002<br><br>ROTEM FIBTEM MCF: 0.962 (0.900–1.000), p<0.001<br><br>TEG FIBTEM MA: 0.945 (0.893–0.997), p<0.001<br><br>ROTEM EXTEM MCF: 0.920 (0.833–1.000), p<0.001<br><br>**Diagnosis of coagulopathy**<br>Reference test: 48h INR ≥1.2<br><br><u>AUC (95% CI)</u><br>TEG FF MA: 0.557 (0.480–0.634), p=0.15	**Level of evidence**<br>3b↓<br><br>**Risk of bias (QUADAS)**<br>Patient selection: ?<br><br>Index test: –<br><br>Reference standard: +<br><br>Flow and timing: +<br><br>**Authors’ conclusion**<br>“all TEG MA and ROTEM MCF predicted hypofibrinogenemia with high accuracies. For the diagnosis of coagulopathy, only EXTEM MCF

Study: Reference, aim, design, setting	Participants: selection criteria, characteristics	N Participants; study groups; tests evaluated	Main outcomes	Assessment: LoE, risk of bias; Conclusions
functional fibrinogen and coagulation assays using TEG and ROTEM and CCTs to determine their capability to monitor coagulation profiles, diagnose coagulopathy and predict blood transfusion requirements in trauma patients."<br><br>**Setting**<br><br>Canada, 2014-2015	of intracranial bleeding with mass effect, transcranial gunshot wound, or open skull fracture with exposure/loss of brain tissue)<br>• non-haemorrhagic shock (i.e. obstructive [cardiac tamponade, tension pneumothorax and massive pulmonary emboli], neurogenic, cardiogenic, or septic)<br>• underlying hereditary or acquired coagulopathy<br>• known or suspected use of anticoagulant medications such as warfarin, low-molecular weight heparin, and direct thrombin and factor Xa inhibitors<br>• moribund and predicted to expire in a few h.<br><br>**Characteristics**<br><br><u>Age [y], median (range)</u><br>FC: 48 (19–78) vs. placebo: 28 (19–88)<br><br><u>Male, %</u>[$]<br>FC: 77 vs. placebo: 87<br><br><u>ISS, median (IQR)</u><br>FC: 25 (19–29) vs. placebo: 23 (18–29)<br><br><u>GCS, median (IQR)</u><br>FC: 15 (14–15) vs. placebo: 15 (12–15)<br><br><u>Systolic blood pressure [mmHg], median (IQR)</u><br>FC: 106 (80–144) vs. placebo: 99 (82–99)<br><br><u>INR, mean (SD)</u><br>FC: 1.2 (0.3) vs. placebo: 1.1 (0.2)	ROTEM EXTEM MCF: ROTEM EXTEM was conducted in parallel using 300 µL of the same blood sample and 20 µL of start-tem together with 20 µL of ex-tem.<br><br>Reference tests: conventional coagulation tests (CCTs) including 48h fibrinogen (threshold <1 g/L) and 48h INR (threshold ≥1.2)<br><br>TEG FF/FIBTEM, ROTEM FIBTEM/EXTEM tests and CCTs were simultaneously performed during hospital admission and 48-h hospitalization.<br><br>**Co-interventions**<br><br>N=21 allocated to fibrinogen concentrate (FC), N=24 allocated to placebo	ROTEM FIBTEM MCF: 0.564 (0.488–0.640), p=0.10<br><br>TEG FIBTEM MA: 0.533 (0.455–0.611), p=0.41<br><br>ROTEM EXTEM MCF: 0.609 (0.535–0.683), p=0.005	performed reasonably well"<br><br>**Reviewers' conclusion**<br><br>The population excluded a potentially relevant group of patients who were taking oral anticoagulant medications or with underlying hereditary or acquired coagulopathy.<br><br>Only AUC values were evaluated, and no cut-offs were derived, which limits the applicability of the study results. It is unclear whether text results were interpreted without knowledge of the reference standard.

Study: Reference, aim, design, setting	Participants: selection criteria, characteristics	N Participants; study groups; tests evaluated	Main outcomes	Assessment: LoE, risk of bias; Conclusions
	$ n/N not reported			
**Rizoli (2016)**<br>"In Trauma, Conventional ROTEM and TEG Results Are Not Interchangeable But Are Similar in Clinical Applicability". *Military Medicine* 2016; 181(5): 117.<br>**Study design**<br>Diagnostic and prognostic cross-sectional study<br>**Aim of the study**<br>"a study on the interchangeability of the conventionally performed TEG and ROTEM. We also investigated whether one test would be superior to the other in predicting mortality, the need for blood transfusion, and diagnosing early trauma coagulopathy."<br>**Setting**<br>Canada, 2012-2013	**Inclusion criteria**<br>• adults (age >16 y)<br>• severely injured (ISS >15)<br>• patients admitted directly from the scene ≤1 h of the trauma<br>• significant bleeding (expected to receive massive transfusion based on the ABC score ≥2 for massive transfusion) and probable coagulopathy (INR ≥1.2 and/or fibrinogen <1 g/L)<br>**Exclusion criteria**<br>• known acquired coagulopathy<br>• not received directly from the injury scene<br>• ≤15 years or ≤50 kg if age unknown<br>• pregnancy<br>**Characteristics**<br><u>Age [y], mean ± SD</u><br>40.2 ± 20.1<br><u>Male, n (%)</u><br>26 (78.79)<br><u>ISS, mean ± SD</u><br>23.5 ± 14.0<br><u>Admission Systolic blood pressure [mmHg], mean ± SD</u><br>123.4 ± 26.5<br><u>Admission heart rate [bpm], mean ± SD</u><br>99.6 ± 20.9<br><u>INR, mean ± SD</u>	**Participants**<br>N=33 patients<br>**Study groups**<br>TEG MA: thrombelastography (TEG) maximum amplitude (MA) on a TEG 5000 Analyzer (Haemoscope, Niles, Illinois) using kaolin activation<br>EXTEM MCF: maximum clot firmness (MCF) by EXTEM assay on a ROTEM delta system (TEM Systems, Durham, North Carolina) using tissue factor (conventional)<br>FIBTEM MCF: maximum clot firmness (MCF) by FIBTEM assay on a ROTEM delta system (TEM Systems, Durham, North Carolina) using added cytochalasin D as a platelet inhibitor<br>Reference tests: International normalized ratio (INR) ≥1.2 or fibrinogen <1 g/L defined coagulopathy.<br>ROTEM and TEG were performed simultaneously in the same patients within 30 minutes of admission and repeated when clinically indicated during the first 12 hours.<br>The results of the VHA were not available to the clinicians and none of the clinical decisions made were based on the ROTEM or TEG results.	**Diagnosis of hypofibrinogenemia**<br>Reference test: fibrinogen <1 g/L<br><u>AUC (95% CI)</u>$<br>TEG MA: 0.743 (0.530–0.956)<br>EXTEM MCF: 0.549 (0.285–0.812), p=0.09<br>FIBTEM MCF: 0.558 (0.348–0.769), p=0.12<br>**Diagnosis of coagulopathy**<br>Reference test: INR ≥1.2<br><u>AUC (95% CI)</u>$<br>TEG MA: 0.595 (0.452–0.738)<br>EXTEM MCF: 0.566 (0.422–0.709), p=0.63<br>FIBTEM MCF: 0.595 (0.452–0.738), p=0.76<br>**Prediction of mortality**<br><u>AUC (95% CI)</u>$<br>TEG MA: 0.709 (0.563–0.855)<br>EXTEM MCF: 0.743 (0.607–0.880), p=0.59<br>FIBTEM MCF: 0.755 (0.563–0.947), p=0.59<br>**Prediction of massive RBC transfusion**<br>replacement of ≥10 units of RBCs within 24 h<br><u>AUC (95% CI)</u>$<br>TEG MA: 0.812 (0.706–0.918)<br>EXTEM MCF: 0.830 (0.734–0.927), p=0.73<br>FIBTEM MCF: 0.783 (0.646–0.919), p=0.0001<br>$ p-values compared to TEG MA	**Level of evidence**<br>3b↓<br>**Risk of bias (QUADAS)**<br>Patient selection: ?<br>Index test: –<br>Reference standard: +<br>Flow and timing: +<br>no tool available for prognostic studies<br>**Authors' conclusion**<br>"The results from TEG and ROTEM, when conventionally performed, failed to reach acceptable limits of agreement and thus are not interchangeable. (…) Although the results are not interchangeable, both VHA appear to have a similar clinical performance in predicting mortality, the need for blood transfusion, and diagnosing early trauma coagulopathy."<br>**Reviewers' conclusion**<br>Only AUC values were evaluated, and no cut-offs were derived, which limits the applicability of the study results. It is unclear whether text results were interpreted without

Study: Reference, aim, design, setting	Participants: selection criteria, characteristics	N Participants; study groups; tests evaluated	Main outcomes	Assessment: LoE, risk of bias; Conclusions
	1.33 ± 0.4			knowledge of the reference standard.<br><br>The population excluded a potentially relevant group of patients with acquired coagulopathy.
**Spagnolello (2020)**<br><br>„Introduction of a ROTEM protocol for the management of trauma-induced coagulopathy". *Trauma* 2020; 1-14.<br><br>**Study design**<br><br>Prognostic cross-sectional study<br><br>**Aim of the study**<br><br>"The aims of this study were (a) to compare time to results for ROTEM testing versus laboratory conventional coagulation testing (CCT) and (b) to compare incidence of Trauma-induced coagulopathy (TIC) for our 5 and 10 minute ROTEM algorithms versus both the CCT-based European guideline algorithm and the ROTEM-based iTACTIC study algorithm, in both MT and non-MT patients."<br><br>**Setting**<br><br>UK, 2016-2019	**Inclusion criteria**<br><br>• adult patient (≥16 y)<br>• major trauma patients thought to be bleeding<br>• underwent ROTEM testing<br><br>**Exclusion criteria**<br><br>• Patients on anticoagulant medication (local protocol suggests point-of-care INR testing followed by immediate reversal using Prothrombin Complex Concentrate (PCC) prior to ROTEM testing)<br><br>**Characteristics**<br><br><u>Age [y], mean ± SD</u><br><br>IG: 47.4 ± 19.3<br><br><u>Male, n (%)</u><br><br>44 (77.2)<br><br><u>ISS, median (IQR)</u><br><br>25.0 (16.0–30.0)<br><br><u>Ssystolic blood pressure [mmHg], median (IQR)</u><br><br>110.0 (90.0–120.00)<br><br><u>TICCS, median (IQR)</u><br><br>7.0 (4.0–9.0)	**Participants**<br><br>N=57 patients<br><br>**Study groups**<br><br>EXTEM A5 <35mm: clot firmness by rotational thromboelastometry at 5 minutes <35mm<br><br>iTACTIC: the implementation treatment algorithms for the correction of trauma-induced coagulopathy (iTACTIC) ROTEM algorithm; for thresholds, see Hagemo (2015)<br><br>RIE A5: Edinburgh ROTEM algorithm using only clot firmness 5 min. after the clot is first detected<br><br>RIE A10: Edinburgh ROTEM algorithm using only clot firmness 10 min. after the clot is first detected<br><br>CCT: conventional coagulation tests with INR >1.5; fibrinogen concentration ≤1.5 g/l; platelet count ≤50 or platelet count ≤100.<br><br>sample for ROTEM testing drawn at the same time as the CCT<br><br>**Full Edinburgh algorithm (considers three aspects of haemostasis)**<br><br>• 1. clot firmness or strength (assessed from the A10 or A5, the clot firmness 10 or 5 mins after the clot is first detected) which is reduced by a low platelet count,	<u>Time from ED admission to result [min], median (IQR)</u><br><br>CCT: 83 (60-93)<br>ROTEM A5: 51 (32–93), p=0.0006 vs. CCT<br>ROTEM A10: 56 (37-98)<br><br>**Prognostic test accuracy: prediction of massive transfusion**<br><br><u>Sensitivity: % (95% CI)</u><br><br>EXTEM A5 <35mm: 54.5 (23.3–83.2)<br>iTACTIC: 100.0 (71.5–100.0)<br>RIE A5: 36.3 (10.9–69.2)<br>RIE A10: 45.4 (16.7–76.6)<br>CCT: 54.5 (23.3–83.2)<br><br><u>Specificity: % (95% CI)</u><br><br>EXTEM A5 <35mm: 65.2 (49.7–78.6)<br>iTACTIC: 23.9 (12.6–38.7)<br>RIE A5: 93.4 (82.1–98.6)<br>RIE A10: 93.5 (82.1–98.6)<br>CCT: 82.6 (68.6–92.2)<br><br><u>PPV: % (95% CI)</u><br><br>EXTEM A5 <35mm: 27.2 (16.1–42.3)<br>iTACTIC: 23.9 (21.1–27.0)<br>RIE A5: 57.1 (25.8–83.6)<br>RIE A10: 62.5 (31.8–85.6)<br>CCT: 42.8 (24.6–63.2)<br><br><u>NPV: % (95% CI)</u><br><br>EXTEM A5 <35mm: 85.7 (75.2–92.2)<br>iTACTIC: 100.0 (71.5–100.0)	**Level of evidence**<br><br>2b<br><br>**Risk of bias**<br><br>no tool available for prognostic studies<br><br>**Authors' conclusion**<br><br>"In summary, we have developed and validated a simplified ROTEM algorithm for the management of trauma patients using 5 and 10 minute EXTEM and FIBTEM thresholds which enables coagulation assessment to be obtained faster than laboratory CCT, identifies more patients with TIC, recommends fewer blood component transfusions to patients with a lesser degree of coagulation abnormality and predicts the need for MT better than other existing ROTEM algorithms."<br><br>**Reviewers' conclusion**<br><br>It is unclear whether the sample was consecutive

Study: Reference, aim, design, setting	Participants: selection criteria, characteristics	N Participants; study groups; tests evaluated	Main outcomes	Assessment: LoE, risk of bias; Conclusions
	<u>Time from admission to sampling [min], median (IQR)</u><br>20 (11-85)	a low plasma fibrinogen concentration and impaired fibrin polymerisation.<br>• 2. the time taken until clot is first detected (assessed from the clotting time; CT) which is prolonged by a low fibrinogen concentration, low concentrations of other coagulation factors, anticoagulants and by thrombocytopenia (because in a whole blood test such as ROTEM, coagulation factors act on the surface of platelets).<br>• 3. whether there is excessive clot lysis.	RIE A5: 86.0 (79.6–90.6)<br>RIE A10: 87.7 (80.6–92.5)<br>CCT: 88.3 (79.7–93.6)<br><u>Test accuracy (ACC): % (95% CI)</u><br>EXTEM A5 <35mm: 63.1 (49.3–75.5)<br>iTACTIC: 38.6 (26.0–52.4)<br>RIE A5: 82.4 (70.0–91.2)<br>RIE A10: 84.2 (72.1–92.5)<br>CCT: 77.2 (64.1–87.2)	because only patients with ROTEM data were included. The cohort is small, leading to wide confidence intervals. The effect of each treatment algorithm on patient-relevant outcomes was not investigated.<br>The population excluded a potentially relevant group of patients on anticoagulant medication.

+: low risk; –: high risk; ?: unclear risk; A: Austria; A10: clot firmness by rotational thromboelastometry at 10 minutes; A5: clot firmness by rotational thromboelastometry at 5 minutes; ABC: assessment of blood consumption score; adj.: adjusted; AIS: Abbreviated Injury Scale; AP: anti-platelets; ARU: Aspirin Reaction Units; ASA: aspirin; ATC: acute traumatic coagulopathy; ATIC: Acute Trauma-Induced Coagulopathy; AUC: Area under the receiver operating characteristic curve; CCT: conventional coagulation tests; CG: control group; CI: Confidence Interval; CT: coagulation time; d: days; DFI: depletion of fibrinolytic inhibitors; ED: emergency department; GCS: Glasgow Coma Score; h: hours; HR: hazard ratio; Ht-TEG: thromboelastography assay with high dose of tissue plasminogen activator; IG: intervention group; INR: international normalized ratio; IQR: Interquartile Range; ISS: injury severity score; iSTBI: Isolated Severe Traumatic Brain Injury; iTACTIC: the implementation treatment algorithms for the correction of trauma-induced coagulopathy; L: litres; LoE: level of evidence; Lt-TEG rapid thromboelastography assay with low dose of tissue plasminogen activator; LY30; lysis at 30 min; m: months; MA: maximum amplitude; MAP: mean arterial pressure; MCF: Maximum Clot Firmness; MHP: major haemorrhage protocol; min: minutes; mmHg: millimetres of mercury; MODS: multiple organ dysfunction syndrome; MT: massive transfusion; MTP: massive transfusion protocol; n.r.: not reported; NPV: negative predictive value; PCC: prothrombin complex concentrate; PLT: platelets; PPV: positive predictive value; PT: prothrombin time; P-TEG: plasmin- thromboelastography; RBC: red blood cells; RIE: Edinburgh ROTEM algorithm; ROC: receiver operating curve; RR: Relative Risk; rTEG: rapid thromboelastography; s: seconds; SBP: systolic blood pressure; SD: Standard deviation; SI: shock index; TASH: trauma associated severe hemorrhage score; TBI: traumatic brain injury; TEG FF MA: maximum amplitude or maximum clot strength determined by standard functional fibrinogen thrombelastography test; TEG FIBTEM MA: maximum amplitude or maximum clot strength determined by a crossover test using ROTEM reagents on thrombelastography with the same reagent:blood ratio as the ROTEM FIBTEM test; TEG: Thromboelastography; TEG-PM AA: Thrombelastography Platelet Mapping percent inhibition of arachidonic acid; TEG-PM: Thrombelastography Platelet Mapping; TICCS: Trauma Induced Coagulopathy Clinical Score; TMA: time to maximum amplitude; tPA TEG: tissue plasminogen activator thromboelastography; tPA: tissue plasminogen activator; TXA: tranexamic acid; UK: United Kingdom; USA: United States of America; VHA: viscoelastic haemostatic assay; VN: Verify Now; y: years

Volume management, permissive hypotension, MAP targets

Study: Reference, aim, design, setting	Participants: selection criteria, characteristics	N Participants; Intervention (IG) vs. Control group (CG)	Main outcomes	Assessment: LoE, risk of bias; Conclusions
**Carrick (2016)**<br>“Intraoperative hypotensive resuscitation for patients undergoing laparotomy or	**Inclusion criteria**<br>• all penetrating trauma patients seen in Ben Taub Hospital Emergency Center (EC)<br>• SBP ≤90 mmHg	**Participants**<br>N=180 patients<br>**Study groups**	**Primary Outcome**<br><u>30d Mortality, n/N (%)</u><br>IG: 18/84 (21.4), p=0.47<br>CG: 21/80 (26.3)	**Level of evidence**<br>2b↓<br>**Risk of bias**<br>Selection bias: +

Study: Reference, aim, design, setting	Participants: selection criteria, characteristics	N Participants; Intervention (IG) vs. Control group (CG)	Main outcomes	Assessment: LoE, risk of bias; Conclusions
thoracotomy for trauma: Early termination of a randomized prospective clinical trial". *Journal of Trauma and Acute Care Surgery* 2016; 80(6): 886-896<br>**Study design**<br>Randomised controlled trial<br>**Aim of the study**<br>"The aim of the study was to assess if intraoperative hypotensive resuscitation would improve survival for patients undergoing operative control of hemorrhage following penetrating trauma."<br>**Setting**<br>USA, 2007-2013	• In need of laparotomy or thoracotomy<br>**Exclusion criteria**<br>• blunt mechanism of injury<br>• age of <14 years or >45 years (older patients could potentially have underlying cerebrovascular or cardiac disease)<br>• known or suspected head injury<br>• pregnant women<br>• incarcerated individuals<br>• patients with "opt-out" bracelets that signify their refusal of participation in the project<br>**Characteristics**<br><u>Age [y], median (range)</u><br>IG: 28 (16 - 54), p=0.24<br>CG: 32 (15 - 54)<br><u>Male, n (%)</u><br>IG: 79 (91.9), p=0.53<br>CG: 73 (89.0)<br><u>ISS, median (range)</u><br>IG: 17 (1 - 43), p=0.43<br>CG: 18 (4 - 75)<br><u>GCS, median (range)</u><br>IG: 15 (3 - 15), p=0.35<br>CG: 14 (3 - 15)<br><u>SBP [mmHg], median (range)</u><br>IG: 85 (11 - 161), p=0.16<br>CG: 79 (40 - 144)	IG: LMAP (target MAP ≥50 mmHg) (N=89 total, N=86 analysed)<br>CG: HMAP (target MAP ≥65 mmHg) (N=91 total, N=82 analysed)<br>Methods for achieving the target blood pressure goals were left to the discretion of the treating anesthesiologist.<br>If patients were able to spontaneously maintain a MAP greater than their assigned target, the blood pressure was not intentionally lowered.	**Other Outcomes**<br><u>Deaths due to exsanguination, n/N (% of deaths)</u><br>IG: 10/18 (56)<br>CG: 15/21 (71)<br><u>Intraoperative MAP [mmHg], mean ± SD</u><br>IG: 65.5 ± 11.6, p=0.07<br>CG: 69.1 ± 13.8<br><u>Percentage of time under target MAP (%)</u><br>IG: 12.6, p<0.001<br>CG: 35.2<br>**Postoperative Complications**<br><u>Acute renal injury, n/N (%)</u><br>IG: 10/75 (13.3), p=0.01<br>CG: 20/66 (30.3)<br><u>Coagulopathy, n/N (%)</u><br>IG: 21/75 (28.0), p=0.92<br>CG: 19/66 (28.8)	Performance bias: +<br>Attrition bias: +<br>Detection bias: +<br>**Authors' conclusion**<br>„The study was unable to demonstrate that hypotensive resuscitation at a target MAP of 50 mm Hg could significantly improve 30-day mortality. Further, the trial was terminated early because of temporal changes in processes of care, lack of equipoise, slow accrual, and futility and therefore was underpowered."<br>**Reviewers' conclusion**<br>Physicians and investigators were not/could not be blinded, but both groups received similar co-interventions. The study was underpowered to detect differences in mortality due to early termination.
**Gu (2020)**<br>„Restricted fluid resuscitation improves the	**Inclusion criteria**<br>• Patients who met the diagnostic criteria of hemorrhagic shock in the	**Participants**<br>N=160 patients	<u>MAP after resuscitation [mmHg]: mean ± SD</u><br>IG: 61.3 ± 3.5 (p<0.001)	**Level of evidence**<br>1b

Study: Reference, aim, design, setting	Participants: selection criteria, characteristics	N Participants; Intervention (IG) vs. Control group (CG)	Main outcomes	Assessment: LoE, risk of bias; Conclusions
prognosis of patients with traumatic hemorrhagic shock”. *International Journal of Clinical and Experimental Medicine* 2020; 13(7): 5319-5327<br>**Study design**<br>Prospective randomised controlled trial<br>**Aim of the study**<br>“To explore the effect of restricted fluid resuscitation on coagulation, the serum inflammatory factors, and the prognoses of patients with traumatic hemorrhagic shock.“<br>**Setting**<br>China, 2018 – 2020	“Chinese emergency medicine expert consensus on diagnosis and treatment of traumatic hemorrhagic shock” issued by the Chinese College of Emergency Physicians in 2017<br>• patients who were admitted to our hospital for the first time and who were admitted within 6 hours of their arrival and who had not been transferred<br>• patients who were over 18 years old<br>**Exclusion criteria**<br>• Patients who had dysfunction of the vital organs including the liver and kidneys before their admission<br>• patients who were admitted with MODS and ARDS<br>• patients with incomplete clinical data<br>**Characteristics**<br>Age [years], mean ± SD<br>IG: 36.8 ± 7.5 (p=0.313)<br>CG: 38.1 ± 8.7<br>Gender, male / female, n (%)<br>IG: 59 (73.8%) / 21 (26.2%) (p=0.301)<br>CG: 53 (66.3%) / 27 (34.7%)<br>Shock index, mean ± SD<br>IG: 2.1 ± 0.4 (p=0.116)<br>CG: 2.2 ± 0.4<br>Injury Severity Score (ISS), mean ± SD<br>IG: 27.6 ± 3.2 (p=0.149)<br>CG: 28.3 ± 2.9	**Study groups**<br>IG: restricted fluid resuscitation (N=80)<br>• 500 - 1,500 mL of compound sodium chloride solution given within 30-60 min<br>• Then 500 mL of hydroxyethyl starch given for the resuscitation<br>• The total liquid infusion volume ranged from 1,500 mL to 2,000 mL<br>• MAP 50-60 mmHg<br>CG: routine fluid resuscitation (N=80)<br>• 1,500 - 2,000 mL of compound sodium chloride solution given for volume expansion<br>• Then 500 - 1,000 mL of hydroxyethyl starch given for the resuscitation<br>• MAP 60-80 mmHg	CG: 71.1 ± 4.6<br>**Comparison of the prognosis**<br>Death: n (%)<br>IG: 5 (6.3) (p=0.045)<br>CG: 13 (16.3)<br>Acute Respiratory Distress Syndrome (ARDS): n (%)<br>IG: 10 (12.5) (p=0.018)<br>CG: 22 (27.5)<br>Multiple Organ Dysfunction Syndrome (MODS): n (%)<br>IG: 7 (8.8) (p=0.017)<br>CG: 18 (22.5)	**Risk of bias**<br>Selection bias: ?<br>Performance bias: ?<br>Attrition bias: ?<br>Detection bias: +<br>**Authors’ conclusion**<br>„Compared with traditional aggressive fluid resuscitation, restricted fluid resuscitation can improve patient prognosis, effectively reducing the mortality and decreasing the incidences of ARDS and MODS.“<br>**Reviewers’ conclusion**<br>Because of incomplete reporting, the risk of selection, performance, and attrition bias cannot be assessed since no information on the randomization process, blinding and completeness of the data is available.

Study: Reference, aim, design, setting	Participants: selection criteria, characteristics	N Participants; Intervention (IG) vs. Control group (CG)	Main outcomes	Assessment: LoE, risk of bias; Conclusions
	<u>Infusion volume during fluid resuscitation [mL], mean ± SD</u><br>IG: 1526.4 ± 115.7 mL (p<0.001)<br>CG: 2754.9 ± 153.8 mL<br><u>MAP at time of admission [mmHg], mean ±SD</u><br>IG: 59.3 ± 6.3 (p=0.222)<br>CG: 58.6 ± 6.9			
**Lu 2018**<br>"Controlled blood pressure elevation and limited fluid resuscitation in the treatment of multiple injuries in combination with shock ". *Pakistan Journal of Medical Sciences* 2018; 34(5): 1120-1124<br>**Study design**<br>Randomised controlled trial<br>**Aim of the study**<br>"The aim of the study was to explore the effectiveness of controlled blood pressure elevation and limited fluid resuscitation in treating patients with multiple injuries in combination with shock in Intensive Care Unit (ICU). "<br>**Setting**	**Inclusion criteria**<br>• admission to ICU due to severe multiple injuries<br>• injury severity score >16<br>• hemorrhagic shock<br>• average arterial pressure <65 mmHg or systolic pressure <40 mmHg<br>• undergone hemostatic treatment one or two hours after admission<br>**Exclusion criteria**<br>• death within 24 hours after admission to ICU<br>• craniocerebral trauma, severe cardiopulmonary and hepatic and renal dysfunction or severe hypertension<br>**Characteristics**<br><u>Age [y], mean ± SD</u><br>IG: 32.3 ± 4.2 (p<0.05)<br>CG: 33.6 ± 4.2<br><u>Sex, n/N</u><br>IG: 53/82 male, 29/82 female<br>CG: 57/82 male, 25/82 female<br><u>Shock, n/N</u><br>*Severe*	**Participants**<br>N=164 patients<br>**Study groups**<br>IG: controlled blood pressure elevation and limited fluid resuscitation with 7.5% sodium chloride solution and plasma solution (N=82)<br>• MAP: 40-50 mmHg<br>CG: conventional fluid resuscitation and controlled blood pressure elevation (N=82)<br>• MAP: 60-80 mmHg	**Primary outcomes**<br><u>Recovery time [min], mean ± SD</u><br>IG: 89.7 ± 25.2 (p=0.000)<br>CG: 193.5 ± 38.7<br><u>Hemoglobin [g/L], mean ± SD</u><br>IG: 102.5 ± 13.0 (p=0.006)<br>CG: 84.6 ± 8.3<br><u>Prothrombin time [s], mean ± SD</u><br>IG: 10.1±13.0<br>CG: 16.9±2.4 p=0.000<br><u>C-reactive protein level [mg/L], mean ± SD</u><br>IG: 101.7±12.3<br>GC: 132.4±20.6, p=0.000<br>**Other outcomes**<br><u>Fatality Rate, n/N (%)</u><br>IG: 2/82 (2.4) (p=0.041)<br>CG: 15/82 (18.3)<br><u>Lactate Clearance [mmol/L], mean ± SD</u><br>Blood lactic acid before resuscitation<br>IG: 5.73 ± 1.29 (p=0.163)<br>CG: 5.94 ± 1.61	**Level of evidence**<br>1b<br>**Risk of bias**<br>Selection bias: +<br>Performance bias: ?<br>Attrition bias: ?<br>Detection bias: ?<br>**Authors' conclusion**<br>"Controlled blood pressure elevation in combination with limited fluid resuscitation is more effective than conventional fluid resuscitation in the treatment of patients with multiple injuries and shock in ICU as it can shorten recovery time, improve microcirculation perfusion and prognosis, and reduce related complications and fatality rate."<br>**Reviewers' conclusion**

Study: Reference, aim, design, setting	Participants: selection criteria, characteristics	N Participants; Intervention (IG) vs. Control group (CG)	Main outcomes	Assessment: LoE, risk of bias; Conclusions
**China, 2014-2017**	IG: 37/82<br>CG: 39/82<br>*Moderate*<br>IG: 31/82<br>CG: 33/82<br>*Mild*<br>IG: 14/82<br>CG: 10/82		Lactate Clearance Rate at 3h<br>IG: 0.22 ± 0.01 (p=0.008)<br>CG: 0.27 ± 0.03<br>Lactate Clearance Rate at 6h<br>IG: 0.37 ± 0.06 (p=0.000)<br>CG: 0.51 ± 0.08<br>Lactate Clearance Rate at 24h<br>IG: 0.77 ± 0.04 (p=0.179)<br>CG: 0.76 ± 0.04<br><u>Complications, n/N (%)</u><br>Disseminated intravascular coagulation<br>IG: 2/82 (2.4) (p=0.039)<br>CG: 14/82 (17.1)<br>Respiratory distress syndrome<br>IG: 10/82 (12.2) (p=0.006)<br>CG: 25/82 (30.5)<br>**Multiple organ dysfunction syndrome**<br>**IG: 10/82 (12.2) (p=0.027)**<br>**CG: 24/82 (29.3)**	**There might be a risk of performance and detection bias, as the study does not provide sufficient information on blinding and length of follow up.**

+: low risk; –: high risk; ?: unclear risk; adj.: adjusted; AIS: Abbreviated Injury Scale; ARDS: Acute Respiratory Distress Syndrome; BD: base deficit; CG: control group; CI: Confidence Interval; d: days; GCS: Glasgow Coma Score; h: hours; IG: intervention group; IQR: Interquartile Range; ISS: injury severity score; ITT: Intention to Treat analysis; L: litres; LoE: level of evidence; m: months; MAP: mean arterial pressure; min: minutes; mmHg: millimetres of mercury; MODS: multiple organ dysfunction syndrome; RR: Relative Risk; s: seconds; SBP: systolic blood pressure; SD: Standard Deviation; TBI: traumatic brain injury; y: years

Colloids

Study: Reference, aim, design, setting	Participants: selection criteria, characteristics	N Participants; Intervention (IG) vs. Control group (CG)	Main outcomes	Assessment: LoE, risk of bias; Conclusions
**Han (2015)**<br>"Comparison of 3% And 7.5% Hypertonic Saline in Resuscitation after Traumatic Hypovolemic	**Inclusion criteria**<br>• trauma victims with prehospital SBP≤70 mmHg or 70 to 90 mmHg<br>• heart rate (HR) of ≥108 beats/min<br>• aged 15 years or older<br>**Exclusion criteria**	**Patients**<br>N=246 patients<br>**Study groups**<br>IG1: 3% HSS (Hypertonic saline solution) (N=82)	<u>Mortality, n (%)</u><br>Total deaths: 37 (15.4)<br>Deaths within first 24 h: 30 (81.1)<br>24-h survival in IG1 and IG2 better than CG, but no statistically significant difference	**Level of evidence**<br>1b<br>**Risk of bias**<br>Selection bias: +<br>Performance bias: +

Study: Reference, aim, design, setting	Participants: selection criteria, characteristics	N Participants; Intervention (IG) vs. Control group (CG)	Main outcomes	Assessment: LoE, risk of bias; Conclusions
Shock". *Shock* 2015; 43(3): 244-249<br><br>**Study design**<br>Randomised controlled trial<br><br>**Aim of the study**<br>"The aim of this study was to evaluate the resuscitative effects and safety of 3% Hypertonic saline solution (HSS) and to compare the risks of complications caused by HSS and standard fluid treatments."<br><br>**Setting**<br>China, 2008-2012	• younger than 15 years<br>• injury during previous 4 h<br>• hypothermia (<28°C)<br>• administration of dopamine or other vasoactive agents<br>• administration of more than 2,000 mL of crystalloid before the study fluid<br>• ongoing cardiopulmonary resuscitation<br>• severe cardio-respiratory dysfunction<br>• known or suspected pregnancy<br>• traumatic brain injury (TBI)<br>• death within 1 h after intervention<br><br>**Characteristics**<br><br>Age, years (mean ± SD)<br>IG1: 45 ± 0.5<br>IG2: 48 ± 3.1<br>CG: 43 ± 9.5<br><br>Males, n (%))<br>IG1: 61 (74.4)<br>IG2: 65 (81.3)<br>CG: 63 (75.0)<br><br>ISS (mean ± SD)<br>IG1: 18.5 ± 2.5<br>IG2: 15.6 ± 3.1<br>CG: 16.5 ± 3.4<br><br>Shock index (mean ± SD)<br>IG1: 1.5 ± 0.2<br>IG2: 1.6 ± 0.3<br>CG: 1.5 ± 0.2<br><br>Preinfusion MAP, mmHg (mean ± SD)<br>IG1: 49 ± 6.6<br>IG2: 51 ± 9.7<br>CG: 52 ± 4.7<br><br>Infusion volume, 1 h, L (mean ± SD)	IG2: 7.5% HSS (Hypertonic saline solution) (N=80)<br><br>• CG: LRS (standard fluid, LactDiated Ringer's solution) (N=84)	**Postinfusion complications, n (%)**<br><br>Tachycardia<br>IG1: 5 (6.1)<br>IG2: 22 (27.5) (vs. IG1 and CG, p<0.05)<br>CG: 4 (4.8)<br><br>Coagulopathy<br>IG1: 0<br>IG2: 2 (2.5)<br>CG: 9 (10.7) (vs. IG1 and IG2, p<0.001)<br><br>Acute renal failure<br>IG1: 0<br>IG2: 0<br>CG: 5 (6.0) (vs. IG1 and IG2, p<0.001)<br><br>Pulmonary edema<br>IG1: 0<br>IG2: 0<br>CG: 4 (4.8) vs. IG1 and IG2, p<0.001)<br><br>Heart failure<br>IG1: 1 (1.2)<br>IG2: 1 (1.3)<br>CG: 2 (2.4)<br><br>Transient Hypotension<br>IG1: 0<br>IG2: 4 (5.0) (vs. IG1 and CG p<0.05)<br>CG: 0<br><br>ARDS<br>IG1: 1 (1.2)<br>IG2: 1 (1.3)<br>CG: 3 (3.6)<br><br>MODS	Attrition bias: ?<br><br>Detection bias: ?<br><br>**Authors' conclusion**<br>"In summary, administration of 3% HSS offered hemodynamic benefits equivalent to those of 7.5% HSS infusion with lower degrees of hypernatremia and hyperchloremia and lower risks of cardiac dysrhythmia and transient hypotension. In addition, higher incidences of pulmonary edema, renal failure, and coagulopathy occurred in the LRS group."<br><br>**Reviewers' conclusion**<br>There might be a risk of attrition bias as information regarding length of follow-up regarding adverse events and the availability of outcome data are lacking.

Study: Reference, aim, design, setting	Participants: selection criteria, characteristics	N Participants; Intervention (IG) vs. Control group (CG)	Main outcomes	Assessment: LoE, risk of bias; Conclusions
	IG1: 1.1 ± 0.2<br>IG2: 1.0 ± 0.2<br>CG: 2.1 ± 0.3 (CG vs. IG1 and IG2, p<0.05)		IG1: 2 (2.4)<br>IG2: 1 (1.3)<br>CG: 3 (3.6)	
+: low risk; –: high risk; ?: unclear risk; adj.: adjusted; CG: control group; CI: Confidence Interval; d: days; g: grams; HR: Heart rate; HSS: Hypertonic saline solution; IG: intervention group; IQR: Interquartile Range; ISS: injury severity score; LoE: level of evidence; m: months; MAP: mean arterial pressure; min: minutes; mmHg: millimetres of mercury; mg: milligrams; MODS: multiple organ dysfunction syndrome; s: seconds; SBP: systolic blood pressure; SD: Standard Deviation; SOFA: Sequential Organ Failure Assessment; y: years				

Base excess/lactate

Study: Reference, aim, design, setting	Participants: selection criteria, characteristics	N Participants; Intervention (IG) vs. Control group (CG)	Main outcomes	Assessment: LoE, risk of bias; Conclusions
**Fligor (2017)**<br>Parathyroid hormone as a marker for hypoperfusion in trauma: A prospective observational study." *The Journal of Trauma and Acute Care Surgery* 2017; 83(6): 1142-1147.<br>**Study design**<br>Prognostic cross-sectional study<br>**Aim of the study**<br>"We hypothesized that early hyperparathyroidism predicts mortality and transfusion in trauma patients."<br>**Setting**<br>n.r. (authors from USA), 2016	**Inclusion criteria**<br>• ≥18 years<br>• received the highest level of trauma team activation<br>**Exclusion criteria**<br>• administration of blood products before phlebotomy<br>• pregnancy<br>• chronic kidney disease (stage III or worse)<br>• primary or secondary bone malignancy<br>• history of hyperparathyroidism or hypoparathyroidism<br>• history of hypercalcemia or hypocalcemia<br>• Labs incorrectly drawn<br>• Did not speak English<br>**Characteristics**<br>Age [y], median (IQR)<br>47 (29-64)<br>Male, %$<br>82.6	**Participants**<br>N=46 patients<br>**Tests evaluated**<br>Test 1: parathyroid hormone (PTH)<br>Test 2: lactic acid<br>Phlebotomists obtained specimens in the trauma bay before administration of blood products. The laboratory processed the samples according to normal laboratory procedures and recorded results in the medical record. The reference range for intact PTH is 9.2 pg/mL to 79.5 pg/mL, and the range for ionized calcium is 1.13 mmol/L to 1.32 mmol/L.	**Prediction of transfusions within 24h**<br>AUC<br>PTH: 0.876 vs. lactic acid: 0.793<br>Prognostic test performance with PTH≥100 pg/mL<br>Sensitivity: 88%<br>Specificity: 86%<br>Positive predictive value: 79%<br>Negative predictive value: 93%<br>**Prediction of mortality**<br>AUC<br>PTH: 0.875 vs. lactic acid: 0.835<br>Prognostic test performance with PTH≥100 pg/mL<br>Sensitivity: 90%<br>Specificity: 72%<br>Positive predictive value: 47%<br>Negative predictive value: 96%	**Level of evidence**<br>3b↓<br>**Risk of bias**<br>no tool available for prognostic studies<br>**Authors' conclusion**<br>"Hyperparathyroidism on hospital arrival in trauma patients predicts mortality and transfusion in the first 24 hours."<br>**Reviewers' conclusion**<br>The results should be interpreted with caution because the threshold was not pre-specified and test performance values of lactic acid are not presented. The majority of patients was not severely injured. The effect on patient-relevant outcomes of using the index test to

Study: Reference, aim, design, setting	Participants: selection criteria, characteristics	N Participants; Intervention (IG) vs. Control group (CG)	Main outcomes	Assessment: LoE, risk of bias; Conclusions
	<u>ISS, median (IQR)</u><br>12 (7-18)<br><u>TRISS, median (IQR)</u><br>0.96 (0.80-0.99)<br><u>Parathyroid hormone [pg/mL], median (IQR)</u><br>95.2 (63.3–149.4)<br><u>Lactic acid [mmol/L], median (IQR)</u><br>2.8 (1.9–4.9)<br>$ n/N not reported			take therapeutic decisions was not investigated.
**Gale (2016)**<br>"A comparison of initial lactate and initial base deficit as predictors of mortality after severe blunt trauma." *Journal of surgical research* 2017; 205(2): 446-455.<br>**Study design**<br>Prognostic cross-sectional study<br>(Glue Grant Trauma-Related Database)<br>**Aim of the study**<br>"Our objective was to compare initial BD with lactate as predictors of inhospital mortality in a large cohort of blunt trauma patients all presenting with hemorrhagic shock."	**Inclusion criteria**<br>• Blunt trauma mechanism<br>• Abbreviated Injury Scale (AIS) severity score >2 outside the head region<br>• Emergency department (ED) arrival<br>• SBP <90 mm Hg or BD >6 mEq/L (prehospital or within 60 min of arrival)<br>• Blood transfusion within 12 h of injury<br>• Intact cervical spinal cord<br>• Records that contained both data points "Initial_Base_Deficit" and "ER_lactate" (ER = emergency room)<br>**Exclusion criteria**<br>• n.r.<br>**Characteristics**<br><u>Age [y], mean ± SD</u><br>42.8 ± 18.7<br><u>Male, %$</u><br><u>66</u><br><u>GCS, mean ± SD</u>	**Participants**<br>N=1,829 patients<br>**Tests evaluated**<br>Test 1: initial base deficit (BD)<br>Test 2: initial lactate<br>**Variables in multivariable logistic regression model**<br>• age<br>• gender<br>• race<br>• ISS<br>• APACHE II	**Overall population**<br><u>Inhospital mortality, multivariable adj. OR (95% CI)</u><br>Initial BD: 1.04 (1.01-1.07), p<0.005<br>Initial lactate: 1.17 (1.12-1.23), p<0.00001<br>"for each 1 meq/L increase in BD, mortality risk increased by 4%; for each 1 mmol/L increase in lactate, mortality risk increased by 17%"<br><u>AUC for prediction of total mortality</u><br>Initial BD: 0.6135<br>Initial lactate: 0.7071<br><u>AUC for prediction of mortality after 24h</u><br>Initial BD: 0.5749<br>Initial lactate: 0.6726<br>**Shock subgroup (lactate ≥4 mmol/L)**<br><u>Inhospital mortality, multivariable adj. OR (95% CI)</u><br>Initial BD: 1.04 (1.01-1.08), p<0.03<br>Initial lactate: 1.15 (1.08-1.22), p<0.00001	**Level of evidence**<br>3b↓<br>**Risk of bias**<br>no tool available for prognostic studies<br>**Authors' conclusion**<br>"After severe blunt trauma, initial serum lactate is superior to initial BD in predicting inhospital survival in patients with and without shock. Initial BD does not predict mortality for patients whose survival is longer than 24 h."<br>**Reviewers' conclusion**<br>The results should be interpreted with caution because of the retrospective data analysis, unknown exclusion criteria,

Study: Reference, aim, design, setting	Participants: selection criteria, characteristics	N Participants; Intervention (IG) vs. Control group (CG)	Main outcomes	Assessment: LoE, risk of bias; Conclusions
**Setting**<br>USA, 2002-2011	8.4 ± 5.6<br>ISS, mean ± SD<br>38.9 ± 14.0<br>Apache II, mean ± SD<br>29.1 ± 7.2<br>Initial base deficit [mEq/L], mean ± SD<br>8.81 ± 4.80<br>Initial lactate [mmol/L], mean ± SD<br>4.57 ± 2.86<br><br>$ n/N not reported		AUC for prediction of total mortality<br>Initial BD: 0.5975<br>Initial lactate: 0.6591<br>AUC for prediction of mortality after 24h<br>Initial BD: 0.5410<br>Initial lactate: 0.6103<br>**TBI (head AIS>3) subgroup**<br>Inhospital mortality, multivariable adj. OR (95% CI)<br>Initial BD: 0.98 (0.93-1.04), p=0.55<br>Initial lactate: 1.11 (1.02-1.22), p<0.03<br>TBI (head AIS ≤3) subgroup<br>Inhospital mortality, multivariable adj. OR (95% CI)<br>Initial BD: 1.07 (1.03-1.10), p<0.0005<br>Initial lactate: 1.20 (1.13-1.27), p<0.00001	and unknown methods of testing. Only AUC values were evaluated, and no cut-offs were derived, which limits the applicability of the study results. The effect on patient-relevant outcomes of using the index test to take therapeutic decisions was not investigated. The study population was limited to blunt trauma patients in haemorrhagic shock.
**Hutchings (2018)**<br>Microcirculatory Impairment Is Associated With Multiple Organ Dysfunction Following Traumatic Hemorrhagic Shock: The MICROSHOCK Study." *Critical Care Medicine* 2018; 46(9): e889-e896.<br>**Study type**<br>Prognostic cross-sectional study<br>**Aim of the study**<br>"The aim of the present study was to examine the	**Inclusion criteria**<br>• had been injured<br>• had required blood product transfusion during resuscitation<br>• lactate concentration ≥2 mmol/L at any stage prior to enrolment<br>• were intubated and ventilated<br>• enrolment as early as was feasible up to 12h after ICU admission<br>**Exclusion criteria**<br>• unsurvivable injuries with a palliative focus of care<br>• facial injuries that precluded hand-held videomicroscopy<br>• insufficient quality of microcirculatory video clips	**Participants**<br>N=60 patients (N=58 after 2 were excluded due to insufficient quality of microcirculatory video clips)<br>**Tests evaluated**<br>Microcirculatory measurements:<br>An assessment was made of microcirculatory perfusion using incident dark field (IDF) videomicroscopy in the tongue. Videos are stored and analysed later. These video analyses give values for total vessel density (TVD), perfused vessel density (PVD), proportion of perfused vessels (PPV), microcirculatory flow index (MFI), and microcirculatory heterogeneity index (MHI)	**Prediction of MODS at day 7, AUC**<br>Perfused vessel density: 0.87 (0.76–0.99)<br>Microcirculatory flow index: 0.83 (0.71–0.95)<br>Lactate: 0.69 (0.53–0.84)	**Level of evidence**<br>3b↓<br>**Risk of bias**<br>no tool available for prognostic studies<br>**Authors' conclusion**<br>"Microcirculatory hypoperfusion immediately following traumatic hemorrhagic shock and resuscitation is associated with increased multiple organ dysfunction syndrome. Microcirculatory variables are better prognostic indicators for

Study: Reference, aim, design, setting	Participants: selection criteria, characteristics	N Participants; Intervention (IG) vs. Control group (CG)	Main outcomes	Assessment: LoE, risk of bias; Conclusions
association between microcirculatory impairment and MODS (…) and to investigate whether there is a threshold of microcirculatory perfusion that might be predictive of MODS."<br><br>**Setting**<br><br>UK, 2014-2017	**Characteristics**<br><br><u>Age [y], mean ± SD</u><br>43 ± 19<br><br><u>Male, %</u><br>81<br><br><u>ISS, mean ± SD</u><br>29 ± 14<br><br><u>Blunt mechanism of injury, %</u><br>75<br><br><u>Highest lactate concentration prior to ICU admission [mmol/L], mean ± SD</u><br>7.3 ± 6.1<br><br><u>Lowest recorded SBP [mmHg], mean ± SD</u><br>69 ± 27	• D0: following bleeding control procedures, but less than 12 hours after admission to the ICU<br>• D1: D0 + 24 hours<br>• D2 D0 + 48 hours<br><br>Lactate: Highest lactate prior to ICU admission<br><br>multiple organ dysfunction syndrome (MODS) defined as Sequential Organ Failure Assessment (SOFA) Score ≥6 at day 7		the development of multiple organ dysfunction syndrome than more traditional indices."<br><br>**Reviewers' conclusion**<br><br>Only AUC values were evaluated, and no cut-offs were derived, which limits the applicability of the study results. The utility of video analysis for treatment decisions and patient outcomes has not yet been established.
+: low risk; –: high risk; ?: unclear risk; adj.: adjusted; AIS: Abbreviated Injury Scale; AUC: Area under the receiver operating characteristic curve; BD: base deficit; CG: control group; CI: Confidence Interval; d: days; ED: Emergency department; ER: emergency room; GCS: Glasgow Coma Score; h: hours; ICU: intensive care unit; IG: intervention group; IQR: Interquartile Range; ISS: injury severity score; ITT: Intention to treat analysis; L: litres; LoE: level of evidence; m: months; MAP: mean arterial pressure; min: minutes; mmHg: millimetres of mercury; MODS: multiple organ dysfunction syndrome; n.r.: not reported; OR: odds ratio; PTH: parathyroid hormone; RR: Relative Risk; s: seconds; SBP: systolic blood pressure; SD: Standard deviation; SOFA: Sequential Organ Failure Assessment; TBI: traumatic brain injury; TRISS: Trauma and Injury Severity Score; UK: United Kingdom; y: years				

Temperature management

Study: Reference, aim, design, setting	Participants: selection criteria, characteristics	N Participants; Intervention (IG) vs. Control group (CG)	Main outcomes	Assessment: LoE, risk of bias; Conclusions
**Cooper (2018)**<br><br>"Effect of early sustained prophylactic hypothermia on neurologic outcomes among patients with severe traumatic brain injury: the POLAR randomized clinical trial."	**Inclusion criteria**<br>• Traumatic brain injury<br>• Age: 18 to 60 y<br>• GCS<9<br>• and had actual or imminent endotracheal intubation<br><br>**Exclusion criteria**	**Participants**<br><br>N=511 patients<br><br>**Study groups**<br><br>IG: hypothermia: 33°C ± 0.5°C (N=266 randomized, N=240 primary outcomes	**Primary outcome**<br><br><u>Favourable outcome (GOS-E score 5-8) at six months, n/N (%)</u><br>IG: 117/240 (48.8) vs. CG: 111/226 (49.1)<br><br><u>Absolute difference (95% CI): -0.4 (-9.4 to 8.7)</u><br>RR (95% CI): 0.99 (0.82-1.19), p=0.94	**Level of evidence**<br><br>1b<br><br>**Risk of bias**<br><br>Selection bias: +<br><br>Performance bias: ?

Study: Reference, aim, design, setting	Participants: selection criteria, characteristics	N Participants; Intervention (IG) vs. Control group (CG)	Main outcomes	Assessment: LoE, risk of bias; Conclusions
JAMA 2018; 320(21): 2211-2220.<br>**Study design**<br>Randomised controlled trial<br>(POLAR-RCT)<br>**Aim of the study**<br>"To determine the effectiveness of early prophylactic hypothermia compared with normothermic management of patients after severe traumatic brain injury."<br>**Setting**<br>Australia, New Zealand, France, Switzerland, Saudi Arabia, and Qatar, 2010-2017	• significant bleeding suggested by systolic hypotension (<90mmHg)<br>• or sustained tachycardia (>120/min),<br>• suspected pregnancy,<br>• possible uncontrolled bleeding, GCS≤3 and unreactive pupils,<br>• or destination hospital not a study site<br>**Characteristics**<br><u>Age [y], mean ± SD</u><br>IG: 35.0 ± 13.5 vs. CG: 34.1 ± 13.4<br><u>Male, n (%)</u><br>IG: 207 (79.6) vs. CG: 194 (80.8)<br><u>GCS, median (IQR)</u><br>IG: 6 (4-7) vs. CG: 6 (4-7)<br>Motor GCS, median (IQR)<br>IG: 3 (1-4) vs. CG: 3 (2-5)<br>ISS, median (IQRI<br>IG: 26.0 (18-34) vs. CG: 20 (20.5-35)	analyzed in ITT, N=256 secondary outcomes analyzed in ITT)<br>• induced by a bolus of up to 2000 mL intravenous ice-cold (4°C) 0.9% saline and surface-cooling wraps once the patient was in the ED<br>• targeting an initial core temperature of 35°C<br>• once significant clinical risk for bleeding was excluded, target core temperature was 33°C<br>• Hypothermia was maintained with Gymar Meditherm 3 console with surface-cooling wraps for at least 72h after randomization<br>CG: Normothermia: 37°C ± 0.5°C (N=245 randomized, N=226 primary outcome analyzed in ITT, N=239 secondary outcome analyzed in ITT)<br>**Co-interventions**<br>Patients in both groups could receive other treatments for evaluated intracranial pressure as clinically indicated, and in both study groups care was recommended to be managed according to international traumatic brain injury guidelines<br>•	<u>Severity-adj. relative risk for favourable outcome (IMPACT-TBI) at six months (95% CI)</u><br>0.98 (0.87-1.11), p=0.75<br>**Secondary outcomes**<br><u>Death in hospital, n/N (%)</u><br>IG: 52/260 (20.0) vs. CG: 43/239 (18.0)<br>Absolute difference (95% CI): 2.0 (−4.9 - 8.9)<br>RR (95% CI): 1.11 (0.77-1.60), p=0.57<br><u>Death at 6 months, n/N (%)</u><br>IG: 54/256 (21.1) vs.CG: 44/239 (18.4)<br>Absolute difference (95% CI): 2.7 (−4.3 - 9.7)<br>RR (95% C). 1.15 (0.80-1.64), p=0.45<br><u>Pneumonia, n/N (%)</u><br>IG: 143/260 (55.0) vs. CG: 123/240 (51.3)<br>Absolute difference (95% CI): 3.8 (−5.0 to 12.5)<br>RR (95% CI): 1.07 (0.91-1.27), p=0.40<br><u>Bacteremia, n/N (%)</u><br>IG: 19/260 (7.3) vs. CG: 12/240 (5.0)<br>Absolute difference (95% CI): 2.3 (−1.9 to 6.5)<br>RR (95% CI): 1.46 (0.72-2.95), p=0.29<br><u>Other infection, n/N (%)</u><br>IG: 36/260 (13.8) vs. CG: 38/240 (15.8)<br>Absolute difference (95% CI): −2.0 (−8.2 - 4.3)<br>RR (95% CI): 0.87 (0.57-1.33), p=0.53<br><u>New or increased intracranial bleeding, n/N (%)</u><br>IG: 47/260 (18.1) vs. CG: 37/240 (15.4)<br>Absolute difference (95% CI): 2.7 (−3.9 - 9.2)	Attrition bias: +<br>Detection bias: +<br>**Authors' conclusion**<br>"Among patients with severe traumatic brain injury, early prophylactic hypothermia compared with normothermia did not improve neurologic outcomes at 6 months. These findings do not support the use of early prophylactic hypothermia for patients with severe traumatic brain injury."<br>**Reviewers' conclusion**<br>Participants randomised to the intervention group had a higher median ISS but were comparable in other baseline characteristics. Due to non-blinded treating physicians, the study has a risk for performance bias.<br>A treatment effect may have been concealed because 32% of patients in the hypothermia group never reached the target temperature of 33°C.

Study: Reference, aim, design, setting	Participants: selection criteria, characteristics	N Participants; Intervention (IG) vs. Control group (CG)	Main outcomes	Assessment: LoE, risk of bias; Conclusions
			RR (95% CI): 1.23 (0.43-3.5), p=0.70<br><br><u>New significant extracranial bleeding, n/N (%)</u><br>IG: 8/260 (3.1) vs. CG: 6/240 (2.5)<br>Absolute difference (95% CI): 0.6 (−2.3 - 3.5)<br>RR (95% CI): 1.17 (0.79-1.74), p=0.43	
**Hifumi (2017)**<br>"Therapeutic hypothermia in patients with coagulopathy following severe traumatic brain injury." *Scandinavian journal of trauma, resuscitation and emergency medicine* 2017; 25(1): 1-8.<br><br>**Study design**<br>Randomised controlled trial<br>(secondary analysis of the B-HYPO trial)<br><br>**Aim of the study**<br>"The purpose of the present study was to examine the effect of coagulopathy on the safety of MTH compared to control in patients with severe TBI."<br><br>**Setting**<br>Japan, 2002-2008	**Inclusion criteria**<br>• age 15–69 years<br>• Glasgow Coma Scale (GCS) score of 4–8<br>• ability to initiate cooling within 2 h after the onset of TBI<br><br>**Exclusion criteria (according to B-HYPO protocol)**<br>• good motor response (GCS motor response=6)<br>• SBP <90 mmHg after fluid and vasopressor resuscitation<br>• platelet count <50,000 /mm^3<br>• severe pre-existing medical conditions (e.g., liver, kidney, or heart failure, or severe arrhythmia)<br>• acute myocardial infarction, pregnancy<br>• severe alcohol intoxication that prevented assessment of consciousness<br>• penetrating brain injury<br>• epidural hematoma without brain parenchymal injury<br>• core body temperature <30°C<br>• *in post-hoc analysis*: missing data activated partial thromboplastin time and fibrin/fibrinogen degradation products<br><br>**Characteristics**<br><br>**Coagulopathy subgroup**	**Participants**<br>N=132 patients in this post-hoc analysis (of N=150 in B-HYPO study)<br><br>**Study groups**<br>IG: mild therapeutic hypothermia (32.0-34.0°C)(N=79 of N=88 in B-HYPO study)<br>• N=20 Coagulopathy<br>• N=59 Non-coagulopathy<br><br>CG: 35.5-37.0°C (N=40 of N=47 in B-HYPO study)<br>• N=12 Coagulopathy<br>• N=28 Non-coagulopathy<br><br>The goal in each group was to achieve the targeted temperature within 6 h of the onset of TBI and to maintain this temperature for at least 72 h, predominantly using surface cooling blankets. After 72 h, the temperature was maintained at <38 °C until 7 days after the onset of TBI.	**Coagulopathy**<br><u>Favourable GOS outcome at 6 m§: n/N (%)</u><br>IG: 7/20 (35) vs. CG: 4/12 (33.3), p=1.00<br><u>Survival rate at 6 m: n/N (%)</u><br>IG: 12/20 (60.0) vs. CG: 6/12 (50.0), p=0.72<br><u>Overall complication rate during temperature management: n/N (%)</u><br>IG: 3/20 (15) vs. CG: 1/12 (8.3), p=1<br><br>**Non-Coagulopathy**<br><u>Favourable GOS outcome at 6 m§: n/N (%)</u><br>IG: 28/59 (47.5) vs. CG: 16/28, p=0.49<br><u>Survival rate at 6 m: n/N (%)</u><br>IG: 38/59 (64.4) vs. CG: 25/28 (89.3), p=0.02<br><u>Overall complication rate during targeted temperature management: n/N (%)</u><br>IG: 12/59 (20.3) vs. CG: 0/28 (0), p<0.01<br><br>**§Good recovery and moderate disability according to the GOS scores at 6 months after injury were designated as a favourable neurological outcome.**	**Level of evidence**<br>2b↓<br><br>**Risk of bias**<br>Selection bias: +<br>Performance bias: ?<br>Attrition bias: +<br>Detection bias: +<br><br>**Authors' conclusion**<br>"Our study suggests that in comparison to control, MTH does not worsen the outcome of patients with coagulopathy following severe TBI."<br><br>**Reviewers' conclusion**<br>The results of this post-hoc analysis should be interpreted with caution due to the small subgroups and higher proportion of excluded patients in the CG due to missing data for coagulopathy markers. Patients with coagulopathy in the intervention group had a higher median ISS but were comparable in other

Study: Reference, aim, design, setting	Participants: selection criteria, characteristics	N Participants; Intervention (IG) vs. Control group (CG)	Main outcomes	Assessment: LoE, risk of bias; Conclusions
	Age [y], median (SD)<br>IG: 48 (25–58) vs. CG: 31 (21–55), p=0.34<br>Male, n (%)<br>IG: 18 (90.0) vs. CG: 9 (81.8), p=0.60<br>GCS, median (IQR)<br>IG: 6 (4–7) vs. CG: 6 (5–7), p=0.72<br>ISS, median (IQR)<br>IG: 34 (22–36) vs. CG: 25 (21–25), p=0.08<br>**Non-coagulopathy subgroup**<br>Age [y], median (SD)<br>IG: 42 (20–55) vs. CG: 41 (23–57), p=0.93<br>Male, n (%)<br>IG: 36 (62.1) vs. CG: 16 (59.3), p=0.82<br>GCS, median (IQR)<br>IG: 6 (4–7) vs. CG: 6 (5–7), p=0.65<br>ISS, median (IQR)<br>IG: 25 (17–34) vs. CG: 22 (16–29), p=0.36			baseline characteristics. In addition, there may be a risk for performance bias due to non-blinding of treatment personnel and patients.
**Maekawa (2015)**<br>"Prolonged Mild Therapeutic Hypothermia versus Fever Control with Tight Hemodynamic Monitoring and Slow Rewarming in Patients with Severe Traumatic Brain Injury: A Randomized Controlled Trial." J Neurotrauma 2015; 32(7): 422–429.	**Inclusion criteria**<br>• age 15–69 years<br>• Glasgow Coma Scale (GCS) score of 4–8<br>• ability to initiate cooling within 2 h after the onset of TBI<br>**Exclusion criteria**<br>• good motor response (GCS motor response=6)<br>• SBP <90 mmHg after fluid and vasopressor resuscitation<br>• platelet count <50,000/mm^3	**Participants**<br>N=150 patients randomised, N=148 patients analysed<br>Enrolment stopped before completion of sample size (300 cases) goals, because of concern about shortage of TBI patients (95 cases) and very small differences in neurological outcome between the two groups at the interim analysis in 2005.<br>**Study groups**	**Primary outcome**<br>Poor GOS outcome at 6m§: n/N (%); RR (98% CI)<br>IG: 50/94 (53) vs. CG: 23/48 (48)<br>RR 1.24 (0.62–2.48), p=0.597<br>**Secondary outcomes**<br>Mortality at 6m: n/N (%); RR (98% CI)<br>IG: 33/94 (35) vs. CG: 11/48 (23)<br>RR 1.82 (0.82–4.03), p=0.180<br>Complications, n/N (%)	**Level of evidence**<br>1b<br>**Risk of bias**<br>Selection bias: +<br>Performance bias: ?<br>Attrition bias: +<br>Detection bias: +<br>**Authors' conclusion**

Study: Reference, aim, design, setting	Participants: selection criteria, characteristics	N Participants; Intervention (IG) vs. Control group (CG)	Main outcomes	Assessment: LoE, risk of bias; Conclusions
**Study design**<br>Randomised controlled trial<br>(Brain-Hypothermia (B-HYPO) Study)<br>**Aim of the study**<br>"to compare the neurological outcomes between TH (32–34°C) and fever control (35.5–37°C) for patients with TBI, and to clarify the clinical efficacy of mild therapeutic hypothermia."<br>**Setting**<br>Japan, 2002-2008	• severe pre-existing medical conditions (e.g., liver, kidney, or heart failure, or severe arrhythmia)<br>• acute myocardial infarction, pregnancy<br>• severe alcohol intoxication that prevented assessment of consciousness<br>• penetrating brain injury<br>• epidural hematoma without brain parenchymal injury<br>• core body temperature <30°C<br>**Characteristics**<br>Age [y], mean ± SD<br>IG: 39 ± 19 vs. CG: 39 ± 18, p=0.940<br>Male, n (%)<br>IG: 69 (70) vs. CG: 34 (68), p=0.763<br>ISS, mean ± SD<br>IG: 27 ± 9 vs. CG: 24 ± 7, p=0.037<br>GCS, mean ± SD<br>IG: 5.8 ± 1.4 vs. CG: 5.9 ± 1.3, p=0.513<br>SBP [mmHg], mean ± SD<br>IG: 144 ± 34 vs. CG: 151 ± 39, p=0.222<br>Heart rate [beats/min], mean ± SD<br>IG: 92 ± 28 vs. CG: 88 ± 24, p=0.431	IG: mild therapeutic hypothermia (32.0-34.0°C) (N=99 randomised, N=98 analysed)<br>CG: fever control (35.5-37.0°C) (N=51 randomised, N=50 analysed)<br>Core body temperature was measured by a thermistor coupled to an internal jugular venous catheter. If the catheter could not be inserted, body temperature was measured in another site that was selected in the following order: pulmonary artery, bladder, rectum, and tympanic membrane.<br>Cooling blankets, rapid cold fluid infusion (up to 1000 mL saline, human plasma products, or dextrose-free plasma expanders), and/or cold gastric lavage could be used during the induction phase in both groups. The aim was to achieve the target temperature within 6 h after the onset of TBI. The desired temperature was to be maintained for ≥72 h, mainly using surface cooling blankets, in each group. The patient was rewarmed at a rate of <1°C/day and core body temperature was maintained at <38°C for 7 days after the onset of TBI.	IG: 17/98 (17) vs CG: 1/50 (2), p=significant<br>No significant differences in GOS or mortality subgroups stratified by GCS or age.<br>**§ Severe disability (SD), persistent vegetative state (PVS), or death (D) were defined as poor neurological outcomes, whereas moderate disability (MD) or good recovery (GR) were defined as good neurological outcomes.**	"Prolonged therapeutic hypothermia (≥72 h) for patients with severe TBI together with tight hemodynamic management and slow rewarming (<1.0°C/day) did not improve neurological outcomes or mortality compared with strict fever control. However, the CIs for the primary outcome were wide, and do not exclude either benefit or harm for MTH.<br>Our results may indicate that TH may be harmful or should be applied more carefully in patients with TBI and a GCS of 6–8."<br>**Reviewers' conclusion**<br>This was a well-conducted RCT. There may be a risk for performance bias as treating clinicians were not blinded.<br>The study may have been underpowered to detect clinically significant differences in outcome, because recruitment was stopped for futility at 50% of target.
**Quine (2021)**<br>"Thromboelastography to Assess Coagulopathy in Traumatic Brain Injury	**Inclusion criteria**<br>• Patients between 18 and 60 years<br>• blunt trauma<br>• severe TBI requiring intubation	**Participants**<br>N=20 patients<br>**Study groups**	R time [seconds]: mean ± SD<br>IG: 7.57 ± 2.6 vs. CG: 6.8 ± 1.7, p=0.41<br>α-Angle [degrees]: median (IQR)	**Level of evidence**<br>3b↓<br>**Risk of bias**

Study: Reference, aim, design, setting	Participants: selection criteria, characteristics	N Participants; Intervention (IG) vs. Control group (CG)	Main outcomes	Assessment: LoE, risk of bias; Conclusions
Patients Undergoing Therapeutic Hypothermia." *Therapeutic Hypothermia and Temperature Management* 2021; 11(1): 53-57.<br>**Study design**<br>Prospective cohort study<br>(POLAR-TEG, nested within the POLAR-RCT)<br>**Aim of the study**<br>"Therefore, we conducted a nested cohort substudy of severe TBI patients enrolled in the prophylactic hypothermia to lessen traumatic brain injury (POLAR) trial to compare the TEG coagulation values of patients being treated with therapeutic hypothermia with controls."<br>**Setting**<br>Australia, 2013-2014	• enrolled in the POLAR trial in a single center<br>**Exclusion criteria**<br>• current anticoagulant treatment and<br>• either clinically significant bleeding or<br>• hemodynamic evidence suggestive of bleeding<br>**Characteristics**<br><u>Age [y], mean ± SD</u><br>IG: 29 ± 9 vs. CG: 38 ± 14, p=0.19<br><u>Male, %§</u><br>IG: 80 vs. 90, p=0.53<br><u>GCS, median (IQR)</u><br>IG: 4 (3-6) vs. CG 5 (3-7), p=0.96<br><u>Motor GCS, median (IQR)</u><br>IG: 2 (1-3) vs. CG: 2 (1-4), p=0.58<br><u>AIS-head, median (IQR)</u><br>IG: 5 (4-5) vs. CG: 5 (5-5), p=0.30<br><u>ISS, median (IQR)</u><br>IG:36 (21-50) vs. CG: 30 (28-34), 0.57<br><br>**§only percentage reported**	IG: induced hypothermia at 33°C (N=10)<br>CG: patients tested at 37°C prior to induced hypothermia (N=10)<br>Hypothermia was maintained for 72h. All patients had been returned to normothermia for their second TEG testing at 120 hours.<br>**Co-interventions**<br>Ten patients (five hypothermia and five control) were given blood products during the study. All received packed red blood cells with some receiving additional products. Two patients received 4% albumin solution (one hypothermia and one control) and two received fresh frozen plasma (FFP; both hypothermia). Both patients treated with FFP were given the product before their first TEG.	IG: 69.2 (63.5–69.9) vs. CG: 72.0 (68.7-73.5), p=0.02<br><u>MA [mm]: mean ± SD</u><br>IG: 73.9 ± 3.5 vs. CG: 73.1 ± 4.4, p=0.79<br><u>LY30: median (IQR)</u><br>IG: 0% (0–0.0%) vs. CG: 0.5% (0.1–5.3%), p<0.01<br><u>FF MA [mm]: mean ± SD</u><br>IG: 32.8 ± 10.1 vs. CG: 29.6 ± 9.5, p=0.47<br><u>FLEV [mg/dL]: mean ± SD</u><br>IG: 602 ± 186 vs. CG: 520 ± 178, p=0.33	Selection bias: +<br>Performance bias: ?<br>Attrition bias: +<br>Detection bias: +<br>**Authors' conclusion**<br>"In patients with TBI receiving therapeutic hypothermia to 33°C as part of a randomized trial, we found on TEG that hypothermia was not associated with any clinically significant changes in bleeding tendency or platelet function. A small decrease in clot growth (decreased alpha angle) during hypothermia was within the normal reference range. (…) Hypothermia was associated with decreased fibrinolysis and this finding requires further investigation"<br>**Reviewers' conclusion**<br>**The study should be interpreted with caution due to the very small sample size and missing patient-relevant outcomes. However, it was nested with a RCT and therefore baseline characteristics were comparable within study groups. There may be a risk for performance**

Study: Reference, aim, design, setting	Participants: selection criteria, characteristics	N Participants; Intervention (IG) vs. Control group (CG)	Main outcomes	Assessment: LoE, risk of bias; Conclusions
				**bias as treating clinicians were not blinded.**
**Zhou (2018)**<br>"Influence of phased body temperature management for severe abdominal traumatic patients with hemorrhagic shock." *International Journal of clinical and experimental medicine* 2018; 11(4): 4056-4063.<br>**Study design**<br>Randomised controlled trial<br>**Aim of the study**<br>"In this study, phased body temperature management was employed for severe abdominal trauma patients with hemorrhagic shock in order to provide a clinical basis for its application in clinical settings."<br>**Setting**<br>China, 2015-2016	**Inclusion criteria**<br>• ≤18 y<br>• injury or rupture of abdominal parenchyma and cavity<br>• bleeding in the abdomen<br>• blood pressure lower than 90/60 mmHg<br>• body temperature and blood pH lower than 35°C and 7.3 respectively,<br>• ISS≥16<br>**Exclusion criteria**<br>• patients with fever<br>• abnormal body temperature regulation<br>• thyroid dysfunction<br>• diabetes<br>• unwilling to participate in this study<br>**Characteristics**<br><u>Age [y], mean ± SD</u><br>IG: 43.978 ± 13.965<br>CG: 45.622 ± 14.767, p=0.589<br><u>Males, n</u><br>IG: 32 vs. CG: 30, p=0.649<br><u>ISS, mean ± SD</u><br>IG: 36.022 ± 7.018<br>CG: 36.533 ± 7.694, p=0.743	**Participants**<br>N=90 patients<br>**Study groups**<br>IG: phased management group (N=45)<br>• Room temperature 26-28°C<br>• Wet clothes on patients were removed as soon as possible, then cotton wadding was used to cover the patient to keep them warm.<br>• Intravenous infusion liquid warmed to 37-38°C<br>• blood transfusion temperature 37-38°C<br>• The independent air-conditioning surgery room, bed, and blanket (40°C) were prepared by the Emergency Operating Department in advance<br>CG: routine body temperature treatment. (N=45)<br>• wet clothes were removed and skin was dried. Clean patient' clothes were put on to keep the body dry.<br>• Then the room temperature was maintained between 22 and 24°C.	<u>Mortality: n (%)</u><br>IG: 1 (2.22) vs. CG: 6 (13.33), p=0.049<br><u>Rate of complication: n (%)</u><br>IG: 3 (6.67) vs. CG: 12 (26.67), p=0.011<br><u>Hospital stay [d]: mean ± SD</u><br>IG: 21.689 ± 4.166<br>CG: 27.733 ± 6.166, p=0.000<br><u>Body temperature recovery§ [h]: mean ± SD</u><br>IG: 4.244±1.401 vs. CG: 13.243±3.809, p=0.000<br><u>Lactate clearance§ [h]: mean ± SD</u><br>IG: 12.600±2.683 vs. CG: 31.267±4.059, p=0.000<br><u>Prothrombin time recovery§ [h]: mean ± SD</u><br>IG: 3.578±1.097 vs. 28.844±10.084, p=0.000<br><u>Activated partial thromboplastin time recovery§ [h]: mean ± SD</u><br>IG: 5.222±1.491 vs. CG: 26.956±10.540, p=0.000<br>§The time from admission to recovery of lactic acid, PT and APPT were referred to the lactic acid clearance time, and PT or APTT recovery time.	**Level of evidence**<br>1b<br>**Risk of bias**<br>Selection bias: +<br>Performance bias: ?<br>Attrition bias: ?<br>Detection bias: ?<br>**Authors' conclusion**<br>„Phased body temperature management could effectively improve treatment of severe abdominal traumatic patients with hemorrhagic shock, including correction of the risk of hypothermia, metabolic acidosis, and blood coagulation disorder, reducing rates of complications and death, and shortening the length of the hospital stay"<br>**Reviewers' conclusion**<br>The risk of performance, attrition and detection bias cannot be assessed due to unclear blinding, unclear completeness of data and unclear length of follow up.

+: low risk; –: high risk; ?: unclear risk; adj.: adjusted; APTT: activated partial thromboplastin time; AIS: Abbreviated Injury Scale; BD: base deficit; CG: control group; CI: Confidence Interval; d: days; F MA: Functional Fibrinogen maximum amplitude assay; FFP: fresh frozen plasma; FLEV: functional fibrinogen level; GCS: Glasgow Coma Score; GOS: Glasgow outcome scale; GOS-E: Glasgow outcome scale extended; h: hours; ICU: intensive care unit; IG: intervention group; IQR: Interquartile Range; ISS: injury severity score; ITT: Intention to Treat analysis; L: litres; LoE: level of evidence; LY30:

Study: Reference, aim, design, setting	Participants: selection criteria, characteristics	N Participants; Intervention (IG) vs. Control group (CG)	Main outcomes	Assessment: LoE, risk of bias; Conclusions
percentage of clot lysed after 30 minutes; m: months; MA: maximal amplitude; MAP: mean arterial pressure; min: minutes; MTH: mild therapeutic hypothermia; mmHg: millimetres of mercury; mg: milligrams; PT: prothrombin time; RCT: randomised controlled trial; RR: Relative Risk**;** s: seconds; SBP: systolic blood pressure**;** SD: Standard Deviation**;** SOFA: Sequential Organ Failure Assessment; TBI: traumatic brain injury; TEG: Thromboelastography; y: years				

Acidemia

No studies identified

Hypocalcemia

Study: Reference, aim, design, setting	Participants: selection criteria, characteristics	N Participants; Intervention (IG) vs. Control group (CG)	Main outcomes	Assessment: LoE, risk of bias; Conclusions
**Moore (2020)**<br><br>**"Forgot calcium? Admission ionized-calcium in two civilian randomized controlled trials of prehospital plasma for traumatic hemorrhagic shock."** ***Journal of Trauma and Acute Care Surgery*** **2020; 88(5), 588-596.**<br><br>**Study design**<br>2 Randomised controlled trials<br>(analysis of the COMBAT and PAMPer RCTs)<br><br>**Aim of the study**<br>"We reviewed the experience of two recent prehospital plasma RCTs regarding admission ionized-calcium (i-Ca) blood levels and its impact on survival. We	For inclusion and exclusion criteria see also PAMPer (Sperry 2018) & COMBAT trial (Moore 2018)<br><br>**Inclusion criteria**<br>• adults with traumatic hemorrhagic shock (SBP≤70mmHg or 71–90mmHg + heart rate [HR] ≥108 bpm)<br>• i-Ca measured before Calciumsupplementation<br>• Age>18 years [COMBAT]/ Age 18-90 years [PAMPer]<br><br>**Exclusion criteria**<br>• prisoner status<br>• known pregnancy<br>• isolated penetrating injury to the head<br>• asystole or cardiopulmonary resuscitation before randomization<br>• known objection to blood products<br>• opt-out bracelets or necklaces,<br>• family objection to the patient's enrolment<br>• documented cervical cord injury [PAMPer]<br><br>**Characteristics**	**Participants**<br>N=160 patients<br><br>**Study groups**<br>IG: 2 Units of universal donor (AB) thawed plasma (N=76)<br>CG: normal saline with or without RBCs if required (N=84)<br><br>**Co-interventions**<br>RBC units, Platelet units, cryoprecipitate units, crystalloids, tranexamic acid	**Effect of interventions on hypocalcemia**<br><br><u>Hypocalcemia[§]: n (%)</u><br>IG: 40 (52.6) vs. CG: 30 (35.7),p=0.03<br>Adj. RR (95% CI): 1.48 (1.03-2.12), p=0.03<br><br>**Effect of hypocalcemia on mortality, univariate Cox proportional hazards analysis**<br>Hazard ratio: 1.07 (95% CI 1.02-1.13), p=0.01<br><br>[§]Hypocalcemia: i-Ca ≤1.0 mmol/L	**Level of evidence**<br>2b↓<br><br>**Risk of bias**<br>Selection bias: +<br>Performance bias: –<br>Attrition bias: ?<br>Detection bias: +<br><br>**Authors' conclusion**<br>"Prehospital plasma in civilian trauma is associated with hypocalcemia, which in turn predicts lower survival and massive transfusion. These data underscore the need for explicit calcium supplementation guidelines in prehospital hemotherapy."<br><br>**Reviewers' conclusion**<br>There may be a performance bias because

Study: Reference, aim, design, setting	Participants: selection criteria, characteristics	N Participants; Intervention (IG) vs. Control group (CG)	Main outcomes	Assessment: LoE, risk of bias; Conclusions
hypothesized that prehospital plasma is associated with hypocalcemia, which in turn is associated with lower survival."<br><br>**Setting**<br><br>USA, 2014-2019	<u>Age [y], median (IQR)</u><br>IG: 41 (29–54) vs. CG: 39.5 (26–52), p=0.72<br><br><u>Female, n (%)</u><br>IG: 20 (26.3) vs. CG: 18 (21.4), p=0.58<br><br><u>ISS, median (IQR).</u><br>IG: 22 (15–35) vs. CG: 23 (17–33), p=0.56<br><br><u>GCS, median (IQR).</u><br>IG: 14 (3–15) vs. CG: 12 (3–15), p=0.32			masking of the care team was not possible and because patients in both groups did not receive similar volumes of plasma and placebo. There is a risk of attrition bias because only 63/125 (50.4%) patients in the COMBAT trial had i-Ca measurement and the distribution between study groups is unclear.<br><br>The effect of hypocalcemia on treatment decisions (supplementation) was not investigated. Its effect on patient-relevant outcomes followed a case-control design and no causality was demonstrated.

+: low risk; –: high risk; ?: unclear risk; adj.: adjusted; AIS: Abbreviated Injury Scale; BD: base deficit; CG: control group; CI: Confidence Interval; d: days; GCS: Glasgow Coma Score; h: hours; HR: heart rate; IG: intervention group; IQR: Interquartile Range; ISS: injury severity score; i-Ca: ionized-calcium; L: litres; LoE: level of evidence; m: months; MAP: mean arterial pressure; min: minutes; mmHg: millimetres of mercury; RBC: red blood cells; RCT: randomised controlled trial; RR: Relative Risk; s: seconds; SBP: systolic blood pressure; SD: Standard Deviation**;** y: years

Transfusion / blood products / plasma

Study: Reference, aim, design, setting	Participants: selection criteria, characteristics	N Participants; Intervention (IG) vs. Control group (CG)	Main outcomes	Assessment: LoE, risk of bias; Conclusions
**Akbari (2018)**<br><br>"The effect of fibrinogen concentrate and fresh frozen plasma on the outcome of patients with acute traumatic coagulopathy: a quasi-experimental study." *The*	**Inclusion criteria**<br>• patients with severe blunt multiple trauma (ISS >16)<br>• age ≥18 y<br>• in need of receiving concentrated red blood cells an<br>• with a fibrinogen blood level <200 mg/dl	**Participants**<br>N=90 patients<br><br>**Study groups**<br>Fibrinogen: 2 g fibrinogen (N=30)<br><br>FFP: at least 2 units of FFP was injected (N=30)	<u>Mortality during hospital stay, n (%)</u><br>Fibrinogen: 3 (10.0) vs. FFP: 11 (36.7) vs. CG: 11 (36.7), p=0.029<br><br><u>Blood received in the initial 24h (unit), mean ±SD</u><br>Fibrinogen: 2.04 ± 1.14 vs. FFP: 2.66 ± 0.65 vs. CG: 2.88 ± 0.88, p=0.044<br><br><u>Fluid received in the initial 24h (L), mean ± SD</u>	**Level of evidence**<br>1b<br><br>**Risk of bias**<br>Selection bias: ?<br><br>Performance bias: –<br><br>Attrition bias: +

Study: Reference, aim, design, setting	Participants: selection criteria, characteristics	N Participants; Intervention (IG) vs. Control group (CG)	Main outcomes	Assessment: LoE, risk of bias; Conclusions
American journal of emergency medicine 2018; 36(11): 1947-1950.<br><br>**Study design**<br><br>Randomised controlled trial<br><br>**Aim of the study**<br><br>"The present study is designed with the aim of comparing the outcome of ATC patients receiving fibrinogen and FFP."<br><br>**Setting**<br><br>Iran, 2015-2016	**Exclusion criteria**<br><br>• penetrating trauma, those with ISS <16<br>• patients with known history of liver dysfunction or coagulation disorders<br><br>**Characteristics**<br><br>Age [y], mean ± SD<br><br>Fibrinogen: 34.93 ± 16.94 vs. FFP: 30.63 ± 13.21 vs. CG: 30.63 ± 13.21, p=0.572<br><br>Males, n (%)<br><br>Fibrinogen: 26 (86.7) vs. FFP: 23 (76.7) vs. CG: 25 (83.3), p=0.587<br><br>Consciousness level (GCS), n (%)<br><br>GSC ≥14: Fibrinogen: 2 (6.7) vs. FFP: 3 (10.0) vs. CG: 3 (10.0)<br><br>GSC 8–14: Fibrinogen: 8 (26.7) vs. FFP: 10 (33.3) vs. CG: 16 (53.3)<br><br>GSC <8: Fibrinogen: 20 (66.7) vs. FFP: 17 (56.7) vs. CG: 11 (36.7)<br><br>p=0.204<br><br>Fibrinogen level [mg/dl], mean ± SD<br><br>Fibrinogen: 106.4 ± 24.6 vs. FFP: 120.0 ± 22.4 vs. CG: 123.2 ± 24.4, p=0.018<br><br>ISS, mean ± SD<br><br>Fibrinogen: 19.3 ± 4.4 vs. FFP: 17.2 ± 3.1 vs. CG: 19.0 ± 4.3, p=0.009<br><br>Fibrinogen level (mg/dl), mean ± SD<br><br>Fibrinogen: 106.4 ± 24.6 vs. FFP: 120.0 ± 22.4 vs. CG: 123.2 ± 24.4, p=0.018	CG: no product other than concentrated red blood cells was injected (N=30)<br><br>**Co-interventions**<br><br>Injection of concentrated red blood cells in all patients	Fibrinogen: 3.4 ± 0.8 vs. FFP: 4.0 ± 1.1 vs. CG: 4.1 ± 1.0, p=0.022<br><br>Need for ICU admission, n (%)<br><br>Fibrinogen: 19 (63.3) vs. FFP: 28 (93.3) vs. CG: 22 (73.3), p=0.020<br><br>Need for mechanical ventilation, n (%)<br><br>Fibrinogen: 10 (33.3) vs. FFP: 14 (46.7) vs. CG: 17 (56.7), p=0.191<br><br>Multiple organ failure, n (%)<br><br>Fibrinogen: 2 (6.7) vs. FFP: 8 (26.7) vs. CG: 7 (23.3), p=0.106<br><br>Sepsis, n (%)<br><br>Fibrinogen: 5 (16.6) FFP: 16 (53.3) vs. CG: 4 (13.3), p=0.001<br><br>Duration of hospitalization [d], mean ± SD<br><br>Fibrinogen: 11.0 ± 6.1 vs. FFP: 10.4 ± 8.2 vs. CG: 14.8 ± 7.6, p=0.045<br><br>Thrombosis, n<br><br>Fibrinogen: 0 vs. FFP: 0 vs. CG: 0	Detection bias: +<br><br>**Authors' conclusion**<br><br>"Multiple trauma patients in need of blood transfusion who received fibrinogen along with concentrated red blood cells had a significantly better outcome regarding mortality, sepsis, need for intensive care unit admission, need for receiving packed cells, need for receiving intravenous fluids in the initial 24h, and duration of hospitalization. Although the rate of multiple organ failure and need for mechanical ventilation was lower in these patients, the difference was not statistically significant."<br><br>**Reviewers' conclusion**<br><br>The risk of selection bias is unclear since no information on allocation concealment is given. There may be a risk for performance and detection due to non-blinded treating staff and investigators.
**Bui (2016)**	**Inclusion criteria**	**Participants**	Mortality: n/N (%)	**Level of evidence**

Study: Reference, aim, design, setting	Participants: selection criteria, characteristics	N Participants; Intervention (IG) vs. Control group (CG)	Main outcomes	Assessment: LoE, risk of bias; Conclusions
"The impact of increased plasma ratios in massively transfused trauma patients: a prospective analysis." *European Journal of Trauma and Emergency Surgery* 2016; 42(4): 519-525.<br><br>**Study design**<br><br>Prospective observational study<br><br>**Aim of the study**<br><br>“The aim of our study was to prospectively analyze the effect of increasing ratios of fresh frozen plasma to packed red blood cells on the survival of massively transfused civilian trauma patients. Time elapsed to achieving the target FFP:PRBC ratio and its effect on mortality was also examined. Our hypothesis was that higher plasma to packed red blood cell ratios would improve survival.“<br><br>**Setting**<br><br>USA, 2009-2011	• Trauma patients who require a massive transfusion (defined as ≥10 units of PRBC in ≤24 h)<br><br>**Exclusion criteria**<br><br>• age <18 years<br>• severe traumatic brain injury defined as the presence of head Abbreviated Injury Scale (AIS) score ≥3<br>• pregnancy<br>• death in the emergency department or during the initial operative intervention<br>• not receiving plasma<br><br>**Characteristics**<br><br><u>Age [y],mean ± SD</u><br><br>IG: 34.6 ± 14 vs. CG: 35 ± 18.4, p=0.895<br><br><u>Males , n/N (%)</u><br><br>IG: 49/54 (90.7) vs. CG: 42/49 (85.7), p=0.543<br><br><u>ISS; mean ± SD</u><br><br>IG: 21.1 ± 13.2 vs. CG: 22.3 ± 10.9, p=0.621<br><br><u>ISS >25, n/N (%)</u><br><br>IG: 18/54 (33.33) vs. CG: 20/49 (40.8), p=0.540<br><br><u>GCS ≤8, n/N (%)</u><br><br>IG: 10/54 (18.5) vs. CG: 10/49 (20.4), p=0.801	N=103 patients<br><br>**Study groups**<br><br>IG: FFP:PRBC <1:1.5 (N=54)<br><br>CG: FFP:PRBC ≥1:1.5 (N=49)	IG: 17/54 (31.5) vs. CG: 7/49 (14.3), p=0.042<br><br><u>Hospital length of stay [d]: mean ± SD</u><br><br>IG: 25.5 ± 7.3 vs. CG: 37.2 ± 4.9, p=0.191<br><br><u>Hospital length of stay [d] after exclusion of deaths: mean ± SD</u><br><br>IG: 31.9 ± 5.7 vs. CG: 47.5 ± 7.5, p=0.107<br><br><u>ICU length of stay [d]: mean ± SD</u><br><br>IG: 18.8 ± 5.4 vs. CG: 19.3 ± 2.4, p=0.914<br><br><u>ICU length of stay [d] after exclusion of deaths: mean ± SD</u><br><br>IG: 17.5 ± 3.3 vs. CG: 21.9 ± 3.2, p=0.334<br><br><u>Ventilation days: mean ± SD</u><br><br>IG: 3.7 ± 1.1 vs. CG: 7.9 ± 1.6, p=0.11<br><br><u>Ventilation days after exclusion of deaths: mean ± SD</u><br><br>IG: 5.1 ± 1.3 vs. CG: 8.3 ± 1.6, p=0.14<br><br><u>Change of hematocrit at the end of resuscitation: mean ± SD</u><br><br>IG: −3.20 ± 2.32 vs. CG: −2.83 ± 1.00, p=0.865<br><br><u>Change of INR at the end of resuscitation; mean ± SD</u><br><br>IG: −0.07 ± 0.13 vs. 0.09 ± 0.05, p=0.378<br><br><u>Change of base deficit at the end of resuscitation; mean ± SD</u><br><br>IG: 9.1 ± 1.87 vs. CG: 7.6 ± 0.86, p=0.401<br><br><u>Change of lactate at the end of resuscitation; mean ± SD</u><br><br>IG: −2.92 ± 1.10 vs. CG: −3.29 ± 0.75, p=0.787	2b<br><br>**Risk of bias**<br><br>Selection bias: +<br><br>Performance bias: ?<br><br>Attrition bias: +<br><br>Detection bias: ?<br><br>**Authors’ conclusion**<br><br>“Achieving a ratio of FFP:PRBC ≥1:1.5 after the initial 24 h of resuscitation significantly improves survival in massively transfused trauma patients compared to patients that achieved a ratio <1:1.5.”<br><br>**Reviewers’ conclusion**<br><br>**The risk of performance and detection bias cannot be assessed due to unclear blinding and unclear follow-up time. It should be noted that not only the ratios but also the total volume of PRBCs and plasma transfused was different between study groups.**
**Cardenas (2018)**	**Inclusion criteria**	**Participants**	<u>24h mortality, n (%)</u>	**Level of evidence**

Study: Reference, aim, design, setting	Participants: selection criteria, characteristics	N Participants; Intervention (IG) vs. Control group (CG)	Main outcomes	Assessment: LoE, risk of bias; Conclusions
"Platelet transfusions improve hemostasis and survival in a substudy of the prospective, randomized PROPPR trial. *Blood advances* 2018; 2(14): 1696-1704.<br><br>**Study design**<br>Comperative registry study<br>(secondary analysis of the PROPPR trial)<br><br>**Aim of the study**<br>"The objective of this study was to examine the effect of platelet transfusions on mortality in severely injured trauma patients."<br><br>**Setting**<br>North America, 2012-2013	• ≥15 years of age,<br>• were received directly from the injury scene,<br>• had been transfused with at least 1 unit of blood product within the first hour of arrival or prehospital,<br>• and were predicted to receive a massive transfusion<br>• patients who received only the first cooler of blood products during the randomized treatment phase and received no additional platelet transfusions post–randomized treatment.<br><br>**Exclusion criteria**<br>• transferred from another hospital,<br>• had a lethal traumatic brain injury,<br>• were prisoners,<br>• were pregnant, were <15 years of age,<br>• had received >5 minutes of cardiopulmonary resuscitation,<br>• had a >20% total bodysurface area burn,<br>• had an inhalation injury,<br>• or had >3 units of RBCs transfused<br><br>**Characteristics**<br><u>Age [y], median (IQR)</u><br>IG: 35 (25-50) vs. CG: 35 (26-49.5), p=0.82<br><u>Males, n (%)</u><br>IG: 109 (79.6) vs. CG: 103 (83.1), p=0.53<br><u>ISS, median (IQR)</u><br>IG: 22 (14-34) vs. CG: 21.5 (11-29.5), p=0.15<br><u>Revised Trauma Score, median (IQR)</u>	N=261 patients<br><br>**Study groups**<br>IG: received platelets (1:1:1 group) (N=137)<br>CG: did not receive platelets (1:1:2 group) (N=124)<br><br>**Co-interventions**<br>• RBCs, Plasma, Cryoprecipitate, Colloids, Crystalloids, L<br>• Randomization in PROPPR trial to receive 1:1:1 transfusion ratio of blood products compared with a 1:1:2 ratio (Plasma: Platelets: Red Blood Cells).<br><br>**Adjustments**<br>For adjustment purposes, total plasma received was categorized into 7 strata (0, 1, 2, 3, 4, 5, and ≥6 units). Stratification methods were used for comparing treatment groups, to adjust for different amounts of plasma in these 2 groups.<br>p_{adj} for p-values adj. for plasma volume	IG: 8 (5.8) vs. CG: 21 (16.9), p_{adj}<0.01<br><u>30d mortality, n (%)</u><br>IG: 13 (9.5) vs. CG: 25 (20.2), p_{adj}<0.01<br><u>Time to death [h], median (IQR)</u><br>IG: 13.8 (0.9-69.5) vs. CG: 0.6 (0.3-5.7), p=0.02<br><u>Achieved homostasis, n (%)</u><br>IG: 130 (94.9) vs. CG: 91 (73.4), p_{adj}<0.01<br><u>Hospital free days, median (IQR)</u><br>IG: 13 (0-22) vs. CG: 15 (0-22), p_{adj}=0.77<br><u>Ventilator-free days, median (IQR)</u><br>IG: 28 (23-29) vs. CG: 28 (9-29), p_{adj}=0.03<br><u>ICU-free days, median (IQR)</u><br>IG: 25 (15-27) vs. CG: 25 (7-27), p_{adj}=0.09<br><u>Cause of death (24 h)</u><br>Exsanguination, n (%)<br>IG: 2 (1.5) vs. CG: 16 (12.9), p=0.01<br><u>Traumatic brain injury n (%)</u><br>IG: 4 (2.9) vs. CG: 5 (4.0), p=0.63<br><u>Respiratory, pulmonary contusion, or tension pneumothorax, n (%)</u><br>IG: 0 (0) vs. CG: 0 (0)<br><u>Multiple organ failure, n (%)</u><br>IG: 0 (0) vs. CG: 0 (0)<br><u>Myocardial infarction, n (%)</u><br>IG: 1 (0.7) vs. CG: 1 (0.8), p=0.94<br><u>Pulmonary embolism, n (%)</u><br>IG: 0 (0) vs. CG: 1 (0.8), p=0.32	2b<br><br>**Risk of bias**<br>Selection bias: –<br>Performance bias: ?<br>Attrition bias: +<br>Detection bias: +<br><br>**Authors' conclusion**<br>"In this subgroup analysis of the PROPPR randomized trial, we have shown that transfusion of platelets in bleeding patients is associated with improved early and late survival, improved hemostasis, and reduced number of deaths resulting from exsanguination, without an increase in significant inflammatory complications like acute respiratory distress syndrome, multiorgan failure, and acute kidney injury."<br><br>**Reviewers' conclusion**<br>There might be a risk of performance bias as staff could not be blinded and the care apart from the intervention was not standardized. There is a risk of selection bias as the groups differ in the amount of plasma received. However, adjustments for

<table>
<tr><th>Study: Reference, aim, design, setting</th><th>Participants: selection criteria, characteristics</th><th>N Participants; Intervention (IG) vs. Control group (CG)</th><th>Main outcomes</th><th>Assessment: LoE, risk of bias; Conclusions</th></tr>
<tr><td></td><td>IG: 7.11 (4.09-7.84) vs. CG: 6.90 (4.09-7.84), p=0.28

<u>INR, median (IQR)</u>
IG: 1.20 (1.10-1.32) vs. CG: 1.20 (1.14-1.40), p=0.18</td><td></td><td><u>Cause of 30 day mortality</u>

<u>Exsanguination, n (%)</u>
IG: 2 (1.5) vs. CG: 16 (12.9), p<0.01

<u>Traumatic brain injury, n (%)</u>
IG: 8 (5.8) vs. CG: 9 (7.3), p=0.64

<u>Respiratory, pulmonary contusion, or tension pneumothorax, n (%)</u>
IG: 1 (0.7) vs. CG: 0 (0), p=0.32

<u>Multiple organ failure, n (%)</u>
IG: 0 (0) vs. CG: 1 (0.8), p=0.32

<u>Myocardial infarction, n (%)</u>
IG: 1 (0.7) vs. CG: 1 (0.8), p=0.94

<u>Pulmonary embolism, n (%)</u>
IG: 0 (0) vs. CG:1 (0.8), p=0.32

<u>Systemic inflammatory response syndrome, %</u>
IG: 65.0 vs. CG: 49.2, p=0.01</td><td>this confounder were done to calculate adjusted p-values, showing consistent results.</td></tr>
<tr><td>Chehab (2021)
"Never-Frozen Liquid Plasma Transfusion in Civilian Trauma: A Nationwide Propensity-Matched Analysis." The Journal of Trauma and Acute Care Surgery 2021; 91(1): 200-205.

Study design
Comparative registry study</td><td>Inclusion criteria
• Adult (≥18 years) trauma patients
• receiving early (≤4 hours) plasma transfusions

Exclusion criteria
• received both LQP and FFP within the initial 24-hour period of their presentation
• Transfer patients
• patients with a history of coagulation disorders,
• patients with pre-hospital cardiac arrest

Characteristics (matched cohort)</td><td>Participants
N=321 patients (N=12,958 pre-matching)

Study groups
IG: FFP within 24 hours of ED presentation (N=214)
CG: Never-frozen liquid plasma (LQP) within 24 hours of ED presentation (N=107)

Co-interventions
PRBC, Platelets

Matching criteria (Propensity score matching)
• demographics</td><td><u>24h-mortality: n (%)</u>
IG: 8 (3.7) vs. CG: 3 (2.8), p=0.664

<u>Died in hospital: n (%)</u>
IG: 43 (20.1) vs. CG: 18 (16.8), p=0.481
Based on Cohen’s effect size (h) calculation for proportions, h (0.201, 0.168) = 0.085 for in-hospital mortality

<u>Time to first plasma unit transfusion [min]: median (IQR)</u>
IG: 98 (59-133) vs. CG: 54 (28-79), p<0.001

<u>Major complications: n (%)</u>
IG: 46 (21.5) vs. CG: 17 (15.9), p=0.233</td><td>Level of evidence
2b

Risk of bias
Selection bias: +
Performance bias: ?
Attrition bias: +
Detection bias: +

Authors’ conclusion
“Transfusion of LQP may be as safe and effective as transfusion of FFP for initial resuscitation after trauma.</td></tr>
</table>

Study: Reference, aim, design, setting	Participants: selection criteria, characteristics	N Participants; Intervention (IG) vs. Control group (CG)	Main outcomes	Assessment: LoE, risk of bias; Conclusions
(Trauma Quality Improvement Program database)<br>**Aim of the study**<br>"This study aims to examine outcomes of trauma patients transfused with LQP compared to thawed FFP."<br>**Setting**<br>USA, 2017	<u>Age [y], mean ± SD</u><br>IG: 47±19 vs. CG: 48±19, P=0.641<br><u>Male, n (%)</u><br>IG: 158 (74) vs. CG:77 (72), p=0.721<br><u>GCS, median (IQR)</u><br>IG: 14 (9-15) vs. CG: 14 (8-15), p=0.661<br><u>ISS, median (IQR)</u><br>IG: 27 (22-41) vs. CG: 27 (26-41), p=0.733	• ED vitals<br>• Injury characteristics<br>• surgical intervention for hemorrhage control<br>• blood product transfusions<br>• ACS trauma center verification level	<u>Acute respiratory distress syndrome: n (%)</u><br>IG: 14 (6.5) vs. CG: 2 (1.9), p=0.070<br><u>Unplanned intubation: n (%)</u><br>IG: 12 (5.6) vs. CG: 3 (2.8), p=0.262<br><u>Sepsis: n (%)</u><br>IG: 8 (3.7) vs. CG: 5 (4.7), p=0.689<br><u>Acute kidney injury: n (%)</u><br>IG: 21 (9.8) vs. CG: 5 (5.6), p=0.111<br><u>Deep vein thrombosis: n (%)</u><br>IG: 13 (6.1) vs. CG: 2 (1.9), p=0.092<br><u>Pulmonary embolism: n (%)</u><br>IG: 2 (0.9) vs. CG: 2 (1.9), p=0.477<br><u>Hospital length of stay, median (IQR)</u><br>IG: 12 (6-23) vs. CG: 12 (6-21), p=0.826	The immediate availability and longer shelf life of LQP units alongside the equivalent clinical outcomes in trauma patients receiving either form of plasma makes LQP an important addition to blood banks in trauma centers across the United States."<br>**Reviewers' conclusion**<br>There might be a risk of performance bias due to unclear blinding. One should be aware that the selected (matched) cohort is much smaller than the total cohort fulfilling the inclusion criteria because most patients received FFP.
**De Roulet (2020)**<br>"Group A emergency-release plasma in trauma patients requiring massive transfusion." *Journal of Trauma and Acute Care Surgery* 2020; 89(6): 1061-1067.<br>**Study design**<br>comparative registry trial<br>(secondary analysis of the PROPPR trial)<br>**Aim of the study**	**Inclusion criteria**<br>• ≥15 years of age,<br>• were received directly from the injury scene,<br>• had been transfused with at least 1 unit of blood product within the first hour of arrival or prehospital,<br>• and were predicted to receive a massive transfusion<br>• at least one unit of ERP<br>**Exclusion criteria**<br>• transferred from another hospital,<br>• had a lethal traumatic brain injury,<br>• were prisoners,<br>• were pregnant, were <15 years of age,	**Participants**<br>N=584 patients<br>**Study groups**<br>IG: group A ERP (N=122)<br>CG: group AB ERP (N=462)<br>Emergency-release plasma (ERP) was defined as plasma delivered and infused before the patient's ABO group being known.<br>**Co-interventions**<br>RBC, Plasma, Platelets, and Crytalloids<br>• Randomization in PROPPR trial to receive 1:1:1 transfusion ratio of blood products	<u>Mortality at 30 days after admission: n (%)</u><br>IG: 30 (24.6) vs. CG: 111 (24.0), p=0.90<br>Adj. HR (95% CI)§:: 1.15 (0.91–1.45)<br><u>Nonfatal complication: n (%)</u><br>IG: 112 (91.8) vs. CG: 370 (80.1), p=0.002<br>Adj. HR (95% CI)§: 1.24 (0.87-1.77)<br><u>Systemic inflammatory response syndrome: n (%)</u><br>IG: 93 (76.2) vs. CG: 297 (64.3), p=0.01<br>Adj. HR (95% CI)§: 1.45 (0.89-2.97)<br><u>Infection: n (%)</u><br>IG: 56 (45.9) vs. CG: 201 (43.5), p=0.64<br>Adj. HR (95% CI)§: 1 (0.74-1.38)	**Level of evidence**<br>2b<br>**Risk of bias**<br>Selection bias: –<br>Performance bias: ?<br>Attrition bias: +<br>Detection bias: +<br>**Authors' conclusion**<br>**"We conclude that the use of ERP is common in patients requiring massive transfusion. Providing group A ERP can facilitate**

Study: Reference, aim, design, setting	Participants: selection criteria, characteristics	N Participants; Intervention (IG) vs. Control group (CG)	Main outcomes	Assessment: LoE, risk of bias; Conclusions
"This secondary analysis of the Pragmatic, Randomized, Optimal Platelet and Plasma Ratios trial examined whether exposure to group A emergency-release plasma (ERP) was noninferior to group AB ERP. We also examined patients whose blood groups were compatible with group A ERP versus patients whose blood groups were incompatible with group A ERP."<br>**Setting**<br>North America, 2012-2013	• had received >5 minutes of cardiopulmonary resuscitation,<br>• had a >20% total body surface area burn,<br>• had an inhalation injury,<br>• or had >3 units of RBCs transfused<br>**Characteristics**<br><u>Age [y], median (IQR)</u><br>IG: 32 (24–51) vs. CG: 35 (25–51), p=0.62<br><u>Male, n (%)</u><br>IG: 102 (83.6) vs. CG:362 (78.4), p=0.20<br><u>ISS, median (IQR)</u><br>IG: 25 (17–34) vs. CG: 29 (18–41), p=0.02<br><u>SBP [mmHg], mean ± SD</u><br>IG: 112.7 ± 33.8 vs. CG: 101.7 ± 31.6, p=0.001<br><u>Blunt injury mechanism, n (%)</u><br>55 (45.1) vs. 261 (56.5), p=0.02	compared with a 1:1:2 ratio (Plasma: Platelets: Red Blood Cells).<br>**Covariates for multivariate model**<br>• Age<br>• ISS>25<br>• GCS <9<br>• 3-hour RBC transfusion<br>• and 3-hour crystalloid infusion<br>• Variables not acting as a confounder and with p>0.05 were excluded from the final model.	<u>Acute kidney injury / acute renal failure: n (%)</u><br>IG: 34 (27.9) vs. CG: 108 (23.4), p=0.30<br>Adj. HR (95% CI)§: 1.11 (0.65-1.94)<br><u>Acute respiratory distress syndrome / acute lung injury: n (%)</u><br>IG: 33 (27.1) vs. CG: 84 (18.2), p=0.03<br>Adj. HR (95% CI)§: 1.41 (0.83-2.4)<br><u>Venous thromboembolism: n (%)</u><br>IG: 12 (9.8) vs. CG: 79 (17.1), p=0.05<br><u>Thrombolytic events: HR (95% CI)§</u><br>Multivariate analysis: 0.52 (0.31–0.90)<br><u>Deep vein thrombosis: n (%)</u><br>IG: 3 (2.5) vs. IG: 41 (8.9)<br><u>Pulmonary embolism: n (%)</u><br>IG: 5 (4.1) vs. IG: 37 (8.0)<br><u>Myocardial infarction: n (%)</u><br>IG: 1 (0.8) vs. IG: 1 (0.2)<br><u>Cerebral vascular accidents: n (%)</u><br>IG: 4 (3.2) vs. CG: 16 (3.5)<br><br>§HR: IG vs. CG	**the early and balanced resuscitation of patients requiring massive transfusion at trauma centers worldwide. (…) Group A ERP is an acceptable option for patients requiring massive transfusion, especially if group AB ERP is not readily available."**<br>**Reviewers' conclusion**<br>There is a risk of selection bias because of unbalanced ISS and SPR but multivariate analyses controlled for confounding. There might be a risk of performance bias due to unknown blinding.
**Innerhofer 2017**<br>"Reversal of trauma-induced coagulopathy using first-line coagulation factor concentrates or fresh frozen plasma (RETIC): a single-centre, parallel-group, open-label, randomised trial". *The*	**Inclusion criteria**<br>• Adult patients (aged 18–80 years)<br>• trauma with Injury Severity Score (ISS) >15<br>• admitted to trauma center<br>• clinical signs or risk of substantial haemorrhage	**Participants**<br>N=100 patients (N=94 in modified ITT analysis)<br>**Study groups**<br>IG: CFC, coagulation factor concentrates (N=52, N=50 in modified ITT analysis)	**NOTE**: ORs and differences were calculated CG vs. IG!<br>**Primary outcome**<br><u>Multiple Organ Failure: n (%), OR (95% CI)</u><br>IG: 25 (50) vs. CG: 29 (66)<br>OR = 1.92 (0.78 - 4.86), p=0.15	**Level of evidence**<br>1b<br>**Risk of bias**<br>Selection bias: +<br>Performance bias: +<br>Attrition bias: +

Study: Reference, aim, design, setting	Participants: selection criteria, characteristics	N Participants; Intervention (IG) vs. Control group (CG)	Main outcomes	Assessment: LoE, risk of bias; Conclusions
Lancet Haematology 2017; 4: e258–71.<br>**Study design**<br>Randomised controlled trial<br>(RETIC trial)<br>**Aim of the study**<br>"The aim of the study was to compare the efficacy of FFP and CFC for reversal of coagulopathy, blood loss-associated transfusion requirements, and the development of multiple organ failure as an overall clinical outcome parameter. "<br>**Setting**<br>Austria, 2012-2016	• screened for trauma-induced coagulopathy (defined as abnormally low fibrin polymerisation as measured with bedside rotational thromboelastometry (ROTEM) using the FibTEM assay (FibA10 <9 mm or prolonged initiation of coagulation in the ExTEM assay (ExCT >90 s)<br>**Exclusion criteria**<br>• injuries incompatible with survival<br>• cardiopulmonary resuscitation on the scene<br>• isolated brain injury<br>• burn injury<br>• avalanche injury<br>• prehospital coagulation therapy other than tranexamic acid<br>**Characteristics**<br><u>Age [y], (median, IQR)</u><br>IG: 42.5 (27.3 - 50.5)<br>CG: 42.5 (24 - 56)<br><u>Sex, n (%)</u><br>IG: 38 (76%) male, 12 (24%) female<br>CG: 32 (73%) male, 12 (27%) female<br><u>ISS (median, IQR)</u><br>IG: 35 (29 - 42)<br>CG: 30 (24 - 45)<br><u>GCS (median, IQR)</u><br>IG: 12 (9 - 15)<br>CG: 11 (7 - 15)<br><u>Lactate (mmol/L, median, IQR)</u><br>IG: 2.22 (1.55 – 3.22)<br>CG: 2.28 (1.64 – 3.03)	CG: FFP, fresh frozen plasma (N=48, N=44 in modified ITT analysis)<br>The trial was stopped early as the preplanned interim analysis showed a significant difference in treatment failure combined with an increased risk of massive transfusion in patients in the FFP group.	**Other outcomes**<br><u>In-hospital mortality: n (%), OR (95% CI)</u><br>IG: 5 (10) vs. CG: 2 (5)<br>OR = 0.43 (0.04 – 2.82), p=0.44<br><u>Ventilator-free days: median (IQR), difference</u><br>IG: 23 (13 – 27) vs. CG: 21 (13 – 27)<br>Difference = 0 (-2 to 3), p=0.99<br><u>Length of hospital stay [d]: median (IQR) difference</u><br>IG: 28 (18 - 28) vs. CG: 27 (16 - 28)<br>Difference = 0 (0 to 1), p=0.61<br><u>Reversal of coagulopathy after single-dose study drug (OR, 95% CI)</u><br>IG vs. CG: OR = OR 8.22 (3.06 – 23.78) (p<0.0001)<br><u>Massive Transfusion within 24 hours: n (%), OR (95% CI)</u><br>IG: 6 (12) vs. CG: 13 (30)<br>OR = 3.04 (0.95 to 10.87), p=0.042	Detection bias: +<br>**Authors' conclusion**<br>"CFC is superior to FFP. However, the trial was terminated early after randomization of 100 patients, as the a-priori planned interim analysis showed an unacceptably high incidence of treatment failure and increased risk for massive transfusion for patients randomly allocated to the FFP group"<br>**Reviewers' conclusion**<br>The study is of high quality. However, one should be aware that physicians were not blinded to the intervention as the difference in the process of study drug administration makes masking impossible.

Study: Reference, aim, design, setting	Participants: selection criteria, characteristics	N Participants; Intervention (IG) vs. Control group (CG)	Main outcomes	Assessment: LoE, risk of bias; Conclusions
	<u>INR (median, IQR)</u><br>IG: 1.3 (1.1 – 1.4)<br>CG: 1.3 (1.2 – 1.5)<br><u>Fibrinogen (mg/dL, median, IQR)</u><br>IG: 196.5 (138.5 - 218.8)<br>CG: 177.0 (140.5 - 222.3)			
**Jones (2017)**<br>"Injury severity, sex, and transfusion volume, but not transfusion ratio, predict inflammatory complications after traumatic injury. *Heart & Lung* 2017; 46(2): 114-119.<br>**Study design**<br>Comparative registry trial<br>(Glue Grant Inflammation and the Host Response to Injury Trauma-Related Database)<br>**Aim of the study**<br>"To evaluate the relationship among blood component ratios (1:1 vs other for PRBC:FFP and PRBC:PLT) and inflammatory complications (primary outcome) in patients with major trauma"<br>**Setting**<br>USA, 2003-2009	**Inclusion criteria**<br>• aged 18-65 years<br>• received blood component transfusion within the first 24 h following hospital admission for trauma,<br>• and were severely injured (New Injury Severity Score [NISS] ≥15)<br>**Exclusion criteria**<br>• died within the 24 h following ED admission, as they were unlikely to develop inflammatory complications in that timeframe<br>**Characteristics**<br><u>Age [y], mean ± SD</u><br>39 ±14<br><u>Male, n (%)</u><br>1040 (68)<br><u>New Injury Severity Score, mean ±SD</u><br>39 ±13	**Participants**<br>N=643 patients in analysis of 1,538 patients in total<br>**Study groups**<br>IG: PRBC:FFP and PRBC:PLT ratios close to 1 (between 0.5-1.5) (N=n.r.)<br>CG: ratios other than 0.5-1.5 (N=n.r.)<br>§Number of patients per study group missing<br>**Covariates for multivariable Cox model**<br>• 24h total unit of PRBC and PLT<br>• Comorbid burden<br>• Age<br>• Sex<br>• NISS	<u>Development of inflammatory complications during hospitalization: adj. HR (95% CI)</u><br>**PRBC:FFP**<br>1.068 (0.900-1.267), p=0.451<br>**PRBC:PLT**<br>0.970 (0.809-1.164), p=0.746	**Level of evidence**<br>2b<br>**Risk of bias**<br>Selection bias: +<br>Performance bias: ?<br>Attrition bias: –<br>Detection bias: +<br>**Authors' conclusion**<br>"In conclusion, we found no relationship between the ratio of blood components transfused in the first 24 h following hospital admission and inflammatory complication development in patients with major trauma."<br>**Reviewers' conclusion**<br>The results of the study need to be interpreted with caution due to a large proportion of patients with missing data (Attrition bias). The risk of performance bias is unclear.

<table>
<tr><th>Study: Reference, aim, design, setting</th><th>Participants: selection criteria, characteristics</th><th>N Participants; Intervention (IG) vs. Control group (CG)</th><th>Main outcomes</th><th>Assessment: LoE, risk of bias; Conclusions</th></tr>
<tr><td>Jones (2014)
"Increased mortality in adult trauma patients transfused with blood components compared with whole blood.” Journal of trauma nursing 2014; 21(1): 22-29.
Study design
Comparative registry trial
(2009 National Trauma databank)
Aim of the study
“The aim of this study was to examine the association of type of blood transfusion, whole blood or blood components, with mortality in adult major trauma patients.”
Setting
USA, 2009</td><td>Inclusion criteria
• Aged 14-45 years
• ISS >25
• admitted to the hospital after care in the ED
• received blood transfusion, either whole blood or blood components, as part of their emergency care
Exclusion criteria
• dead on arrival to the emergency department or
• were discharged to home after ED care and not admitted to the hospital
• age>45 years
Characteristics
<u>Age [y], mean ± SD</u>
IG: 27 ± 8 vs. CG: 29 ± 8, p=0.01
<u>Male, n (%)</u>
IG: 69 (83) vs. CG: 1184 (71), p=0.02
<u>ISS, mean ±SD</u>
IG: 39 ± 17 vs. CG: 35 ± 13, p=0.13</td><td>Participants
N=1,745 patients
Study groups
IG: whole blood transfusion (N=83)
CG: blood components transfusion (PRBCs and PLTs in combination) (N=1662)
Covariates for logistic regression
• Age
• Gender
• Ethnicity
• ISS
• Emergency medical system transfer time
• Transfers from another facility</td><td>Unadj. outcomes
<u>Mortality during hospital stay: n (%)</u>
IG: 17 (21) vs. CG: 429 (26), p=0.27
Adj. outcomes
<u>Mortality during hospital stay: adj. OR (95% CI)</u>
CG vs. IG: 3.164 (1.314 – 7.618), p=0.010</td><td>Level of evidence
2b
Risk of bias
Selection bias: ?
Performance bias: ?
Attrition bias: +
Detection bias: +
Authors’ conclusion
“We found that transfusion of whole blood rather than blood components produced superior survival in adult trauma patients from the NTDB.”
Reviewers’ conclusion
Although results were controlled for confounding factors, the results of the study need to be interpreted with caution due to a possible selection bias caused by unbalanced basic characteristics between study groups. The risk of performance bias is unclear.</td></tr>
<tr><td>Kutcher 2014
“The Natural History and Effect of Resuscitation Ratio on Coagulation After Trauma - A Prospective Cohort Study“. Annals of</td><td>Inclusion criteria
• Adult patients requiring highest-level trauma activation and subsequent intensive care unit (ICU) admission, prespecified by physiologic (at least one pre-hospital or hospital SBP <90, heart rate >110, or Glasgow Coma</td><td>Participants
N=143 patients
Study groups
IG: Low ratio RBC:FFP (RBC:FFP ≤1.5:1) (N=91)</td><td><u>Mortality (%)</u>
IG: 42.9 vs. CG: 55.8, p=0.165
<u>In-Hospital Mortality, Hazard Ratio (95% CI)</u>
IG: HR 1.661 (0.943 – 2.925), p=0.079
CG: HR 3.402 (1.693 - 6.833), p=0.001</td><td>Level of evidence
2b
Risk of bias
Selection bias: –
Performance bias: ?</td></tr>
</table>

Study: Reference, aim, design, setting	Participants: selection criteria, characteristics	N Participants; Intervention (IG) vs. Control group (CG)	Main outcomes	Assessment: LoE, risk of bias; Conclusions
Surgery 2014; 260(6): 1103–1111.<br><br>**Study design**<br>Prospective cohort study<br><br>**Aim of the study**<br>"The aim of the study was to investigate the natural history of coagulation factor perturbation after injury, and identify longitudinal differences in clotting factor repletion by RBC:FFP transfusion ratio."<br><br>**Setting**<br>USA, 2005-2011	Score 8) or anatomic criteria (penetrating torso trauma or evidence of high-energy blunt trauma)<br>• also patients who died in the operating room or emergency department prior to ICU admission<br><br>**Exclusion criteria**<br>• age <18 years<br>• incarceration<br>• pregnancy<br>• transfer from another hospital<br>• administration of >2 liters of crystalloid prior to initial blood draw<br>• Patients on warfarin<br>• Patients possessing a preexisting bleeding diathesis at the time of injury<br><br>**Characteristics**<br><br><u>Age [y], mean ± SD</u><br>IG: 38.7 ± 18.3, p=0.253<br>CG: 42.8 ± 21.5<br><br><u>Sex (n.r.)</u><br><br><u>ISS, mean ± SD</u><br>IG: 32.7 ± 16.2, p=0.164<br>CG: 36.6 ± 15.5<br><br><u>GCS, median (IQR)</u><br>IG: 6 (3 - 15), p=0.029<br>CG: 12 (4 - 15)<br><br><u>RBC units / 24h, median (IQR)</u><br>IG: 7 (4 - 14), p=0.001<br>CG: 10 (7 - 22)<br><br><u>FFP units / 24h, median (IQR)</u>	CG: High ratio RBC:FFP (RBC:FFP >1.5:1) (N=52)<br><br>**Note**<br>The original study population consisted of 336 patients who were divided into the groups "transfused" (N=143) and "non-transfused" (N=193). Those who received transfusions were further divided into the two groups "Low ratio" and "High ratio" RBC:FFP.	<u>Total hospital days, median (IQR)</u><br>IG: 11 (2 - 32) vs. CG: 6.5 (1.5 - 24), p=0.135<br><br><u>Longitudinal factor levels</u><br><br><u>Prothrombin time up to 72h [sec], median (IQR):</u><br>*0h:* IG: 16.7 (14.5 – 21.2) vs. CG: 16.2 (14.6 – 20.6)<br>*6h:* IG: 15.3 (14.2 - 17.6) vs. CG: 16.2 (15.1 --18.3)<br>*12h:* IG: 15.6 (14.6 - 16.9) vs. CG: 15.9 (15.0 - 17.0)<br>*24h:* IG: 16.9 (15.5 - 19.1) vs. CG: 17.0 (15.8 - 17.7)<br>*48h:* IG: 16.2 (14.9 - 18.8) vs. CG: 17.6 (15.6 - 18.8)<br>*72h:* IG: 15.5 (14.1 - 17.8) vs. CG: 15.6 (15.2 - 17.3)<br><br><u>Activated partial thromboplastin time up to 72h [sec], median (IQR):</u><br>*0h:* IG: 32.5 (27.5 – 42.2) vs. CG: 32.5 (27.6 – 41.8)<br>*6h:* IG: 33.2 (28.6 - 36.1)* vs. CG: 36.6 (32.5 - 41.7)*<br>*12h:* IG: 33.7 (30.8 - 38.7)* vs. CG: 35.9 (32.8 - 40.4)*<br>*24h:* IG: 38.2 (34.8 - 43.1) vs. CG: 39.8 (36.0 - 46.4)<br>*48h:* IG: 39.2 (36.4 - 47.4) vs. CG: 47.0 (38.6 - 49.5)<br>*72h:* IG: 38.5 (32.9 - 43.8) vs. CG: 41.5 (39.4 - 45.8)<br><br><u>Fibrinogen up to 72h [ng/mL], median (IQR):</u><br>*0h:* IG: 157 (106 – 200) vs. CG: 139 (125 – 205)<br>*6h:* IG: 142 (111 – 205) vs. CG: 139 (125 – 205)<br>*12h:* IG: 180 (133 – 234) vs. CG: 183 (157 – 230)<br>*24h:* IG: 288 (162 – 362) vs. CG: 258 (213 – 291)<br>*48h:* IG: 325 (264 – 514) vs. CG: 547 (530 – 566)<br>*72h:* IG: 494 (395 – 559) vs. CG: 668 (566 – 780)	Attrition bias: +<br>Detection bias: +<br><br>**Authors' conclusion**<br>"[…] targeting ratios of RBC:FFP <=1.5:1 leads to earlier correction of PT and PTT, and earlier and prolonged repletion of specific clotting factor deficits compared to higher ratio transfusion strategies."<br><br>**Reviewers' conclusion**<br>There is a risk of selection bias because basic characteristic were not equal and the initial decision to transfuse and the specific array of blood products transfused were entirely at the discretion of the attending trauma surgeon. There might also be a risk of performance bias.

Study: Reference, aim, design, setting	Participants: selection criteria, characteristics	N Participants; Intervention (IG) vs. Control group (CG)	Main outcomes	Assessment: LoE, risk of bias; Conclusions
	IG: 7 (4 - 12), p=0.160<br>CG: 4.5 (3 - 11)		Factor II up to 72h [%], mean ± SD:<br>*0h:* IG: 66.2 ± 21.3* vs. CG: 57.7 ± 18.3*<br>*6h:* IG: 61.6 ± 17.1 vs. CG: 54.1 ± 14.9<br>*12h:* IG: 64.4 ± 17.5 vs. CG: 59.4 ± 12.1<br>*24h:* IG: 64.0 ± 18.5 vs. CG: 60.6 ± 12.4<br>*48h:* IG: 66.8 ± 20.2 vs. CG: 61.8 ± 14.2<br>*72h:* IG: 72.2 ± 14.4 vs. CG: 70.2 ± 15.4<br>Factor V up to 72h [%], mean ± SD:<br>*0h:* IG: 38.7 ± 24.7 vs. CG: 38.0 ± 26.8<br>*6h:* IG: 43.2 ± 20.7* vs. CG: 33.3 ± 16.2*<br>*12h:* IG: 43.7 ± 19.3 vs. CG: 39.7 ± 16.7<br>*24h:* IG: 45.3 ± 23.4 vs. CG: 43.2 ± 17.6<br>*48h:* IG: 58.8 ± 29.0 vs. CG: 43.2 ± 17.6<br>*72h:* IG: 79.6 ± 44.2 vs. CG: 61.0 ± 23.0<br>Factor VII up to 72h [%], mean ± SD:<br>*0h:* IG: 72.3 ± 31.7 vs. CG: 73.9 ± 46.3<br>*6h:* IG: 107.0 ± 77.6 vs. CG: 86.3 ± 51.8<br>*12h:* IG: 100.3 ± 69.7 vs. CG: 101.1 ± 59.4<br>*24h:* IG: 48.2 ± 22.9 vs. CG: 61.1 ± 36.3<br>*48h:* IG: 61.8 ± 34.6 vs. CG: 52.9 ± 19.<br>*72h:* IG: 79.6 ± 29.8 vs. CG78.7 ± 32.7<br>Factor VIII up to 72h [%], mean ± SD:<br>*0h:* IG: 188.1 ± 128.5* vs. CG: 266.6 ± 172.0*<br>*6h:* IG: 115.3 ± 80.3 vs. CG: 106.4 ± 77.4<br>*12h:* IG: 105.2 ± 62.2 vs. CG: 106.2 ± 78.5<br>*24h:* IG: 110.6 ± 51.0 vs. CG: 112.5 ± 43.7<br>*48h:* IG: 179.3 ± 147.7 vs. CG: 114.1 ± 53.4	

Study: Reference, aim, design, setting	Participants: selection criteria, characteristics	N Participants; Intervention (IG) vs. Control group (CG)	Main outcomes	Assessment: LoE, risk of bias; Conclusions
			72h: IG: 192.9 ± 94.6 vs. CG: 152.6 ± 78.1<br><br><u>Factor IX up to 72h [%], mean ± SD:</u><br>*0h:* IG: 101.2 ± 40.6 vs. CG: 96.5 ± 44.2<br>*6h:* IG: 116.3 ± 39.4* vs. CG: 86.5 ± 29.7*<br>*12h:* IG: 114.0 ± 35.4* vs. CG: 100.7 ± 18.5*<br>*24h:* IG: 114.6 ± 36.7 vs. CG: 107.1 ± 25.1<br>*48h:* IG: 151.6 ± 45.6 vs. CG: 142.9 ± 36.1<br>*72h:* IG: 184.4 ± 46.2 vs. CG: 179.0 ± 53.1<br><br><u>Factor X up to 72h [%], mean ± SD:</u><br>*0h:* IG: 64.1 ± 23.9 vs. CG: 61.0 ± 23.1<br>*6h:* IG: 72.6 ± 33.4* vs. CG: 54.7 ± 16.9*<br>*12h:* IG: 65.0 ± 19.9 vs. CG: 62.3 ± 20.9<br>*24h:* IG: 60.7 ± 17.9 vs. CG: 58.2 ± 10.4<br>*48h:* IG: 66.2 ± 16.8 vs. CG: 57.9 ± 12.9<br>*72h:* IG: 72.6 ± 15.7 vs. CG: 72.7 ± 13.0<br><br><u>Antithrombin III up to 72h [%], mean ± SD:</u><br>*0h:* IG: 75.5 ± 24.9 vs. CG: 73.8 ± 23.4<br>*6h:* IG: 67.6 ± 18.9 vs. CG: 67.2 ± 19.7<br>*12h:* IG: 73.8 ± 17.6 vs. CG: 74.7 ± 16.7<br>*24h:* IG: 69.8 ± 16.7 vs. CG: 71.9 ± 20.0<br>*48h:* IG: 66.8 ± 21.0 vs. CG: 61.2 ± 18.9<br>*72h:* IG: 72.4 ± 22.6 vs. CG: 66.8 ± 24.1<br><br><u>Protein C up to 72h [%], mean ± SD:</u><br>*0h:* IG: 77.4 ± 29.0 vs. CG: 79.5 ± 27.1<br>*6h:* IG: 66.8 ± 17.0 vs. CG: 63.9 ± 17.8<br>*12h:* IG: 72.0 ± 18.5 vs. CG: 69.7 ± 12.9<br>*24h:* IG: 64.5 ± 22.4 vs. CG: 64.4 ± 15.1	

Study: Reference, aim, design, setting	Participants: selection criteria, characteristics	N Participants; Intervention (IG) vs. Control group (CG)	Main outcomes	Assessment: LoE, risk of bias; Conclusions
			48h: IG: 65.9 ± 21.6 vs. CG: 57.7 ± 11.9<br>*72h:* IG: 73.4 ± 30.4 vs. CG: 61.3 ± 18.6<br>Activated Protein C up to 72h [ng/mL], median (IQR):<br>*0h:* IG: 15.3 (5.4 – 49.5) vs. CG: 13.7 (3.5 – 51.2)<br>*6h:* IG: 2.8 (0.7 – 4.7) vs. CG: 1.4 (0.6 – 2.7)<br>*12h:* IG: 1.2 (0.6 – 2.5) vs. CG: 1.0 (0.4 – 2.3)<br>*24h:* IG: 1.0 (0.5 – 1.9) vs. CG: 0.5 (0.0 – 1.4)<br>*48h:* IG: 0.5 (0.3 – 1.7) vs. CG: 1.2 (1.1 – 1.4)<br>*72h:* IG: 0.8 (0.6 – 2.4) vs. CG: 1.2 (0.5 – 13.4)<br>D-dimer up to 72h [mcg/mL], median (IQR):<br>*0h:* IG: 6.7 (2.4 – 9.8) vs. CG: 7.5 (3.8 – 12.0)<br>*6h:* IG: 6.4 (3.2 – 9.4) vs. CG: 6.5 (2.6 – 23.0)<br>*12h:* IG: 6.8 (3.8 – 10.6) vs. CG: 7.4 (4.2 – 14.6)<br>*24h:* IG: 7.6 (3.9 – 11.4) vs. CG: 7.4 (3.8 – 10.6)<br>*48h:* IG: 3.3 (2.1 – 5.0) vs. CG: 4.7 (2.8 – 6.7)<br>*72h:* IG: 3.7 (3.1 – 6.1) vs. CG: 4.4 (3.2 – 6.7)<br>Tissue plasminogen activator up to 72h [ng/mL], median (IQR):<br>*0h:* IG: 31.0 (9.0 – 46.7) vs. CG: 25.0 (14.7 – 38.3)<br>*6h:* IG: 15.3 (11.2 – 22.6) vs. CG: 12.5 (8.0 – 15.6)<br>*12h:* IG: 13.5 (11.4 – 18.0) vs. CG: 10.2 (8.4 – 14.3)<br>*24h:* IG: 9.8 (6.1 – 15.3 vs. CG: 6.8 (5.8 – 11.1)<br>*48h:* IG: 4.3 vs. CG: 5.0 (4.6 – 5.3)<br>*72h:* IG: - vs. CG: 4.5 (3.5 – 5.4)<br>Plasminogen activator inhibitor-1 up to 72h [ng/mL], median (IQR):<br>*0h:* IG: 25.3 (11.8 – 30.2) vs. CG: 25.7 (12.4 – 38.4)	

Study: Reference, aim, design, setting	Participants: selection criteria, characteristics	N Participants; Intervention (IG) vs. Control group (CG)	Main outcomes	Assessment: LoE, risk of bias; Conclusions
			6h: IG: 146.9 (138.7 – 372.4) vs. CG: 125.1 (49.5 – 132.6)<br>*12h:* IG: 142.4 (134.2 – 278.9) vs. CG: 141.6 (84.1 – 251.4)<br>*24h:* IG: 86.1 (57.7 – 124.6) vs. CG: 64.6 (43.8 – 143.4)<br>*48h:* IG: 25.5 (8.3 – 33.5) vs. CG: 27.1 (19.1 – 33.2)<br>*72h:* IG: - vs. CG: 19.2 (13.8 – 24.6)<br>* p<0.05 for comparison between „low" and "high ratio" patients at each time point	
**Nederpelt (2019)**<br>"Fresh Frozen Plasma-To-Packed Red Blood Cell Ratio and Mortality in Traumatic Hemorrhage: Nationwide Analysis of 4,427 Patients", *Journal of the American College of Surgeons* 2019; 230(6): 893-901<br>**Study design**<br>Comparative registry study<br>(TQIP Database)<br>**Aim of the study**<br>"The aim of the study was to investigate the association between different FFP to pRBC transfusion ratios and 24-hour mortality in trauma patients requiring mass transfusion with an attempt to statistically	**Inclusion criteria**<br>• all trauma patients 18 years or older<br>• transfused ≥10 pRBCs and ≥1 FFP within 24 hours<br>**Exclusion criteria**<br>• all transfer patients<br>• patients with incorrect or missing transfusion data<br>• patients who died in the emergency room<br>• patients whose FFP:pRBC ratio was different in the first 4 vs. 24 hours of hospitalization<br>• Patients with an FFP:RBC ratio of exactly 1:1.5, 1:2.5<br>**Characteristics**<br><u>Age [y], mean ± SD</u><br>1:1: 39 ± 18, p=0.091<br>1:2: 42 ± 19<br>1:3: 40 ± 19<br>1:4: 41 ± 19<br>1:5: 40 ± 21	**Participants**<br>N=4,427 patients<br>**Study groups**<br>1:1: (N=1392) (31.44%)<br>1:2: (N=1801) (40.68%)<br>1:3: (N=492) (11.11%)<br>1:4: (N=190) (4.29%)<br>1:5: (N=79) (1.78%)<br>1:6: (N=51) (1.15%)<br>1:6+: (N=422) (9.53%)<br>Patients were assigned to seven FFP:pRBC cohorts from ratios 1:1 to 1:6, and 1:6+<br>**Covariates**<br>• ACS trauma center level<br>• patient demographics<br>• comorbidities<br>• ED presentation<br>• injury characteristics<br>• surgeries and procedures performed in the first 24h	**Primary outcome**<br><u>24-hour mortality, n/N (%), adj. OR (95% CI)</u><br>1:1: 395/1392 (28.38) (Reference), p<0.0001<br>1:2: 598/1801 (33.20), OR 1.23 (1.02 to 1.48)<br>1:3: 200/492 (40.65), OR 1.62 (1.24 to 2.11)<br>1:4: 91/190 (47.89), OR 2.11 (1.42 to 3.13)<br>1:5: 49/79 (62.03), OR 4.11 (2.31 to 7.31)<br>1:6: 29/51 (56.86), OR 2.98 (2.31 to 6.13)<br>1:6+: 135/422 (31.99), OR 1.25 (0.94 to 1.67)<br>**Other outcomes**<br><u>In-hospital mortality, n/N (%), adj. OR (95% CI)</u><br>1:1: 675/1392 (48.49) (Reference), p<0.0001<br>1:2: 888/1801 ((49.31), OR 1.16 (0.90 to 1.49)<br>1:3: 266/492 (54.07), OR 1.51 (1.03 to 2.20)<br>1:4: 108/190 ((56.84), OR 1.52 (0.92 to 2.49)<br>1:5: 56/79 (70.89), OR 3.64 (1.84 to 7.22)<br>1:6: 36/51 (70.59), OR 2.65 (1.14 to 6.13)<br>1:6+: 203/422 (48.10), OR 1.43 (1.14 to 6.13)<br><u>ICU length of stay, mean ± SD</u><br>1:1: 12 ± 13, p<0.0001<br>1:2: 12 ± 15<br>1:3: 10 ± 11<br>1:4: 10 ± 13	**Level of evidence**<br>2b<br>**Risk of bias**<br>Selection bias: +<br>Performance bias: ?<br>Attrition bias: +<br>Detection bias: +<br>**Authors' conclusion**<br>"[…] an FFP:pRBC ratio of 1:1 was associated with the lowest odds or 24-hour mortality. Our data suggests that the correct interpretation of the well-known PROPPR trial is perhaps that the 1:1:1 ratio is indeed superior, and that its failure to show that superiority might have been due to a type II error and a smaller patient sample than necessary. […] we suggest the use of a 1:1

Study: Reference, aim, design, setting	Participants: selection criteria, characteristics	N Participants; Intervention (IG) vs. Control group (CG)	Main outcomes	Assessment: LoE, risk of bias; Conclusions
address delay and survival bias."<br><br>**Setting**<br><br>All TQIP participating hospitals (USA), 2013-2016	1:6: 41 ± 21<br>1:6+: 41 ± 18<br><br><u>Females, n/N (%)</u><br><br>1:1: 287/1392 (20.63)<br>1:2: 375/1801 (20.82)<br>1:3: 106/492 (21.54)<br>1:4: 48/190 (25.26)<br>1:5: 19/79 (24.05)<br>1:6: 15/51 (29.41)<br>1:6+: 76/422 (18.01)<br><br><u>ISS, median (IQR)</u><br><br>1:1: 33 (22-43), p=0.000<br>1:2: 29 (22-42)<br>1:3: 29 (22-41)<br>1:4: 29 (20-42)<br>1:5: 29 (25-38)<br>1:6: 27 (22-43)<br>1:6+: 27 (17-38)<br><br><u>GCS: median (IQR)</u><br><br>1:1: 8 (3-15), p=0.128<br>1:2: 10 (3-15)<br>1:3: 7 (3-14)<br>1:4: 8 (3-15)<br>1:5: 3 (3-13)<br>1:6: 6.5 (3-14)<br>1:6+: 8 (3-15)		1:5: 7 ± 7<br>1:6: 10 ± 12<br>1:6+: 10 ± 11	FFP:pRBC ratio rather than a 1:2 ratio in the massively transfused trauma patient"<br><br>**Reviewers' conclusion**<br><br>The results of the study need to be interpreted carefully due to unclear performance bias. However, a large sample size and adjustment for various confounders indicate reliable results.
**Pusateri (2020)**<br><br>"Association of Prehospital Plasma Transfusion With Survival in Trauma Patients With Hemorrhagic Shock When Transport Times Are Longer Than 20 Minutes: A Post Hoc Analysis of the PAMPer and COMBAT	**For inclusion and exclusion criteria see PAMPer (Sperry 2018) & COMBAT trial (Moore 2018)**<br><br>**Characteristics**<br><br><u>Age, years, median (IQR)</u><br><br>Total: 42 (27-52)<br><br>IG: 43 (29-56)<br>CG: 42 (26-57)	**Participants**<br><br>N=626 patients<br><br>(N=125 COMBAT, N=501 PAMPer)<br><br>**Study groups**<br><br>IG: prehospital plasma (N=297)<br><br>CG: standard care (crystalloid) (N=329)	**Influence of prehospital transport time** (subgroup analysis of PAMPer & COMBAT)<br><br><u>Mortality (28 days, 1ary outcome): HR (95% CI)[§]</u><br><br>≤20 min transport time<br><br>IG vs. CG: 1.71 (0.70-4.16), p=0.24<br><br>>20 min transport time<br><br>IG vs. CG: 0.56 (0.40-0.80) p=0.001<br><br><u>Mortality (24 h): Hazard ratio (95% CI)[§]</u>	**Level of evidence**<br><br>2b↓<br><br>**Risk of bias**<br><br>Selection bias: +<br><br>Performance bias: –<br><br>Attrition bias: +<br><br>Detection bias: +

Study: Reference, aim, design, setting	Participants: selection criteria, characteristics	N Participants; Intervention (IG) vs. Control group (CG)	Main outcomes	Assessment: LoE, risk of bias; Conclusions
Clinical Trials". *JAMA Surgery* 2020; 155(2): e195085.<br><br>**Study design**<br>Post-hoc subgroup analysis of data from 2 RCTs (PAMPer & COMBAT)<br><br>**Aim of the study**<br>"Examine the combined data set to address the post hoc hypothesis that the benefits of prehospital administration of plasma are influenced by prehospital transport time."<br><br>**Setting**<br>USA, 2014-2019	<u>Males, n (%)</u><br>Total: 467 (84.6)<br>IG: 216 (72.7)<br>CG: 251 (76.3)<br><br><u>ISS, median (IQR)</u><br>Total: 22 (12-34)<br>IG: 22 (12-34)<br>CG: 22 (12-33)		≤20 min transport time<br>IG vs. CG: 1.89 (0.65-5.40), p=0.25<br>>20 min transport time<br>IG vs. CG: 0.53 (0.34-0.82) p=0.004<br>§Patients with event not reported per group, analyses adjusted for age and ISS	**Authors' conclusion**<br>"These data suggest that prehospital plasma is associated with a survival benefit when transport times are longer than 20 minutes and that the benefit-risk ratio is favorable for use of prehospital plasma."<br><br>**Reviewers' conclusion**<br>This is post-hoc subgroup analysis of harmonized data from the PAMPer and COMBAT trial. There may be a performance bias because masking of the care team was not possible and because patients in both groups did not receive similar volumes of plasma and placebo.<br><br>Downgraded due to post-hoc analysis.
**Reitz (2020)**<br>"Prehospital plasma in injured patients is associated with survival principally in blunt injury: Results from two randomized prehospital plasma trials". *The Journal of Trauma and Acute Care Surgery* 2020; 88(1): 33-41<br><br>**Study design**	For inclusion and exclusion criteria see PAMPer (Sperry 2018) & COMBAT trial (Moore 2018)<br><br>**Characteristics**<br><u>Age, years, median (IQR)</u><br>Blunt trauma: 45 (28-61)<br>Penetrating trauma: 35 (26-49) (p<0.001)<br><br><u>Males, n (%)</u><br>Blunt trauma: 326 (70.1)<br>Penetrating trauma: 141 (87.6) (p<0.001)	**Participants**<br>N=626 patients (N=501 PAMPer, N=125 COMBAT)<br><br>**Study groups**<br>IG: prehospital plasma (N=not reported)<br>CG: standard care (crystalloid) (N=not reported)<br><br>**Subgroup analysis mechanism of injury**	**Mechanism of injury (subgroup analysis of PAMPer & COMBAT)**<br><br><u>28-day mortality (primary endpoint): n (%)</u><br>Blunt trauma<br>IG: 50 (23.5) vs CG: 86 (34.1), p=0.012<br>Multivariate Cox-hazard regression HR (95% CI):<br>HR: 0.68 (0.472-0.965), p=0.031<br>Penetrating trauma<br>IG: 12 (14.3) vs. CG. 8 (10.4), p=0.454	**Level of evidence**<br>2b↓<br><br>**Risk of bias**<br>Selection bias: +<br>Performance bias: –<br>Attrition bias: +<br>Detection bias: +<br><br>**Authors' conclusion**

Study: Reference, aim, design, setting	Participants: selection criteria, characteristics	N Participants; Intervention (IG) vs. Control group (CG)	Main outcomes	Assessment: LoE, risk of bias; Conclusions
Post-hoc subgroup analysis of data from 2 randomized controlled trials (PAMPer & COMBAT)<br><br>**Aim of the study**<br><br>“Our overall objective was to characterize prehospital plasma outcomes across mechanism of injury using harmonized data obtained from these two recently completed prehospital plasma clinical trials. We hypothesized that the safety and beneficial effects of prehospital plasma would be consistent across blunt and penetrating mechanism of injury.”<br><br>**Setting**<br><br>USA, 2014-2019	<u>ISS, median (IQR)</u><br><br>Total: 22 (12-34)<br><br>Blunt trauma: 24 (17-34)<br>Penetrating trauma: 14 (6-25) (p<0.001)<br><br><u>GCS: median (IQR)</u><br><br>Total: 6 (3-15)<br><br>Blunt trauma: 3 (3-15)<br>Penetrating trauma: 14 (3-15) (p=0.004)	<u>Blunt</u>: n=465, 75%, (including 10 suffering from blunt and penetrating trauma) (n=406 PAMPer, n=59 COMBAT)<br><br><u>Penetrating</u>: n=161, 25% (n=95 PAMPer, n=66 COMBAT)	Multivariate Cox-hazard regression HR (95% CI):<br><br>HR: 1.16 (0.430 – 3.103), p=0.775<br><br><u>24h mortality: n (%)</u><br><br>Blunt trauma<br><br>IG: 29 (15.2) vs. CG: 58 (25.8), p=0.010<br><br>Multivariate Cox-hazard regression HR (95% CI):<br><br>HR: 0.59 (0.370-0.947), p=0.029<br><br>Penetrating trauma<br><br>IG: 8 (10.4) vs. CG: 11 (13.23), p=0.595<br><br>Multivariate Cox-hazard regression HR (95% CI):<br><br>HR: 1.16 (0.430 – 3.103), p=0.775	“A survival benefit associated with prehospital plasma at 24 hours and 28 days exists primarily in blunt injured patients with no benefit shown in penetrating trauma patients”<br><br>**Reviewers’ conclusion**<br><br>This is post-hoc subgroup analysis of harmonized data from the PAMPer and COMBAT trial. There may be a performance bias because masking of the care team was not possible and because patients in both groups did not receive similar volumes of plasma and placebo.<br><br>**Downgraded due to post-hoc analysis.**
**Roquet (2019)**<br><br>**“Association of Early, High Plasma-to–Red Blood Cell Transfusion Ratio With Mortality in Adults With Severe Bleeding After Trauma.”** ***JAMA network open*** **2019; 2(9), e1912076.**<br><br>**Study design**	**Inclusion criteria**<br><br>• patients with severe bleeding after trauma (defined as ≥4 PRBC units ≤6 h after admission)<br>• patients who died of hemorrhagic causes before receiving 4 units of PRBCs<br><br>**Exclusion criteria**<br><br>• died on the scene<br>• died during hospital transfer without any blood transfusion	**Participants**<br><br>N=897 patients<br><br>**Study groups**<br><br>IG: high ratio, FFP-to-PRBC ratio of more than 1:1.5 (N=506)<br><br>CG: low ratio, FFP-to-PRBC ratio of 1:1.5 or less (N=391)<br><br>**Covariates for regression model (30d mortality)**<br><br>• age	**Primary outcome**<br><br><u>30-d mortality: adj. HR (95% CI)</u><br><br>After multiple imputation (n=897)<br><br>IG: 0.74 (0.58-0.94), p=0.01<br><br>Complete cases (n=594)<br><br>IG: 0.57 (0.33-0.97), p=0.04<br><br>**Secondary outcomes**<br><br><u>6 h mortality: adj. HR (95% CI)</u><br><br>After multiple imputation (n=897)	**Level of evidence**<br><br>2b<br><br>**Risk of bias**<br><br>Selection bias: +<br><br>Performance bias: ?<br><br>Attrition bias:+<br><br>Detection bias: +<br><br>**Authors’ conclusion**

Study: Reference, aim, design, setting	Participants: selection criteria, characteristics	N Participants; Intervention (IG) vs. Control group (CG)	Main outcomes	Assessment: LoE, risk of bias; Conclusions
Comparative registry trial (Traumabase)<br><br>**Aim of the study**<br><br>"To study the association of an early, high FFP-to-PRBC ratio with all-cause 30-day mortality in patients with severe bleeding after trauma."<br><br>**Setting**<br><br>France, 2012-2017	**Characteristics**<br><br>Age [y], median (IQR)<br><br>IG: 36 (25-54) vs. CG: 39 (26-54). p=0.10<br><br>Male, n (%)<br><br>IG: 351 (69.4) vs. CG: 288 (73.7), p=0.18<br><br>ISS, median (IQR)<br><br>IG: 34 (22-45) vs. CG: 34 (21-50), p=0.62<br><br>GCS <9, n (%)<br><br>IG: 190 (37.5) vs. CG: 132 (33.8), p=0.26	• gender<br>• anticoagulant or antiplatelet medication history<br>• trauma characteristics including intentionality and mechanism<br>• Injury Severity Score (ISS)<br>• Simplified Acute Physiology Score II (SAPS II)<br>• initial Glasgow Coma Scale (GCS)<br>• lowest body temperature<br>• prehospital tracheal intubation<br>• prehospital cardiac arrest or cardiac arrest at hospital admission<br>• prehospital lactatemia<br>• prehospital capillary hemoglobin measurement<br>• prehospital clinical parameters incl. lowest SBP and highest heart rate<br>• prothrombin time (PT) at admission<br>• surgery (orthopaedic, vascular, thoracic, abdominal surgery, neurosurgery)<br>• interventional radiology<br>• fluid replacement ≤6 h (colloids & crystalloids)<br>• need for vasopressor<br>• tranexamic acid administration<br>• platelet and fibrinogen concentrate transfusion	0.91 (0.61-1.35), p=0.60<br><br>Complete cases (n=594)<br><br>0.71 (0.35-1.43), p=0.30<br><br>24 h mortality, n/N (%)<br><br>IG: 91/506 (18.0) vs. 113/391 (28.9)<br><br>HR (95% CI): 0.79 (0.58-1.06), p=0.11<br><br>Length of ICU stay [d], median (IQR)<br><br>IG: 16 (8-32) vs. CG: 11 (4-24)<br><br>OR (95% CI): 1.25 (0.28-5.40), p=0.31	"In this study, a transfusion strategy based on an early FFP-to-PRBC ratio of more than 1:1.5 was associated with decreased 30-day mortality among patients with severe bleeding after trauma. Further studies are needed to identify optimal, personalized, and dynamic transfusion strategies to help clinicians adjust the transfusion strategy in real time."<br><br>**Reviewers' conclusion**<br><br>There may be a risk for performance bias. Missing data might lead to a residual risk of attrition bias although the results remained significant across multiple imputation methods.<br><br>About a third of patients who died in the low-ratio group did not receive any FFP.
**Shea (2020)**<br><br>"The use of low-titer group O whole blood is independently associated with improved survival compared to component therapy in adults with severe traumatic hemorrhage." *Transfusion* 2020; 60, S2-S9.	**Inclusion criteria**<br><br>• ≤18 years of age<br>• traumatic injury<br>• MTP activation<br><br>**Exclusion criteria**<br><br>• no exclusion criteria reported<br><br>**Characteristics**	**Participants**<br><br>N=86 patients<br><br>**Study groups**<br><br>IG: low-titer group O whole blood (LTOWB) (N=44 analysed)<br><br>CG: Component therapy (N=42 analysed)<br><br>**Co-interventions**	**Adj. mortality**<br><br>by multivariate logistic regression<br><br>24 h mortality: OR (95% CI)<br><br>0.81 (0.69-0.96), p=0.017<br><br>28 day mortality: OR (95% CI)<br><br>0.81 (0.65-1.02), p=0.059<br><br>by Cox regression	**Level of evidence**<br><br>2b<br><br>**Risk of bias**<br><br>Selection bias: +<br><br>Performance bias: –<br><br>Attrition bias: +<br><br>Detection bias: +

Study: Reference, aim, design, setting	Participants: selection criteria, characteristics	N Participants; Intervention (IG) vs. Control group (CG)	Main outcomes	Assessment: LoE, risk of bias; Conclusions
**Study design**<br>Prospective observational study<br>**Aim of the study**<br>"We hypothesized that the use of LTOWB is independently associated with improved 24-hour mortality and 28-day mortality, reduces the total amount of blood products transfused in the first 72 hours after injury, and does not increase 72-hour multiple organ dysfunction scores (MODS) compared to the exclusive use of CT in adult patients with traumatic injury requiring massive transfusion protocol (MTP) activation."<br>**Setting**<br>USA, 2018-2019<br>CG: Aug 2018-Dec 2018<br>IG: Dec 2018- May 2019	Age [y], median (IQR)<br>IG: 32 (28-32) vs. CG: 28 (22-38), p=0.158<br>Male, n (%)<br>IG: 35 (80) vs. CG: 31 (74), p=0.708<br>ISS, median (IQR)<br>IG: 18 (10-29) vs. CG: 22 (17-33), p=0.162<br>GCS, median (IQR)<br>IG: 11 (3-15) vs. CG: 12 (3-15), p=0.796	Normal saline, lactated Ringer´s, crystalloid fluids, albumin, TXA, Calcium<br>**Covariates for regression analysis**<br>• maximum clot firmness (MCF)<br>• other variables (LTOWB use, Glasgow Coma Scale (GCS) score, ISS, PT) were removed by backwards stepwise selection to arrive at a fitted model	24 h mortality: HR (95% CI)<br>0.15 (0.03-0.49), p=0.001<br>28 day mortality: HR (95% CI)<br>0.30 (0.14-0.65), p=0.002<br>**Unadjusted mortality**<br>Unadj. 24-h mortality: n/N (%)<br>IG: 7/44 (16) vs. CG: 9/42 (21), p=0.518<br>Unadj. 28-day mortality: n/N (%)<br>IG: 14/44 (44) vs. CG: 14/42 (33), p=0.86<br>**Unadj. outcomes: Multiple organ dysfunction scores (MODS)**<br>total MODs, median (IQR)<br>IG: 4 (0-7) vs. CG: 4 (2-7), p=0.913<br>Respiratory, median (IQR)<br>IG: 0 (0-0) vs. CG: 0 (0-0), p=0.386<br>Renal, median (IQR)<br>IG: 0 (0-1) vs. CG: 1 (0-1), p=0.461<br>Hepatic, median (IQR)<br>IG: 0 (0-0) vs. CG: 0 (0-0), p=0.838<br>Cardiologic, median (IQR)<br>IG: 0 (0-0) vs. CG: 0 (0-0), p=NA<br>Hematologic, median (IQR)<br>IG: 0 (0-2) vs. CG: 1 (0-2), p=0.838<br>Neurologic, median (IQR)<br>IG: 3 (0-4) vs. CG: 4 (2-7), p=0.913	**Authors' conclusion**<br>"The use of LTOWB is independently associated with improved 24-hour and 28-day survival, and does not increase organ dysfunction at 72 hours. Use of LTOWB most impacted survival of patients with reduced clot firmness (MCF ≤60 mm). Collectively, these data support the clinical use and continued study of LTOWB for hemostatic resuscitation."<br>**Reviewers' conclusion**<br>The study involved consecutive time periods for the study groups, leading to a risk for performance bias.
**Stanworth (2016)**	**Inclusion criteria**<br>• adult injured patients ≥16 years	**Participants**<br>N=442 patients	Mortality at 3h: n/N (%)<br>IG: 12/67 (18) vs. CG: 7/141 (5)	**Level of evidence**<br>2b

Study: Reference, aim, design, setting	Participants: selection criteria, characteristics	N Participants; Intervention (IG) vs. Control group (CG)	Main outcomes	Assessment: LoE, risk of bias; Conclusions
Mortality from trauma haemorrhage and opportunities for improvement in transfusion practice. *BJS* 2016; 103(4): 357-365.<br><br>**Study design**<br><br>Prospective observational study<br><br>(TARN)<br><br>**Aim of the study**<br><br>"The aim of this study was to describe the prevalence, patterns of blood use and outcomes of major haemorrhage in trauma."<br><br>**Setting**<br><br>UK, 2009-2011	• admitted to the 22 trauma receiving hospitals who fulfilled the TARN inclusion criteria: injuries that result in a hospital stay of 72 h or more, transfer for specialist or intensive care, or patient death.<br>• received at least 4 units of packed red blood cells (PRBCs) in the first 24 h of admission<br>• with activation of the massive haemorrhage protocol (MHP). The rationale for requiring MHP activation was to identify the more severely injured bleeding patients.<br><br>**Exclusion criteria**<br><br>• Patients transferred from another hospital<br>• Patients who died within 1 h were excluded from this analysis<br><br>**Characteristics**<br><br><u>Age [y], median (IQR)</u><br><br>18 (26-54)<br><br><u>Male, n (%)</u><br><br>326 (73,8)<br><br><u>ISS, median (IQR)</u><br><br>27 (17-41)<br><br>At 24h: IG: 29 (19-44) vs. 28 (17-43)<br><br><u>GCS, median (IQR) *</u><br><br>14 (6-15)<br><br>*data available for 391 patients	**Study groups**<br><br>IG: FFP : PRBC ratio <1:2 (N=67 within 3h, N=92 within 24h)<br><br>CG: FFP : PRBC ratio ≥1:2 (N=141 within 3h, N=206 within 24h)	<u>Mortality at 24h: n/N (%)</u><br><br>IG: 26/92 (28) vs. CG: 25/206 (12.1)	**Risk of bias**<br><br>Selection bias: –<br><br>Performance bias: ?<br><br>Attrition bias: ?<br><br>Detection bias: +<br><br>**Authors' conclusion**<br><br>"Higher FFP : PRBC ratios were associated with lower mortality rates, with no apparent difference in injury severity or physiology between the groups."<br><br>**Reviewers' conclusion**<br><br>The study results need to be interpreted with cause because of the risk of selection bias and unclear risk of performance and attrition bias.<br><br>A substantial proportion of patients who died in the low-ratio group did not receive any FFP.
**Stevens (2017)**	**Inclusion criteria**<br><br>• Trauma patients	**Participants**<br><br>N=1,536 patients	**Adj. outcomes**<br><br><u>Adj. overall mortality at 28 d: OR (95% CI)</u>	**Level of evidence**<br><br>2b

Study: Reference, aim, design, setting	Participants: selection criteria, characteristics	N Participants; Intervention (IG) vs. Control group (CG)	Main outcomes	Assessment: LoE, risk of bias; Conclusions
"Incompatible type A plasma transfusion in patients requiring massive transfusion protocol: outcomes of an Eastern Association for the Surgery of Trauma multicenter study." *Journal of Trauma and Acute Care Surgery* 2017; 83(1): 25-29.<br><br>**Study design**<br>Comparative registry trial<br>(trauma registry, blood bank, and medical record data, including centers that use type A plasma for trauma resuscitation)<br><br>**Aim of the study**<br>"The goal of this study is to determine outcomes for trauma patients who received incompatible plasma transfusions as part of a massive transfusion protocol (MTP)"<br><br>**Setting**<br>USA, 2012-2016	• Initiation of Massive transfusion protocol<br><br>**Exclusion criteria**<br>• patients less than 15 years of age (all participating institutions were adult trauma centers),<br>• patients for whom a blood type was never identified (e.g., due to early demise),<br>• and patients with preferences limiting blood transfusion<br><br>**Characteristics**<br><u>Age [y], median</u><br>IG: 37 vs. CG: 36, p=0.802<br><u>Male, %</u><br>IG: 75.2% vs. 82,5%, p=0.076<br><u>ISS, median</u><br>IG. 25 vs. CG: 25, p=0.303<br><u>Penetrating injuries, %</u><br>IG: 36.1 vs. CG: 48.3, p=0.010	**Study groups**<br>IG: patients receiving compatible Type A plasma (N=1,416)<br>CG: patients receiving incompatible Type A plasma (N=120)<br><br>**Co-interventions**<br>Transfusion of RBC, plasma<br><br>**Covariates for logistic regression**<br>• reporting center<br>• age<br>• injury severity<br>• method of injury<br>• number of units of red blood cells transfused at 4 hours (as a proxy for bleeding rate)	1.00 (0.65–1.51), p=0.981<br><br>**Unadj. outcomes**<br><u>Mortality at 6h: % (N=1317)</u><br>IG: 15.2 vs. CG: 16.5, p=0.775<br><u>Mortality at 24h: % (N=1314)</u><br>IG: 22.8 vs. CG: 25.2, p=0.544<br><u>Mortality at 7d: % (N=1314)</u><br>IG: 31.6 vs. CG: 35.0, p=0.509<br><u>Mortality at 28d or at discharge: % (N=1,536)</u><br>IG: 34.8 vs. 38.3, p=0.486<br><u>Adj. morbidity: OR (95% CI)</u><br>1.12 (0.74–1.70), p=0.581<br><u>Morbidity (any of the following six morbidities): %</u><br>IG: 27.8 vs. CG: 34.2, p=0.140<br><u>ARDS: %</u><br>IG: 7.6 vs. CG: 5.8, p=0.589<br><u>Pulmonary embolism or Deep vein thrombosis: %</u><br>IG: 7.2 vs. CG: 9.2, p=0.464<br><u>Pneumonia: %</u><br>IG: 15.3 vs. CG: 19.2, p=0.294<br><u>Sepsis: %</u><br>IG: 7.6 vs. CG: 5.8, p=0.589<br><u>Acute renal failure: %</u><br>IG: 7.9 vs. CG: 8.3, p=0.860<br><u>Transfusion related acute lung injury (TRALI): n</u><br>IG: 2 vs. CG: 0, p=1.000<br><u>Vent days</u>	**Risk of bias**<br>Selection bias: +<br>Performance bias: ?<br>Attrition bias: +<br>Detection bias: +<br><br>**Authors' conclusion**<br>"These data support the safety of incompatible type A plasma transfusions as part of a MTP in trauma centers, although perhaps at a cost of more overall plasma transfusions."<br><br>**Reviewers' conclusion**<br>The risk of performance bias is unclear. Unadjusted values need to be interpreted with caution due to variations in co-interventions.

Study: Reference, aim, design, setting	Participants: selection criteria, characteristics	N Participants; Intervention (IG) vs. Control group (CG)	Main outcomes	Assessment: LoE, risk of bias; Conclusions
			IG: 2 vs. CG: 3.5, p=0.415<br><br><u>ICU LOS [d]</u><br>IG: 5 vs. CG: 6, p=0.715<br><br><u>Hospital LOS [d]</u><br>IG: 9 vs. CG: 12, p=0.514<br><br><u>Number of operations, n</u><br>IG: 2 vs. CG: 2, p=0.328	
**Zeeshan (2019)**<br><br>**Four-factor prothrombin complex concentrate is associated with improved survival in trauma-related hemorrhage: a nationwide propensity-matched analysis. *Journal of Trauma and Acute Care Surgery* 2019; 87(2): 274-281.**<br><br>**Study design**<br>Comparative registry trial<br>(American College of Surgeons-Trauma Quality Improvement Program database)<br><br>**Aim of the study**<br>**"The aim of our study was to evaluate outcomes in severely injured trauma patients who received 4-PCC + FFP compared to FPP alone"**<br><br>**Setting**	**Inclusion criteria**<br>• adult trauma patients (age ≥18 years)<br>• presented to trauma center<br>• Received either FFP alone or 4-PCC + FFP for initial resuscitation in the ED.<br><br>**Exclusion criteria**<br>• documented bleeding disorders,<br>• chronic liver disease,<br>• history of preinjury anticoagulants use<br>• received PCC alone (without FFP).<br>• Patients who were not managed at trauma centers using ICD-10 codes<br><br>**Characteristics (matched cohort)**<br><u>Age [y], mean ± SD</u><br>IG: 51 ± 19 vs. CG: 50 ± 21, p=0.28,<br><br><u>Male, %</u><br>IG: 69.5 vs. CG: 70.3, p=0.28<br><br><u>GCS, median (IQR)</u><br>IG: 14 (12–15) vs. CG: 14 (12–15), p=0.18<br><br><u>ISS, median (IQR)</u><br>IG: 27 (19–35) vs. CG: 27 (20–37), p=0.28	**Participants**<br>N=468 (N=118,970 before matching)<br><br>**Study groups**<br>IG: 4-PCC + FFP (N=234)<br>CG: FFP alone (N=234)<br><br>**Matching criteria**<br>• Age<br>• Sex<br>• Race<br>• Body mass index<br>• SBP<br>• HR<br>• GCS<br>• Time to ignition oh therapy<br>• Mechanism of injury<br>• ISS<br>• Head-AIS<br>• Chest-AIS<br>• Abdominal-AIS<br>• Spine-AIS<br>• Extremity-AIS<br>• Comorbidities<br>• preinjury antiplatelet use<br>• level of trauma center	<u>ED mortality: n (%)</u><br>IG: 5 (2.1) vs. CG: 6 (2.5) vs., p=0.28<br><br><u>Overall in-hospital mortality: n (%)</u><br>IG: 41 (17.5) vs. CG: 65 (27.7), p=0.01<br><br><u>Acute kidney injury: n (%)</u><br>IG: 5 (2.1) vs. CG: 17 (7.3), p=0.001<br><br><u>Acute respiratory syndrome: n (%)</u><br>IG: 3 (1.3) vs. CG: 11 (4.7), p=0.04<br><br><u>Deep Venous Thrombosis: n (%)</u><br>IG: 8 (3.4) vs. CG: 13 (5.5), p=0.11<br><br><u>Pulmonary Embolism: n (%)</u><br>IG: 3 (1.3) vs. CG: 4 (1.7), p=0.33<br><br><u>Hospital stay [d]: median (IQR)</u><br>IG: 5 (2–8) vs. CG: 8 (3–11), p=0.03<br><br><u>ICU stay [d]: median (IQR)</u><br>IG: 1 (1–3) vs. CG: 1 (1–2), p=0.19<br><br><u>Skilled nursing facility/ Rehabilitation disposition: n (%)</u><br>IG: 92 (39.8) vs. CG: 90 (38.4), p=0.21	**Level of evidence**<br>2b↓<br><br>**Risk of bias**<br>Selection bias: +<br>Performance bias: ?<br>Attrition bias: +<br>Detection bias: +<br><br>**Authors' conclusion**<br>"Our study demonstrates that compared to FFP alone, the use of 4-factor PCC as an adjunct to FFP is associated with improved survival and reduction in transfusion requirements without increasing the risk of venous thromboembolic complications."<br><br>**Reviewers' conclusion**<br>The results need to be interpreted with caution due to the retrospective nature of the study and unclear risk for

<table>
<tr><th>Study: Reference, aim, design, setting</th><th>Participants: selection criteria, characteristics</th><th>N Participants; Intervention (IG) vs. Control group (CG)</th><th>Main outcomes</th><th>Assessment: LoE, risk of bias; Conclusions</th></tr>
<tr><td>USA; 2015-2016</td><td></td><td></td><td></td><td>performance bias. No match was found for around 40% of patients receiving 4-PCC because of systematic differences prior to matching.</td></tr>
<tr><td>Zhang (2019)

“Low-dose, early fresh frozen plasma transfusion therapy after severe trauma brain injury: a clinical, prospective, randomized, controlled study.” World neurosurgery 2019; 132: e21-e27.

Study design
Randomised controlled trial

Aim of the study
“To investigate role of Low-dose, Early Fresh frozen plasma Transfusion (LEFT) therapy in preventing perioperative coagulopathy and improving longterm outcome after severe traumatic brain injury (TBI).”

Setting
China 2018</td><td>Inclusion criteria
adult patients who underwent emergency craniotomy evacuation of hematomas and decompressive hemicraniectomy for subdural hematoma
preoperative GCS score of 3-8
subdural hematoma with TCDB ≥4
<3 hours after admission
Exclusion criteria
severe cardiac dysfunction
pulmonary dysfunction
hepatic dysfunction
renal dysfunction
history of dementia
history of prior central nervous system disease
history of coagulating disorders
anticoagulant drug usage (e.g., aspirin, clopidogrel, warfarin)
Characteristics (analysed patients)

<u>Age [y], mean ± SD</u>
IG: 65.7 ± 10.4 vs. CG: 64.1 ± 7.7, p=0.548

<u>Females, n$</u>
IG: 7 vs. CG: 6, p=0.510

<u>GCS, mean ± SD</u>
IG: 4.7 ±1.9 vs. CG: 5.3 ±2.9, p=0.434</td><td>Participants
N=63 (N=52 analysed)

Study groups
IG: 5 mL/kg of FFP (N=28 randomized, N=20 analysed)§

CG: normal saline (5 mL/kg) (N=35 randomized, N=32 analysed)
§ 5 withdrew, 3 were radiologically misclassified, 2 were misclassified according to the GCS, 3 were lost in follow-up
$ 2 withdrew, 1 was radiologically misclassified, 1 had no FFP transfusion, 1 was lost to follow-up</td><td><u>Mortality at 6 months: n (%)</u>
IG: 10 (50) vs. CG: 15 (46.9), p=1.000
RR: 1.133, 95% CI: 0.370-3.467, p=1.000

<u>Glagow Outcome Scale at 6 m: mean ± SD</u>
IG: 1.9 ± 0.8 vs. CG: 2.41 ± 1.2, p=0.082

<u>Delayed traumatic intracranial hematoma: n (%)§</u>
IG: 7 (35.0) vs. CG: 3 (9.4), p=0.033
RR (95% CI): 5.2505 (1.159-23.384), p=0.023
adj. OR (95% CI): 5.493 (1.953-28.652), p=0.043

<u>Length of mechanical ventilation [d]: mean ± SD</u>
IG: 2.4 ±3.8 vs. CG: 1.97 ± 3.9, p=0.697

<u>Length of antibiotics administration [d]: mean ± SD</u>
IG: 28.3 ± 21.4 vs. CG: 16.91 ±14.8, p=0.027

<u>Length of hospital stay [d] mean ± SD</u>
IG: 45.15 ±39.4 vs. CG: 25.91 ± 23.6, p=0.032

<u>Acute renal failure: n (%)</u>
IG: 2 (10.0) vs. CG: 3 (9.4), p=1.000

<u>Pneumonia: n (%)</u>
IG: 11 (55.0) vs. CG: 12 (37.5), p=0.216

<u>Acute respiratory distress syndrome: n (%)</u>
IG: 3 (15.0) vs. CG: 5 (15.6), p=1.000

<u>Intracranial infection: n (%)</u></td><td>Level of evidence
2b↓

Risk of bias
Selection bias: +
Performance bias: +
Attrition bias: –
Detection bias: +

Authors’ conclusion
“In our study comparing LEFT with NO LEFT treatment for coagulation during surgery, DTICH and prolonged length of antibiotic administration were observed in patients with severe TBI. These findings suggest that a restricted FFP transfusion protocol in the right clinical setting may be more appropriate in patients with severe TBIs.”

Reviewers’ conclusion
The results of the study need to be interpreted with caution due to the small study size and substantial</td></tr>
</table>

Study: Reference, aim, design, setting	Participants: selection criteria, characteristics	N Participants; Intervention (IG) vs. Control group (CG)	Main outcomes	Assessment: LoE, risk of bias; Conclusions
	ISS, mean ± SD<br>IG: 19.8 ±7.4 vs. CG: 23.2 ±10.2, p=0.171<br>ASA, score, mean ± SD<br>IG: 4.0 ± 0.32 vs. CG: 3.8 ± 0.5, p=0.231<br><br>**$unclear percentage values**		IG: 5 (25.0) vs. CG: 4 (12.5), p=0.280<br>Second surgery: n (%)<br>IG: 6 (30.0) vs. CG: 3 (9.4), p=0.071<br>RR (95% CI): 4.143 (0.901-19.049), p=0.071<br>PLT, x 10^9/L: mean ± SD<br>IG: 86.2 ± 36.3 vs. CG: 122.5 ± 40.7, p=0.008<br>Hemoglobin, g/L, mean ± SD<br>IG: 102.3 ± 22.1 vs. CG: 100.9 ± 25.5, p=0.859<br>§ The study was terminated early for futility and safety reasons because a high proportion of patients in the LEFT group developed new delayed traumatic intracranial hematoma after surgery compared with the NO LEFT group	dropout rate (no ITT analysis).
**Zielinski (2015)**<br>"Multicenter comparison of emergency release group A versus AB plasma in blunt-injured trauma patients." *Clinical and translational science* 2015; 8(1): 43-47.<br>**Study design**<br>Comparative registry trial<br>(Trauma databases at two institution, both part of the National Trauma Databank and the Trauma Quality Improvement Project)<br>**Aim of the study**<br>"To discriminate outcomes between	**Inclusion criteria**<br>• Bluntly injured trauma patients<br>• Age 18 or older<br>• Received at least one emergency release plasma unit<br>**Exclusion criteria**<br>N.r.<br>**Characteristics**<br>Age [y], mean ± SD<br>57.6 (25.5) 51.9 (20.0), p=0.102<br>Male, %<br>IG: 65 vs. CG:67, p=0.876<br>ISS, mean ± SD<br>IG: 20.3 ± 14.5 vs. CG: 26.8 ± 16.6, p=0.007<br>GCS, mean ± SD	**Participants**<br>N=191 patients<br>**Study groups**<br>IG: group A plasma (N=115)<br>CG: group AB plasma (N=76)<br>**Co-interventions**<br>Plasma units (more plasma and more compatible nonidentical plasma was transfused to Group AB plasma patients), pRBC, Platelets, cristalloids (Group A received more crystalloids), tranexamic acid	**Adj. outcomes**<br>Mortality: OR (95% CI)<br>0.66 (0.21–2.06)<br>**Unadj. outcomes**<br>Mortality: %<br>IG: 17 vs. CG: 26, p=0.150<br>Ventilator days: mean ± SD<br>IG: 2.8 ± 7.0 vs. CG: 3.8 ± 6.0, p=0.310<br>ICU days: mean ± SD<br>IG: 3.8 ± 7.7 vs. CG: 7.2 ± 8.8, p=0.007<br>Hospital days: mean ± SD<br>IG: 8.2 ± 9.7 vs. CG: 9.5 ± 9.1, p=0.350<br>Highest positive end expiratory pressure: mean ± SD<br>IG: 10 ± 4.6 vs. CG: 8 ±3.1, p=0.002	**Level of evidence**<br>2b<br>**Risk of bias**<br>Selection bias: +<br>Performance bias: –<br>Attrition bias: +<br>Detection bias: +<br>**Authors' conclusion**<br>"Utilization of Group A plasma for emergency blood resuscitation is a safe option which may alleviate potential shortages of AB plasma."<br>**Reviewers' conclusion**

Study: Reference, aim, design, setting	Participants: selection criteria, characteristics	N Participants; Intervention (IG) vs. Control group (CG)	Main outcomes	Assessment: LoE, risk of bias; Conclusions
practices using either the traditional universal plasma product (i.e., Group AB) and an alternative universal plasma product (i.e., Group A) in bluntly injured trauma patients, we performed a multiinstitutional retrospective review"<br><br>**Setting**<br><br>USA, 2012	IG: 11.3 ± 5.3 vs. CG: 10.6 ± 5.2, p=0.371<br><br>TRISS, mean ± SD<br><br>IG: 0.80 ± 0.29 vs. CG: 0.75 ± 0.32, p=0.330<br><br>Total plasma [units], mean ± SD<br><br>IG: 4.5 ± 5.7 vs. CG: 7.0 ± 9.0, p=0.031<br><br>Compatible nonidentical plasma [units], mean ± SD<br><br>IG: 0.17 ± 0.67 vs. CG: 1.5 ± 4.2<br><br>Total platelets [units], mean ± SD<br><br>IG: 0.52 ± 1.9 vs. CG: 1.05 ± 1.1, p=0.030<br><br>Total crystalloids [mL], mean ± SD<br><br>IG: 3845 ± 3163 vs. CG: 2501 ± 1569, p<0.001		Lowest PaO_2 /FiO_2 ratio: mean ± SD<br><br>IG: 226 ± 145 vs. CG: 206 ± 97, p=0.326<br><br>Acute respiratory distress syndrome: %<br><br>IG: 2 vs. CG: 8, p=0.060<br><br>Sepsis: %<br><br>IG: 0 vs. CG: 5, p=0.024<br><br>Pneumonia: %<br><br>IG: 7 vs. CG: 14, p=0.137<br><br>Acute renal failure: %<br><br>IG: 1 vs. CG: 11, p=0.003<br><br>Deep venous thrombosis: %<br><br>IG: 5 vs. CG: 16, p=0.021<br><br>Pulmonary embolus: %<br><br>IG: 0 vs. CG: 5, p=0.013	The study has a risk for performance bias due to unequal co-interventions.

+: low risk; –: high risk; ?: unclear risk; ACS: American College of Surgeons; adj.: adjusted; AIS: Abbreviated Injury Scale; ARDS: Acute Respiratory Distress Syndrome; ASA: American Society of Anesthesiology Score; ATC: acute traumatic coagulopathy; CI: confidence interval; CFC: coagulation factor concentrates; CG: control group; d: days; dl: decilitres; ED: emergency department; ERP: emergency-release plasma; FFP: fresh frozen plasma; GCS: Glasgow Coma Score; h: hours; HR: hazard ratio; ICU: intensive care unit; IG: intervention group; INR: International Normalized Ratio; IQR: Interquartile Range; ITT: intention to treat; ISS: injury severity score; LEFT: low-dose, early fresh frozen plasma transfusion; mg: milligrams; mmHg: millimetres of mercury; MTP: massive transfusion protocol; NISS: New Injury Severity Score; n.r.: not reported; NTDB: National Trauma Data Bank; L: litres; LoE: level of evidence; LTOWB: low-titer group O whole blood; LQP: Never-frozen liquid plasma; min: minutes; PCC: prothrombin complex concentrate; PRBC: packed red blood cells; PLT: platelets; PT: prothrombin time; PTT: activated partial thromboplastin time; OR: Odds ratio; RBC: red blood cells; SBP: systolic blood pressure; sec: seconds; SD: Standard Deviation**;** TBI: traumatic brain injury; TCDB: Traumatic Coma Data Bank; TRISS: trauma injury severity score; TXA: tranexamic acid; y: years

Tranexamic acid

Study: Reference, aim, design, setting	Participants: selection criteria, characteristics	N Participants; Intervention (IG) vs. Control group (CG)	Main outcomes	Assessment: LoE, risk of bias; Conclusions
**CRASH-3 collaborators (2019).**<br><br>"Effects of tranexamic acid on death, disability, vascular occlusive events	**Inclusion criteria**<br><br>• Adults with traumatic brain injury (TBI) ≤3 h of injury (at the beginning ≤8 h)<br>• Glasgow Coma Scale (GCS) score ≤12	**Participants**<br><br>N=12,737 patients, out of whom 9,202 (72.2%) patients within 3 h of injury.	All results for patients randomly assigned within 3h:<br><br>**Head injury-related death in hospital (28 days): n/N (%)**	**Level of evidence**<br><br>1b<br><br>**Risk of bias**

Study: Reference, aim, design, setting	Participants: selection criteria, characteristics	N Participants; Intervention (IG) vs. Control group (CG)	Main outcomes	Assessment: LoE, risk of bias; Conclusions
and other morbidities in patients with acute traumatic brain injury (CRASH-3): a randomised, placebo-controlled trial". *Lancet* 2019; 394(10210): 1713-23.<br><br>**Study design**<br><br>Randomised controlled trial<br><br>**Aim of the study**<br><br>"The CRASH-3 trial aimed to quantify the effects of tranexamic acid on head injury-related death, disability, and adverse events in patients with TBI."<br><br>**Setting**<br><br>29 countries, 2012-2019	• OR any intracranial bleeding on CT scan,<br>• no major extracranial bleeding<br>• responsible clinician was substantially uncertain as to the appropriateness of tranexamic acid treatment<br><br>**Baseline characteristics**<br><br>of those randomly assigned within 3 h of injury<br><br><u>Age [y], mean ± SD</u><br><br>IG: 41.7 (19.0)<br>CG: 41.9 (19.0)<br><br><u>Males, n (%)</u><br><br>IG: 3,742 (80)<br>CG: 3,660 (80)<br><br><u>Glagow Coma Scale Scores, n (%)</u><br><br><u>GCS 3</u>: IG: 495 (11) vs. CG: 506 (11)<br><br><u>GCS 4</u>: IG: 213 (5) vs. CG: 213 (5)<br><br><u>GCS 5</u>: IG: 163 (4) vs. CG: 172 (4)<br><br><u>GSC 6</u>: IG: 221 (5) vs. CG: 232 (5)<br><br><u>GCS 7:</u> IG: 311 (7) vs. CG: 294 (6)<br><br><u>GCS 8</u>: IG: 354 (8) vs. CG: 315 (7)<br><br><u>GCS 9</u>: IG: 335 (7) vs. CG: 292 (6)<br><br><u>GCS 10</u>: IG: 371 (8) vs. CG: 364 (8)<br><br><u>GCS 11</u>: IG: 375 (8) vs. CG: 390 (9)<br><br><u>GCS 12</u>: IG: 476 (10) vs. CG: 478 (10)<br><br><u>GCS 13</u>: IG: 297 (6) vs. CG: 312 (7)<br><br><u>GCS 14</u>: IG: 526 (11) vs. CG: 458 (10)<br><br><u>GCS 15</u>: IG: 484 (10) vs. CG: 492 (11)	**Study groups**<br><br>IG: TXA (N=6,406; 4,649 ≤3 h)<br><br>1 g of tranexamic acid infused over 10 min, started immediately after randomisation, followed by an intravenous infusion of 1 g over 8 h (four ampules of TXA 500 mg)<br><br>CG: 100 mL bag of 0.9% sodium chloride (N=6,331; 4,553 ≤3 h)	<u>Overall:</u><br><br>IG: 855/4,613 (18.5) vs. CG: 892/4514 (19.8)<br>Risk ratio (95% CI): 0.94 (0.86–1.02)<br><br><u>GCS severe (3-8)</u><br><br>IG: 689/1,739 (39.6) vs. GC: 685/1,710 (40.1)<br>Risk ratio (95% CI): 0.99 (0.91–1.07)<br><br>no obvious effect of time to treatment in patients with severe head injury (p=0·73).<br><br>**Stratification by time**<br><br>Early treatment was more effective than later treatment in patients with mild and moderate head injury (p=0·005) but we found no obvious effect of time to treatment in patients <u>with severe head injury</u> (p=0·73).<br><br>Other endpoints were not reported separately for severe TBI.	Selection bias: +<br><br>Performance bias: +<br><br>Attrition bias: +<br><br>Detection bias: +<br><br>**Authors' conclusion**<br><br>"We found a substantial reduction in head injury-related deaths with tranexamic acid in patients with mild and moderate head injuries <u>but no apparent reduction in those with severe head injury</u>."<br><br>**Reviewers' conclusion**<br><br>CRASH-3 is a randomized controlled trial of good quality with large sample size, indicating reliable results.

Study: Reference, aim, design, setting	Participants: selection criteria, characteristics	N Participants; Intervention (IG) vs. Control group (CG)	Main outcomes	Assessment: LoE, risk of bias; Conclusions
	<u>Unknown</u>: IG: 28 (1) vs. CG: 35 (1)			
**Guyette (2020)**<br>"Tranexamic Acid During Prehospital Transport in Patients at Risk for Hemorrhage After Injury: A Double-blind, Placebo-Controlled, Randomized Clinical Trial". *JAMA Surgery* 2021; 156(1): 11-20.<br>(STAAMP)<br>**Study design**<br>Randomised controlled trial<br>**Aim of the study**<br>"To assess the effectiveness and safety of tranexamic acid administered before hospitalization compared with placebo in injured patients at risk for hemorrhage."<br>**Setting**<br>USA, 2015-2019	**Inclusion criteria**<br>• injured patients at risk for hemorrhage transported from the scene or transferred from an outside emergency department<br>• at least 1 episode of hypotension (SBP ≤90 mmHg) or tachycardia (heart rate ≥110 beats per minute) before arrival at a participating center<br>**Exclusion criteria**<br>• age >90 years, <18 years<br>• lack of intravenous or intraosseous access<br>• isolated fall from standing<br>• documented cervical cord injury<br>• known prisoner or pregnancy<br>• traumatic arrest of >5 minutes<br>• penetrating brain injury<br>• isolated drowning or hanging<br>• objection to study voiced at scene<br>• wearing a STAAMP study opt-out bracelet.<br>**Characteristics**<br><u>Age [y], mean ± SD</u><br>Overall: 42 ± 18<br>IG: 41 ± 17<br>CG: 42 ± 18<br><u>Males, n (%)</u><br>Overall: 686 (74.0)<br>IG: 327 (73.2)<br>CG: 341 (74.8)<br>ISS, median (IQR)	**Participants**<br>N=903 patients<br>**Study groups**<br>IG: TXA (N=477)<br>The treatment arms received a 1-g bolus of tranexamic acid (for 10 minutes) en route to the hospital.<br>• TXA abbreviated: 1g TXA + placebo + placebo (N=151)<br>• TXA standard: 1g TXA +1g TXA + placebo (N=141)<br>• TXA repeat: 1g TXA + 1g TXA + 1g TXA (N=150)<br>CG: placebo bolus + placebo bolus + placebo infusion (N=456)<br>**Co-interventions**<br>Phase A (prehospital): infusion over 10 min<br>• 1 g of TXA 10 mL of solution + 100 mL bag of 0.9% saline<br>• 10 mL of sterile water + 100 mL bag of 0.9% saline.<br>Phase B intervention (hospital): infusion over 10 min<br>• 1g TXA in 10 mL of solution<br>• 10 mL of placebo (sterile water) added to a 100 mL bag of 0.9% saline<br>Phase C intervention (hospital): infusion over 8h<br>• 1 g of TXA in 10 mL of solution	**Mortality (30 days): n/N (%)**<br><u>Overall</u><br>IG: 36/442 (8.1) vs. 45/452 (10.0) (9 missing)<br>Risk ratio (95% CI) 0.82 (0.60-1.11)<br><u>By dosing regimen</u><br>*TXA abbreviated* 14/150 (9.3) vs. CG: 45/452 (10.0)<br>Risk ratio (95% CI): 0.94 (0.65-1.36), p=0.74<br>*TXA standard* 11/141 (7.8) vs. CG: 45/452 (10.0)<br>Risk ratio (95% CI): 0.78 (0.50-1.24), p=0.30<br>*TXA repeat* 11/151 (7.3) vs. CG: 45/452 (10.0)<br>Risk ratio (95% CI): 0.73 (0.54-0.99), p=0.04<br>**By TBI severity**<br><u>No severe TBI (head AIS≤2)</u><br>IG: 17/352 (4.8) vs. CG: 25/374 (6.7)<br>Risk ratio (95% CI): 0.72 (0.46-1.14)<br><u>Severe TBI (head AIS>2)</u><br>IG: 19/90 (21.1) vs. 20/78 (25.6)<br>Risk ratio (95% CI): 0.82 (0.55-1.24)<br>Adj. p=0.86 for interaction<br>**By transfusion received**<br><u>No transfusion received</u><br>IG: 10/289 (3.5) vs. CG: 10/295 (3.4)<br>Risk ratio (95% CI): 1.02 (0.49-2.15)<br><u>Transfusion received</u><br>IG: 26/153 (17.0) vs. 35/157 (22.3)<br>Risk ratio (95% CI): 0.76 (0.57-1.01)<br>Adj. p=0.32 for interaction	**Level of evidence**<br>1b<br>**Risk of bias**<br>Selection bias: +<br>Performance bias: +<br>Attrition bias: +<br>Detection bias: +<br>**Authors' conclusion**<br>"Patients with severe shock (SBP ≤70mmHg) who received tranexamic acid demonstrated lower 30-day mortality compared with placebo."<br>**Reviewers' conclusion**<br>The study has a low risk of bias. The subgroup of severe shock patients (SBP ≤70mmHg) contains a small number of patients (N=58).

Study: Reference, aim, design, setting	Participants: selection criteria, characteristics	N Participants; Intervention (IG) vs. Control group (CG)	Main outcomes	Assessment: LoE, risk of bias; Conclusions
	Overall: 12 (5-22)<br>IG: 13 (5-22)<br>CG: 11 (4-22)<br><u>Initial GCS<8: n (%)</u><br>IG: 89 (19.9)<br>CG: 107 (23.5)	10 mL of placebo + 100 mL bag of 0.9% saline	**By shock severity (post-hoc analysis)**<br><u>Tachycardia only</u><br>IG: 18/316 (5.7) vs. CG: 21/320 (6.6)<br>Risk ratio (95% CI): 0.87 (0.56-1.34), p=0.52<br><u>SBP <90 mm Hg</u><br>IG: 13/99 (13.1) vs. CG: 13/101 (12.9)<br>Risk ratio (95% CI): 1.02 (0.55-1.90), p=0.95<br><u>SBP <70 mm Hg</u><br>IG: 5/27 (18.5) vs. CG: 11/31 (35.5)<br>Risk ratio (95% CI): 0.52 (0.34-0.80), p=0.003<br>**By time from injury (post-hoc analysis)**<br><u>≤1h</u><br>IG: 10/219 (4.6) vs. CG: 18/238 (7.6)<br>Risk ratio (95% CI): 0.60 (0.44-0.83)<br><u>>1h</u><br>IG: 26/223 (11.7) vs. 27/214 (12.6)<br>Risk ratio (95% CI): 0.92 (0.52-1.64)<br>§only percentage reported	
**Khan (2018)**<br>„Severely Injured Trauma Patients With Admission Hyperfibrinolysis; Is There A Role Of Tranexemic Acid? Findings From The PROPPR Trial", *Journal of Trauma and Acute Care Surgery* 2019; 85(5): 851–857.<br>**Study design**	In Addition to PROPPR<br>**Inclusion criteria**<br>• trauma patients with hyperfibrinolysis on admission measured via thromboelastography. Hyperfibrinolysis was defined as Ly30 ≥3% on thromboelastography<br>**Exclusion criteria**<br>• all patients who received TXA >3 hours of injury<br>**Characteristics**<br><u>Age: mean, ± SD</u>	**Participants**<br>N=93 patients, matched in 1:2 ratio<br>(117 patients pre-matching)<br>**Study groups**<br>IG: TXA (N=31)<br>CG: no TXA (N=62)<br>**Matching criteria**<br>Propensity score matching according to age, gender, race, ED SBP, ED heart rate, mechanism of injury, ISS, head-AIS, GCS,	**Mortality§ - primary outcomes**<br><u>6-hour: %§</u><br>IG: 13 (p=0.04)<br>CG: 34<br><u>24 hour: %§</u><br>IG: 26 (p=0.25)<br>CG: 39<br><u>30 day: %§</u><br>IG: 45 (p=0.82)<br>CG: 50<br>**Cause of death§**	**Level of evidence**<br>2b<br>**Risk of bias**<br>Selection bias: +<br>Performance bias: +<br>Attrition bias: +<br>Detection bias: +<br>**Authors' conclusion**<br>"Tranexamic acid (TXA) was associated with increased 6 hour survival but does not

Study: Reference, aim, design, setting	Participants: selection criteria, characteristics	N Participants; Intervention (IG) vs. Control group (CG)	Main outcomes	Assessment: LoE, risk of bias; Conclusions
Comparative registry trial (secondary analysis of PROPPR database)<br><br>**Aim of the study**<br><br>“The aim of the study was to analyze the role of TXA in severely injured trauma patients with admission hyperfibrinolysis.“<br><br>**Setting**<br><br>North America, PROPPR Trial 2012-2013	IG: 42.5 ± 20 (p=0.33)<br>CG: 38.7 ± 17<br><br>Male gender: % §<br><br>IG: 66% (p=0.84)<br>CG: 68%<br><br>Injury Severity Score (ISS): median (IQR)<br><br>IG: 38 (23 - 45) (p=0.56)<br>CG: 35 (21 - 45)<br><br>GCS: median (IQR)<br><br>IG: 6 (3 - 15) (p=0.34)<br>CG: 8 (3 - 15)<br><br>SBP: median (IQR)<br><br>IG: 90 (70 - 126) (p=0.28)<br>CG: 101 (80 - 131)<br><br>Lactate: median (IQR)<br><br>IG: 8.3 (5.1 - 11.7) (p=0.83)<br>CG: 9.5 (5.1 - 12.7)<br><br>Transfusion ratio (1:1:1): %§<br><br>IG: 55%<br>CG: 47%<br><br>§ n=total number of patients not reported	and PROPPR intervention groups (1:1:1 or 1:1:2 transfusion ratios).	Exsanguination/Hemorrhagic shock: %§<br><br>IG: 26 (p=0.39)<br>CG: 32<br><br>TBI: %§<br><br>IG: 10 (p=0.62)<br>CG: 13<br><br>Respiratory: %§<br><br>IG: 6.4 (p=0.26)<br>CG: 1.6<br><br>Other: %§<br><br>IG: 3.2 (p=1.00)<br>CG: 3.2<br><br>**Complications§ - Secondary outcomes**<br><br>Deep venous thrombosis: %<br><br>IG: 6.5 (p=0.59)<br>CG: 3.2<br><br>Acute Kidney Injury: %<br><br>IG: 45 (p=0.01)<br>CG: 19<br><br>Sepsis: %<br><br>IG: 35 (p=0.04)<br>CG: 16<br><br>Multiple organ failure: %<br><br>IG: 19 (p=0.01)<br>CG: 6.4<br><br>ICU free days: median (IQR)<br><br>IG: 0 (0 – 3) (p=0.22)<br>CG: 0 (0–5)<br><br>§ n=number of patients with event not reported	improve long term outcomes in severely injured trauma patients with hemorrhage who develop hyperfibrinolysis.”<br><br>**Reviewers’ conclusion**<br><br>There may be a risk of performance bias because TXA use was not prescribed in the PROPPR study protocol and left to the discretion of the trauma attending. However, patients received the same care apart from TXA use and cohorts were matched according to confounders and transfusion ratios showing consistent results.

Study: Reference, aim, design, setting	Participants: selection criteria, characteristics	N Participants; Intervention (IG) vs. Control group (CG)	Main outcomes	Assessment: LoE, risk of bias; Conclusions
**Moore (2017)**<br>"Tranexamic acid is associated with increased mortality in patients with physiological fibrinolysis ", *Journal of Surgical Research* 2017; 220: 438-443.<br>**Study design**<br>Prospective cohort study<br>**Aim of the study**<br>"The aim of the study was to investigate if TXA in patients with a physiological level of fibrinolysis will have an increase in mortality compared with other fibrinolytic phenotypes."<br>**Setting:**<br>USA, 2014-2016	**Inclusion criteria**<br>• Adult trauma patients (aged >18 years)<br>• highest level of activation at level I trauma center<br>• new injury severity score (NISS) >15<br>**Characteristics**<br><u>Age: median (IQR)</u><br>IG: 27 (24-54) (p=0.214)<br>CG: 34 (27-49)<br><u>Male gender: %[§]</u><br>IG: 85% (p=0.362)<br>CG: 77%<br><u>NISS: median (IQR)</u><br>IG: 48 (29-57) (p=0.001)<br>CG: 29 (22-43)<br><u>INR:median (IQR)</u><br>IG: 1.4 (1.2-1.8) (p<0.001)<br>CG: 1.2 (1.1-1.3)<br>**Fibrinolysis phenotype (n)**<br><u>Hyperfibrinolysis: n (N=64)</u><br>IG: 10<br>CG: 54<br><u>Shutdown: n (N=54)</u><br>IG: 8<br>CG: 46<br><u>Physiologic: n (N=114)</u><br>IG: 8<br>CG: 106	**Participants**<br>N=232 patients<br>**Study groups**<br>IG: TXA (N=26)<br>CG: no TXA (N=206)	**Mortality**<br><u>Mortality (in-hospital): %[§]</u><br>IG: 50% (p<0.001)<br>CG: 17%<br><u>Mortality (in-hospital) within phenotypes (n, (%))</u><br><u>Hyperfibrinolysis</u><br>IG: 56% (p=0.023)<br>CG: 19%<br><u>Shutdown</u><br>IG: 38% (p=0.604)<br>CG: 28%<br><u>Physiologic</u><br>IG: 63% (p<0.001)<br>CG: 11%<br>**Death associated with haemorrhage[§] (%)**<br>IG: 55% (p=0.060)<br>CG: 23%<br>**TXA as predictor of mortality by fibrinolysis phenotype (adj. for NISS)**<br>Physiologic (p=0.018)<br>Hyperfibrinolysis (p=0.116)<br>Shutdown (p=0.597)<br>**Massive transfusion: %[§]**<br>IG: 69% (p<0.001)<br>CG: 12%<br><br>[§] n=number of patients with event not reported	**Level of evidence**<br>2b<br>**Risk of bias**<br>Selection bias: –<br>Performance bias: –<br>Attrition bias: +<br>Detection bias: +<br>**Authors' conclusion**<br>"There was no clear benefit of receiving TXA in this study, and patients who present to the hospital with physiologic levels of fibrinolysis, who received TXA, had the highest mortality."<br>**Reviewers' conclusion**<br>**There is a substantial risk of selection bias due to imbalance of NISS and INR. Blinding was unclear and co-interventions were different (patients in the TXA group tended to receive more blood products), so that there is a risk for performance bias. The risk of attrition bias is unclear because the numbers in the analyses were not reported.**

Study: Reference, aim, design, setting	Participants: selection criteria, characteristics	N Participants; Intervention (IG) vs. Control group (CG)	Main outcomes	Assessment: LoE, risk of bias; Conclusions
	§ n=number of patients with event not reported			
**Nishijima (2019)**<br>“The Effect of Tranexamic Acid on Functional Outcomes: An Exploratory Analysis of the CRASH-2 Randomized Controlled Trial“. *Annals of Emergency Medicine* 2019; 74(1), 79-87<br>**Study design**<br>Randomised controlled trial (exploratory Analysis of CRASH-2 trial data)<br>**Aim of the study**<br>“The aim of the study was to evaluate whether tranexamic acid was associated with improved functional outcomes and, if so, which patients benefitted from tranexamic acid use. “<br>**Setting**<br>40 countries, 2005-2010	**Inclusion criteria**<br>• Same as in CRASH-2 trial and<br>• only patients randomized 3 hours or less from the time of injury<br>**Exclusion criteria**<br>• patients who did not have modified Oxford Handicap Scale scores reported<br>**Characteristics**<br><u>Age (mean ± SD)</u><br>IG: 34.1 (± 13.8)<br>CG: 34.1 (± 14.2)<br><u>Males: n (%))</u><br>IG: 5,605 (83.0)<br>CG: 5,606 (84.0)<br><u>Initial GCS: median (IQR)</u><br>IG: 12.7 (3.6)<br>CG: 12.7 (3.6)<br>**Baseline risk of mortality stratum: n %)§**<br><u><6</u><br>IG: 2,415 (35.8)<br>CG: 2,325 (34.9)<br><u>6-20</u><br>IG: 2,410 (35.7)<br>CG: 2,391 (35.9)<br><u>21- 50</u><br>IG: 1,171 (17.4)<br>CG: 1,201 (18.0)	**Participants**<br>N=13,432 patients<br>subset of CRASH-2 dataset<br>**Study groups**<br>IG: TXA (N=6,753)<br>CG: Placebo (N=6,679)	**Modified Oxford Handicap Scale score (at discharge or at 28 days): n (%)**<br><u>No symptoms</u><br>IG: 1,052 (15.6)<br>CG: 941 (13.9)<br><u>Minor symptoms</u><br>IG: 2,190 (32.4)<br>CG: 2,140 (32.0)<br><u>Some restrictions</u><br>IG: 1,311 (19.4)<br>CG: 1,324 (19.8)<br><u>Dependent</u><br>IG: 807 (11.9)<br>CG: 779 (11.7)<br><u>Fully dependent</u><br>IG: 421 (6.2)<br>CG: 396 (5.9)<br><u>Dead</u><br>IG: 972 (14.4)<br>CG: 1,109 (16.6)<br><u>mean utility-weighted modified Oxford Handicap Scale score: mean ± SD</u><br>IG: 0.66 (± 0.33)<br>CG: 0.64 (± 0.34)<br>mean difference = 0.02 (95% CI 0.01 – 0.03) (p<0.001)<br><u>28-day mean utility-weighted modified Oxford Handicap Scale score (Area under the curve analysis): mean ± SD:</u>	**Level of evidence**<br>1b<br>**Risk of bias**<br>Selection bias: +<br>Performance bias: +<br>Attrition bias: +<br>Detection bias: +<br>**Authors’ conclusion**<br>“In this exploratory analysis of the CRASH-2 study, we found that adult trauma patients randomized to tranexamic acid within 3 hours of injury had better functional outcomes compared with patients randomized to placebo.”<br>**Reviewers’ conclusion**<br>The trial is of good quality indicating reliable results.

Study: Reference, aim, design, setting	Participants: selection criteria, characteristics	N Participants; Intervention (IG) vs. Control group (CG)	Main outcomes	Assessment: LoE, risk of bias; Conclusions
	>50<br>IG: 753 (11.2)<br>CG: 752 (11.3)<br>Days in hospital (median, IQR)<br>IG: 7 (3–14)<br>CG: 7 (3–14)		IG: 0.55 (± 0.30)<br>CG: 0.53 (± 0.31)<br>mean difference = 0.02 (95% CI 0.01 – 0.03)<br>**Functional outcomes, stratified by CRASH-2 prognostic score: n (%) (95% CI) §**<br>0-6 % baseline risk<br>Overall favourable outcome (no symptoms)<br>IG: 534 (22.1) (95% CI 20.5 – 23.8)<br>CG: 425 (18.3) (95% CI 16.7 – 19.9)<br>IG vs. CG: adj. OR for favourable outcome = 1.28 (95% CI 1.11 – 1.48)<br>6-20 % baseline risk<br>Overall favourable outcome (no or minor symptoms)<br>IG: 1209 (50.2) (95% CI 48.1 – 52.2)<br>CG: 1202 (50.3) (95% CI 48.2 – 52.3)<br>IG vs. CG: adj. OR for favourable outcome = 0.99 (95% CI 0.88 – 1.11)<br>21-50 % baseline risk<br>Overall favourable outcome (no or minor symptoms or some restrictions)<br>IG: 611 (52.2) (95% CI 49.3 – 55.1)<br>CG: 588 (49.0) (95% CI 46.1% - 51.8)<br>IG vs. CG: adj. OR for favourable outcome = 1.15 (95% CI 0.97 – 1.37)<br>>50 % baseline risk<br>Overall favourable outcome (no or minor symptoms or some restrictions or dependent)<br>IG: 238 (31.6) (95% CI 28.3 – 35.1)<br>CG: 217 (28.9) (95% CI 25.6 – 32.2)	

Study: Reference, aim, design, setting	Participants: selection criteria, characteristics	N Participants; Intervention (IG) vs. Control group (CG)	Main outcomes	Assessment: LoE, risk of bias; Conclusions
			IG vs. CG: adj. OR for favourable outcome = 1.24 (95% CI 0.97 – 1.57)<br><br>Overall proportion of patients with favourable outcomes: n (%) (95% CI):<br>IG: 5,360 (79.4) (95% CI 78.4% - 80.3)<br>CG: 5,174 (77.) (95% CI 76.5% - 78.5)<br><br>difference 1.9% (95% CI 0.5% - 3.3)<br><br>NNT = 52 (95% CI 30 – 196)<br><br>§ Favourable versus unfavourable outcomes were defined separately for each risk stratum	
**Roberts (2017)**<br>"Tranexamic acid in bleeding trauma patients: an exploration of benefits and harms." *Trials* 2017; 18: 48.<br><br>**Study design**<br>Randomised controlled trial (predefined subgroup analysis of CRASH-2)<br><br>**Aim of the study**<br>"We examine how patient characteristics vary by time to treatment in the CRASH-2 trial and explore whether any such variations explain the time-dependent treatment effect."<br><br>**Setting**<br>40 countries, 2005-2010	Same as *CRASH-2:*<br><br>**Inclusion criteria**<br>• Adult trauma patients<br>• with, or at risk of, significant bleeding within 8 h of their injury<br><br>**Characteristics**<br>no patient characteristics reported	**Participants**<br>N=20,211 patients<br><br>**Study groups**<br>IG: TXA (loading dose 1 g over 10 min followed by an infusion of 1 g over 8 h) (N=10,093)<br><br>CG: matching placebo (N=10,114)	**Subgroup analyses of CRASH-2**<br>• SBP (≤75, 76–89, >89 mmHg)<br>• GCS score (severe 3–8, moderate 9–12, mild 13–15)<br>• type of injury (penetrating versus blunt)<br><br>1. Effects of **early** tranexamic acid (TXA) treatment stratified by systolic blood pressure on death due to bleeding: Risk Ratio (95% CI)<br>SBP ≤75 RR: 0.73 (0.61-0.86)<br><br>SBP 76-89 RR: 0.86 (0.64-1.16)<br><br>SBP>89 RR: 0.71 (0.54-0.92)<br><br>SBP <100 mg and treatment initiated **within 1h**<br><br>RR = 0.69 (0.58 - 0.83)<br><br>SBP <100 mg and treatment **between 1-3h**<br><br>RR = 0.84; 95% (0.67 - 1.04)<br><br>Effects of **late** tranexamic acid (TXA) treatment stratified by **systolic blood pressure** (SBP) on death due to bleeding: Risk Ratio (95% CI)<br>Systolic blood pressure (mm Hg) ≤75,<br><br>1.36 (0.92-2.01)	**Level of evidence**<br>1b<br><br>**Risk of bias**<br>Selection bias: +<br><br>Performance bias: +<br><br>Attrition bias: +<br><br>Detection bias: +<br><br>**Authors' conclusion**<br>"When given within 3 h of injury, TXA reduces death due to bleeding regardless of injury type, GCS or blood pressure."<br><br>**Reviewers' conclusion**<br>This predefined subgroup analyses of the CRASH-2 trial is of good quality indicating reliable results.

Study: Reference, aim, design, setting	Participants: selection criteria, characteristics	N Participants; Intervention (IG) vs. Control group (CG)	Main outcomes	Assessment: LoE, risk of bias; Conclusions
			<u>2. Effects of **early** tranexamic acid (TXA) treatment stratified by **Glasgow Coma Scale** (GCS) score on death due to bleeding: Risk Ratio (95% CI)</u><br>GCS 3-8 RR: 0.82 (0.66-1.02)<br><u>Effects of **late** tranexamic acid (TXA) treatment stratified by **Glasgow Coma Scale** (GCS) score on death due to bleeding: Risk Ratio (95% CI)</u><br>GCS 3-8<br>1.42 (0.90-2.25)<br><u>3. Effects of **early** tranexamic acid (TXA) treatment stratified by **type of injury** on death due to bleeding: Risk Ratio (95% CI)</u><br>Blunt 0.72 (0.60 - 0.86)<br>Penetrating 0.73 (0.60 – 0.90)<br><u>Effects of **late** tranexamic acid (TXA) treatment stratified by **type of injury** on death due to bleeding: Risk Ratio (95% CI)</u><br>Blunt 1.48 (1.12 – 1.96)<br>Penetrating 1.25 (0.74 – 2.12)	
**Roberts (2014)**<br>"Mechanism of action of tranexamic acid in bleeding trauma patients: an exploratory analysis of data from the CRASH-2 trial." *Critical Care* 2014; 18(6): 1-5.<br>**Study design**<br>Randomised controlled trial (CRASH-2)<br>**Aim of the study**	Same as *CRASH-2:*<br>**Inclusion criteria**<br>• adult trauma patients with, or at risk of, significant bleeding, and<br>• who were within 8 h of their injury<br>**Characteristics**<br>no patient characteristics reported	**Participants**<br>N=20,211 patients<br>**Study groups**<br>IG: TXA (loading dose 1 g over 10 minutes followed by an infusion of 1 g over 8 h) (N=10,060 with outcome data)<br>CG: matching placebo (N=10,067 with outcome data)	**All cause mortality (incl. non-bleeding patients!)**<br><u>0 days since injury, Hazard Ratio (95% CI)</u><br>0.83 (0.73, 0.93)<br><u>1 day since injury, Hazard Ratio (95% CI)</u><br>0.91 (0.79, 1.04)<br><u>2 days since injury, Hazard Ratio (95% CI)</u><br>0.96 (0.77, 1.19)<br><u>3 days since injury, Hazard Ratio (95% CI)</u><br>1.01 (0.76, 1.34)<br><u>4 days since injury, Hazard Ratio (95% CI)</u>	**Level of evidence**<br>1b<br>**Risk of bias**<br>Selection bias: +<br>Performance bias: +<br>Attrition bias: +<br>Detection bias: +<br>**Authors' conclusion**<br>"Early administration of tranexamic acid appears to

Study: Reference, aim, design, setting	Participants: selection criteria, characteristics	N Participants; Intervention (IG) vs. Control group (CG)	Main outcomes	Assessment: LoE, risk of bias; Conclusions
"We conducted further analyses of the CRASH-2 trial data to examine the timing of the effect of TXA on mortality."<br>**Setting**<br>40 countries, 2005-2010			0.96 (0.70, 1.36)<br>**Mortality due to bleeding**<br><u>0 days since injury, Hazard Ratio (95% CI)</u><br>0.80 (0.68, 0.94)<br><u>1 day since injury, Hazard Ratio (95% CI)</u><br>0.89 (0.72, 1.11)<br><u>2 days since injury, Hazard Ratio (95% CI)</u><br>1.17 (0.74, 1.86)<br><u>3 days since injury, Hazard Ratio (95% CI)</u><br>0.66 (0.32, 1.37)<br><u>4 days since injury, Hazard Ratio (95% CI)</u><br>0.77 (0.29, 2.06)<br>**Non-bleeding mortality**<br><u>0 days since injury, Hazard Ratio (95% CI)</u><br>0.87 (0.71, 1.06)<br><u>1 day since injury, Hazard Ratio (95% CI)</u><br>0.92 (0.76, 1.11)<br><u>2 days since injury, Hazard Ratio (95% CI)</u><br>0.91 (0.71, 1.16)<br><u>3 days since injury, Hazard Ratio (95% CI)</u><br>1.09 (0.80, 1.48)<br><u>4 days since injury, Hazard Ratio (95% CI)</u><br>1.01 (0.71, 1.43)<br>**All cause mortality, Time to treatment <u>≤3h</u> (incl. non-bleeding patients!)**<br><u>0 days since injury, Hazard Ratio (95% CI)</u><br>0.78 (0.68, 0.90)	reduce mortality primarily by preventing exsanguination on the day of the injury."<br>**Reviewers' conclusion**<br>It is unclear if the analysis was predefined. Apart from that, this subgroup analyses of the CRASH-2 trial is of good quality indicating reliable results.

Study: Reference, aim, design, setting	Participants: selection criteria, characteristics	N Participants; Intervention (IG) vs. Control group (CG)	Main outcomes	Assessment: LoE, risk of bias; Conclusions
			<u>1 day since injury, Hazard Ratio (95% CI)</u><br>0.86 (0.72, 1.02)<br><u>2 days since injury, Hazard Ratio (95% CI)</u><br>0.86 (0.65, 1.13)<br><u>3 days since injury, Hazard Ratio (95% CI)</u><br>0.95 (0.66, 1.37)<br><u>4 days since injury, Hazard Ratio (95% CI)</u><br>0.94 (0.61, 1.45)<br>**All cause mortality, time to treatment <u>>3h</u> (incl. non-bleeding patients!)**<br><u>0 days since injury, Hazard Ratio (95% CI)</u><br>1.02 (0.76, 1.36)<br><u>1 day since injury, Hazard Ratio (95% CI)</u><br><u>1.02 (0.80, 1.31)</u><br><u>2 days since injury, Hazard Ratio (95% CI)</u><br>1.16 (0.81, 1.66)<br><u>3 days since injury, Hazard Ratio (95% CI)</u><br>1.11 (0.73, 1.71)<br><u>4 days since injury, Hazard Ratio (95% CI)</u><br>1.04 (0.62, 1.75)	
**Spinella (2020)**<br>"The immunologic effect of early intravenous two and four gram bolus dosing of tranexamic acid compared to placebo in patients with severe traumatic bleeding (TAMPITI): A randomized, double-blind, placebo-	**Inclusion criteria**<br>• Age ≥18<br>• sustained a traumatic injury which required them to receive at least one unit of red blood cells (RBC) or required an emergent operation for possible bleeding control	**Participants**<br>N=150 patients<br>**Study groups**<br>TXA 2g: 2 g of TXA (N=49)*<br>TXA 4g: 4 g of TXA (N=50)<br>CG: placebo (N=50)	<u>28-day mortality n/N, (%)</u><br>CG: 6/49 (12.2) vs.<br>TXA 2g: 5/44 (11.4) vs.<br>TXA 4g: 4/48 (8.33), p=0.8<br><u>Thromboembolic event n/N, (%)</u><br>CG: 6/50 (12.0) vs.<br>TXA 2g: 13/49 (26.5) vs.<br>TXA 4g: 16/50 (32.0), p=0.05	**Level of evidence**<br>2b↓<br>**Risk of bias**<br>Selection bias: +<br>Performance bias: +<br>Attrition bias: +

Study: Reference, aim, design, setting	Participants: selection criteria, characteristics	N Participants; Intervention (IG) vs. Control group (CG)	Main outcomes	Assessment: LoE, risk of bias; Conclusions
controlled, single-center trial". *Frontiers in Immunology* 2020; 11: 2085.<br><br>**Study design**<br>Randomised controlled trial<br><br>**Aim of the study**<br>"The hemostatic properties of tranexamic acid (TXA) are well described, but the immunological effects of TXA administration after traumatic injury have not been thoroughly examined. We hypothesized TXA would reduce monocyte activation in bleeding trauma patients with severe injury."<br><br>**Setting**<br>USA, 2016-2017	• were able to receive the study medication (TXA or placebo) within 2 h of time of injury<br><br>**Exclusion criteria**<br>• Suspected acute MI or stroke (thromboembolic and/or hemorrhagic) on admission<br>• Known inherited coagulation disorders<br>• Known past medical history of thromboembolic events (DVT, PE, MI, Thromboembolic Stroke)<br>• Known history of seizures and/or seizure after injury/on admission related to this hospitalization<br>• Suspected or known pregnancy<br>• Futile care<br>• Known current state of immunosuppression (i.e. on high dose steroids, chemotherapeutics, etc.)<br>• Unknown estimated time of injury<br>• Patients wearing an "Opt Out" TAMPITI Study bracelet<br>• Known presence of subarachnoid hemorrhage<br>• Isolated injuries to hands and/or feet (distal)<br>• Administration of antifibrinolytics pre-hospital and/or during this ED admission prior to enrollment<br><br>**Characteristics**<br><u>Age [y], median (IQR)</u><br>CG: 27.0 (22.0 – 34.0) vs.<br>TXA 2g: 26.0 (22.0 – 40.0) vs.<br>TXA 4g: 31.0 (25.0 – 44.0), p=0.13<br><br><u>Male n (%)</u>	each in 40 mL of normal saline i.v. over 10 min<br><br>* 1 patient withdrawn (age <18 y)	<u>ICU admission n/N, (%)</u><br>CG: 37/50 (74.0) vs.<br>TXA 2g: 36/49 (73.5) vs.<br>TXA 4g: 38/50, p=0.96<br><br><u>Mechanical ventilation n/N, (%)</u><br>CG: 30/50 (60.0) vs.<br>TXA 2g: 28/48 (58.3) vs.<br>TXA 4g: 30/49 (61.2), p=0.96<br><br><u>ICU-free Days, [N] median (IQR)</u><br>CG: [50] 27.3 (17.4 – 28.6) vs.<br>TXA 2g: [45] 27.1 (24.0 – 29.4) vs.<br>TXA 4g: [49] 27.1 (24.3 – 29.0), p=0.77<br><br><u>Max MODS in 7 days , [N] median (IQR)</u><br>CG: [49] 4.00 (1.00 – 7.00) vs.<br>TXA 2g: [49] 4.00 (1.00 – 6.00) vs.<br>TXA 4g: [50] 4.00 (1.00 – 8.00), p=0.79<br><br><u>Seizure n/N, (%)</u><br>CG: 0/49 (0.00) vs.<br>TXA 2g: 1/44 (2.27) vs.<br>TXA 4g 2/48 (4.17), p=0.42	Detection bias: +<br><br>**Authors' conclusion**<br>"In conclusion, in this RCT in patients with primarily penetrating traumatic injuries, 2 and 4 g i.v. bolus dosing of TXA had minimal immunomodulatory and hemostatic effects."<br><br>**Reviewers' conclusion**<br>The clinical outcomes (mortality, morbidity) were secondary outcomes.<br>The RCT was not powered to detect differences in clinical outcomes.

Study: Reference, aim, design, setting	Participants: selection criteria, characteristics	N Participants; Intervention (IG) vs. Control group (CG)	Main outcomes	Assessment: LoE, risk of bias; Conclusions
	CG: 45 (90.0) vs.<br>TXA 2g: 44 (90.0) vs.<br>TXA 4g: 42 (84.0), p=0.58<br><br><u>GCS, [n] median (IQR)</u><br>CG: 15.0 (12.0 – 15.0) vs.<br>TXA 2g: 15.0 (11.0 – 15.0) vs.<br>TXA 4g: 15.0 (14.0 – 15.0), p=0.26			
+: low risk; –: high risk; ?: unclear risk; adj.: adjusted; AIS: Abbreviated Injury Scale; BD: base deficit; CG: control group; CI: Confidence Interval; CRASH-3: Corticosteroid randomisation after significant head injury – 3; d: days; DVT: deep venous thrombosis; ED: emergency department; GCS: Glasgow Coma Score; h: hours; IG: intervention group; INR: international normalized ratio; IQR: Interquartile Range; ISS: injury severity score; ITT: Intention to treat analysis; L: litres; LoE: level of evidence; m: months; MAP: mean arterial pressure; MI: myocardial infarction; min: minutes; mmHg: millimetres of mercury; MODS: multiple organ dysfunction syndrome; NISS: new injury severity score; NTT: number needed to treat; PE: pulmonary embolism; PROPPR: Pragmatic, Randomized Optimal Platelet and Plasma Ratios; RBC: red blood cells; RR: Relative Risk; s: seconds; SBP: systolic blood pressure; SD: Standard Deviation; TBI: traumatic brain injury; TXA: tranexcamic acid; y: years				

Fibrinogen

Study: Reference, aim, design, setting	Participants: selection criteria, characteristics	N Participants; Intervention (IG) vs. Control group (CG)	Main outcomes	Assessment: LoE, risk of bias; Conclusions
**Curry (2018)**<br>"Early fibrinogen concentrate therapy for major haemorrhage in trauma (E-FIT 1): results from a UK multi-centre, randomised, double blind, placebo-controlled pilot trial." *Critical Care* 2018; 22(1): 1-9.<br><br>**Study design**<br>Randomized controlled feasibility trial<br><br>**Aim of the study**<br>"The primary objectives of the E-FIT 1 study were to determine whether it was possible to deliver FgC	**Inclusion criteria**<br>• adults (judged to be aged 16 years or older),<br>• were actively bleeding and in haemorrhagic shock and therefore required activation of the major haemorrhage protocol (MHP) or had already received a transfusion of emergency red blood cells (RBC)<br><br>**Exclusion criteria**<br>• patient transferred from another hospital,<br>• the trauma team leader deemed the injury incompatible with life,<br>• more than 3 hours had elapsed from time of injury,<br>• pregnant women	**Participants**<br>N=39 patients (N=48 randomized, n=2 no intravenous access could be established, n=7 initially deemed eligible were subsequently found not to meet eligibility criteria)<br><br>**Study groups**<br>IG: 6 g of fibrinogen concentrate (N=24 randomized; N=20 analyzed)<br>CG: equivalent volume (300 ml) 0.9% saline (N=24 randomized; N=19 analysed)<br><br>**Definition standard therapy**<br>Typically an MHP constituted two transfusion packs—pack 1 followed by repeated use of pack 2—until bleeding was controlled. Pack 1 included 4 RBC and 4 FFP; pack 2 included 4 RBC, 4 FFP, 10 U of	**Primary outcomes**<br><u>Proportion of all participants randomised who started their infusion within 45 min: % (95% CI)</u><br>69 (52–83)<br><br><u>Proportion of participants whose fibrinogen level remained at 2 g/L or above during first 2h: n/N (%) (95% CI in %)</u><br>IG: 15/20 (75) (51-91%) vs. CG: 8/17 (47) (23-72%), p=0.10<br><br>**Secondary outcomes**<br><u>All-cause mortality at 28 days: % (95% CI)</u><br>IG: 42.0 (25.2–64.0%) vs. CG: 29.2 (15.1–51.6)<br><br><u>Number of participants experiencing at least one serious adverse events: n</u><br>IG: 13 vs. CG: 11	**Level of evidence**<br>2b↓<br><br>**Risk of bias**<br>Selection bias: +<br>Performance bias: +<br>Attrition bias: +<br>Detection bias: +<br><br>**Authors' conclusion**<br>"Although evidence points to a key role for fibrinogen in the treatment of major bleeding, researchers need to recognise the challenges of timely delivery in the emergency setting."

Study: Reference, aim, design, setting	Participants: selection criteria, characteristics	N Participants; Intervention (IG) vs. Control group (CG)	Main outcomes	Assessment: LoE, risk of bias; Conclusions
therapy early (within 45 minutes) to adult trauma patients and the proportion of participants whose fibrinogen levels were maintained ≥2 g/L during active haemorrhage."<br><br>**Setting**<br><br>UK, 2016	• severe isolated or unsalvageable head injury<br><br>**Characteristics**<br><br>Age [y], median (IQR)<br><br>IG: 38 (31–47) vs. CG: 36 (22–56)<br><br>Males, n (%)<br><br>IG: 20 (83) vs. CG: 19 (79)<br><br>ISS, median (IQR)<br><br>IG: 34 (24–43) vs. CG: 29 (22–34)<br><br>GCS, median (IQR)<br><br>IG: 3 (3–14) vs. CG: 3 (3–15)	cryoprecipitate (approximately 300 ml, 4 g of fibrinogen) and 1 pool platelets.	Number of serious adverse events: n<br><br>IG: 29 vs. CG: 21<br><br>Myocardial infarction: n<br><br>IG: 0 vs. CG: 0<br><br>Stroke: n<br><br>IG: 1 vs. CG: 1<br><br>Other arterial symptomatic thrombotic events: n<br><br>IG: 0 vs. CG: 1<br><br>Deep venous thrombosis: n<br><br>IG: 0 vs. CG: 0<br><br>Pulmonary embolus: n<br><br>IG: 2 vs. CG: 0<br><br>Sepsis: n<br><br>IG: 4 vs. CG: 6<br><br>Organ failure: n<br><br>IG: 10 vs. CG: 2<br><br>Multiple organ failure: n<br><br>IG: 4 vs. CG: 1<br><br>Single organ failure: n<br><br>IG: 6 vs. CG: 1<br><br>New-oneset major bleeding: n<br><br>IG: 1 vs. CG: 3<br><br>Uncontrolled major bleeding: n<br><br>IG: 2 vs. CG: 1<br><br>Other serious adverse events: n<br><br>IG: 9 vs. CG: 7<br><br>Deaths: n	**Reviewers' conclusion**<br><br>The clinical outcomes (mortality, morbidity) were secondary outcomes. The pilot RCT was not powered to detect differences in clinical outcomes.

Study: Reference, aim, design, setting	Participants: selection criteria, characteristics	N Participants; Intervention (IG) vs. Control group (CG)	Main outcomes	Assessment: LoE, risk of bias; Conclusions
			IG: 8 vs. CG: 3<br><br><u>Deaths due to bleeding: n</u><br><br>IG: 2 vs. CG: 1	
**Garrigue 2018**<br><br>"French lyophilized plasma versus fresh frozen plasma for the initial management of trauma-induced coagulopathy: a randomized open-label trial". *Journal of Thrombosis and Haemostasis* 2018, 16: 481–489<br><br>**Study design**<br><br>Randomised controlled trial<br><br>**Aim of the study**<br><br>"The aim of the study was to investigate whether, in trauma patients requiring immediate delivery of plasma at a high ratio, lyophilized plasma is more effective than FFP for the initial management of trauma-induced coagulopathy."<br><br>**Setting**<br><br>France, 2013-2016	**Inclusion criteria**<br><br>• Severely injured adult trauma patients<br>• admitted directly to the trauma center from the injury scene<br>• attending decided on immediate transfusion of an 'emergency pack' of 4 red blood cell units associated with 4 plasma units in a 1 : 1 ratio within 6 h of injury<br><br>**Exclusion criteria**<br><br>• age under 18 years<br>• transfusion of any blood product or coagulation factor concentrate prior to randomization<br>• admission from another healthcare facility<br>• devastating injuries and expected imminent death<br>• recent history of anticoagulant therapy<br>• known pregnancy<br>• lack of mental capacity per national legal standards prior to trauma<br><br>**Characteristics**<br><br><u>Age [y], mean ± SD</u><br><br>IG: 48.0 ± 16.5<br>CG: 38.0 ± 15.6<br><br><u>Males, n/N (%)</u><br><br>IG: 19/23 (82.6)<br>CG: 16/24 (66.7)<br><br><u>ISS, mean ± SD</u>	**Participants**<br><br>N=48 patients<br><br>**Study groups**<br><br>IG: FLyP, French lyophilized plasma (N=24 total, 23 analysed for primary and 21 for secondary outcomes)<br><br>**CG: FFP, fresh frozen plasma (N=24 total, 24 analysed for primary and 21 for secondary outcomes)**	**Primary outcome**<br><br><u>Fibrinogen concentration 45 minutes after randomization, mean ± SD, difference (95% CI)*</u><br><br>IG: 1.56 ± 0.81, p<0.001<br>CG: 0.93 ± 0.40<br>IG vs. CG: difference: 0.40 (0.23 to 0.57)<br><br>**Other outcomes**<br><br><u>30-day in-hospital mortality, n/N (%)</u><br><br>IG: 5/21 (22), p=0.56<br>CG: 7/21 (29)<br><br><u>Hemostatic parameters from randomization to 45 minutes, mean ± SD, difference (95% CI)</u><br><br>PT ratio<br>IG: 1.46 ± 0.4, p<0.001<br>CG: 1.74 ± 0.5<br>IG vs. CG: difference: -0.28 (-0.43 to -0.13)<br><br>Factor II<br>IG: 59.35 ± 21, p<0.001<br>CG: 43.83 ± 16<br>IG vs. CG: difference: 10.5 (5.55 to 15.44)<br><br>Factor V<br>IG: 53.17 ± 28, p<0.001<br>CG: 32.83 ± 23<br>IG vs. CG: difference: 11.05 (5.89 to 16.21)<br><br>Serum Lactate<br>IG: 3.95 ± 1.7, p=0.24<br>CG: 5.74 ± 5.2<br>IG vs. CG: difference: -0.74 (1.99 to 0.52)	**Level of evidence**<br><br>1b<br><br>**Risk of bias**<br><br>Selection bias: +<br><br>Performance bias: –<br><br>Attrition bias: +<br><br>Detection bias: +<br><br>**Authors' conclusion**<br><br>"[…] FLyP is superior to FFP for faster plasma transfusion. FLyP induces a faster and greater fibrinogen increase and TIC improvement. FLyP is an attractive option for trauma management, especially when facing logistical issues such as combat casualties or civilian mass casualties related to terrorism or natural disasters."<br><br>**Reviewers' conclusion**<br><br>**There may be a risk of performance bias as individuals administering care could not be blinded due to the investigation of inherent characteristics of the studied products**

Study: Reference, aim, design, setting	Participants: selection criteria, characteristics	N Participants; Intervention (IG) vs. Control group (CG)	Main outcomes	Assessment: LoE, risk of bias; Conclusions
	IG: 23.5 ± 9.7<br>CG: 27.5 ± 11.4<br><br><u>GCS, median (IQR)</u><br>IG: 3 (3 – 15)<br>CG: 3 (3 – 13.5)<br><br><u>Tranexamic acid, n/N (%)</u><br>IG: 19/23 (82.6)<br>CG: 22/24 (91.7)		<u>Change in coagulation parameters in fibrinogen concentration, from randomization to 45 min and 6 h, mean (95% CI)</u><br><br>45 min<br>IG: 0.17 (0.02 to 0.31), p=0.006<br>CG: 0.12 (0.27 to 0.02)<br><br>6 h<br>IG: 1.18 (0.87 to 1.49), p=0.008<br>CG: 0.68 (0.37 to 0.98)<br><br><u>Requirement of fibrinogen concentrates 24h after randomization, median (IQR)</u><br>IG: 2 (0 - 3), p=0.052<br>CG: 3 (2 - 4)<br><br>* per protocol analysis and adj. on baseline value	
**Nascimento 2016**<br>"Fibrinogen in the initial resuscitation of severe trauma (FiiRST): a randomized feasibility trial", *British Journal of Anaesthesia* 2016; 117(6): 775–82<br><br>**Study design**<br><br>**Randomized controlled feasibility trial**<br><br>**Aim of the study**<br>"The aim of the study was to evaluate the feasibility, effect on plasma fibrinogen concentration and complications of early infusion of FC in trauma patients."	**Inclusion criteria**<br>• Adult (age>18 yr)<br>• severe trauma (blunt or penetrating)<br>• assessed by trauma team at institution<br>• identified as being at risk for significant haemorrhage as evidenced by systolic arterial pressure ≤100 mmHg and requiring uncrossmatched red blood cell (RBC) transfusion at any time from injury until 30min after hospital arrival<br><br>**Exclusion criteria**<br>• received any blood or blood products before admission to trauma center<br>• presented more than 6h after injury<br>• estimated body weight <50 kg<br>• known or suspected pregnancy<br>• catastrophic brain injury<br>• non- haemorrhagic shock<br>• underlying hereditary or acquired coagulopathy	**Participants**<br>N=50 patients<br><br>**Study groups**<br>IG: lyophilized fibrinogen concentrate (FC, 6 g) (N=25 randomized, N=21 analysed)<br><br>CG: Placebo (normal saline) (N=25 randomized, N=24 analyzed)<br><br>**Co-interventions**<br>blood product (plasma, platelet and cryoprecipitate) transfusion was ordered based on standard coagulation tests, as per our institution's massive haemorrhage protocol.	**Primary outcome**<br><br><u>Feasibility (proportion of subjects receiving study intervention (FC or placebo) within 1h of hospital admission), n/N (%):</u><br>**43/45 (95.6) (95% CI 86-99)**<br><br><u>Time to start of infusion [min]: mean ± SD$^{\$}$</u><br>IG: 51 ± 9 vs. CG: 59 ±8, p=0.6<br><br>**Other outcomes**<br><br><u>All-cause 28-day mortality: n/N (%) (RR, 95% CI)</u><br>ITT: IG: 2/25 (8) vs. 3/24 (12.5), p=0.67<br><br>PT: IG: 2/20 (10) vs. CG: 1/24 (4.2)<br><br>RR = 2.4 (-0.2 to 23)<br><br><u>Acute Kidney Injury: n/N (%) (RR, 95% CI)</u><br>IG: 3/21 (14.3) vs. CG: 2/24 (8.3)<br><br>RR = 1.7 (-0.3 to 9.3)<br><br><u>Multiple Organ Failure: n/N (%) (RR, 95% CI)</u>	**Level of evidence**<br>2b↓<br><br>**Risk of bias**<br>Selection bias: +<br><br>Performance bias: +<br><br>Attrition bias: –<br><br>Detection bias: +<br><br>**Authors' conclusion**<br><br>**"Infusion of 6 g of FC within 1 h of arrival is feasible and improves plasma fibrinogen concentration by approximately 1 g L^{-1} 1 in a population of trauma patients at risk of significant haemorrhage."**

Study: Reference, aim, design, setting	Participants: selection criteria, characteristics	N Participants; Intervention (IG) vs. Control group (CG)	Main outcomes	Assessment: LoE, risk of bias; Conclusions
**Setting**<br>Canada, 2014-2015	• known or suspected use of anticoagulant medications<br>• moribund and predicted to expire in a few h<br>**Characteristics**<br>Age (median, range)<br>IG: 48 (19–78) vs. CG: 28 (19–88), p=0.05<br>Males (%)[§]<br>IG: 77 vs. CG: 87<br>ISS (median, IQR)<br>IG: 25 (19–29) vs. CG: 23 (18–29)<br>GCS (median, IQR)<br>IG: 15 (14–15) vs. CG: 15 (12–15)<br>Acute Traumatic Coagulopathy (%)[§]<br>IG: 26 vs. CG: 18<br>Fibrinogen <2 g L-1 (%)[§]<br>IG: 53vs. CG: 54<br>[§] n=number of patients not reported		IG: 2/21 (9.5) vs. CG: 2/24 (8.3)<br>RR = 1.1 (-0.2 to 7.4)<br>Plasma fibrinogen concentration at 3 h [mg dL^{-1}]: mean ± SD[$]<br>IG 2.9 vs. CG: 1.8 (p<0.01)<br>[$]units unclear	**Reviewers' conclusion**<br>**The clinical outcomes were secondary outcomes. The RCT was not powered to detect differences in clinical outcomes. There may be low risk of selection bias as the study groups significantly differed regarding age only.**
+: low risk; –: high risk; ?: unclear risk; adj.: adjusted; AIS: Abbreviated Injury Scale; CG: control group; CI: Confidence Interval; d: days; E-FIT 1: Early fibrinogen concentrate therapy for major haemorrhage in trauma; FC: fibrinogen concentrate; FFP: fresh frozen plasma; FgC: fibrinogen concentrate; FLyP: French lyophilized plasma; GCS: Glasgow Coma Score; h: hours; IG: intervention group; IQR: Interquartile range; ISS: injury severity score; L: litres; LoE: level of evidence; m: months; MHP: major haemorrhage protocol; min: minutes; mmHg: millimetres of mercury; MODS: multiple organ dysfunction syndrome; PT: prothrombin time; RBC: red blood cells; RCT: randomised controlled trial; s: seconds; SBP: systolic blood pressure; SD: Standard deviation; TBI: traumatic brain injury; TIC: trauma-induced coagulopathy; UK: United Kingdom; y: years				

Thromboprophylaxis

Study: Reference, aim, design, setting	Participants: selection criteria, characteristics	N Participants; Intervention (IG) vs. Control group (CG)	Main outcomes	Assessment: LoE, risk of bias; Conclusions
**Byrne (2016)**<br>"Timing of pharmacologic venous thromboembolism	**Inclusion criteria**<br>• adult patients (16 years or older)	**Participants**	Mortality: n (%)[§]<br>IG: 121 (9.8) vs. CG: 111 (9.0)	**Level of evidence**<br>2b

Study: Reference, aim, design, setting	Participants: selection criteria, characteristics	N Participants; Intervention (IG) vs. Control group (CG)	Main outcomes	Assessment: LoE, risk of bias; Conclusions
prophylaxis in severe traumatic brain injury: a propensity-matched cohort study." *Journal of the American College of Surgeons* 2016; 223(4): 621-631.<br><br>**Study design**<br>Comparative registry trail<br>(American College of Surgeons TQIP)<br><br>**Aim of the study**<br>"The purpose of this study was to compare the effectiveness of early vs late VTE prophylaxis in patients with sTBI, and to characterize the risk of subsequent intracranial hemorrhage (ICH)-related complication"<br><br>**Setting**<br>North America, 2012-2014	• isolated severe TBI (defined as head Abbreviated Injury Scale [AIS] ≥3 and Glasgow Coma Scale ≤8)<br>• received VTE prophylaxis with either low-molecular-weight heparin (LMWH) or unfractionated heparin (UH)<br>• survived at least 5 days<br><br>**Exclusion criteria**<br>• Trauma centers missing >10% of VTE prophylaxis data<br>• penetrating injuries<br>• bleeding disorders<br>• or severe injury (AIS 3) to other body regions<br><br>**Characteristics of the matched cohort**<br><br><u>Age [y], median (IQR)</u><br>IG: 43 (27-57) vs. CG: 43 (28-58)<br><br><u>Male, %</u><br>IG: 77.0 vs. CG: 75.8<br><br><u>ED total GCS, median (IQR)</u><br>IG: 3 (3-6) vs. CG: 3 (3-6)<br><br><u>ED motor GCS, median (IQR)</u><br>IG: 1 (1-4) vs. CG: 1 (1-4)<br><br><u>VTE prophylaxis with Low-molecular-weight heparin (vs. unfractionated heparin), %</u><br>IG: 48.2 vs. CG: 47.7	N=2,468 patients (after matching) (N=3,634 pre-matching)<br><br>**Study groups**<br>IG: early venous thromboembolism prophylaxes [<72 h] (N=1,234)<br><br>CG: late venous thromboembolism prophylaxes [>72 h] (N=1,234)<br><br>**Matching criteria**<br>• age<br>• sex<br>• race<br>• insurance type<br>• comorbidities<br>• transfer status<br>• injury mechanism<br>• injury severity (as measured by head AIS score),<br>• total GCS<br>• GCS motor score<br>• shock in the emergency department (SBP)<br>• early blood transfusion (within 12 hours of arrival at hospital)<br>• need for early neurosurgical intervention (within 48 hours of hospital arrival)<br>• choice of prophylaxis agent (UH or LMWH)	Adj. OR (95% CI): 1.10 (0.84-1.45)<br><br><u>Venous thromboembolism: adj. OR (95% CI)</u><br>0.48 (0.35-0.66)<br><br><u>Pulmonary embolism: n (%)</u><br>IG: 14 (1.1) vs. CG: 29 (2.4),<br>Adj. OR (95% CI): 0.48 (0.25-0.91)<br><br><u>Deep vein thrombosis: n (%)</u><br>IG: 52 (4.2) vs. CG: 98 (7.9)<br>Adj. OR (95% CI): 0.51 (0.36-0.72)<br><br><u>Craniotomy/ craniotomy after 72h: n (%)§</u><br>IG: 31 (2.5) vs. CG: 36 (2.9)<br>Adj. OR (95% CI): 0.86 (0.53-1.40)<br><br><u>Intracranial monitor placement after 72h: n (%)§</u><br>IG: 13 (1.1) vs. CG: 17 (1.4)<br>Adj. OR (95% CI): 0.76 (0.37-1.58)<br><br>§ Late neurosurgical interventions defined as those occurring after 72 hours in hospital.	**Risk of bias**<br>Selection bias: +<br>Performance bias: ?<br>Attrition bias: +<br>Detection bias: +<br><br>**Authors' conclusion**<br>"In this observational study of patients with sTBI, early initiation of VTE prophylaxis was associated with decreased risk of pulmonary embolism and deep vein thrombosis, but no increase in risk of late neurosurgical intervention or death. Early prophylaxis may be safe and should be the goal for each patient in the context of appropriate risk stratification."<br><br>**Reviewers' conclusion**<br>There is a risk of performance bias because physicians were probably not blinded. However, cohorts were matched according to confounders, early transfusions and VTE prophylaxis agent showing consistent results.
**Schellenberg (2021)**<br>"When Is It Safe to Start Pharmacologic Venous Thromboembolism Prophylaxis After Pelvic	**Inclusion criteria**<br>• Patients who sustained a pelvic fracture<br><br>**Exclusion criteria**	**Participants**<br>N=146 patients<br><br>**Study groups**	**Adjusted outcomes**<br><br><u>Venous thromboembolism: adj. OR (95% CI)</u><br>IG: 0.647 (0.002-4.510), p=0.999<br>CG: reference	**Level of evidence**<br>3b↓<br><br>**Risk of bias**

Study: Reference, aim, design, setting	Participants: selection criteria, characteristics	N Participants; Intervention (IG) vs. Control group (CG)	Main outcomes	Assessment: LoE, risk of bias; Conclusions
Fractures? A Prospective Study From a Level I Trauma Center." *Journal of Surgical Research* 2021; 258: 272-277.<br><br>**Study design**<br>Prospective observational study<br><br>**Aim of the study**<br>"The objective of this study was to determine if pharmacologic VTE prophylaxis initiation at ≤48 h of hospital arrival reduced the rates of VTE (defined as deep vein thrombosis [DVT] or pulmonary embolism [PE]) after blunt pelvic fracture. In addition, this study aimed to address the safety of early prophylaxis (EP), determining if EP resulted in potential bleeding complications, need for blood transfusion, or need for delayed intervention for hemorrhage control."<br><br>**Setting**<br>USA, 2016-2017	• death in the emergency department (ED)<br>• need for emergent operative intervention, defined as disposition from the ED directly to the operating room<br>• transfer from an outside hospital<br>• preexisting bleeding disorder<br>• home antiplatelet or anticoagulant medication; pregnancy<br>• patients who did not receive pharmacologic VTE prophylaxis during hospitalization<br><br>**Characteristics**<br><u>Age [y], median (IQR)</u><br>IG: 44 (29-57) vs. CG: 42 (29-56), p=0.697<br><u>Male, n (%)</u><br>IG: 36 (49) vs. CG: 39 (54), p=0.513<br><u>ISS, median (IQR)</u><br>IG: 14 (9-19) vs. CG: 17 (12-22), p=0.025<br><u>GCS, median (IQR)</u><br>IG: 15 (15-15) vs. CG: 15 (14-15), p=0.009<br><u>TBI, n (%)</u><br>IG: 2 (3) vs. CG: 23 (32), p<0.001<br><u>Type of prophylaxis heparin, n (%)</u><br>IG: 8 (11) vs. CG: 6 (8), p=0.780	IG: early VTE prophylaxis (≤48 h) (N=74)<br><br>CG: late VTE prophylaxes (>48 h) (N=72)<br><br>Prophylaxis was delivered as enoxaparin 30 mg subcutaneously every 12 h or unfractionated heparin 5000 units subcutaneously every 8 h, with unfractionated heparin administration reserved for patients with acute or chronic renal failure<br><br>**Co-interventions**<br>All patients without contraindication (e.g., lower extremity fracture) received mechanical VTE prophylaxis (intermittent pneumatic compression devices).<br><br>**Adjusting variables in multivariate logistic regression**<br>• timing of VTE prophylaxis initiation<br>• traumatic brain injury (TBI), defined by the presence of at least one of subarachnoid hemorrhage, subdural hematoma, or epidural hematoma<br>• sex<br>• lower extremity fracture<br>• solid organ injury<br>• ISS<br>• need for angioembolization	<u>Unadjusted outcomes</u><br>Mortality: n (%)<br>IG: 1 (1) vs. CG: 1 (1), p=1.000<br><u>Venous thromboembolism: n (%)</u><br>IG: 3 (4) vs. CG: 6 (8), p=0.323<br><u>Deep vein thrombosis: n (%)</u><br>IG: 0 (0) vs. CG: 5 (7), p=0.027<br><u>Pulmonary embolism: n (%)</u><br>IG: 3 (4) vs. CG: 2 (3), p=1.000<br><u>Hospital length of stay: median (IQR)</u><br>IG: 11 (5-19) vs. CG: 18 (8-47), p=0.007<br><u>ICU length of stay: median (IQR)</u><br>IG: 3 (0-4) vs. CG: 5 (2-9), p=0.005	Selection bias: –<br>Performance bias: ?<br>Attrition bias: +<br>Detection bias: +<br><br>**Authors' conclusion**<br>"Early initiation of pharmacologic VTE prophylaxis after blunt pelvic fracture is safe. Although early prophylaxis initiation did not reduce the rate of VTE, these data identify angioembolization as an independent risk factor for VTE. Patients with blunt pelvic fracture who undergo angioembolization may therefore represent a high-risk population who may especially benefit from early prophylaxis."<br><br>**Reviewers' conclusion**<br>There is a risk of selection bias because patients in the control group had a higher ISS and rate of TBI and secondary outcomes were not adjusted for important risk factors. There is a risk of performance bias because physicians were not blinded.<br><br>The study was underpowered to detect clinically significant effects

Study: Reference, aim, design, setting	Participants: selection criteria, characteristics	N Participants; Intervention (IG) vs. Control group (CG)	Main outcomes	Assessment: LoE, risk of bias; Conclusions
				on VTE events and mortality.<br>(cohort overlaps with Schellenberg 2019)
**Schellenberg (2019)**<br>"When is it safe to start VTE prophylaxis after blunt solid organ injury? A prospective study from a level I trauma Center." *World journal of surgery* 2019; 43(11): 2797-2803.<br>**Study design**<br>Prospective observational study<br>**Aim of the study**<br>"The primary objective of this study was to prospectively determine the optimal timing of VTE prophylaxis initiation among patients with blunt solid organ injury managed nonoperatively."<br>**Setting**<br>USA, 2016-2017	**Inclusion criteria**<br>• sustained a solid organ injury (liver, spleen, and/or kidney) managed nonoperatively (defined by a documented plan in the ED by the trauma team for nonoperative management and the lack of laparotomy within 4 h of admission)<br>• age >15<br>• blunt trauma patients LAC+USC Medical Center<br>**Exclusion criteria**<br>• transferred from an outside hospital<br>• died in the emergency department (ED)<br>• had a pre-existing bleeding disorder<br>• were on home antiplatelet<br>• anticoagulation medication<br>• received no VTE prophylaxis during their hospital admission<br>**Characteristics**<br>Age [y], median (IQR)<br>IG: 36 (27–54) vs. CG: 36 (27–56), p=0.631<br>Male, n (%)<br>IG: 39 (64) vs. CG: 39 (68), p=0.698<br>GCS, median (IQR)<br>IG: 15 (14–15) vs. CG: 14 (13–15), p=0.009<br>ISS, median (IQR)	**Participants**<br>N=118 patients<br>**Study groups**<br>IG: early VTE prophylaxis (≤48 h) (N=61)<br>CG: late VTE prophylaxis (>48 h) (N=57)<br>**Co-interventions**<br>All patients without contraindication (e.g., lower extremity fracture) received sequential compression devices to bilateral lower extremities until ambulation.	Mortality: n (%)<br>IG: 2 (3) vs. CG: 1 (2), p=1.000<br>Venous thromboembolic event: n (%)<br>IG: 2 (3) vs. CG: 6 (11), p=0.153<br>Deep vein thrombosis: n (%)<br>IG: 0 (0) vs. CG: 5 (9), p=0.024<br>Pulmonary embolism: n (%)<br>IG: 2 (3) vs. CG: 3 (5), p=0.672<br>Hospital length of stay: median (IQR)<br>IG: 6 (4–11) vs. CG: 14 (7–35), p<0.001<br>Need for ICU admission: n (%)<br>IG: 52 (85) vs. CG: 52 (91), p=0.398<br>ICU length of stay: median (IQR)<br>IG: 3 (2–6) v. CG: 7 (4–12), p<0.001	**Level of evidence**<br>2b<br>**Risk of bias**<br>Selection bias: –<br>Performance bias: ?<br>Attrition bias: +<br>Detection bias: +<br>**Authors' conclusion**<br>"In this prospective study of patients with nonoperative blunt solid organ injuries, early (≤48 h) initiation of VTE prophylaxis resulted in a lower incidence of DVTs without an associated increase in bleeding or need for intervention. Early initiation of VTE prophylaxis is likely to be safe and beneficial for patients with blunt solid organ injury."<br>**Reviewers' conclusion**<br>There is a risk of selection bias since patients in the control group had a higher ISS and rate of TBI, and the analysis is unadjusted. There is a risk of performance bias because

Study: Reference, aim, design, setting	Participants: selection criteria, characteristics	N Participants; Intervention (IG) vs. Control group (CG)	Main outcomes	Assessment: LoE, risk of bias; Conclusions
	IG: 17 (14–22) vs. CG: 22 (17–27), p=0.002<br><br><u>TBI, n (%)</u><br>IG: 5 (8) vs. CG: 18 (32). p=0.002			physicians were not blinded.<br><br>(cohort overlaps with Schellenberg 2021)
+: low risk; –: high risk; ?: unclear risk; adj.: adjusted; AIS: Abbreviated Injury Scale; CG: control group; CI: Confidence Interval; d: days; DVT: deep vein thrombosis; ED: emergency department; GCS: Glasgow Coma Score; h: hours; ICU: intensive care unit; ICH: intracranial haemorrhage; IG: intervention group; IQR: Interquartile Range; ISS: injury severity score; ITT: Intention to Treat analysis; LMWH: low-molecular-weight heparin; LoE: level of evidence; m: months; MAP: mean arterial pressure; min: minutes; mmHg: millimetres of mercury; MODS: multiple organ dysfunction syndrome; OR: odds ratio; PE: pulmonary embolism; RR: Relative Risk; s: seconds; SBP: systolic blood pressure; SD: Standard deviation; sTBI: severe traumatic brain injury; TBI: traumatic brain injury; UH: unfractionated heparin; VTE: venous thromboembolism; USA: United States of America; y: years				

Access routes

Study: Reference, aim, design, setting	Participants: selection criteria, characteristics	N Participants; Intervention (IG) vs. Control group (CG)	Main outcomes	Assessment: LoE, risk of bias; Conclusions
**Kunhahamed (2019)**<br>"A comparison of internal jugular vein cannulation by ultrasound-guided and anatomical landmark technique in resource-limited emergency department setting", *Journal of Medical Ultrasound* 2019; 27: 187-91.<br><br>**Study design**<br>Prospective observational study<br><br>**Aim of the study**<br>"The aim of the study was to measure and compare the success rate, time to completion, number of central venous access attempts, and acute	**Inclusion criteria**<br>• ≥18 years<br>• presented to the ED<br>• in need of central venous access through internal jugular vein (IJV) as part of their treatment<br><br>**Exclusion criteria**<br>• Patients <18 years<br>• patients with suspected cervical spine injury or penetrating injury to the neck<br>• patients with coagulopathy<br>• local site infections or burns<br>• head-and-neck cancer patients<br><br>**Characteristics**<br><u>Age [y], mean ± SD</u><br>IG: 46.74 ± 16.36<br>CG: 50.41 ±17.93<br><br><u>Sex, n/N (%)</u>	**Participants**<br>N=70 patients<br><br>**Study groups**<br>IG: Ultrasonography-guided technique technique (N=35)<br><br>CG: Anatomical Landmark (AL) technique (N=35)	**Primary outcome**<br><u>Successful cannulations, n/N (%)</u><br>IG: 35/35 (100) vs. CG: 32/35 (91.4), p=0.239<br><br>**Other outcomes**<br><u>Number of attempts for successful cannulation, n/N (%)</u><br>1 attempt<br>IG: 32/35 (91.4) vs. CG: 17/35 (48.6), p<0.001<br><br>2 attempts<br>IG: 3/35 (8.6) vs. CG: 10/35 (28.6)<br><br>3 attempts<br>IG: 0/35 (0) vs. CG: 8/35 (22.9)<br><br><u>Cannulation time [sec]: mean ± SD</u><br>IG: 293.03 ± 71.15 vs. CG: 305.88 ± 66.84, p=0.425<br><br>Defined as time interval between observing blood at the syringe hub and confirming backflow of blood at all three ports in the triple lumen catheter.	**Level of evidence**<br>3b↓<br><br>**Risk of bias**<br>Selection bias: ?<br><br>Performance bias: ?<br><br>Attrition bias: +<br><br>Detection bias: +<br><br>**Authors' conclusion**<br>"The real-time USG guided technique significantly reduces the number of attempts to cannulate, has a higher first-pass success rate, a quicker flash time, and fewer complications when compared to the AL technique."<br><br>**Reviewers' conclusion**

Study: Reference, aim, design, setting	Participants: selection criteria, characteristics	N Participants; Intervention (IG) vs. Control group (CG)	Main outcomes	Assessment: LoE, risk of bias; Conclusions
complications during IJV catheterization by the AL technique and real-time USG-guided technique in emergency department (ED) setting."<br><br>**Setting**<br><br>India, 2017-2018	IG: 21/35 (60) male, 14/35 (40) female<br>CG: 17/35 (49) male, 18/35 (51) female<br><br>Provisional Diagnosis, n/N (%)<br><br>Polytrauma with haemorrhagic shock<br><br>IG: 8/35 (22.9) vs. CG: 4/35 (11.4)<br><br>Sepsis/Septic shock<br><br>IG: 8/35 (22.9) vs. CG: 10/35 (28.6)<br><br>Acute respiratory distress syndrome<br><br>IG: 2/35 (5.7) vs. CG: 2/35 (5.7)<br><br>Burns<br><br>IG: 6 /35 (17.1) vs. CG: 8/35 (22.9)<br><br>Diabetic ketoacidosis<br><br>IG: 6 /35 (17.1) vs. 3/35 (8.6)		Flash time [sec]: mean ± SD<br><br>IG: 4.86 ± 2.18 vs. CG: 16.59 ± 10.67, p<0.001<br><br>defined as the time interval between skin puncture and observing blood at the syringe hub.	There might be a risk of selection bias because it was up to the physician to decide for the method of cannulation. In addition, there might be a risk of performance bias as sufficient information on patient allocation and blinding is lacking.<br><br>**CAVE: only 12% trauma patients.** It was included because this was the only study identified for the new questions.
+: low risk; –: high risk; ?: unclear risk; AL: anatomical landmark; CG: control group; CI: Confidence Interval; ED: emergency department; IG: intervention group; IJV: internal jugular vein; ISS: injury severity score; LoE: level of evidence; sec: seconds; SD: Standard deviation; TBI: traumatic brain injury; USG: real-time ultrasonography				

Table S5. Deleted Recommendations

Number	Recommendation (German)	Reason
2.115<br>B	Bei Blutung sollte eine Substitution von Fibrinogen bei thrombelastometrischen Zeichen eines funktionellen Fibrinogendefizites oder Werten von <1,5 g/l (150 mg/dl) durchgeführt werden.	Replaced by 2.4.20
